# Supplementary material for: Chemoselective catalytic hydrodefluorination of trifluoromethylalkenes towards mono-/gem-di-fluoroalkenes under metal-free conditions
Source: Nat Commun. 2021 May 14;12:2835. doi: 10.1038/s41467-021-23101-3 (PMC8121801; doi:10.1038/s41467-021-23101-3)
Supplement: Supplementary file 1 — Supplementary Information [file 41467_2021_23101_MOESM1_ESM.pdf]

Supplementary Information

Chemoselective Catalytic Hydrodefluorination of Trifluoromethylalkenes towards Mono-/*gem*-Di-Fluoroalkenes under Metal-free Conditions

Zhang *et al.*

Supplementary Methods

General information

Chemicals: Catalyst **1** has been synthesized and characterized in our previous paper.[1] Trifluoromethyl alkenes **2** were synthesized according to references. Other reagents and solvent were purchased from J&K or TCI Chemicals and used without further purification unless specified otherwise. Acetonitrile was purchased from J&K Chemical (99.9 %, Extra dry, water < 10 ppm, J&K seal) and degassed and distilled by standard methods. Reaction temperature refers to the temperature of an aluminum heating block or a silicon oil bath, which was controlled by an electronic temperature modulator from IKA.

Reactions: All hydrodefluorination reactions were carried out in dry glass wares under an argon atmosphere using Schlenk technique throughout the reaction procedures.

Analytics: ^{1}H and ^{13}C NMR, ^{19}F NMR spectra were recorded in $CDCl_3$ (δ = 7.26 for ^{1}H NMR, δ = 77.16 ppm for ^{13}C NMR) on 400 MHz NMR instrument at Center of Basic Molecular Science (CBMS) of Tsinghua University.

DFT calculations: Geometry optimizations and frequency computations were performed using Gaussian 09[2] at the M06-2X[3, 4]/6-31+G(d) level of theory, in conjunction with the SMD[5] model to account for the solvation effect of acetonitrile. To obtain more accurate electronic energies, single point energy calculations were performed at the SMD-M06-2X/[6-311++G(2df, 2p)] level with the SMD-M06-2X/[6-31+G(d)] structures.

Experimental procedure

The reaction of trifluoromethyl alkene 2a with stoichiometric catalyst 1

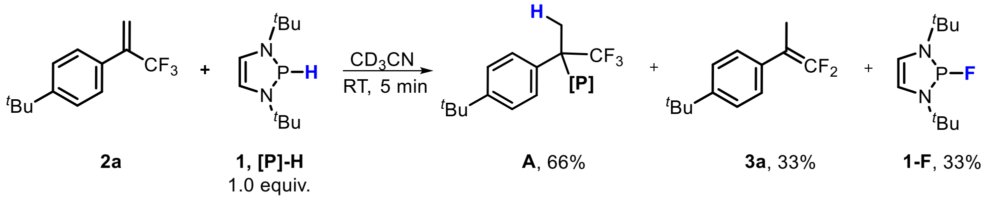

(S1)

Trifluoromethyl alkene **2a** (0.5 mmol), diazaphospholene **1** (0.5 mmol) and CD_3CN (1.0 mL) were taken in a Schlenk tube under argon. The mixture was stirred at room temperature for 5 minutes. The reaction were monitored by ^{19}F NMR and ^{31}P NMR spectra, confirming the generation of intermediate **A** as the major product with a small number of **3a** and **1-F**. The ^{19}F NMR and ^{31}P NMR spectra were shown as below:

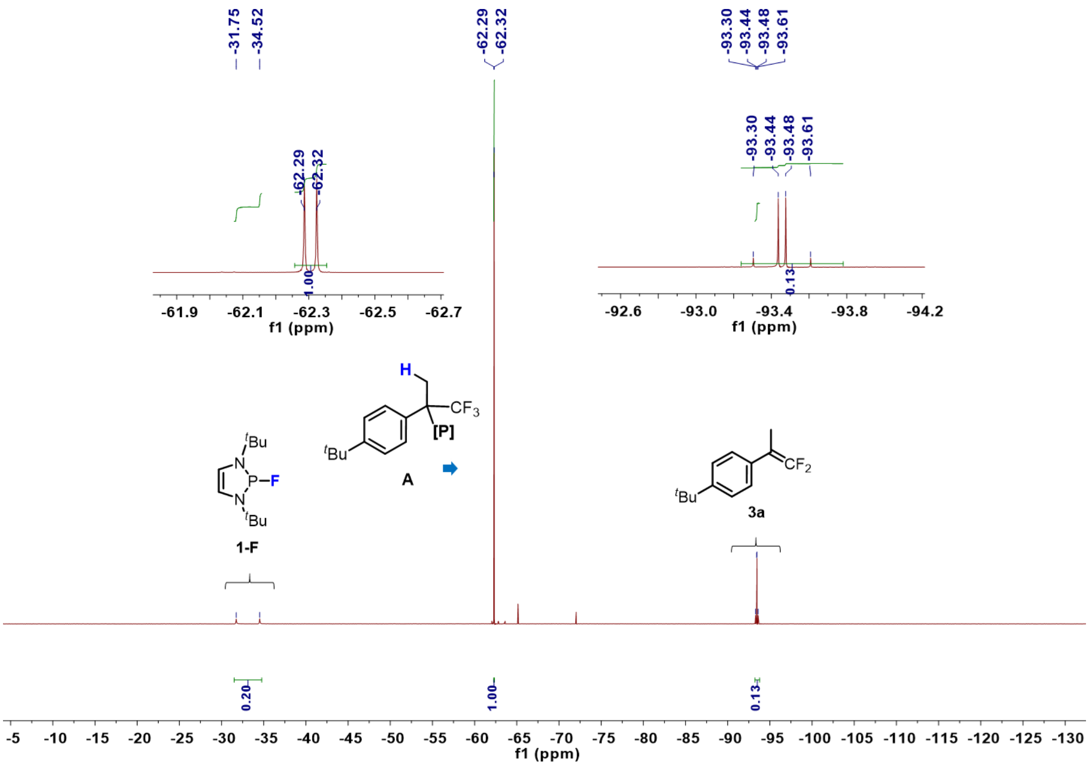

Supplementary Figure 1. The ^{19}F NMR spectrum (with ^{1}H decoupling) of the reaction mixture in CD_3CN.

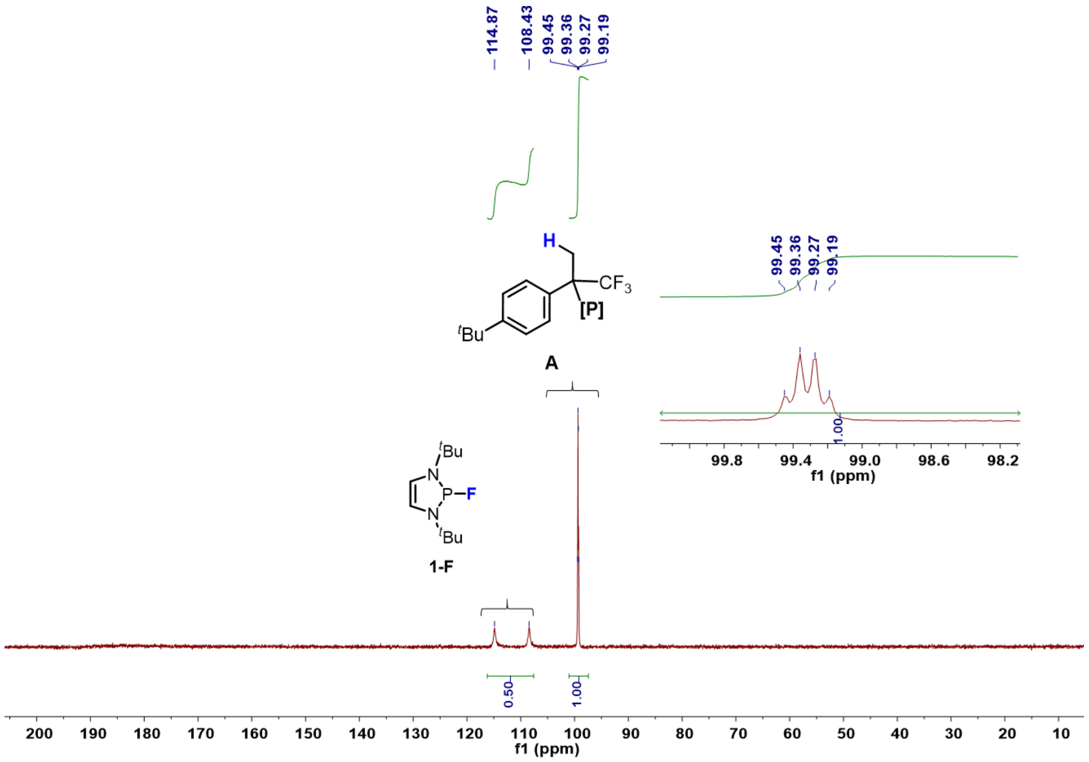

Supplementary Figure 2. The ^{31}P NMR spectrum (with ^{1}H decoupling) of the reaction mixture in CD_3CN.

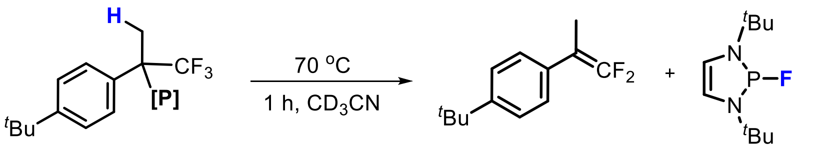

(S2)

A **3a**, quant. **1-F**, quant.

The mixture of Eq. S1 was stirred at 70 °C for one hour. The reaction were monitored by ^{19}F NMR and ^{31}P NMR spectra. The ^{19}F NMR and ^{31}P NMR spectra were shown as below:

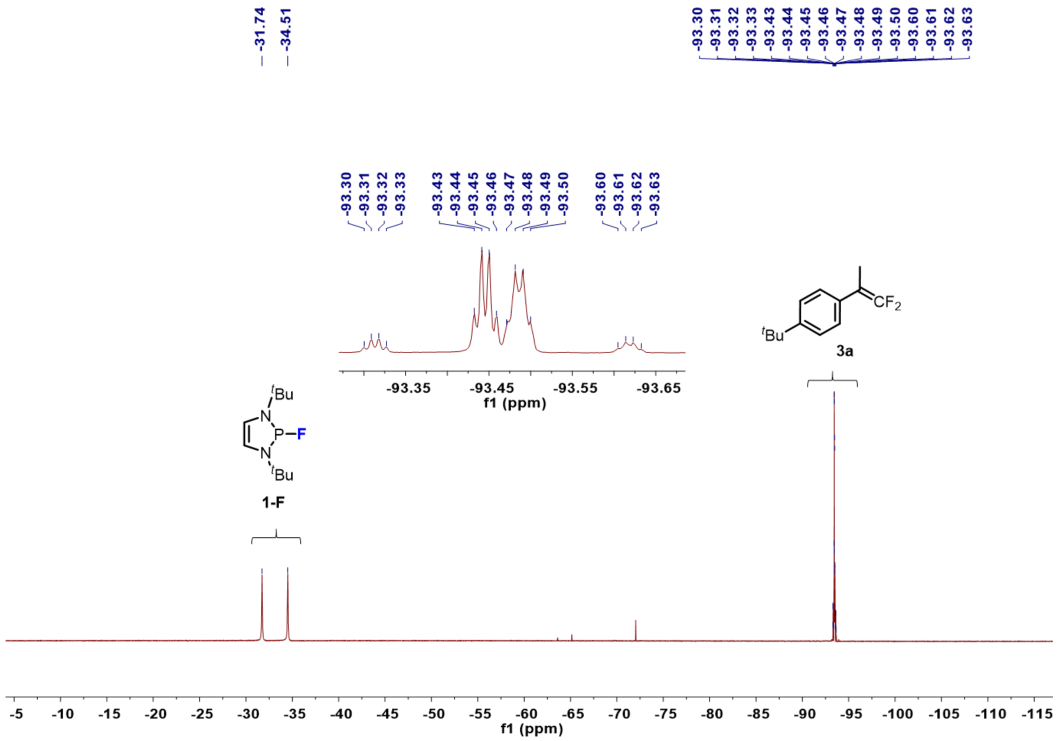

Supplementary Figure 3. The ^{19}F NMR spectrum of the reaction mixture in CD_3CN.

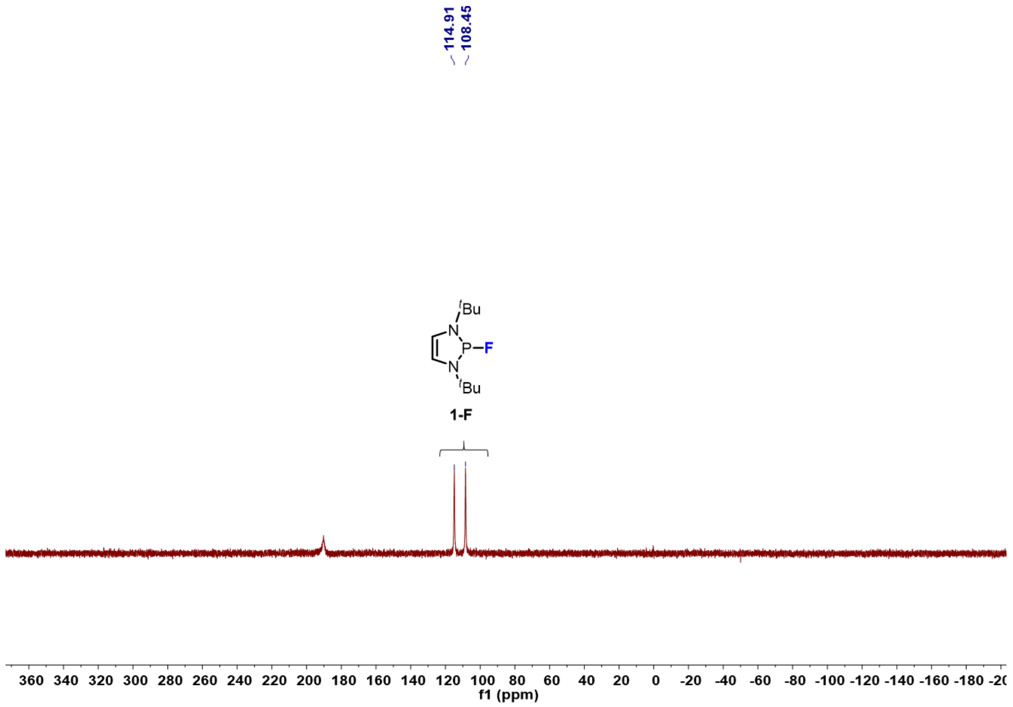

Supplementary Figure 4. The ^{31}P NMR spectrum of the reaction mixture in CD_3CN.

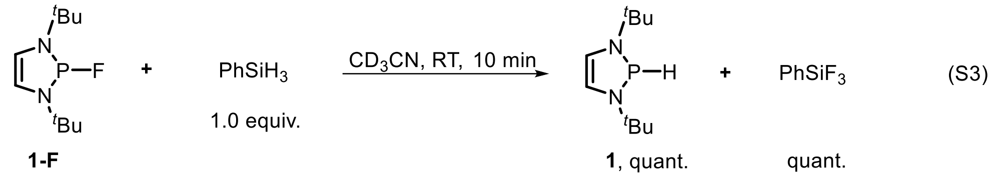

$PhSiH_3$ was added to the CD_3CN solution of **1-F** at room temperature for 10 minutes. The reactions were monitored by ^{31}P NMR spectra. The ^{31}P NMR spectrum was shown as below:

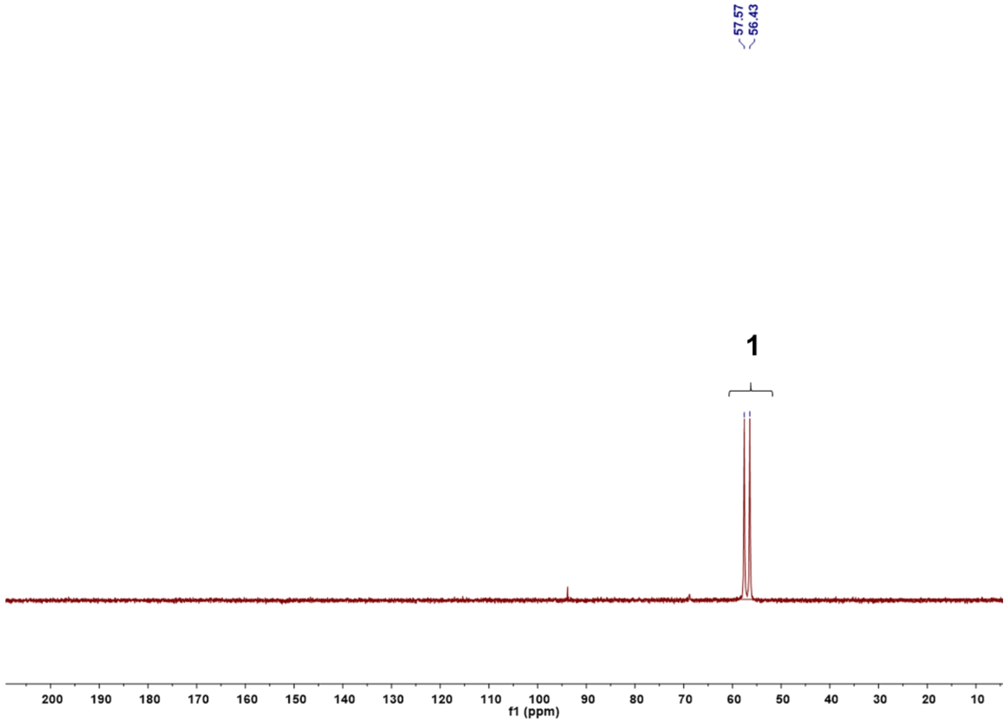

Supplementary Figure 5. The ^{31}P NMR spectrum of the reaction mixture in CD_3CN.

The synthesis and analysis of trifluoromethyl alkenes 2 and 5

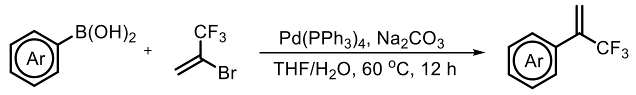

The trifluoromethyl alkenes **2** were synthesized according to reported methods.[6] The **2s** was obtained with a mixture of the olefins (*E*/*Z* 1/1), and the NMR spectra were consistent with that reported in the reference.[7]

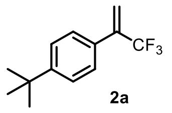

^{1}H NMR (400 MHz, $CDCl_3$) δ 7.46 (s, 4H), 5.96 (d, *J* = 1.2 Hz, 1H), 5.81 (d, *J* = 1.6 Hz, 1H), 1.39 (s, 9H). **^{19}F NMR** (376 MHz, $CDCl_3$) δ -64.68 (s). **^{13}C NMR** (101 MHz, $CDCl_3$) δ 152.1, 138.76 (q, *J* = 29.9 Hz), 130.7, 127.0, 125.5, 123.5 (q, *J* = 274.0 Hz), 119.6 (q, *J* = 5.8 Hz), 34.6, 31.2. The NMR spectra were in agreement with that reported in the reference.[8]

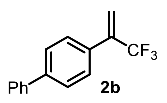

^{1}H NMR (400 MHz, $CDCl_3$) δ 7.69 – 7.67 (m, *J* = 5.3, 2.5 Hz, 4H), 7.60 (d, *J* = 8.2 Hz, 2H), 7.56 – 7.47 (m, 2H), 7.47 – 7.38 (m, 1H), 6.03 (d, *J* = 1.4 Hz, 1H), 5.88 (d, *J* = 1.7 Hz, 1H). **^{19}F NMR** (376 MHz, $CDCl_3$) δ -64.58. **^{13}C NMR** (101 MHz, $CDCl_3$) δ 141.9, 140.3, 138.6 (q, *J* = 30.0 Hz), 132.5, 128.9, 127.8, 127.7, 127.3, 127.1, 123.4 (q, *J* = 274.2 Hz), 120.2 (q, *J* = 5.8 Hz). The NMR spectra were in agreement with that reported in the reference.[8]

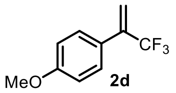

2d

^{1}H NMR (400 MHz, CDCl$_3$) δ 7.51 – 7.37 (m, 2H), 6.95 (d, *J* = 8.9 Hz, 2H), 5.91 (q, *J* = 1.3 Hz, 1H), 5.73 (q, *J* = 1.7 Hz, 1H), 3.86 (s, 3H). **^{19}F NMR** (376 MHz, CDCl$_3$) δ -64.85. **^{13}C NMR** (101 MHz, CDCl$_3$) δ 160.2, 138.4 (q, *J* = 29.8 Hz), 128.6, 126.0, 123.5 (q, *J* = 274.0 Hz), 118.8 (q, *J* = 5.8 Hz), 114.0, 55.3. The NMR spectra were in agreement with that reported in the reference.[9]

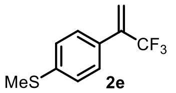

2e

^{1}H NMR (400 MHz, CDCl$_3$) δ 7.42 (d, *J* = 8.2 Hz, 2H), 7.28 (d, *J* = 8.5 Hz, 2H), 5.99 – 5.89 (m, 1H), 5.79 (d, *J* = 1.7 Hz, 1H), 2.53 (s, 3H). **^{19}F NMR** (376 MHz, CDCl$_3$) δ -64.71. **^{13}C NMR** (101 MHz, CDCl$_3$) δ 140.1, 138.4 (q, *J* = 29.9 Hz), 130.1, 127.6, 126.1, 123.4 (q, *J* = 274.0 Hz), 119.7 (q, *J* = 5.8 Hz), 15.4. The NMR spectra were in agreement with that reported in the reference.[10]

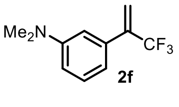

2f

^{1}H NMR (400 MHz, CDCl$_3$) δ 7.31 – 7.26 (m, 1H), 6.88 – 6.75 (m, 3H), 5.96 (q, *J* = 1.4 Hz, 1H), 5.79 (q, *J* = 1.7 Hz, 1H), 3.01 (s, 6H). **^{19}F NMR** (376 MHz, CDCl$_3$) δ -64.69. **^{13}C NMR** (101 MHz, CDCl$_3$) δ 150.6, 139.8 (q, *J* = 29.8 Hz), 134.6, 129.2, 123.5 (q, *J* = 274.1 Hz), 120.1 (q, *J* = 5.7 Hz), 115.7, 113.1, 111.5, 40.5.

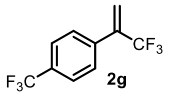

2g

^{1}H NMR (400 MHz, CDCl$_3$) δ 7.68 (d, *J* = 8.3 Hz, 2H), 7.60 (d, *J* = 8.2 Hz, 2H), 6.09 (d, *J* = 1.5 Hz, 1H), 5.87 (d, *J* = 1.7 Hz, 1H). **^{19}F NMR** (376 MHz, CDCl$_3$) δ -62.97, -64.94. **^{13}C NMR** (101 MHz, CDCl$_3$) δ 138.1 (q, *J* = 30.4 Hz), 137.1, 131.1 (q, *J* = 32.8 Hz), 127.8, 125.6 (q, *J* = 3.7 Hz), 123.9 (q, *J* = 272.1 Hz), 122.1 (q, *J* = 5.7 Hz), 123.0 (q, *J* = 273.9 Hz). The NMR spectra were in agreement with that reported in the reference.[9]

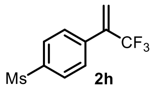

2h

^{1}H NMR (400 MHz, CDCl$_3$) δ 7.97 (d, *J* = 8.5 Hz, 2H), 7.73 – 7.59 (m, 2H), 6.12 (s, 1H), 5.91 (s, 1H), 3.08 (s, 3H). **^{19}F NMR** (376 MHz, CDCl$_3$) δ -64.74. **^{13}C NMR** (101 MHz, CDCl$_3$) δ 141.0, 138.9, 137.7 (q, *J* = 31.0 Hz), 128.4, 127.8, 122.8 (d, *J* = 274.1 Hz), 123.1 (q, *J* = 5.7 Hz), 44.5. The NMR spectra were in agreement with that reported in the reference.[11]

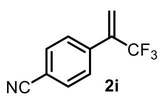

2i

^{1}H NMR (400 MHz, CDCl$_3$) δ 7.71 (d, *J* = 8.5 Hz, 2H), 7.59 (d, *J* = 8.2 Hz, 2H), 6.12 (d, *J* = 1.5 Hz, 1H), 5.91 (d, *J* = 1.7 Hz, 1H). **^{19}F NMR** (376 MHz, CDCl$_3$) δ -64.71. **^{13}C NMR** (101 MHz, CDCl$_3$) δ 137.9, 137.7 (q, *J* = 30.8 Hz),132.4, 128.1, 122.82 (q, *J* = 5.6 Hz), 122.80 (q, *J* = 273.9 Hz), 118.2, 112.9. The NMR spectra were in agreement with that reported in the reference.[12]

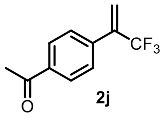

2j

^{1}H NMR (400 MHz, CDCl$_3$) δ 7.97 (d, *J* = 8.6 Hz, 2H), 7.65 – 7.47 (m, 2H), 6.05 (d, *J* = 1.4 Hz, 1H), 5.87 (d, *J* = 1.7 Hz, 1H), 2.61 (s, 3H). **^{19}F NMR** (376 MHz, CDCl$_3$) δ -64.67. **^{13}C NMR** (101 MHz, CDCl$_3$) δ 197.3, 138.2 (q, *J* = 30.4 Hz), 137.9, 137.2, 128.5, 127.5, 123.0 (q, *J* = 274.0 Hz), 121.9 (q, *J* = 5.7 Hz), 26.5. The NMR spectra were in agreement with that reported in the reference.[13]

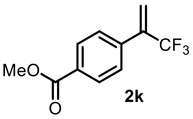

2k

^{1}H NMR (400 MHz, CDCl$_3$) δ 8.07 (d, *J* = 8.8 Hz, 2H), 7.55 (d, *J* = 8.0 Hz, 2H), 6.06 (d, *J* = 1.4 Hz, 1H), 5.88 (d, *J* = 1.7 Hz, 1H), 3.95 (s, 3H). **^{19}F NMR** (376 MHz, CDCl$_3$) δ -64.69. **^{13}C NMR** (101 MHz, CDCl$_3$) δ 166.5, 138.3 (q, *J* = 30.4 Hz), 137.8, 130.6, 129.8, 127.4, 123.1 (q, *J* = 274.0 Hz), 121.8 (q, *J* = 5.7 Hz), 52.2. The NMR spectra were in agreement with that reported in the reference.[8]

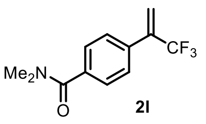
2l

^{1}H NMR (400 MHz, $CDCl_3$) δ 7.50 (d, *J* = 8.2 Hz, 2H), 7.45 (d, *J* = 8.4 Hz, 2H), 6.01 (d, *J* = 1.4 Hz, 1H), 5.82 (d, *J* = 1.7 Hz, 1H), 3.13 (s, 3H), 3.00 (s, 3H). **^{19}F NMR** (376 MHz, $CDCl_3$) δ -64.77. **^{13}C NMR** (101 MHz, $CDCl_3$) δ 170.8, 138.3 (q, *J* = 30.3 Hz), 136.9, 134.7, 127.4, 127.3, 123.1 (q, *J* = 274.0 Hz), 121.2 (q, *J* = 5.8 Hz), 39.5, 35.3. The NMR spectra were in agreement with that reported in the reference.[14]

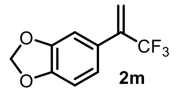
2m

^{1}H NMR (400 MHz, $CDCl_3$) δ 7.03 – 6.95 (m, 2H), 6.88 – 6.79 (m, 1H), 6.02 (s, 2H), 5.91 (d, *J* = 1.3 Hz, 1H), 5.71 (d, *J* = 1.7 Hz, 1H). **^{19}F NMR** (376 MHz, $CDCl_3$) δ -64.90. **^{13}C NMR** (101 MHz, $CDCl_3$) δ 148.3, 147.9, 138.5 (q, *J* = 29.9 Hz), 127.5, 123.3 (q, *J* = 273.9 Hz), 121.5, 119.6 (q, *J* = 5.7 Hz), 108.3, 107.8, 101.4. The NMR spectra were in agreement with that reported in the reference.[11]

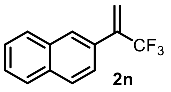
2n

^{1}H NMR (400 MHz, $CDCl_3$) δ 7.99 (s, 1H), 7.93 – 7.87 (m, 3H), 7.65 – 7.59 (m, 1H), 7.60 – 7.52 (m, 2H), 6.10 (d, *J* = 1.4 Hz, 1H), 5.94 (d, *J* = 1.7 Hz, 1H). **^{19}F NMR** (376 MHz, $CDCl_3$) δ -64.39. **^{13}C NMR** (101 MHz, $CDCl_3$) δ 139.0 (q, *J* = 30.0 Hz), 133.3, 133.1, 130.9, 128.5, 128.3, 127.6, 127.0, 126.8, 126.6, 123.5 (q, *J* = 274.0 Hz), 124.7, 120.7 (q, *J* = 5.8 Hz). The NMR spectra were in agreement with that reported in the reference.[15]

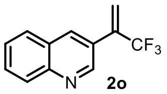
2o

^{1}H NMR (400 MHz, $CDCl_3$) δ 9.00 (d, *J* = 2.3 Hz, 1H), 8.35 – 8.21 (m, 1H), 8.14 (d, *J* = 8.5 Hz, 1H), 7.86 (dd, *J* = 8.3, 1.3 Hz, 1H), 7.77 (ddd, *J* = 8.4, 6.8, 1.4 Hz, 1H), 7.60 (td, *J* = 7.4, 6.8, 1.1 Hz, 1H), 6.17 (d, *J* = 1.6 Hz, 1H), 5.99 (d, *J* = 1.7 Hz, 1H). **^{19}F NMR** (376 MHz, $CDCl_3$) δ -64.97. **^{13}C NMR** (101 MHz, $CDCl_3$) δ 148.9, 147.9, 136.3 (q, *J* = 30.9 Hz), 134.4, 130.3, 129.3, 128.3, 127.4, 127.1, 126.4, 123.1 (q, *J* = 274.1 Hz), 122.1 (q, *J* = 5.7 Hz). The NMR spectra were in agreement with that reported in the reference.[16]

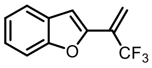
2p

^{1}H NMR (400 MHz, $CDCl_3$) δ 7.62 (dt, *J* = 7.7, 1.0 Hz, 1H), 7.52 (dq, *J* = 8.3, 0.9 Hz, 1H), 7.38 (ddd, *J* = 8.3, 7.3, 1.3 Hz, 1H), 7.28 (td, *J* = 7.5, 1.0 Hz, 1H), 6.93 (s, 1H), 6.34 (d, *J* = 1.8 Hz, 1H), 6.03 (d, *J* = 1.3 Hz, 1H). **^{19}F NMR** (376 MHz, $CDCl_3$) δ -65.58. **^{13}C NMR** (101 MHz, $CDCl_3$) δ 154.7, 148.5, 129.4 (q, *J* = 32.3 Hz), 128.4, 125.7, 123.3, 122.4 (q, *J* = 273.1 Hz), 121.8, 118.1 (q, *J* = 5.3 Hz), 111.1, 106.3. The NMR spectra were in agreement with that reported in the reference.[17]

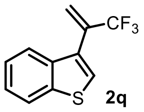
2q

^{1}H NMR (400 MHz, $CDCl_3$) δ 7.99 – 7.93 (m, 1H), 7.92 – 7.87 (m, 1H), 7.60 (d, *J* = 1.1 Hz, 1H), 7.54 – 7.36 (m, 2H), 6.32 (d, *J* = 1.5 Hz, 1H), 5.90 (d, *J* = 1.5 Hz, 1H). **^{19}F NMR** (376 MHz, $CDCl_3$) δ -66.51. **^{13}C NMR** (101 MHz, $CDCl_3$) δ 140.0, 138.1, 133.1 (q, *J* = 31.6 Hz), 128.7, 126.3, 124.8, 124.7, 122.7 (q, *J* = 5.5 Hz), 122.9, 122.7 (q, *J* = 231.0 Hz), 122.5. The NMR spectra were in agreement with that reported in the reference.[18]

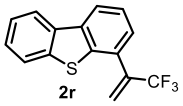
2r

^{1}H NMR (400 MHz, $CDCl_3$) δ 8.25 – 8.11 (m, 2H), 7.96 – 7.81 (m, 1H), 7.62 – 7.44 (m, 4H), 6.38 (d, *J* = 1.5 Hz, 1H), 6.10 (d, *J* = 1.5 Hz, 1H). **^{19}F NMR** (376 MHz, $CDCl_3$) δ -65.85. **^{13}C NMR** (101 MHz, $CDCl_3$) δ 140.1, 139.2, 137.8 (q, *J* = 31.2 Hz), 136.4, 135.7, 128.7, 127.1, 126.5, 124.6, 124.6, 123.7 (q, *J* = 5.4 Hz), 123.0 (q, *J* = 274.2 Hz), 122.7, 122.0, 121.8. The NMR spectra were in agreement with that reported in the reference.[19]

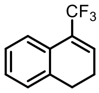

2t

^{1}H NMR (400 MHz, $CDCl_3$) δ 7.55 – 7.49 (m, 1H), 7.35 – 7.21 (m, 4H), 6.81 – 6.77 (m, 1H), 2.88 (t, *J* = 8.1 Hz, 2H), 2.49 (ddd, *J* = 8.6, 5.1, 2.6 Hz, 2H). **^{19}F NMR** (376 MHz, $CDCl_3$) δ -63.88. The NMR spectra were in agreement with that reported in the reference.[16]

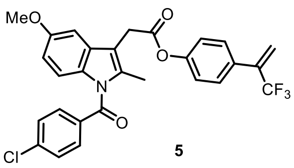

5

The trifluoromethyl alkenes **5** were synthesized according to reported methods.[20] **^{1}H NMR** (400 MHz, $CDCl_3$) δ 7.75 – 7.66 (m, 2H), 7.55 – 7.44 (m, 4H), 7.17 – 7.06 (m, 3H), 6.93 (d, *J* = 9.0 Hz, 1H), 6.73 (dd, *J* = 9.0, 2.5 Hz, 1H), 6.01 – 5.96 (m, 1H), 5.77 (d, *J* = 1.6 Hz, 1H), 3.95 (s, 2H), 3.87 (s, 3H), 2.49 (s, 3H). **^{19}F NMR** (376 MHz, $CDCl_3$) δ -64.88. **^{13}C NMR** (101 MHz, $CDCl_3$) δ 169.1, 168.3, 156.2, 151.2, 139.4, 138.0 (q, *J* = 30.3 Hz), 136.3, 133.8, 131.4, 131.2, 130.9, 130.5, 129.2, 128.6, 123.2 (q, *J* = 273.8 Hz), 121.6, 120.8 (q, *J* = 5.7 Hz), 115.1, 111.8, 101.2, 55.7, 30.6, 13.5. The NMR spectra were in agreement with that reported in the reference.[20]

The HDF of trifluoromethyl alkenes 2 to furnish gem-difluoroalkenes 3

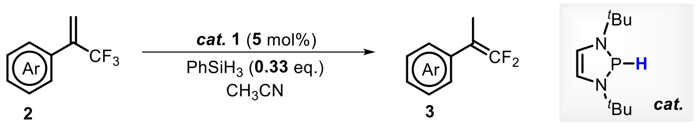

Trifluoromethyl alkenes **2** (0.3 mmol), diazaphospholene **1** (0.015 mmol, 5 mol%), $PhSiH_3$ (0.1 mmol) and CH_3CN (1.0 mL) were taken in a Schlenk tube under argon. The mixture was stirred at the specified temperature. The reactions were monitored by ^{19}F NMR spectra to ensure conversion to desired products. The resulting mixture was cooling down to room temperature and concentrated under vacuum (Note: due to the low boiling point of products, the solvent was evaporated by the rotary evaporator at about 450 mbar). The crude products were purified by flash column chromatography through a silica plug using petroleum ether as the eluent.

The *E* isomer of the **2s** did not react at all. The yield from the *Z* isomer was determined by ^{19}F NMR. Spectral data matches the reported data in the literature.[21]

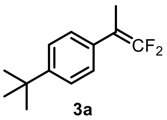

3a

Colorless liquid, 62.3 mg, 0.3 mmol, 99% yield. **^{1}H NMR** (400 MHz, $CDCl_3$) δ 7.47 – 7.29 (m, 4H), 2.01 (t, *J* = 3.4 Hz, 3H), 1.37 (s, 9H). **^{19}F NMR** (376 MHz, $CDCl_3$) δ -90.76 (d, *J* = 44.8 Hz), -91.05 (d, *J* = 44.9 Hz). **^{13}C NMR** (101 MHz, $CDCl_3$) δ 153.5 (dd, *J* = 289.8, 285.5 Hz), 150.0, 131.9, 127.1 (t, *J* = 4.0 Hz), 125.3, 87.2 (dd, *J* = 22.0, 14.4 Hz), 34.5, 31.3, 13.1. The NMR spectra were in agreement with that reported in the reference.[22]

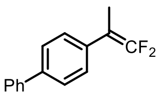

3b

White solid, 68.2 mg, 0.3 mmol, 99% yield. **^{1}H NMR** (400 MHz, $CDCl_3$) δ 7.70 – 7.61 (m, 4H), 7.55 – 7.47 (m, 4H), 7.45 – 7.38 (m, 1H), 2.08 (t, *J* = 3.4 Hz, 3H). **^{19}F NMR** (376 MHz, $CDCl_3$) δ -89.72 (d, *J* = 42.9 Hz), -90.11 (d, *J* = 42.9 Hz). **^{13}C NMR** (101 MHz, $CDCl_3$) δ 153.63 (dd, *J* = 290.8, 286.3 Hz), 140.6, 139.9, 133.9(t, *J* = 4.3 Hz), 128.9, 127.9 (t, *J* = 4.1 Hz), 127.4, 127.1, 127.0, 87.3 (dd, *J* = 22.7, 14.1 Hz), 13.2. **M.P.** 64 - 65 °C. The NMR spectra were in agreement with that reported in the reference.[23]

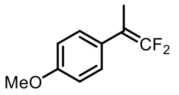
3d

Colorless liquid, 54.7 mg, 0.3 mmol, 99% yield. **^{1}H NMR** (400 MHz, $CDCl_3$) δ 7.36 – 7.27 (m, 2H), 6.97 – 6.86 (m, 2H), 3.84 (s, 3H), 1.97 (t, *J* = 3.4 Hz, 3H). **^{19}F NMR** (376 MHz, $CDCl_3$) δ -91.74 (d, *J* = 47.0 Hz), -91.97 (d, *J* = 47.0 Hz). **^{13}C NMR** (101 MHz, $CDCl_3$) δ 158.5, 153.3 (dd, *J* = 288.6, 285.1 Hz), 128.6 (dd, *J* = 4.3, 3.6 Hz), 127.1 (t, *J* = 3.5 Hz), 113.8, 86.9 (dd, *J* = 22.1, 15.1 Hz), 55.3, 13.4 (t, *J* = 1.8 Hz). The NMR spectra were in agreement with that reported in the reference.[24]

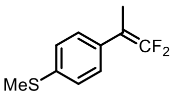
3e

Colorless liquid, 59.1 mg, 0.29 mmol, 98% yield. **^{1}H NMR** (400 MHz, $CDCl_3$) δ 7.35 – 7.30 (m, 2H), 7.29 – 7.25 (m, 2H), 2.52 (s, 3H), 1.98 (t, *J* = 3.4 Hz, 3H). **^{19}F NMR** (376 MHz, $CDCl_3$) δ -90.17 (d, *J* = 43.6 Hz), -90.44 (d, *J* = 43.6 Hz). **^{13}C NMR** (101 MHz, $CDCl_3$) δ 153.5 (dd, *J* = 290.1, 286.1 Hz), 137.3, 131.6 (t, *J* = 4.0 Hz), 127.8 (t, *J* = 4.2 Hz), 126.5, 87.1 (dd, *J* = 22.5, 14.4 Hz), 15.8, 13.1. The NMR spectra were in agreement with that reported in the reference.[6]

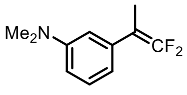
3f

Colorless liquid, 57.9 mg, 0.3 mmol, 99% yield. **^{1}H NMR** (400 MHz, $CDCl_3$) δ 7.31 – 7.21 (m, 1H), 6.80 – 6.66 (m, 3H), 2.99 (s, 6H), 2.00 (t, *J* = 3.4 Hz, 3H). **^{19}F NMR** (376 MHz, $CDCl_3$) δ -90.96 (d, *J* = 45.0 Hz), -91.25 (d, *J* = 45.3 Hz). **^{13}C NMR** (101 MHz, $CDCl_3$) δ 153.4 (dd, *J* = 285.1, 286.1 Hz), 150.6, 135.7 (t, *J* = 3.8 Hz), 129.0, 116.1 (t, *J* = 3.6 Hz), 111.9 (t, *J* = 3.4 Hz), 111.6, 88.1 (dd, *J* = 21.9, 14.8 Hz), 40.6, 13.6. **ESI-HR** calcd for $C_{11}H_{14}F_2N^+$: 198.1089, found 198.1092.

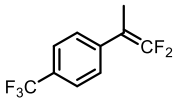
3g

Colorless liquid, 60.6 mg, 0.27 mmol, 91% yield. **^{1}H NMR** (400 MHz, $CDCl_3$) δ 7.65 – 7.58 (m, 2H), 7.53 – 7.44 (m, 2H), 2.00 (t, *J* = 3.4 Hz, 3H). **^{19}F NMR** (376 MHz, $CDCl_3$) δ -62.7, -88.0 (d, *J* = 38.8 Hz), -88.8 (d, *J* = 38.8 Hz). The NMR spectra were in agreement with that reported in the reference.[25]

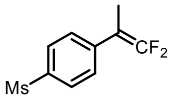
3h

Colorless liquid, 68.6 mg, 0.3 mmol, 99% yield. **^{1}H NMR** (400 MHz, $CDCl_3$) δ 7.98 – 7.87 (m, 2H), 7.63 – 7.50 (m, 2H), 3.06 (s, 3H), 2.01 (t, *J* = 3.4 Hz, 3H). **^{19}F NMR** (376 MHz, $CDCl_3$) δ -86.45 (d, *J* = 35.5 Hz), -87.49 (d, *J* = 35.4 Hz). **^{13}C NMR** (101 MHz, $CDCl_3$) δ 154.0 (dd, *J* = 293.3, 288.7 Hz), 140.7 (t, *J* = 4.8 Hz), 138.9, 128.3 (dd, *J* = 5.3, 3.4 Hz), 127.5, 87.0 (dd, *J* = 24.2, 13.2 Hz), 44.5, 12.9. **ESI-HR** calcd for $C_{10}H_{11}F_2O_2S^+$: 233.0442, found 233.0450.

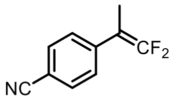
3i

Colorless liquid, 50.5 mg, 0.28 mmol, 94% yield. **^{1}H NMR** (400 MHz, $CDCl_3$) δ 7.66 (d, *J* = 8.4 Hz, 2H), 7.55 – 7.46 (m, 2H), 2.02 (d, *J* = 3.4 Hz, 3H). **^{19}F NMR** (376 MHz, $CDCl_3$) δ -86.19 (d, *J* = 34.8 Hz), -87.07 (d, *J* = 34.8 Hz). **^{13}C NMR** (101 MHz, $CDCl_3$) δ 154.0 (dd, *J* = 293.8, 288.8 Hz), 139.7 (t, *J* = 4.8 Hz), 132.1, 128.0 (dd, *J* = 5.5, 3.4 Hz), 118.7, 110.7, 87.0 (dd, *J* = 24.1, 13.0 Hz), 12.7. The NMR spectra were in agreement with that reported in the reference.[26]

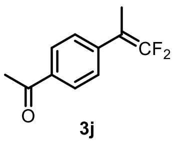
3j

Colorless liquid, 55.9 mg, 0.285 mmol, 95% yield. **^{1}H NMR** (400 MHz, $CDCl_3$) δ 7.94 (d, *J* = 8.4 Hz, 2H), 7.51 – 7.42 (m, 2H), 2.60 (s, 3H), 2.00 (t, *J* = 3.4 Hz, 3H). **^{19}F NMR** (376 MHz, $CDCl_3$) δ -87.39 (d, *J* = 37.4 Hz), -88.02 (d, *J* = 37.4 Hz). **^{13}C NMR** (101 MHz, $CDCl_3$) δ 197.4, 153.9 (dd, *J* = 292.9, 287.9 Hz), 139.8 (t, *J* = 4.7 Hz), 135.6, 128.4, 127.5 (dd, *J* = 5.2, 3.4 Hz), 87.3 (dd, *J* = 23.4, 13.3 Hz), 26.5, 12.9 (t, *J* = 1.8 Hz). The NMR spectra were in agreement with that reported in the reference.[6]

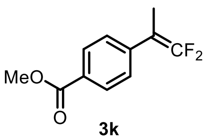

3k

White solid, 62.8 mg, 0.3 mmol, 99% yield. **^{1}H NMR** (400 MHz, $CDCl_3$) δ 8.03 (d, *J* = 8.4 Hz, 2H), 7.50 – 7.41 (m, 2H), 3.93 (d, *J* = 0.7 Hz, 3H), 2.01 (t, *J* = 3.4 Hz, 3H). **^{19}F NMR** (376 MHz, $CDCl_3$) δ -87.68 (d, *J* = 37.6 Hz), -88.22 (d, *J* = 37.6 Hz). **^{13}C NMR** (101 MHz, $CDCl_3$) δ 166.7, 153.8 (dd, *J* = 292.7, 287.7 Hz), 139.6 (t, *J* = 4.6 Hz), 129.8, 129.6, 127.3 (dd, *J* = 5.3, 3.4 Hz), 87.3 (dd, *J* = 23.3, 13.4 Hz), 52.1, 12.9 (d, *J* = 1.8 Hz). **M.P.** 38 - 39 °C. The NMR spectra were in agreement with that reported in the reference.[23]

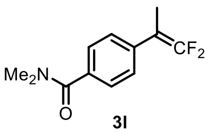

3l

White solid, 62.7 mg, 0.28 mmol, 93% yield. **^{1}H NMR** (400 MHz, $CDCl_3$) δ 7.48 – 7.35 (m, 4H), 3.06 (s, 6H), 1.98 (t, *J* = 3.4 Hz, 3H). **^{19}F NMR** (376 MHz, $CDCl_3$) δ -89.06 (d, *J* = 40.9 Hz), -89.63 (d, *J* = 40.8 Hz). ^{13}C NMR (101 MHz, $CDCl_3$) δ 171.3, 153.7 (dd, *J* = 291.4, 286.9 Hz), 136.3 (t, *J* = 4.5 Hz), 134.5, 127.4, 127.3, 87.2 (dd, *J* = 23.1, 13.9 Hz), 37.6, 13.0. **M.P.** 57 - 58 °C. **ESI-HR** calcd for $C_{12}H_{14}F_2NO^+$: 226.1038, found 226.1041.

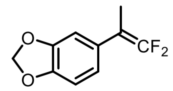

3m

Colorless liquid, 58.0 mg, 0.3 mmol, 99% yield. **^{1}H NMR** (400 MHz, $CDCl_3$) δ 6.92 – 6.77 (m, 3H), 5.99 (s, 2H), 1.95 (t, *J* = 3.4 Hz, 3H). **^{19}F NMR** (376 MHz, $CDCl_3$) δ -91.24 (d, *J* = 3.4 Hz), -91.26 (d, *J* = 3.4 Hz). **^{13}C NMR** (101 MHz, $CDCl_3$) δ 153.3 (t, *J* = 287.2 Hz), 147.6, 146.5, 128.7, 121.0 (t, *J* = 4.0 Hz), 108.2, 108.1 (t, *J* = 4.2 Hz), 101.1, 87.3 (dd, *J* = 19.2, 18.4 Hz), 13.6 (t, *J* = 1.7 Hz). **ESI-HR** calcd for $C_{10}H_9F_2O_2^+$: 199.0565, found 199.0570.

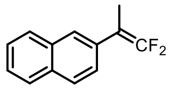

3n

White solid 60.1 mg, 0.29 mmol, 98% yield. **^{1}H NMR** (400 MHz, $CDCl_3$) δ 7.84 – 7.70 (m, 4H), 7.53 – 7.40 (m, 3H), 2.04 (t, *J* = 3.4 Hz, 3H). **^{19}F NMR** (376 MHz, $CDCl_3$) δ -89.64 (d, *J* = 42.6 Hz), -90.28 (d, *J* = 42.9 Hz). **M.P.** 65 - 66 °C. The NMR spectra were in agreement with that reported in the reference.[27]

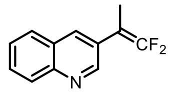

3o

White solid, 61.2 mg, 0.3 mmol, 99% yield. **^{1}H NMR** (400 MHz, $CDCl_3$) δ 8.96 (t, *J* = 2.1 Hz, 1H), 8.22 – 8.00 (m, 2H), 7.90 – 7.73 (m, 1H), 7.70 (ddd, *J* = 8.4, 6.9, 1.5 Hz, 1H), 7.55 (ddd, *J* = 8.1, 6.9, 1.2 Hz, 1H), 2.09 (t, *J* = 3.4 Hz, 3H). **^{19}F NMR** (376 MHz, $CDCl_3$) δ -87.92 (d, *J* = 39.5 Hz), -88.79 (d, *J* = 39.0 Hz). **^{13}C NMR** (101 MHz, $CDCl_3$) δ 154.0 (dd, *J* = 291.6, 288.3 Hz), 149.7 (dd, *J* = 6.5, 2.7 Hz), 146.9, 133.8 (t, *J* = 4.2 Hz), 129.5, 129.2, 128.1 (t, *J* = 4.6 Hz), 127.7, 127.6, 127.0, 85.3 (dd, *J* = 24.4, 14.6 Hz), 13.0. **M.P.** 65 - 66 °C. The NMR spectra were in agreement with that reported in the reference.[6]

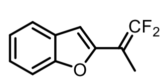

3p

Colorless liquid, 57.8 mg, 0.29 mmol, 98% yield. **^{1}H NMR** (400 MHz, $CDCl_3$) δ 7.61 – 7.54 (m, 1H), 7.51 – 7.48 (m, 1H), 7.34 – 7.20 (m, 2H), 6.72 (p, *J* = 0.7 Hz, 1H), 2.07 (t, *J* = 3.3 Hz, 3H). **^{19}F NMR** (376 MHz, $CDCl_3$) δ -81.04 (d, *J* = 27.3 Hz), -87.84 (d, *J* = 27.2 Hz). **^{13}C NMR** (101 MHz, $CDCl_3$) δ 154.4 (t, *J* = 1.6 Hz), 154.3 (dd, *J* = 297.7, 287.9 Hz), 150.7 (dd, *J* = 7.3, 5.6 Hz), 128.7, 124.1, 122.9, 120.6, 111.0, 103.9 (dd, *J* = 8.5, 6.3 Hz), 81.8 (dd, *J* = 29.0, 13.2 Hz), 10.0 (t, *J* = 1.5 Hz). **M.P.** 42 - 43 °C. **ESI-HR** calcd for $C_{11}H_8F_2NaO^+$: 217.0435, found 217.0439.

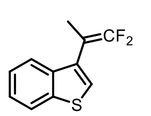

3q

Colorless liquid, 62.5 mg, 0.3 mmol, 99% yield. **^{1}H NMR** (400 MHz, $CDCl_3$) δ 7.95 – 7.88 (m, 1H), 7.78 – 7.72 (m, 1H), 7.49 – 7.39 (m, 2H), 7.35 (s, 1H), 2.07 (t, *J* = 3.4 Hz, 3H). **^{19}F NMR** (376 MHz, $CDCl_3$) δ -87.50 (d, *J* = 41.5 Hz), -91.55 (d, *J* = 41.0 Hz). **^{13}C NMR** (101 MHz, $CDCl_3$) δ 153.40 (t, *J* = 287.7 Hz), 140.0, 137.6, 130.5 (dd, *J* = 4.8, 2.0 Hz), 124.7 (dd, *J* = 4.2, 1.8 Hz), 124.5, 124.3, 122.9, 122.7 (d, *J* = 2.6 Hz), 82.5 (dd, *J* = 25.5, 18.5 Hz), 14.5. **ESI-HR** calcd for $C_{11}H_8F_2NaS^+$:

233.0207, found 233.0210.

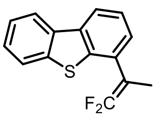

3r

White solid, 77.2 mg, 0.3 mmol, 99% yield. **^{1}H NMR** (400 MHz, $CDCl_3$) δ 8.22 – 8.17 (m, 1H), 8.15 (dd, *J* = 7.9, 1.1 Hz, 1H), 7.95 – 7.85 (m, 1H), 7.54 – 7.47 (m, 3H), 7.39 (dd, *J* = 7.5, 1.1 Hz, 1H), 2.13 (t, *J* = 3.4 Hz, 3H). **^{19}F NMR** (376 MHz, $CDCl_3$) δ -87.61 (d, *J* = 40.3 Hz), -92.04 (d, *J* = 40.3 Hz). **^{13}C NMR** (101 MHz, $CDCl_3$) δ 153.0 (t, *J* = 288.3 Hz), 139.3, 139.2, 136.1, 135.7, 130.1 (d, *J* = 5.0 Hz), 127.1 (t, *J* = 2.5 Hz), 127.0, 124.8, 124.5, 122.8, 121.8, 121.0, 87.0 (dd, *J* = 24.5, 18.4 Hz), 13.6. **M.P.** 72 - 73 °C. **ESI-HR** calcd for $C_{15}H_{10}F_2NaS^+$: 283.0363, found 283.0361.

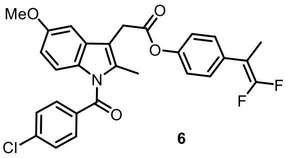

6

White solid,129.8 mg, 0.255 mmol, 85% yield. **^{1}H NMR** (400 MHz, $CDCl_3$) δ 7.75 – 7.67 (m, 2H), 7.55 – 7.47 (m, 2H), 7.37 (dd, *J* = 8.8, 1.1 Hz, 2H), 7.13 – 7.04 (m, 3H), 6.93 (d, *J* = 9.0 Hz, 1H), 6.73 (dd, *J* = 9.0, 2.6 Hz, 1H), 3.93 (s, 2H), 3.87 (s, 3H), 2.48 (s, 3H), 1.97 (t, *J* = 3.4 Hz, 3H). **^{19}F NMR** (376 MHz, $CDCl_3$) δ -89.98 (d, *J* = 42.9 Hz), -90.46 (d, *J* = 42.9 Hz). **^{13}C NMR** (101 MHz, $CDCl_3$) δ 169.3, 168.3, 156.1, 153.5 (dd, *J* = 290.4, 286.2 Hz), 149.5, 139.4, 136.2, 133.8, 132.6 (t, *J* = 4.3 Hz), 131.2, 130.9, 130.5, 129.2, 128.5 (dd, *J* = 4.8, 3.4 Hz), 121.3, 115.0, 111.9, 111.8, 101.2, 86.9 (dd, *J* = 23.3, 14.2 Hz), 55.8, 30.6, 13.5, 13.3 (t, *J* = 1.9 Hz). **ESI-HR** calcd for $C_{28}H_{23}ClF_2NO_4^+$: 510.1278, found 510.1281. **IR** (thin film, cm^{-1}) 649, 723, 903, 1091, 1133, 1239, 1317, 1732. **M.P.** 106 - 107 °C.

The HDF of trifluoromethyl alkenes 2 to furnish monofluoroalkenes 4

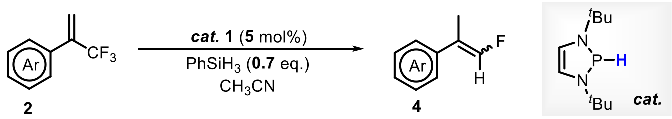

Trifluoromethyl alkenes **2** (0.3 mmol), diazaphospholene **1** (0.015 mmol, 5 mol%), $PhSiH_3$ (0.21 mmol) and CH_3CN (1.0 mL) were taken in a Schlenk tube under argon. The mixture was stirred at the specified temperature. The reactions were monitored by ^{19}F NMR spectra to ensure conversion to desired products. The resulting mixture was cooling down to room temperature and concentrated under vacuum (Note: due to the low boil of products, the solvent was evaporated by the rotary evaporator at about 450 mbar). The crude products were purified by flash column chromatography through a silica plug using petroleum ether as the eluent.

The *E* isomer of the **2s** did not react at all. The yield from the Z isomer was determined by ^{19}F NMR. Spectral data matches the reported data in the literature.[28]

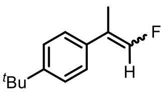

4a
E/*Z* = 83/17

Colorless liquid, 57.0 mg, 0.3 mmol, 99% yield. **^{1}H NMR** (400 MHz, $CDCl_3$, major *E* isomer) δ 7.42 – 7.36 (m, 2H), 7.27 (d, *J* = 8.5 Hz, 2H), 6.93 (dq, *J* = 85.4, 1.5 Hz, 1H), 2.06 (dd, *J* = 3.8, 1.6 Hz, 3H), 1.35 (s, 9H). **^{19}F NMR** (376 MHz, $CDCl_3$) δ -129.39 (*Z*), -132.16 (*E*). **ESI-HR** calcd for $C_{13}H_{18}F^+$: 193.1387, found 193.1388.

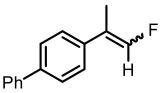

4b
E/*Z* = 84/16

White solid, 63.1 mg, 0.3 mmol, 99% yield. **^{1}H NMR** (400 MHz, $CDCl_3$, major *E* isomer) δ 7.65 – 7.56 (m, 4H), 7.47 (dd, *J* = 8.4, 6.8 Hz, 2H), 7.44 – 7.37 (m, 3H), 7.00 (dq, *J* = 85.0, 1.6 Hz, 1H), 2.11 (dd, *J* = 3.8, 1.5 Hz, 3H). **^{19}F NMR** (376 MHz, $CDCl_3$) δ -128.13 (*Z*), -130.84 (*E*). **^{13}C NMR** (101 MHz, $CDCl_3$, major *E* isomer) δ 146.1 (d, *J* = 258.3 Hz), 140.6, 140.3, 136.5 (d, *J* = 8.9 Hz), 128.8, 127.4, 127.3, 127.0, 126.2 (d, *J* = 3.3 Hz), 119.7 (d, *J* = 10.0 Hz), 12.2 (d, *J* = 5.9 Hz). **M.P.** 51 - 52 °C. The NMR spectra were in agreement with that reported in the reference.[29]

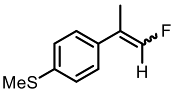

4e
E/*Z* = 88/12

Colorless liquid, 53.9 mg, 0.3 mmol, 99% yield. **^{1}H NMR** (400 MHz, $CDCl_3$, major *E* isomer) δ 7.26 (s, 4H), 6.93 (dd, *J* = 85.0, 1.6 Hz, 1H), 2.52 (s, 3H), 2.06 (dd, *J* = 3.8, 1.6 Hz, 3H). **^{19}F NMR** (376 MHz, $CDCl_3$) δ -128.36 (*Z*), -131.40 (*E*). The NMR spectra were in agreement with that reported in the reference.[30]

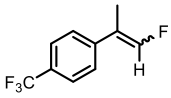

4g
E/*Z* = 82/18

Colorless liquid, 56.3 mg, 0.276 mmol, 92% yield. **^{1}H NMR** (400 MHz, $CDCl_3$) δ 7.61 (d, *J* = 8.5 Hz, 2H), 7.44 (d, *J* = 8.1 Hz, 2H), 6.98 (dd, *J* = 83.9, 1.7 Hz, 1H), 2.09 (dd, *J* = 3.9, 1.5 Hz, 3H). **^{19}F NMR** (376 MHz, $CDCl_3$) δ -63.09 (*E*), -63.16 (*Z*), -127.06 (*Z*), -128.50 (*E*). The NMR spectra were in agreement with that reported in the reference.[29]

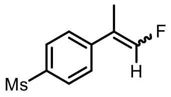

4h
E/*Z* = 89/11

White solid, 63.3 mg, 0.3 mmol, 99% yield. **^{1}H NMR** (400 MHz, $CDCl_3$, major *E* isomer) δ 7.91 (d, *J* = 8.5 Hz, 2H), 7.50 (d, *J* = 8.5 Hz, 2H), 7.00 (dq, *J* = 83.3, 1.4 Hz, 1H), 3.06 (s, 3H), 2.08 (dd, *J* = 3.8, 1.5 Hz, 3H). **^{19}F NMR** (376 MHz, $CDCl_3$) δ -124.95 (*Z*), -125.88 (*E*). **M.P.** 59 - 60 °C. **ESI-HR** calcd for $C_{10}H_{11}FNaO_2S^+$: 237.0356, found 237.0354.

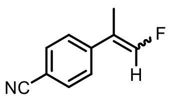

4i
E/*Z* = 88/12

Colorless liquid, 44.4 mg, 0.276 mmol, 92% yield. **^{1}H NMR** (400 MHz, $CDCl_3$, major *E* isomer) δ 7.64 (d, *J* = 8.4 Hz, 2H), 7.42 (d, *J* = 8.4 Hz, 2H), 7.00 (dq, *J* = 83.2, 1.6 Hz, 1H), 2.07 (dd, *J* = 3.9, 1.5 Hz, 3H). **^{19}F NMR** (376 MHz, $CDCl_3$) δ -124.46 (*Z*), -126.12 (*E*). The NMR spectra were in agreement with that reported in the reference.[30]

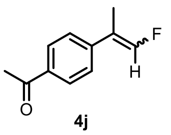

4j
E/*Z* = 90/10

Colorless liquid, 49.7 mg, 0.28 mmol, 93% yield. **^{1}H NMR** (400 MHz, $CDCl_3$, major *E* isomer) δ 7.94 (d, *J* = 8.4 Hz, 2H), 7.41 (d, *J* = 8.5 Hz, 2H), 7.01 (dq, *J* = 83.9, 1.5 Hz, 1H), 2.61 (s, 3H), 2.08 (dd, *J* = 3.8, 1.5 Hz, 3H). **^{19}F NMR** (376 MHz, $CDCl_3$) δ -125.62 (*Z*), -127.62 (*E*). **^{13}C NMR** (101 MHz, $CDCl_3$, major *E* isomer) δ 197.5, 147.2 (d, *J* = 261.4 Hz), 142.4 (d, *J* = 9.2 Hz), 136.0 (d, *J* = 1.4 Hz), 128.7, 125.8 (d, *J* = 3.3 Hz), 119.5 (d, *J* = 10.6 Hz), 26.5, 11.9 (d, *J* = 6.0 Hz). **ESI-HR** calcd for $C_{11}H_{11}FNaO^+$: 201.0686, found 201.0687.

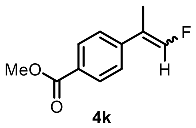

4k
E/*Z* = 88/12

Colorless liquid, 53.5 mg, 0.276 mmol, 92% yield. **^{1}H NMR** (400 MHz, $CDCl_3$) δ 8.01 (d, *J* = 8.2 Hz, 2H), 7.38 (d, *J* = 8.2 Hz, 2H), 7.01 (d, *J* = 83.3 Hz, 1H), 3.93 (s, 3H), 2.07 (dd, *J* = 4.0, 1.4 Hz, 3H). **^{19}F NMR** (376 MHz, $CDCl_3$) δ -125.83 (*Z*), -128.00 (*E*). **^{13}C NMR** (101 MHz, $CDCl_3$, major *E* isomer) δ 166.8, 147.1 (d, *J* = 260.8 Hz), 142.2 (d, *J* = 9.1 Hz), 129.9, 129.1, 125.6 (d, *J* = 3.3 Hz), 119.6 (d, *J* = 10.5 Hz), 52.1, 11.9 (d, *J* = 6.0 Hz). **M.P.** 40 - 41 °C. **ESI-HR** calcd for $C_{11}H_{11}FNaO_2^+$: 217.0635, found 217.0632.

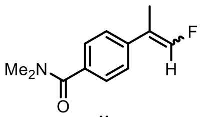

4l
E/*Z* = 85/15

White solid, 61.8 mg, 0.3 mmol, 99% yield. **^{1}H NMR** (400 MHz, $CDCl_3$, major *E* isomer) δ 7.39 (d, *J* = 8.3 Hz, 2H), 7.33 (d, *J* = 8.3 Hz, 2H), 6.93 (dq, *J* = 84.4, 1.5 Hz, 1H), 3.11 (s, 3H), 3.00 (s, 3H), 2.04 (dd, *J* = 3.8, 1.5 Hz, 3H). **^{19}F NMR** (376 MHz, $CDCl_3$) δ -127.51 (*Z*), -129.60 (*E*). **^{13}C NMR** (101 MHz, $CDCl_3$, major *E* isomer) δ 171.2, 146.5 (d, *J* = 259.4 Hz), 138.9 (d, *J* = 9.0 Hz), 135.3, 127.5, 125.7 (d, *J* = 3.3 Hz), 119.6 (d, *J* = 10.3 Hz), 39.6, 35.4, 12.1 (d, *J* = 5.9 Hz). **M.P.** 60 - 61 °C. **ESI-HR** calcd for $C_{12}H_{14}FNNaO^+$: 230.0952, found 230.0946.

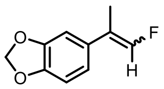

4m
E/*Z* = 81/19

Colorless liquid, 53.4 mg, 0.3 mmol, 99% yield. **^{1}H NMR** (400 MHz, $CDCl_3$, major *E* isomer) δ 6.85 (dd, *J* = 85.2, 1.5 Hz, 4H), 6.83 – 6.77 (m, 3H), 5.98 (s, 2H), 2.02 (dd, *J* = 3.8, 1.6 Hz, 3H). **^{19}F NMR** (376 MHz, $CDCl_3$) δ -129.33 (*Z*), -132.17 (*E*). The NMR spectra were in agreement with that reported in the reference.[30]

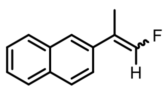

4n
E/*Z* =86/14

White solid, 55.2 mg, 0.3 mmol, 99% yield. **^{1}H NMR** (400 MHz, $CDCl_3$, major *E* isomer) δ 7.91 – 7.75 (m, 4H), 7.58 – 7.45 (m, 3H), 7.09 (dq, *J* = 84.9, 1.5 Hz, 1H), 2.19 (dd, *J* = 3.8, 1.5 Hz, 3H). **^{19}F NMR** (376 MHz, $CDCl_3$) δ -128.37 (*Z*), -130.39 (*E*). **M.P.** 59 - 60 °C. The NMR spectra were in agreement with that reported in the reference.[30]

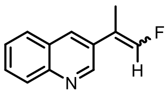

4o
E/*Z* = 79/21

White solid, 55.6 mg, 0.3 mmol, 99% yield. **^{1}H NMR** (400 MHz, $CDCl_3$, major *E* isomer) δ 8.90 (d, *J* = 2.3 Hz, 1H), 8.08 (d, *J* = 8.5 Hz, 1H), 7.99 (d, *J* = 2.3 Hz, 1H), 7.84 – 7.78 (m, 1H), 7.73 – 7.62 (m, 1H), 7.59 – 7.49 (m, 1H), 7.08 (dd, *J* = 83.5, 1.6 Hz, 1H), 2.15 (dd, *J* = 3.7, 1.5 Hz, 3H). **^{19}F NMR** (376 MHz, $CDCl_3$) δ -126.13 (*Z*), -127.10 (*E*). **M.P.** 55 - 56 °C. **ESI-HR** calcd for $C_{12}H_{11}FN^+$: 188.0870, found 188.0865.

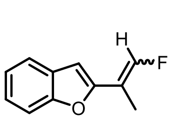

4p
E/*Z* = 86/14

White solid, 40.6 mg, 0.23 mmol, 77% yield. **^{1}H NMR** (400 MHz, $CDCl_3$, *E* isomer) δ 7.51 – 7.46 (m, 1H), 7.43 (d, *J* = 84.0 Hz, 1H), 7.39 (d, *J* = 8.1 Hz, 1H), 7.28 – 7.21 (m, 1H), 7.18 (t, *J* = 7.5 Hz, 1H), 6.58 (s, 1H), 2.01 (dd, *J* = 3.5, 1.4 Hz, 3H). **^{19}F NMR** (376 MHz, $CDCl_3$, *E* isomer) δ-132.54. **^{13}C NMR** (101 MHz, $CDCl_3$, *E* isomer) δ 154.4, 154.1 (d, *J* = 8.8 Hz), 148.2 (d, J = 261.7 Hz), 128.6, 124.4, 122.8, 120.5, 111.3 (d, *J* = 15.0 Hz), 110.8, 103.0 (d, *J* = 10.0 Hz), 9.5 (d, *J* = 5.6 Hz). White solid, 6.3 mg, 0.036 mmol, 12% yield. **^{1}H NMR** (400 MHz, $CDCl_3$, *Z* isomer) δ 7.57 (dd, *J* = 7.8, 2.9 Hz, 1H), 7.48 (dd, *J* = 8.2, 2.9 Hz, 1H), 7.30 – 7.13 (m, 2H), 6.95 (s, 1H), 6.70 (d, *J* = 80.0 Hz, 1H), 2.03 – 1.94 (m, 3H). **^{9}F NMR** (376 MHz, $CDCl_3$, *Z* isomer) δ-120.12. **M.P.** 45 - 46 °C. **ESI-HR** calcd for $C_{11}H_9FNaO^+$: 199.0530, found 199.0532.

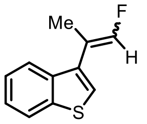

4q
E/*Z* = 70/30

Colorless liquid, 46.0 mg, 0.24 mmol, 80% yield. **^{1}H NMR** (400 MHz, $CDCl_3$, major *E* isomer) δ 7.93 – 7.86 (m, 3H), 7.47 – 7.39 (m, 3H), 7.28 (s, 1H), 6.94 (d, *J* = 85.1 Hz, 1H), 2.17 (dd, *J* = 3.7, 1.5 Hz, 3H). **^{19}F NMR** (376 MHz, $CDCl_3$) δ -124.65 (*Z*), -128.26 (*E*). **ESI-HR** calcd for $C_{11}H_9FNaO^+$: 215.0301, found 215.0308.

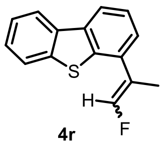

4r
E/*Z* = 79/21

White solid, 46.5 mg, 0.19 mmol, 64% yield for *E* isomer of **4r**. **^{1}H NMR** (400 MHz, $CDCl_3$, *E* isomer) δ 8.22 – 8.15 (m, 1H), 8.12 (dd, *J* = 7.8, 1.1 Hz, 1H), 7.93 – 7.84 (m, 1H), 7.54 – 7.46 (m, 3H), 7.35 (dd, *J* = 7.4, 1.2 Hz, 1H), 7.06 (dq, *J* = 84.5, 1.6 Hz, 1H), 2.22 (dd, *J* = 3.8, 1.6 Hz, 3H). **^{19}F NMR** (376 MHz, $CDCl_3$) δ -125.12 (*Z*), -128.23 (*E*). **^{13}C NMR** (101 MHz, $CDCl_3$, *E* isomer) δ 147.72 (d, *J* = 262.0 Hz), 139.3, 138.7 (d, *J* = 2.5 Hz), 136.1, 135.7, 133.0 (d, *J* = 9.9 Hz), 126.9, 126.3 (d, *J* = 3.4 Hz), 124.8, 124.5, 122.7, 121.7, 120.7, 119.7 (d, *J* = 10.5 Hz), 13.2 (d, *J* = 5.0 Hz). **M.P.** 67 - 68 °C. **ESI-HR** calcd for $C_{15}H_{11}FNaS^+$: 265.0458, found 265.0460.

NMR spectra

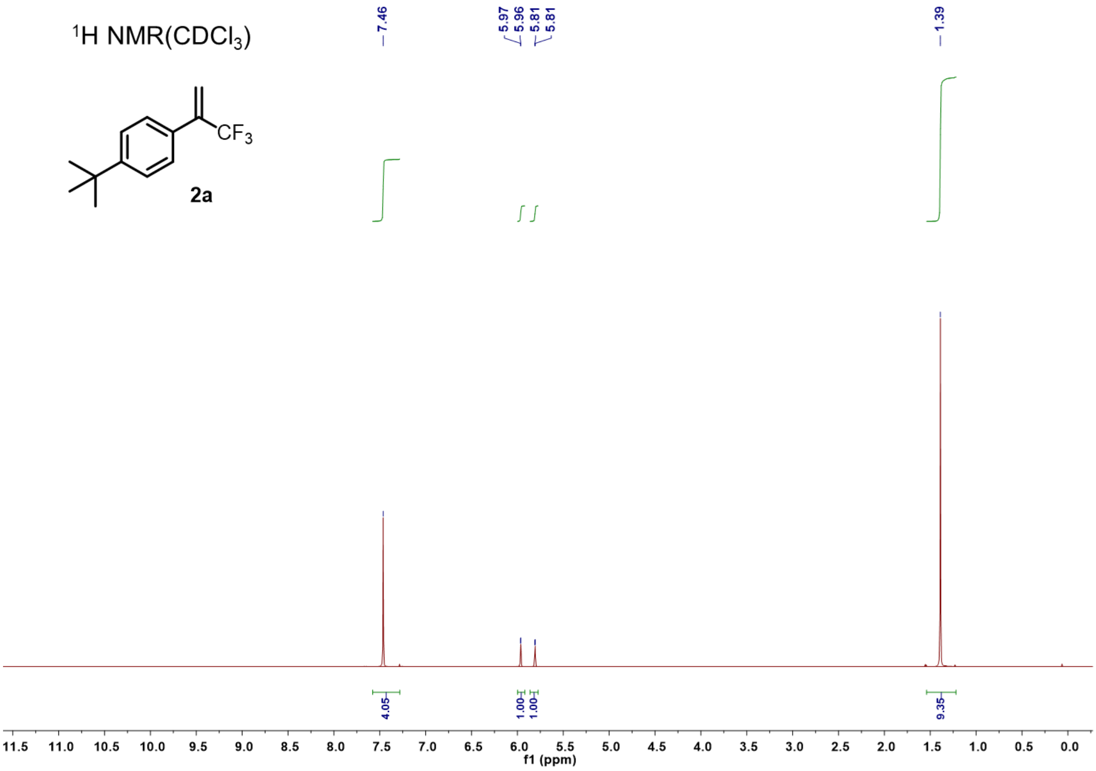

Supplementary Figure 6. ^{1}H NMR Spectrum of **2a**.

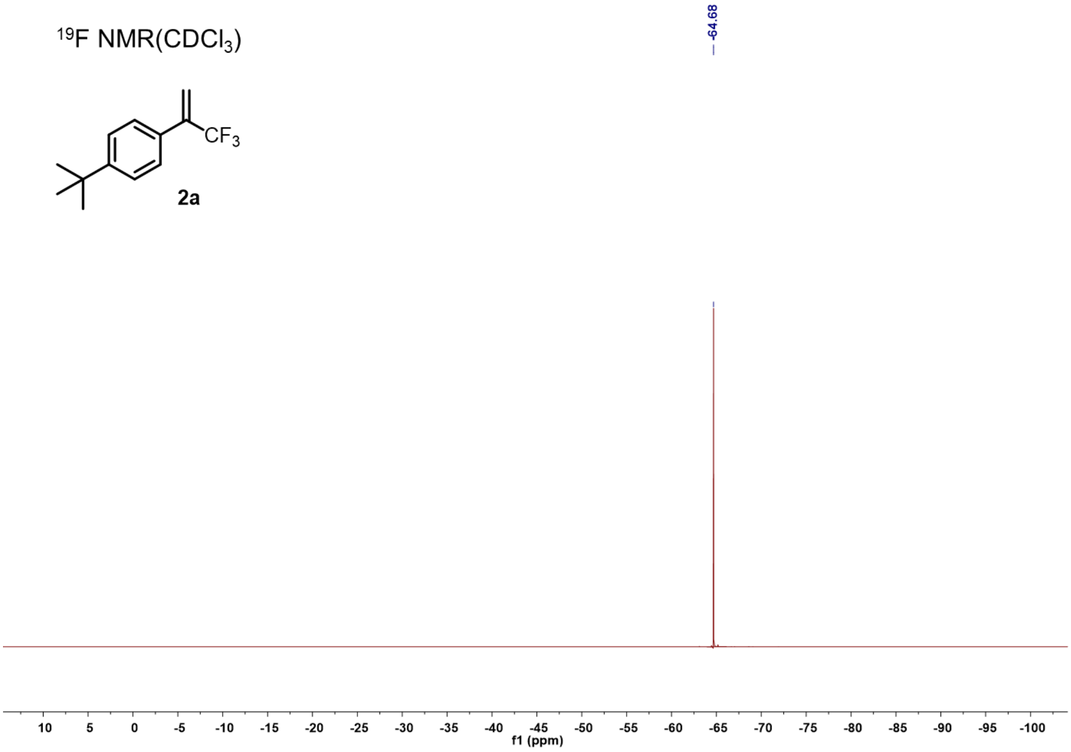

Supplementary Figure 7. ^{19}F NMR Spectrum of **2a**.

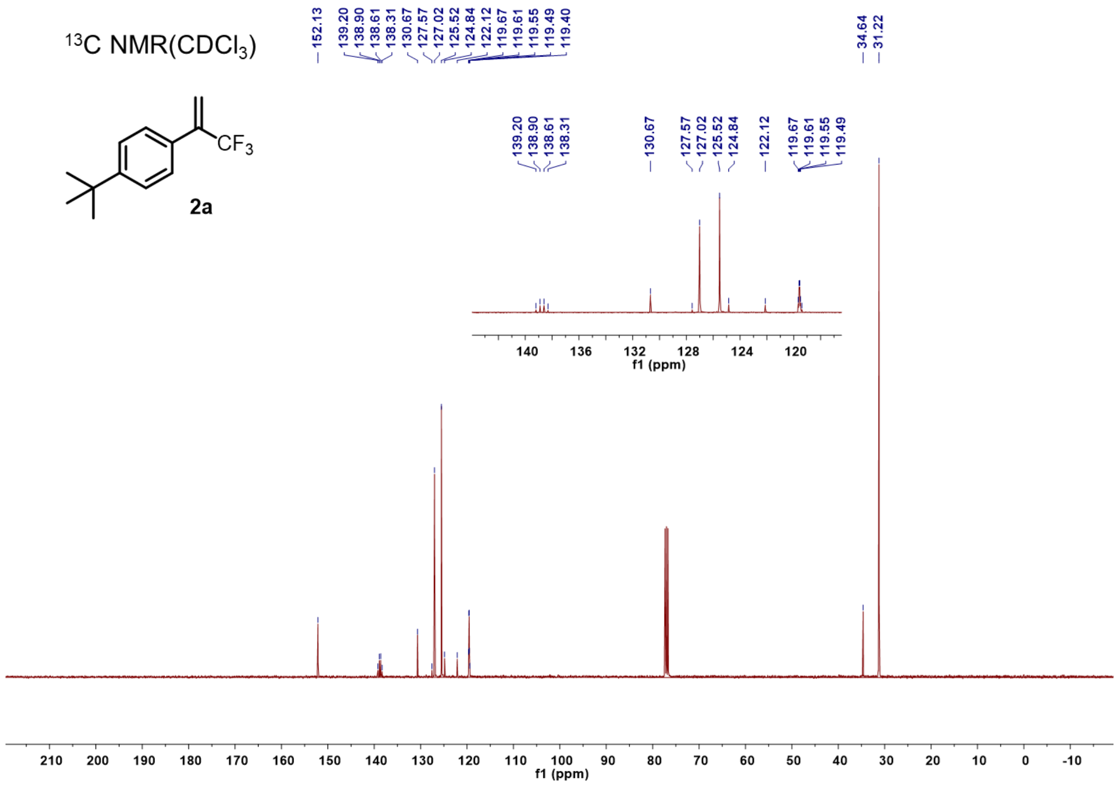

Supplementary Figure 8. ^{13}C NMR Spectrum of **2a**.

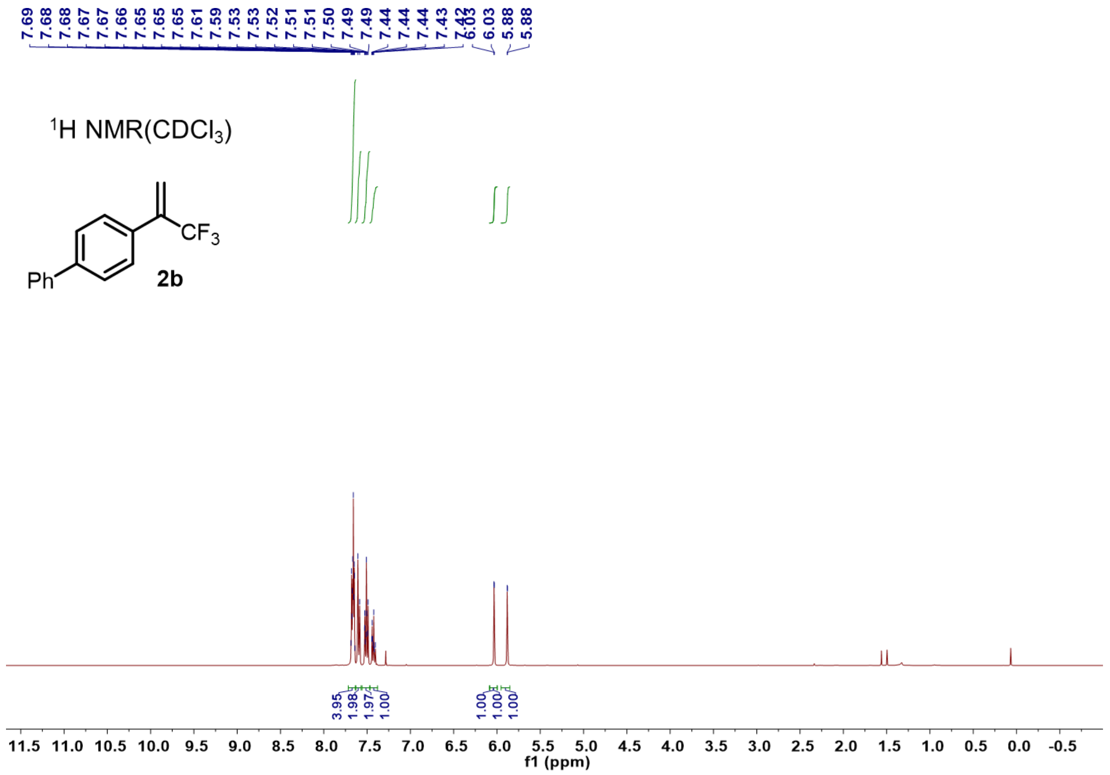

Supplementary Figure 9. ^{1}H NMR Spectrum of **2b**.

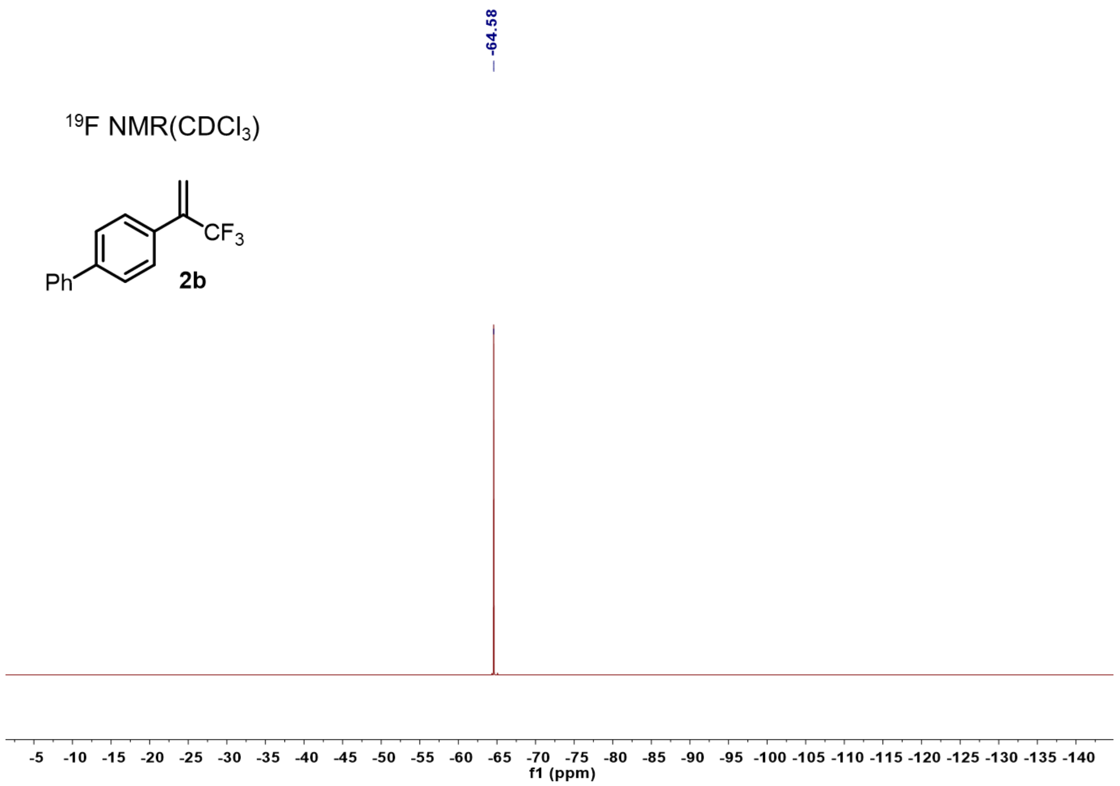

Supplementary Figure 10. ^{19}F NMR Spectrum of **2b**.

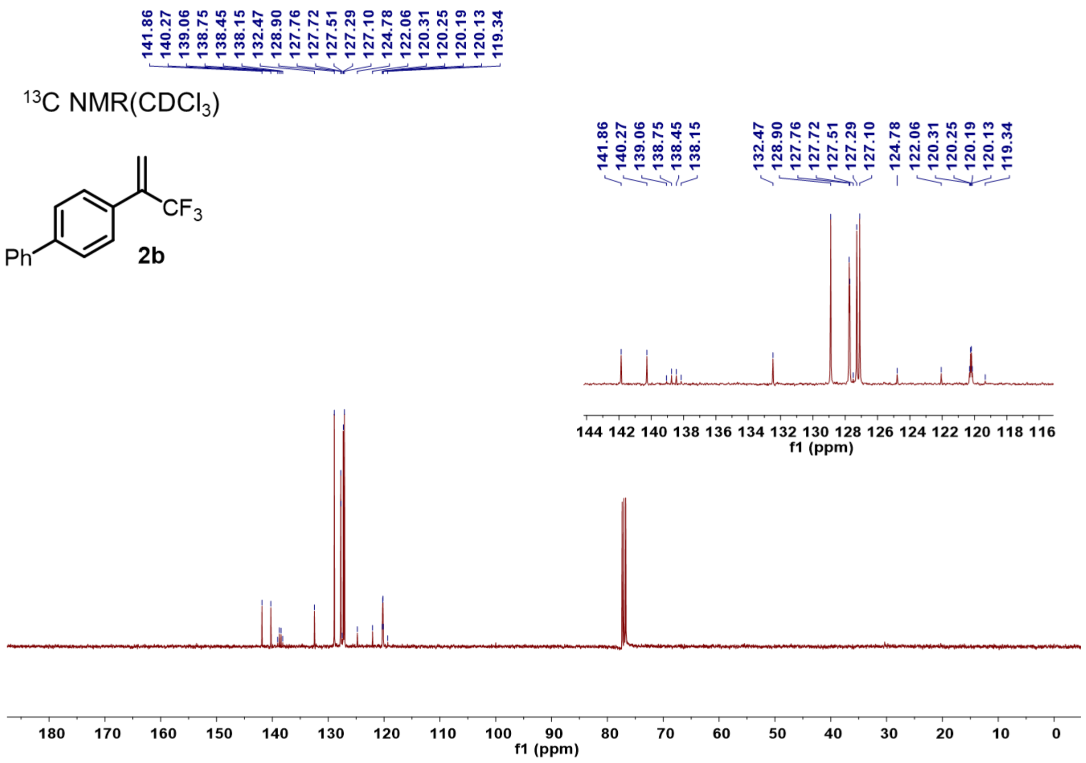

Supplementary Figure 11. ^{13}C NMR Spectrum of **2b**.

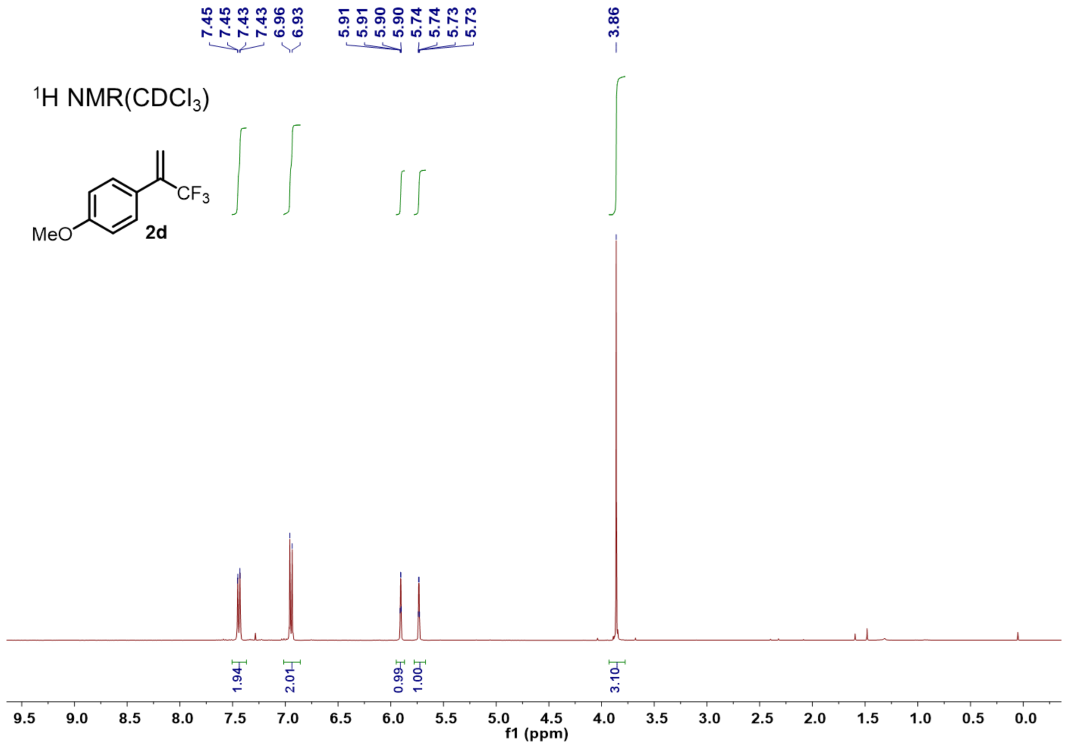

Supplementary Figure 12. ^{1}H NMR Spectrum of **2d**.

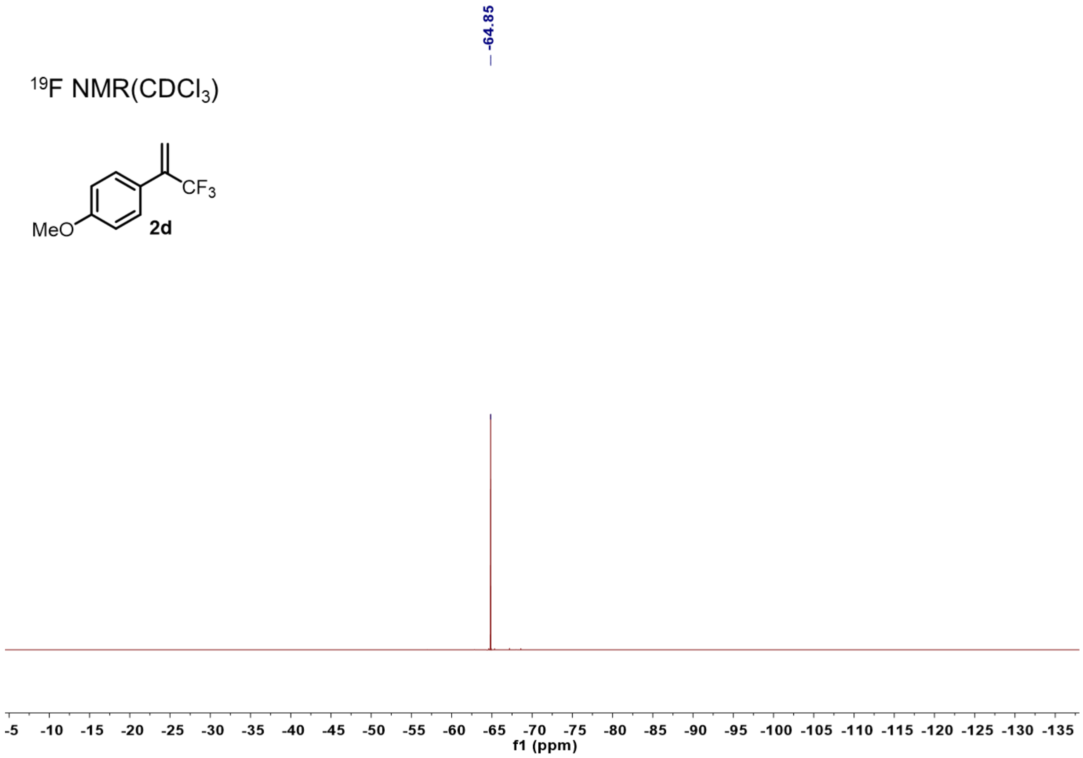

Supplementary Figure 13. ^{19}F NMR Spectrum of **2d**.

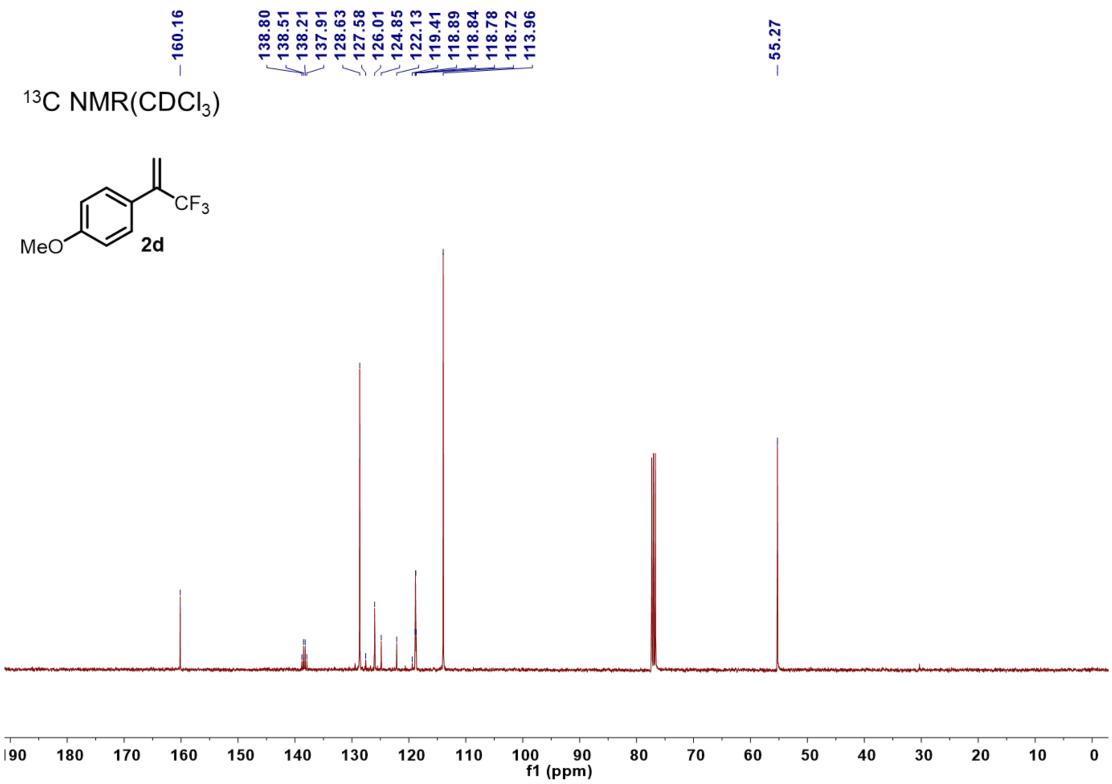

Supplementary Figure 14. ^{13}C NMR Spectrum of **2d**.

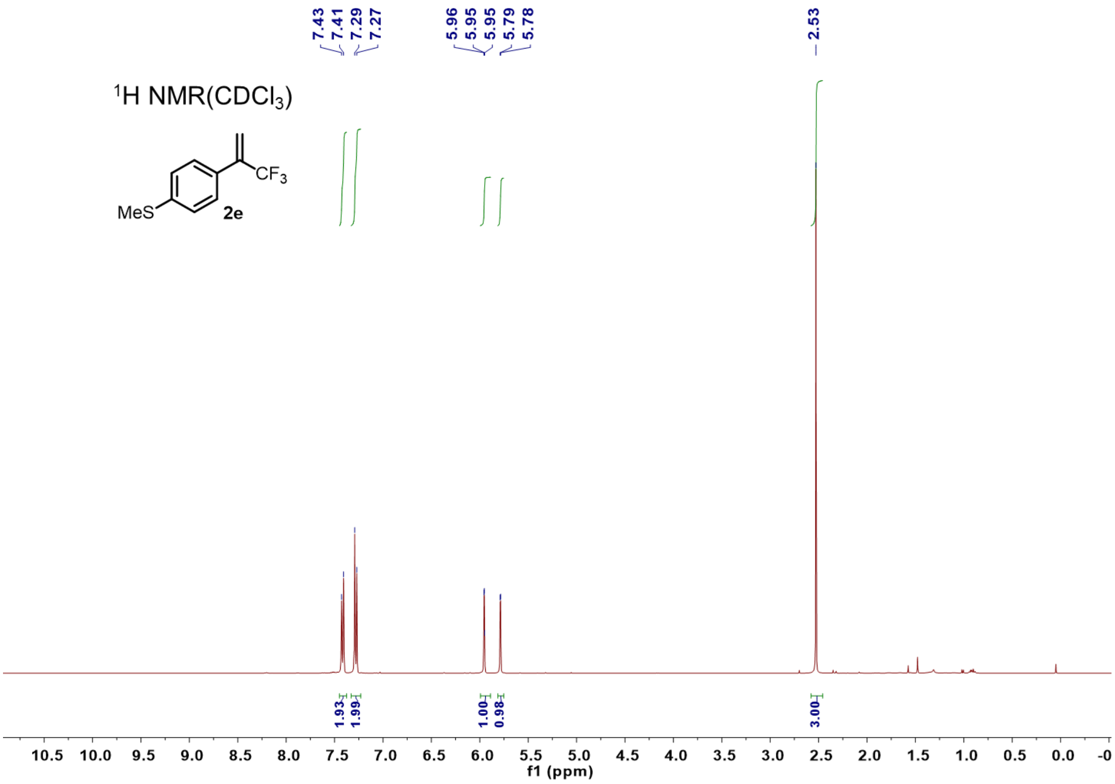

Supplementary Figure 15. ^{1}H NMR Spectrum of **2e**.

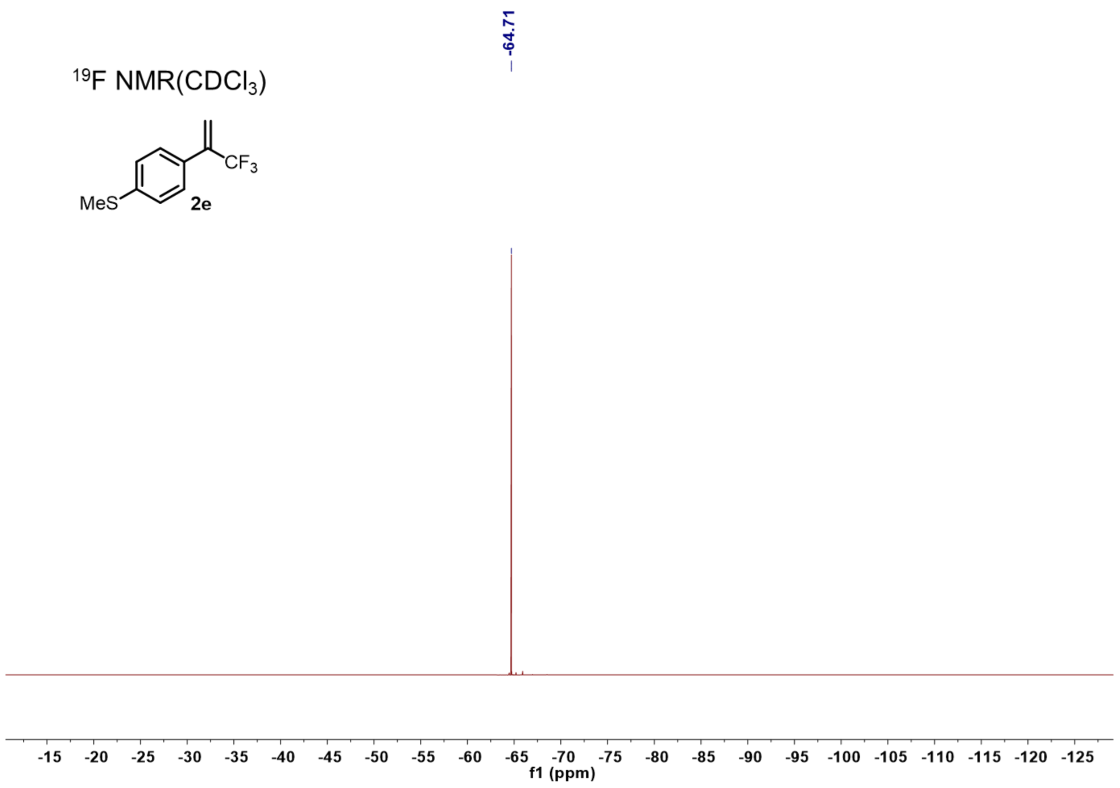

Supplementary Figure 16. ^{19}F NMR Spectrum of **2e**.

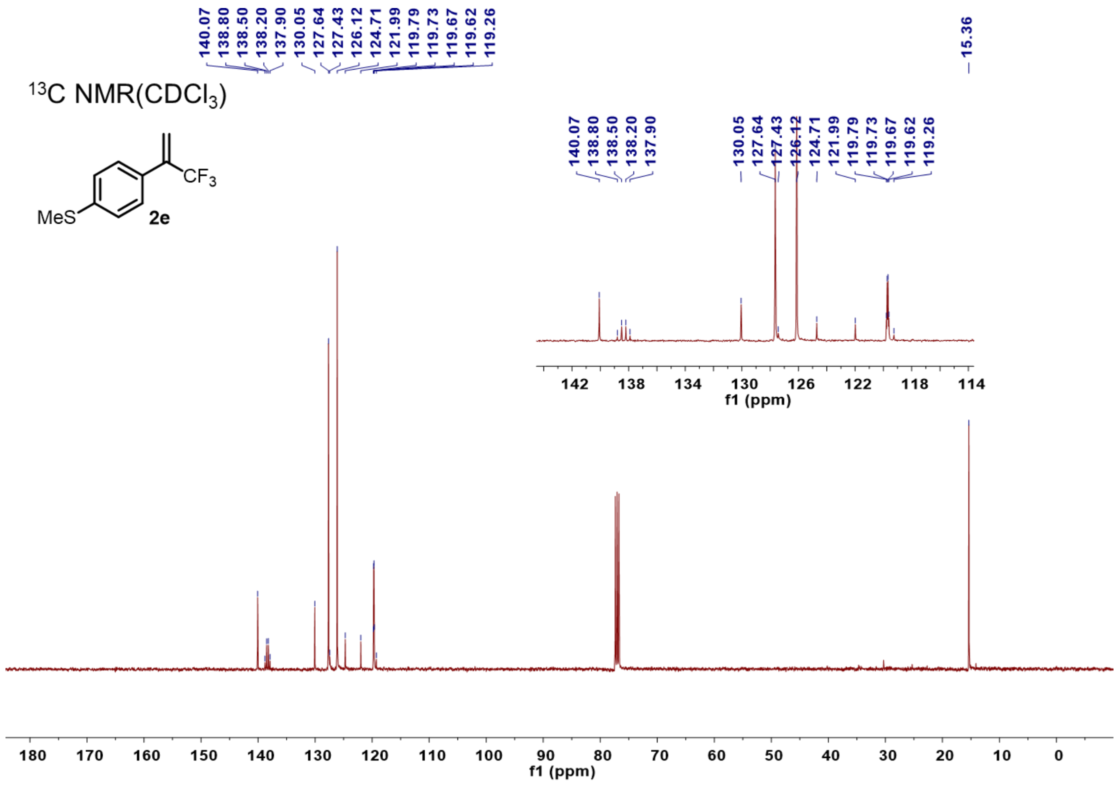

Supplementary Figure 17. ^{13}C NMR Spectrum of **2e**.

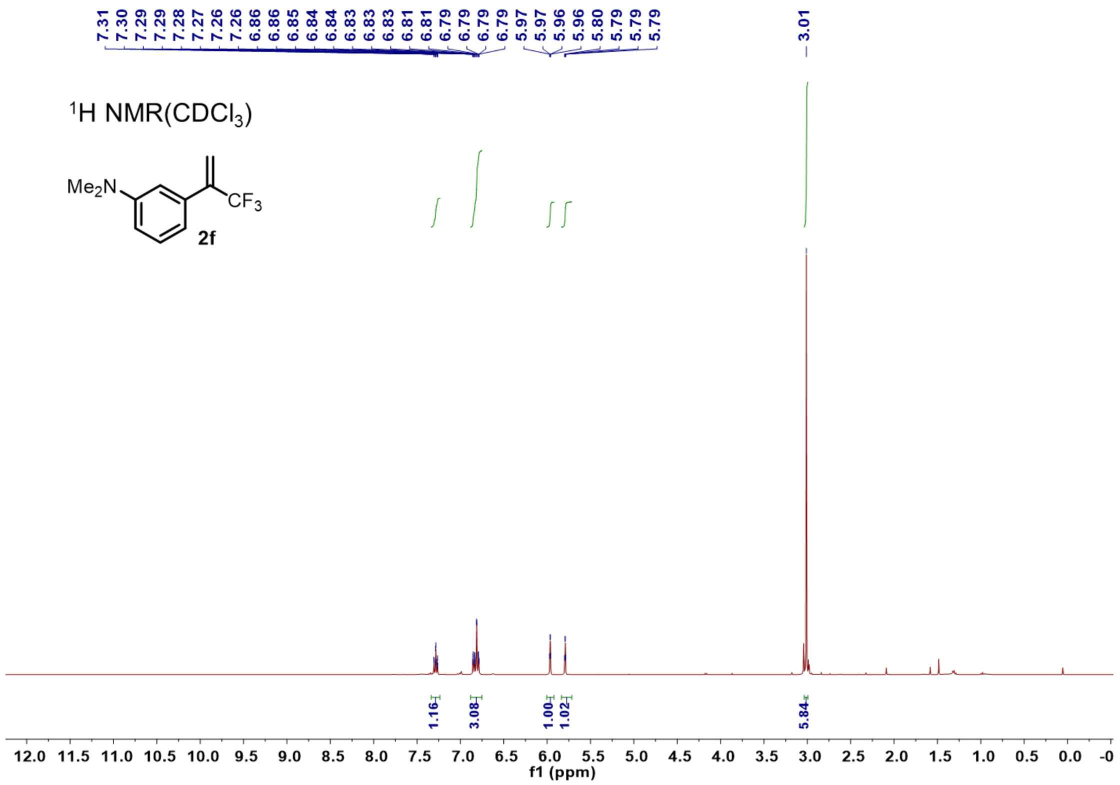

Supplementary Figure 18. ^{1}H NMR Spectrum of **2f**.

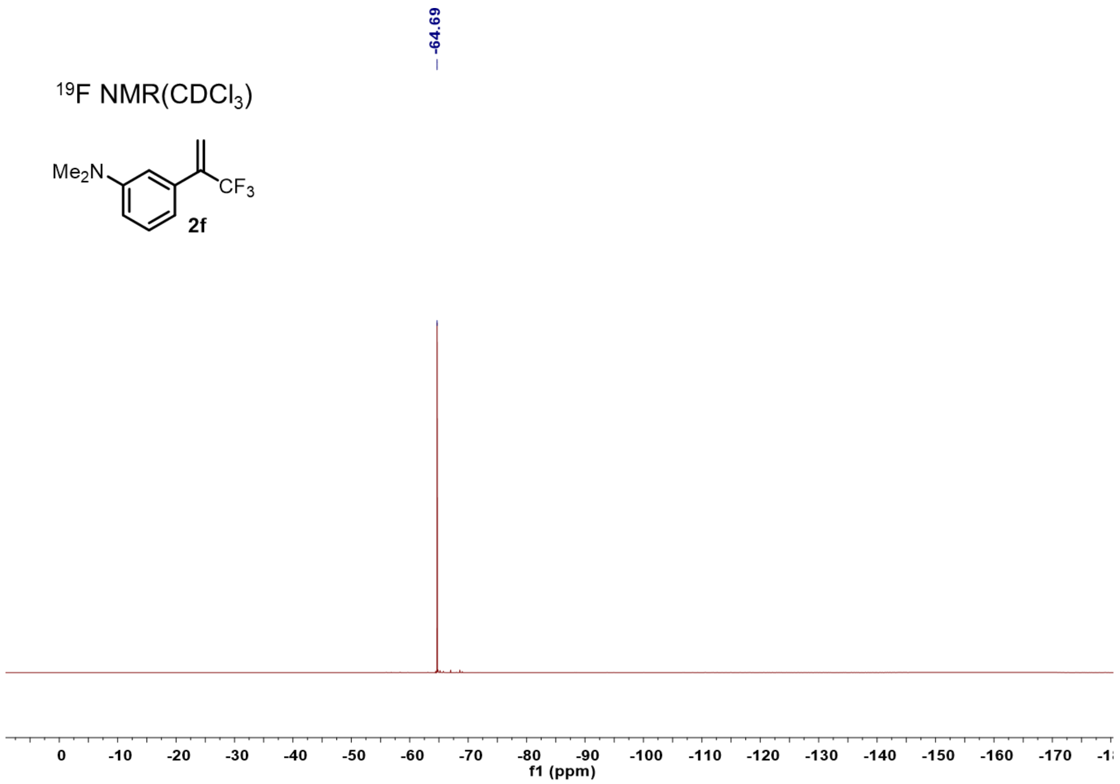

Supplementary Figure 19. ^{19}F NMR Spectrum of **2f**.

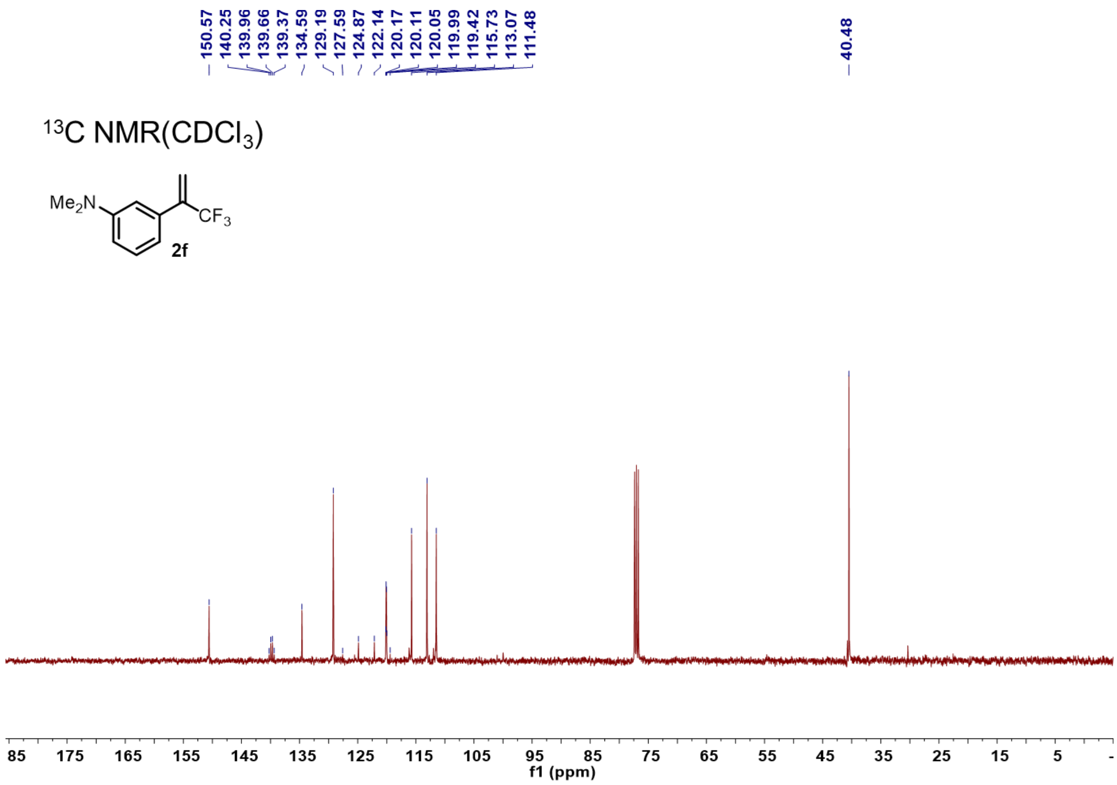

Supplementary Figure 20. ^{13}C NMR Spectrum of **2f**.

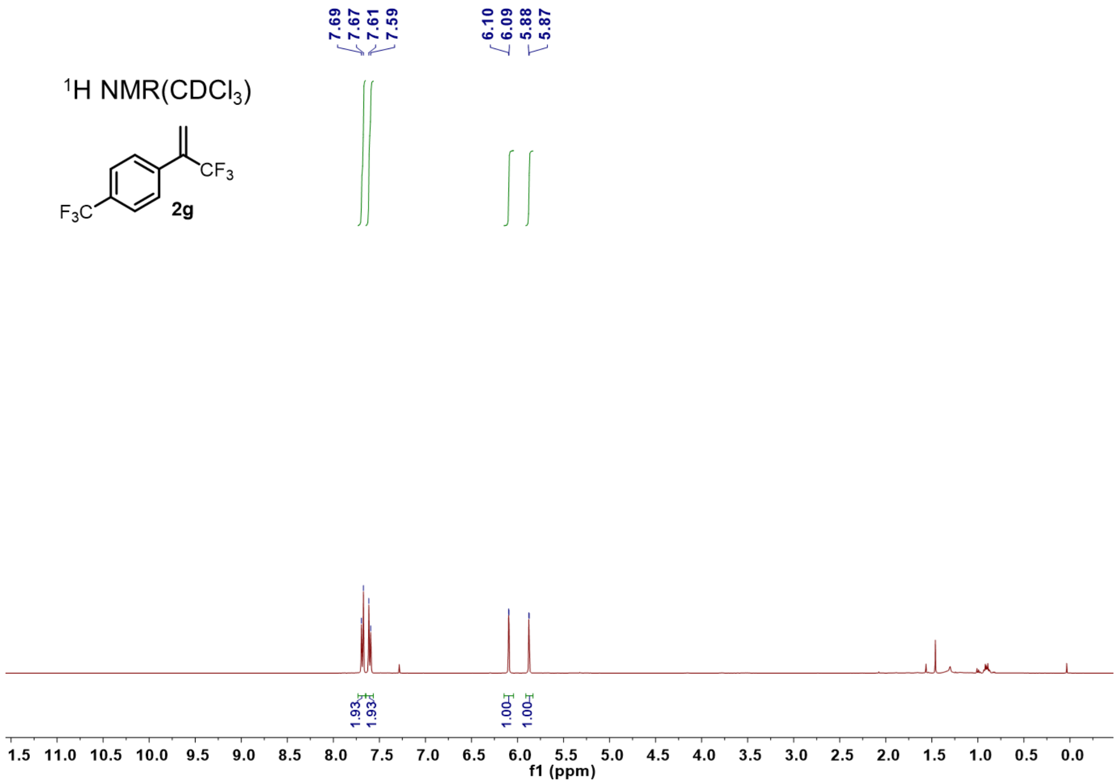

Supplementary Figure 21. ^{1}H NMR Spectrum of **2g**.

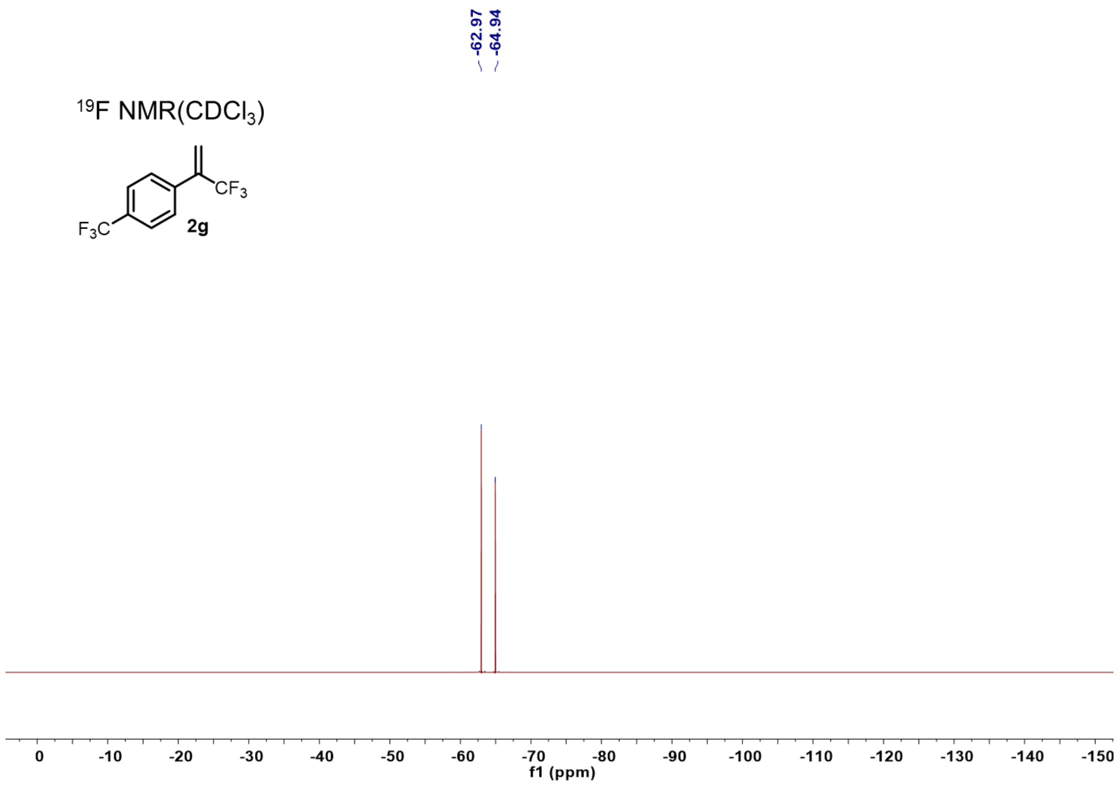

Supplementary Figure 22. ^{19}F NMR Spectrum of **2g**.

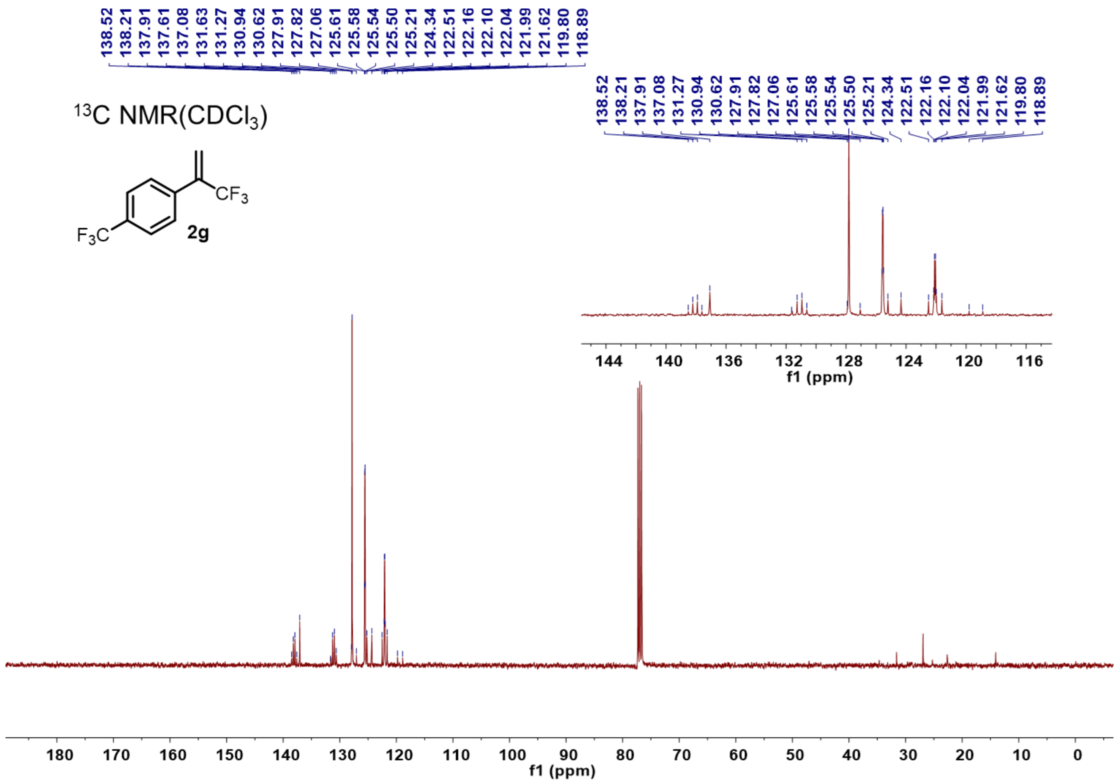

Supplementary Figure 23. ^{13}C NMR Spectrum of **2g**.

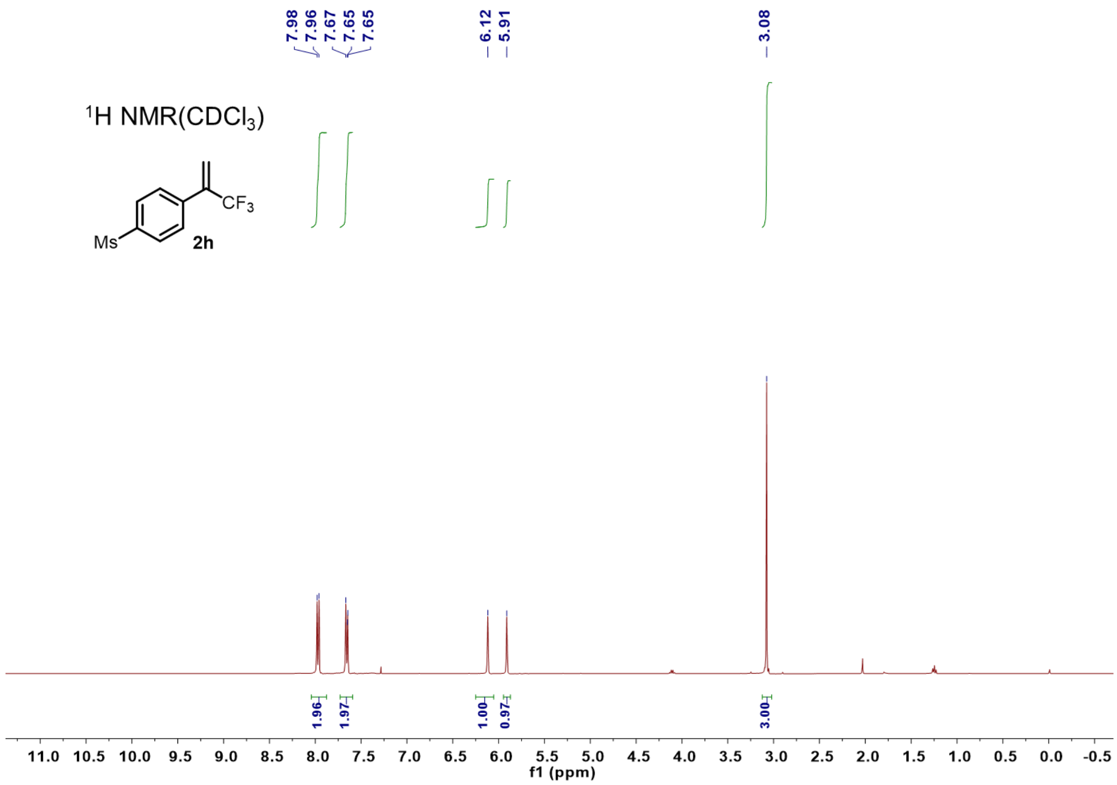

Supplementary Figure 24. ^{1}H NMR Spectrum of **2h**.

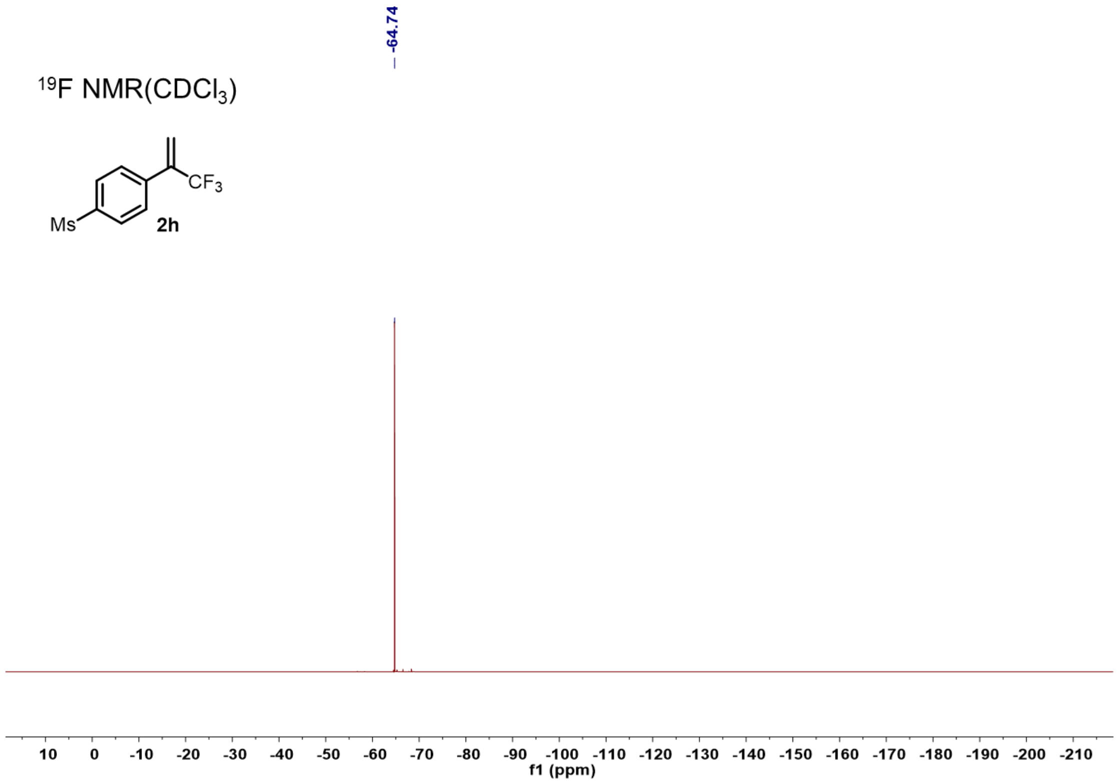

Supplementary Figure 25. ^{19}F NMR Spectrum of **2h**.

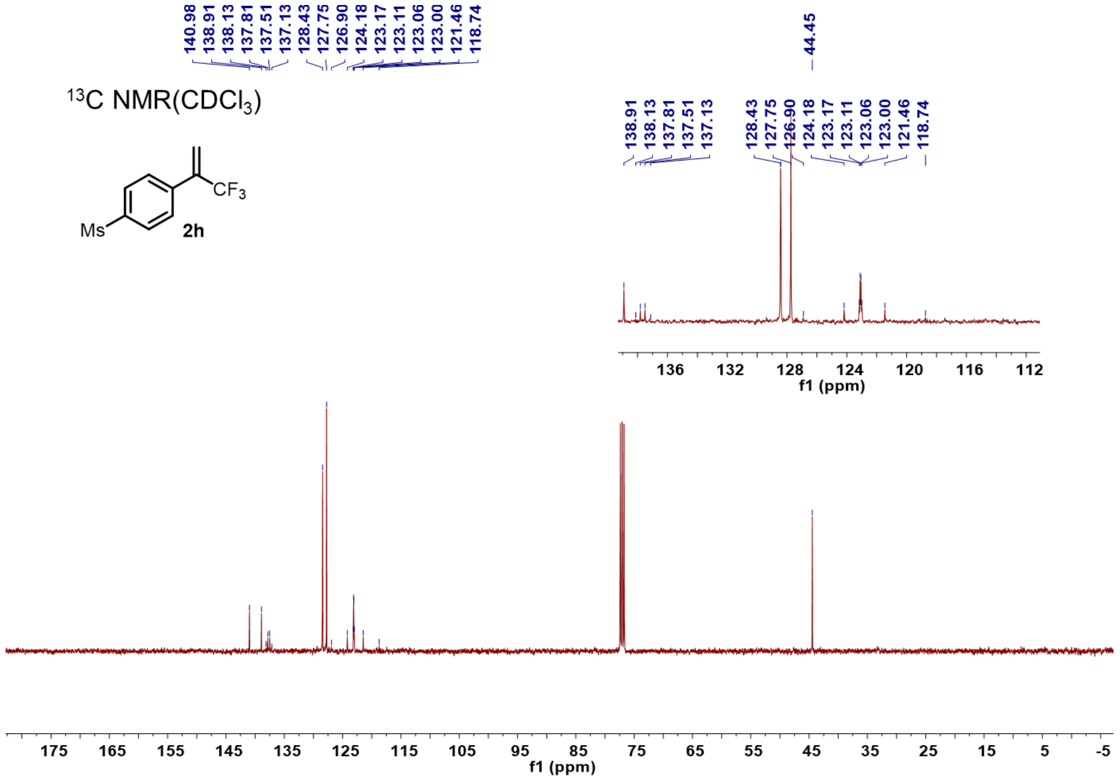

Supplementary Figure 26. ^{13}C NMR Spectrum of **2h**.

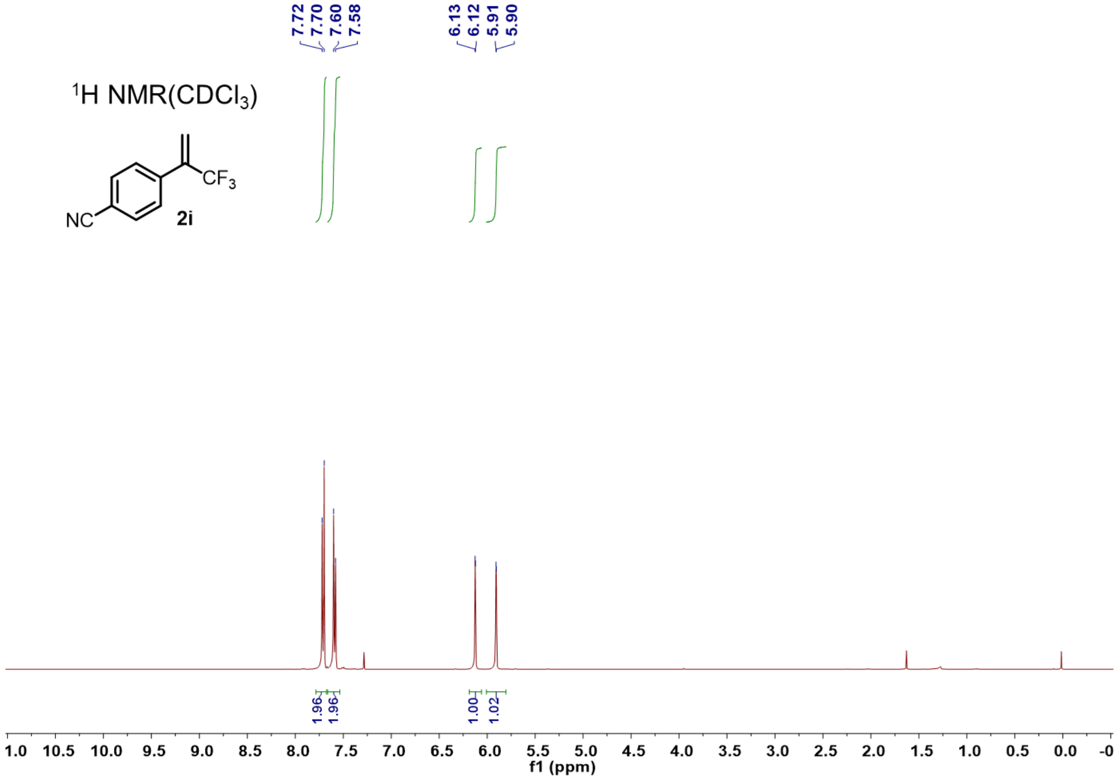

Supplementary Figure 27. ^{1}H NMR Spectrum of **2i**.

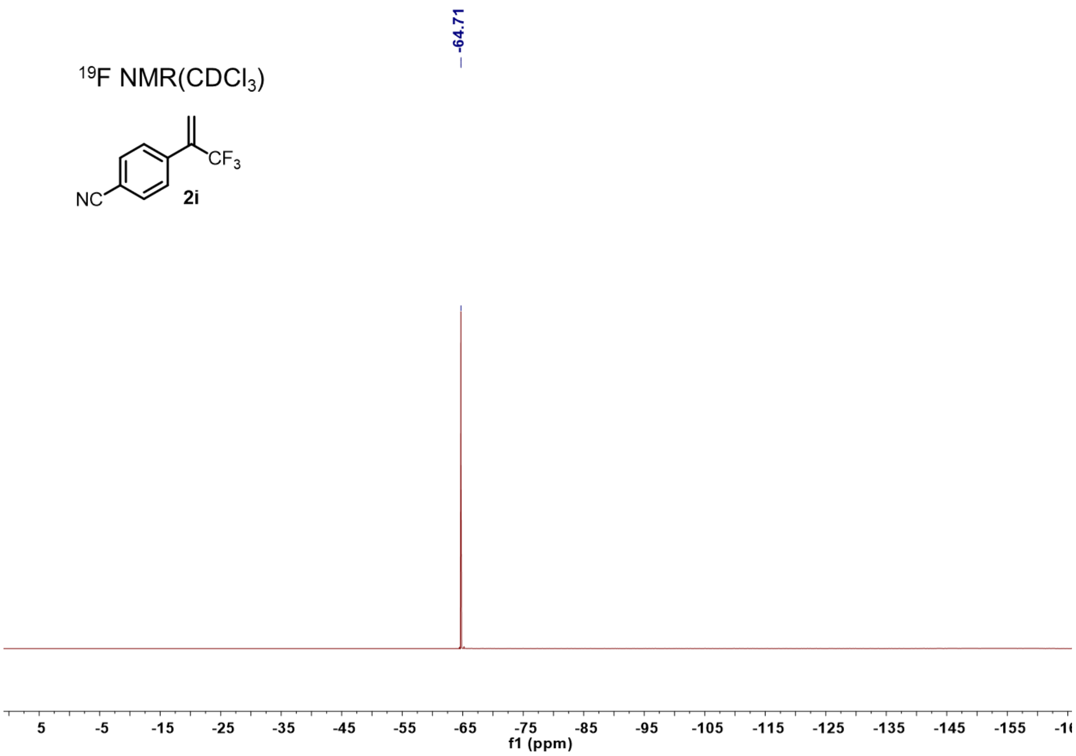

Supplementary Figure 28. ^{19}F NMR Spectrum of **2i**.

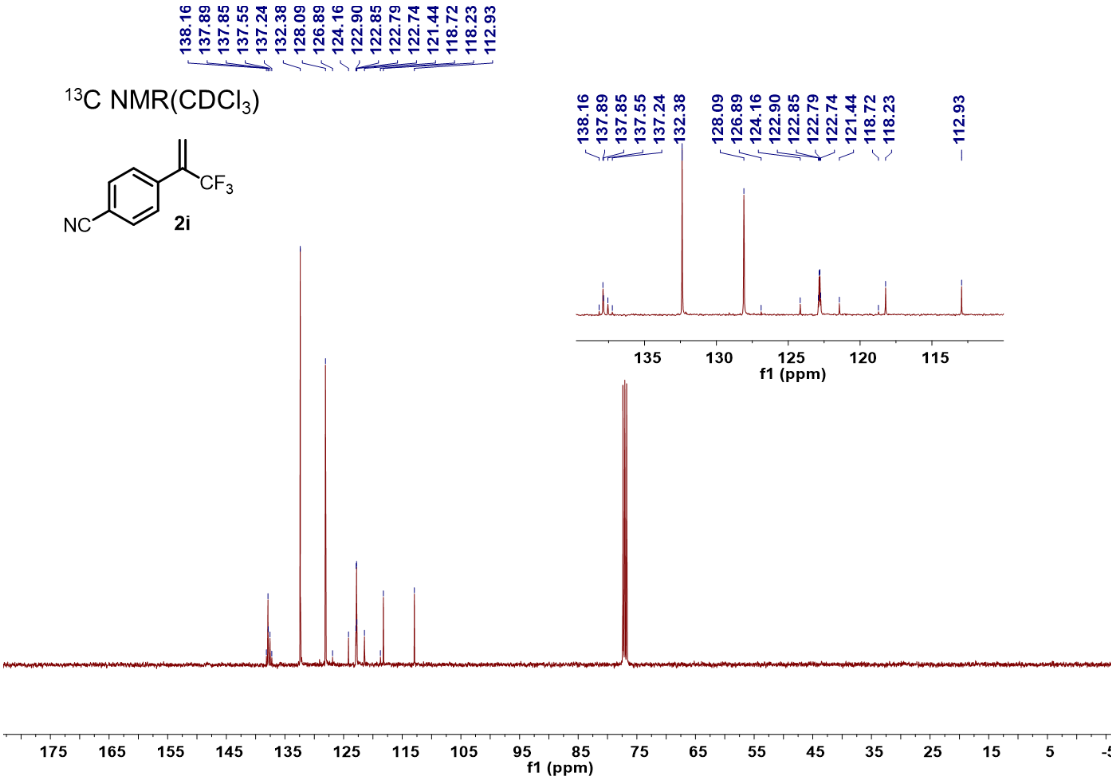

Supplementary Figure 29. ^{13}C NMR Spectrum of **2i**.

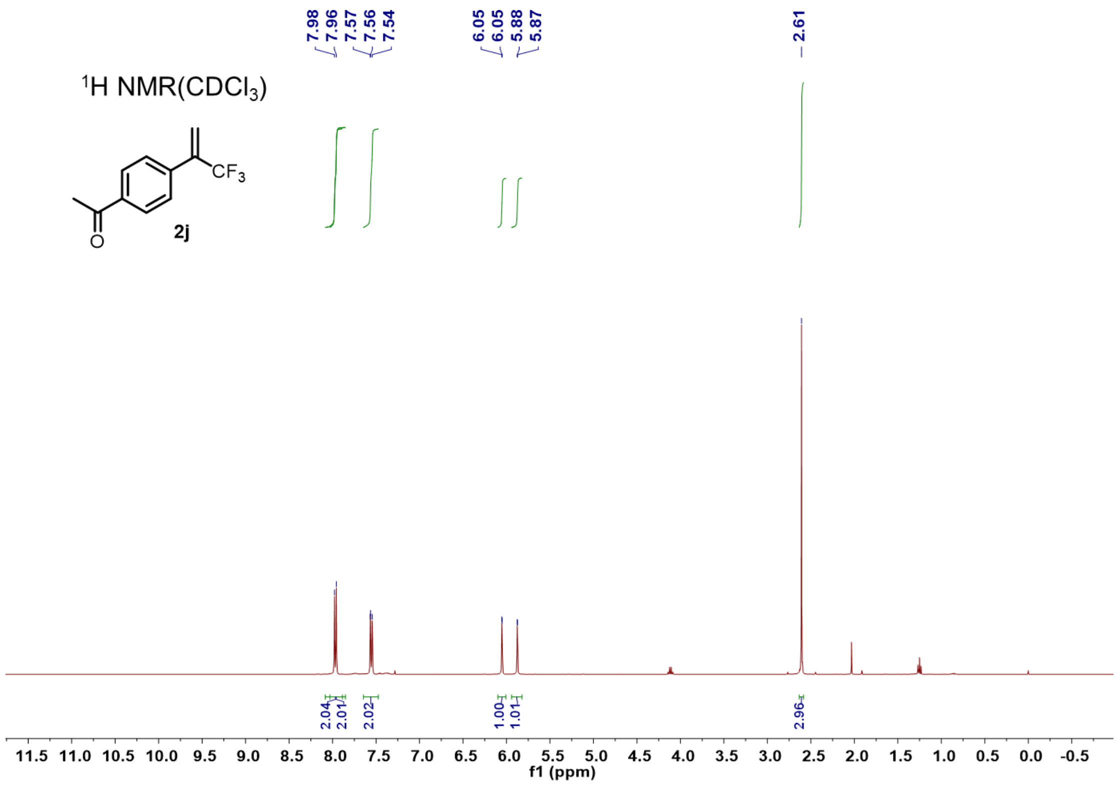

Supplementary Figure 30. ^{1}H NMR Spectrum of **2j**.

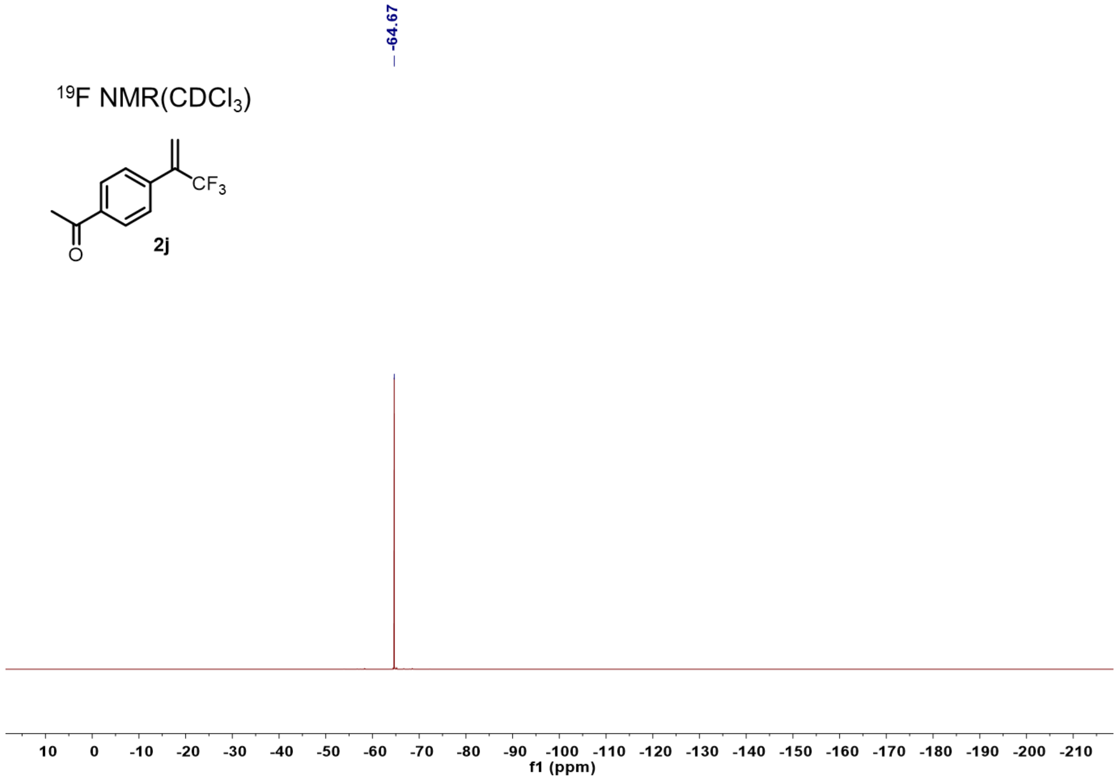

Supplementary Figure 31. ^{19}F NMR Spectrum of **2j**.

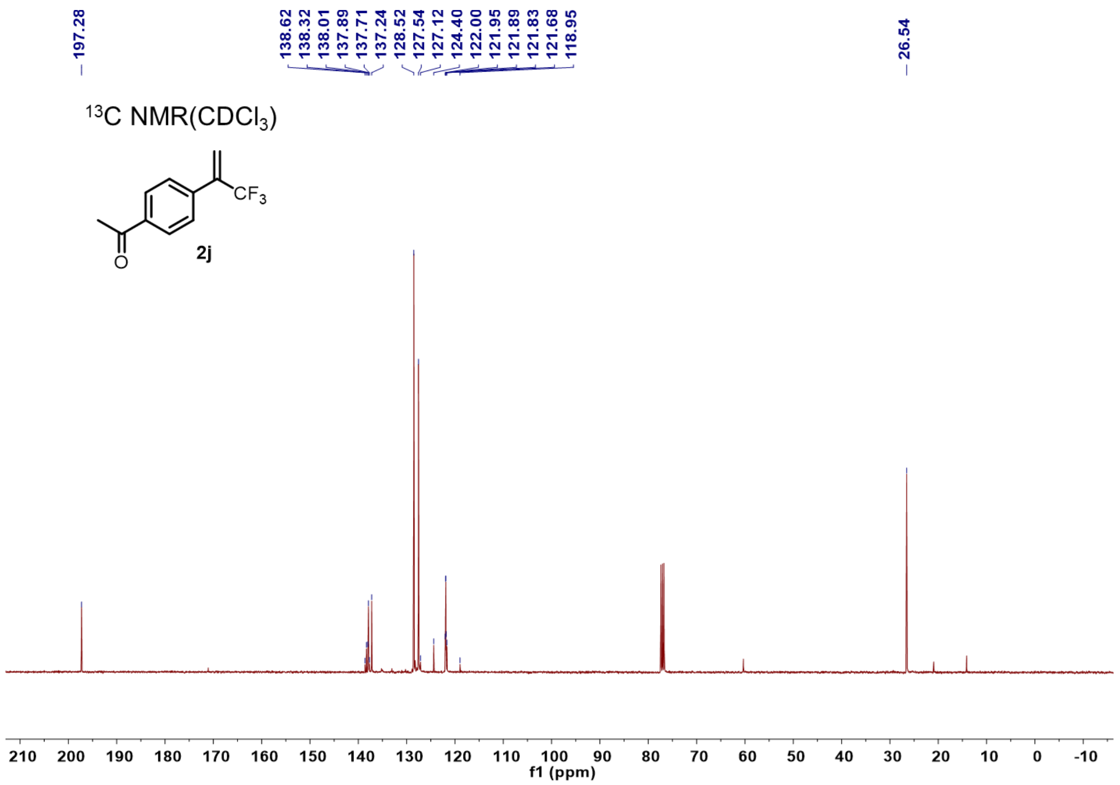

Supplementary Figure 32. ^{13}C NMR Spectrum of **2j**.

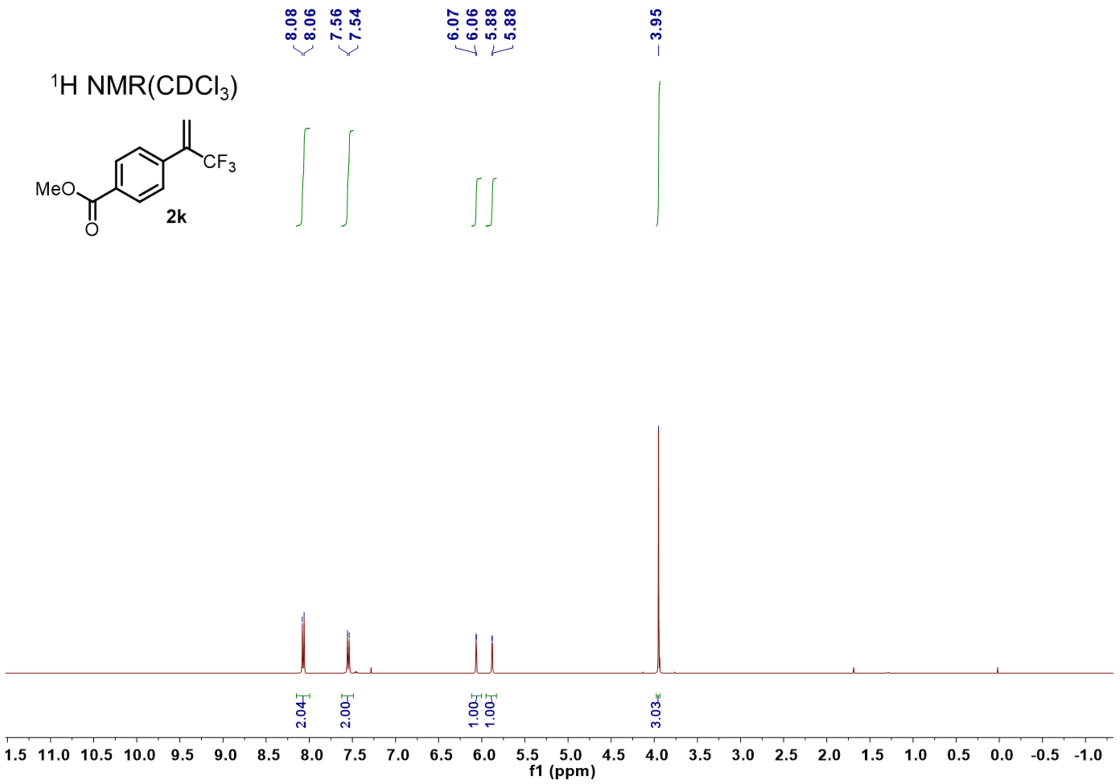

Supplementary Figure 33. ^{1}H NMR Spectrum of **2k**.

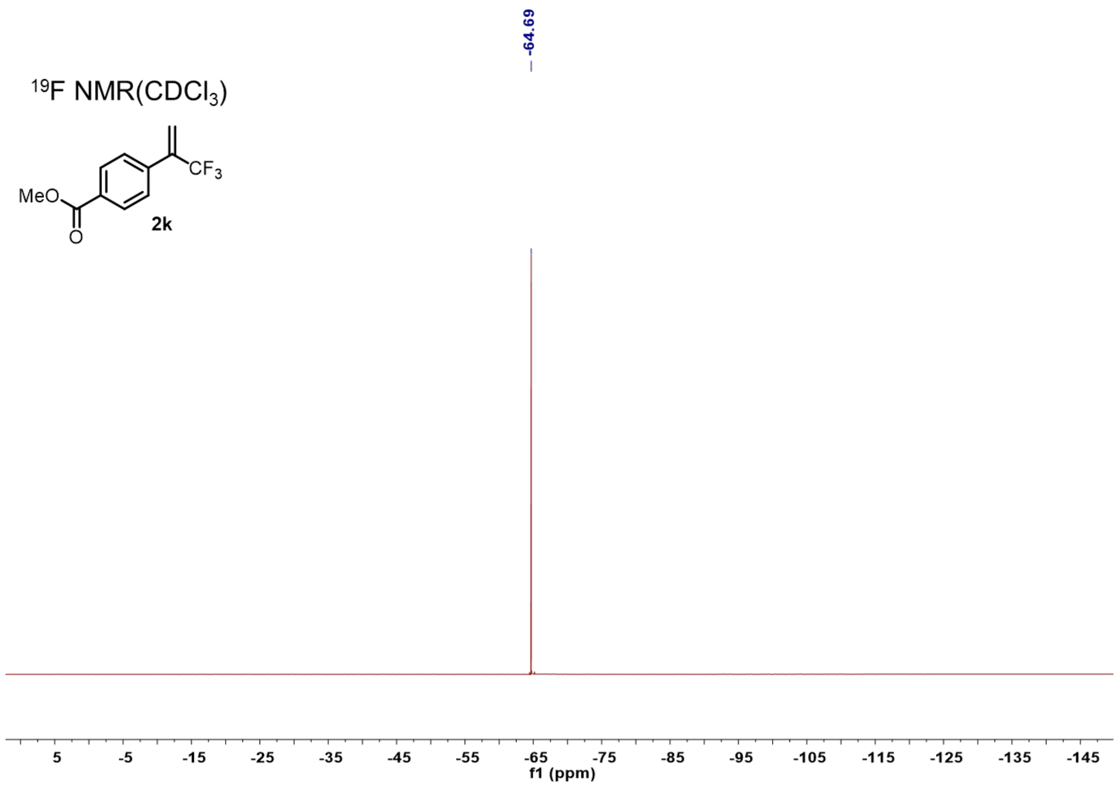

Supplementary Figure 34. ^{19}F NMR Spectrum of **2k**.

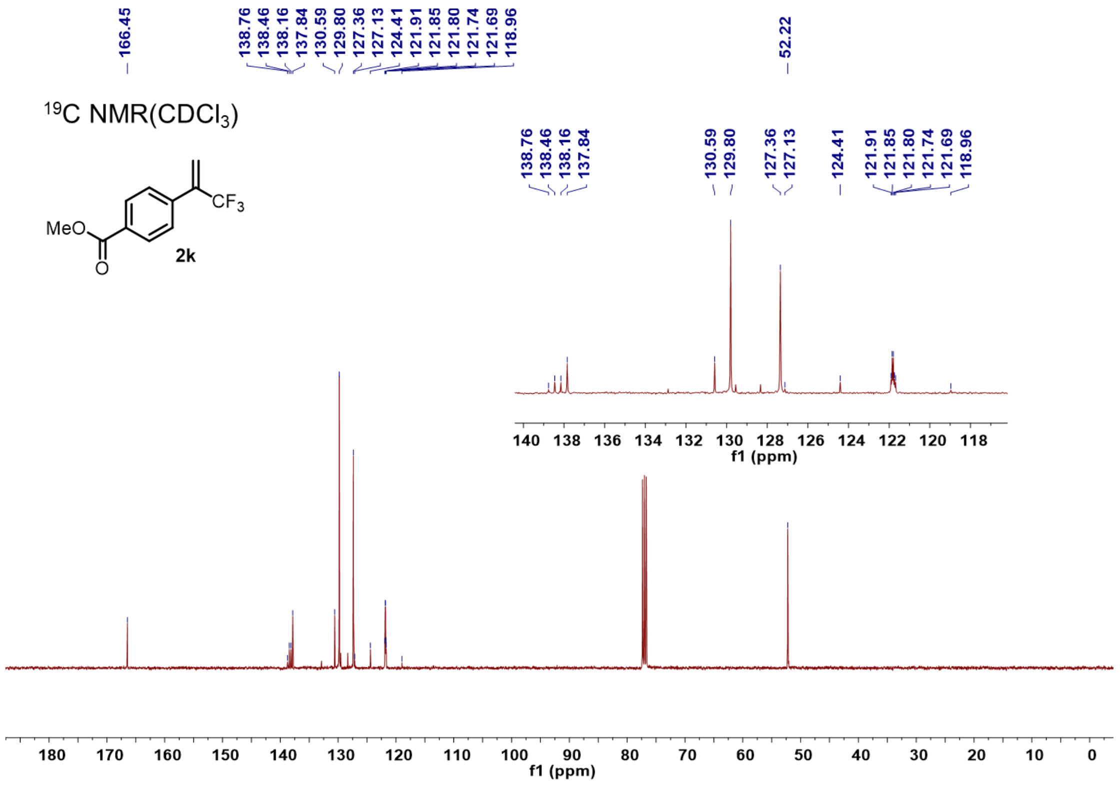

Supplementary Figure 35. ^{13}C NMR Spectrum of **2k**.

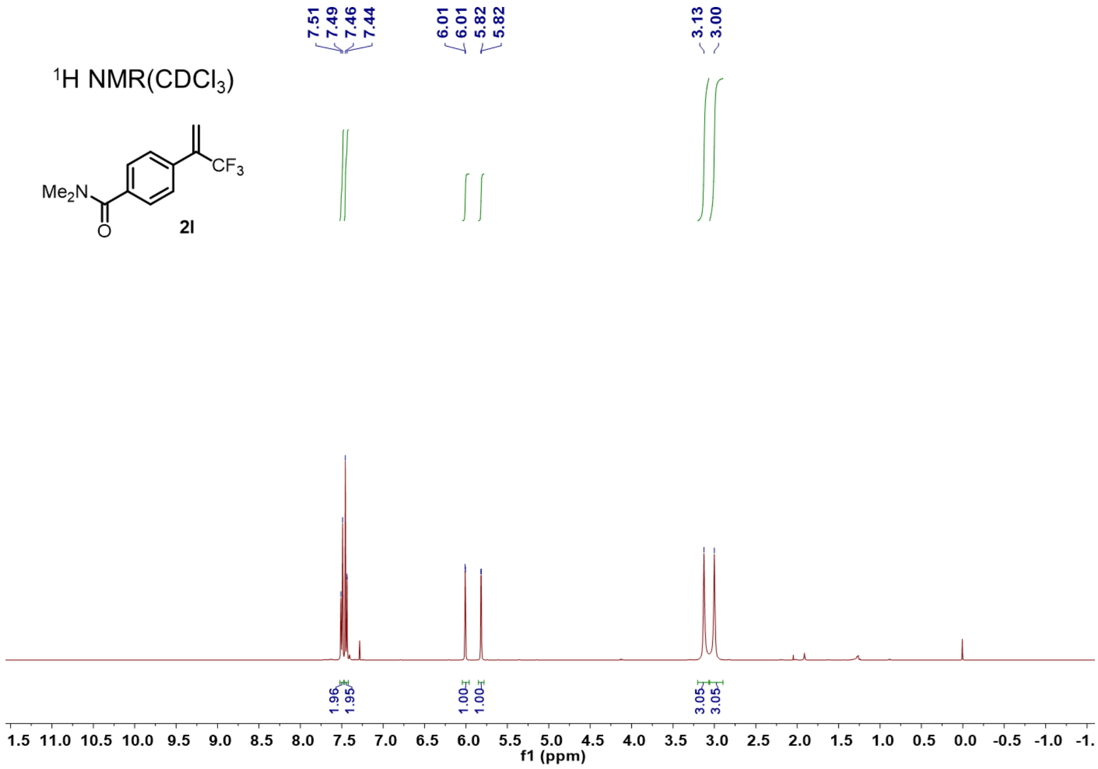

Supplementary Figure 36. ^{1}H NMR Spectrum of **2l**.

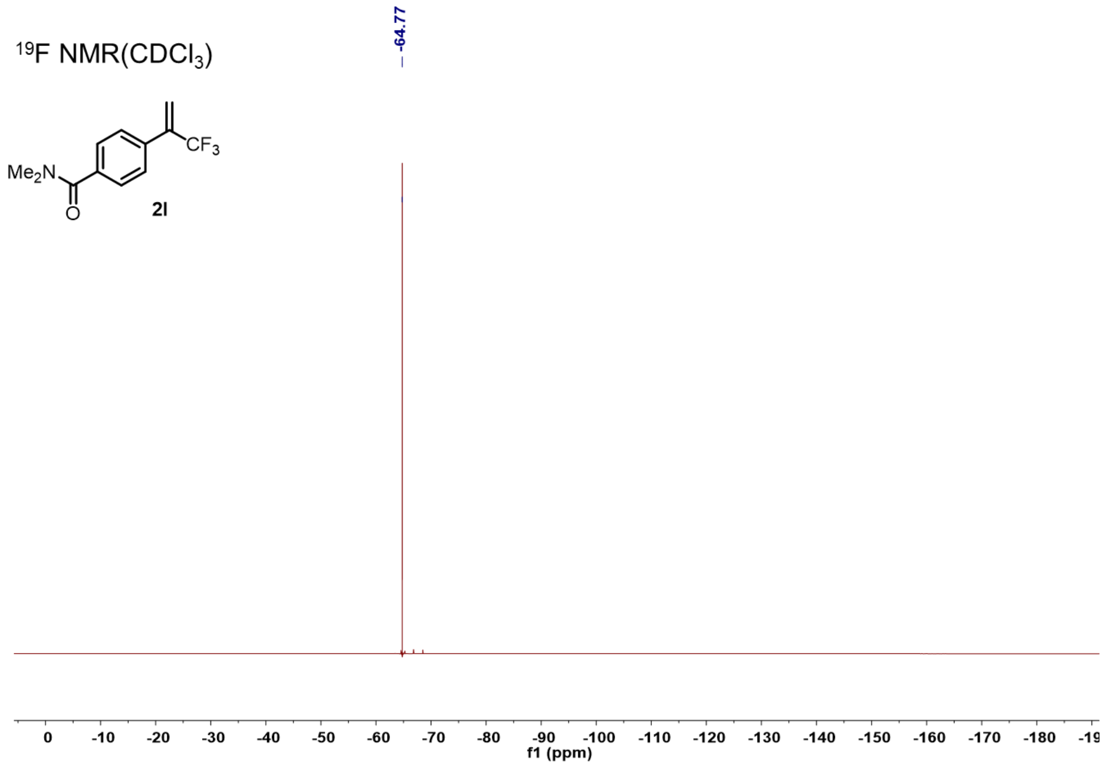

Supplementary Figure 37. ^{19}F NMR Spectrum of **2l**.

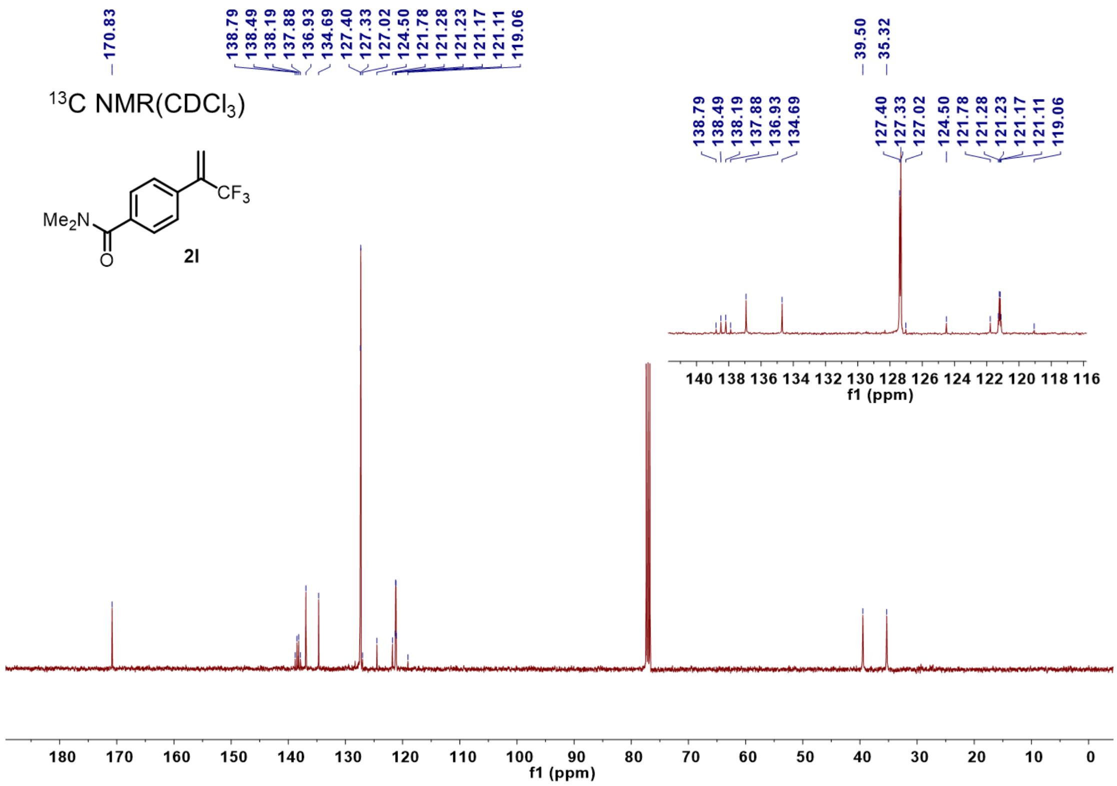

Supplementary Figure 38. ^{13}C NMR Spectrum of **2l**.

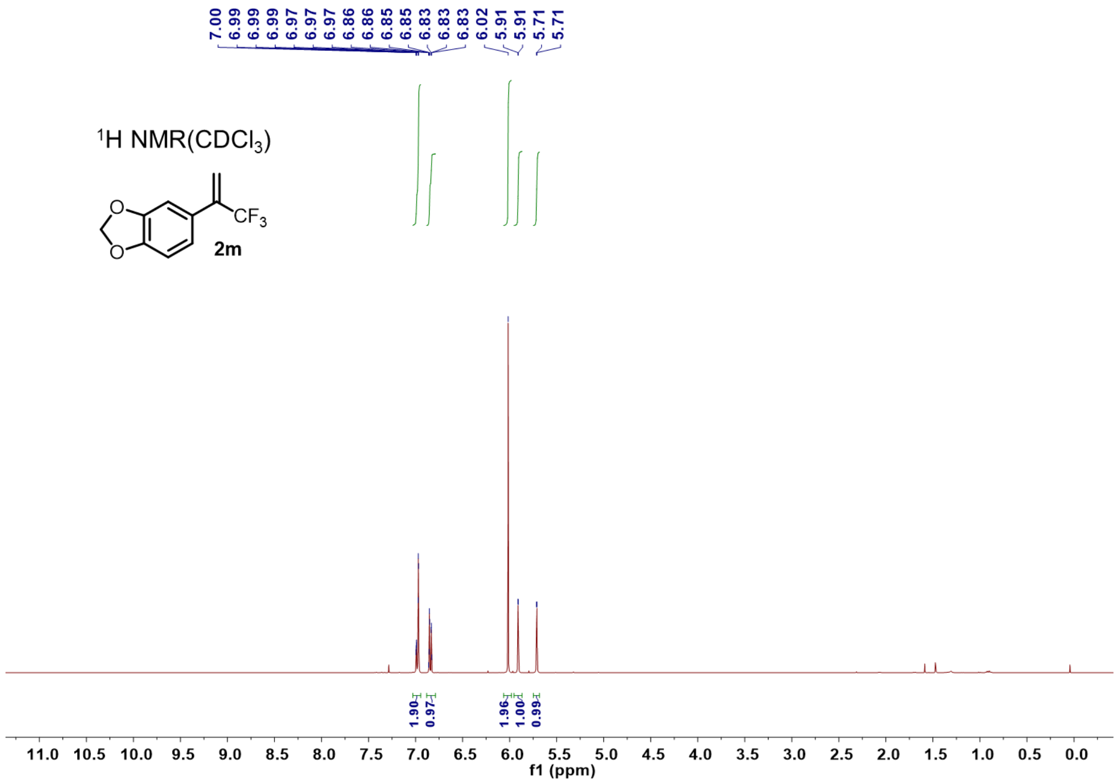

Supplementary Figure 39. ^{1}H NMR Spectrum of **2m**.

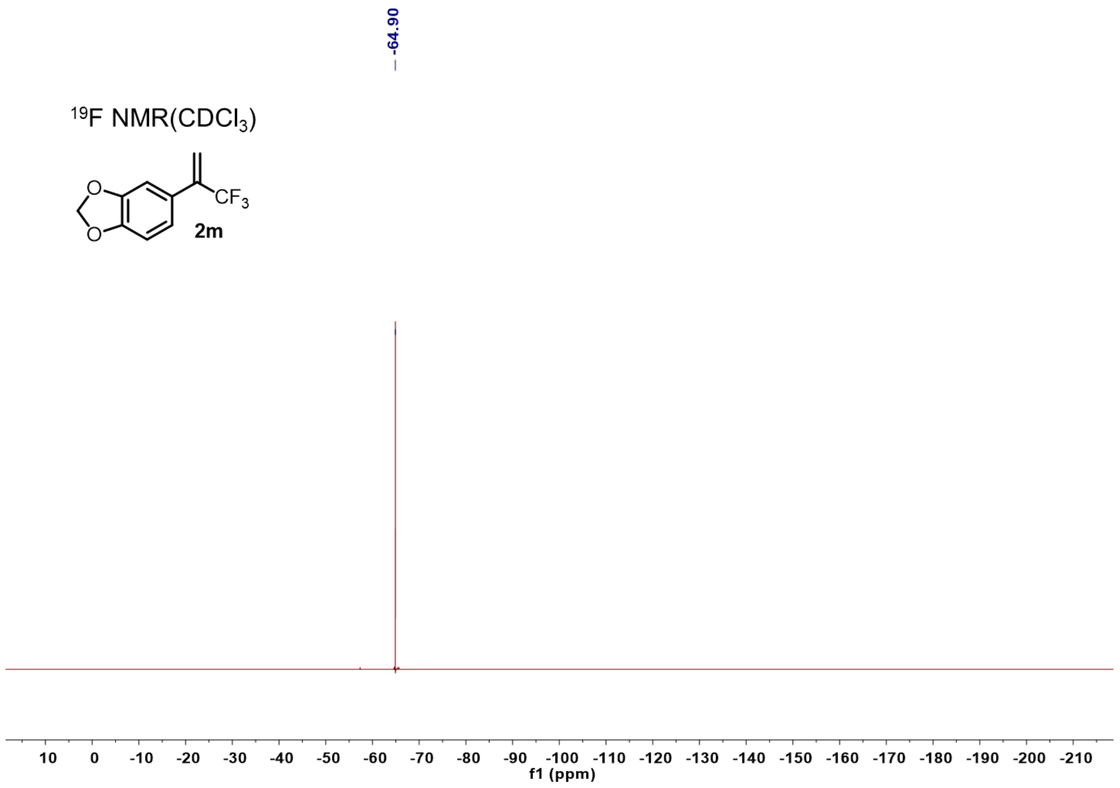

Supplementary Figure 40. ^{19}F NMR Spectrum of **2m**.

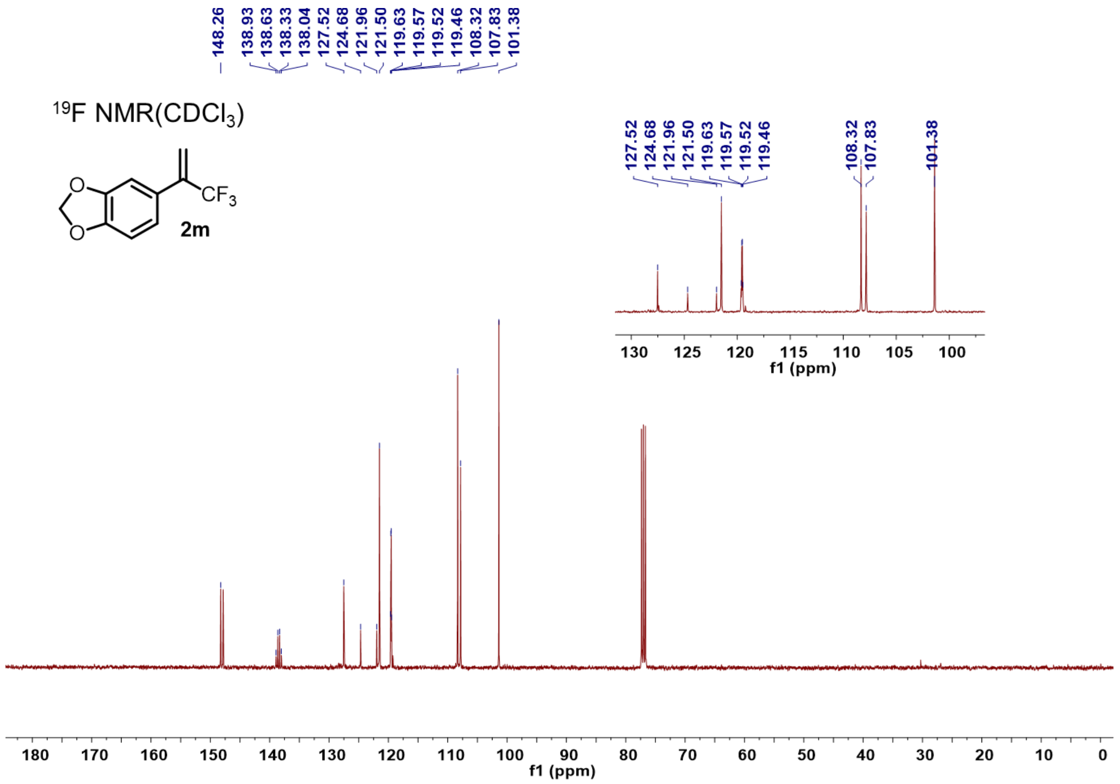

Supplementary Figure 41. ^{13}C NMR Spectrum of **2m**.

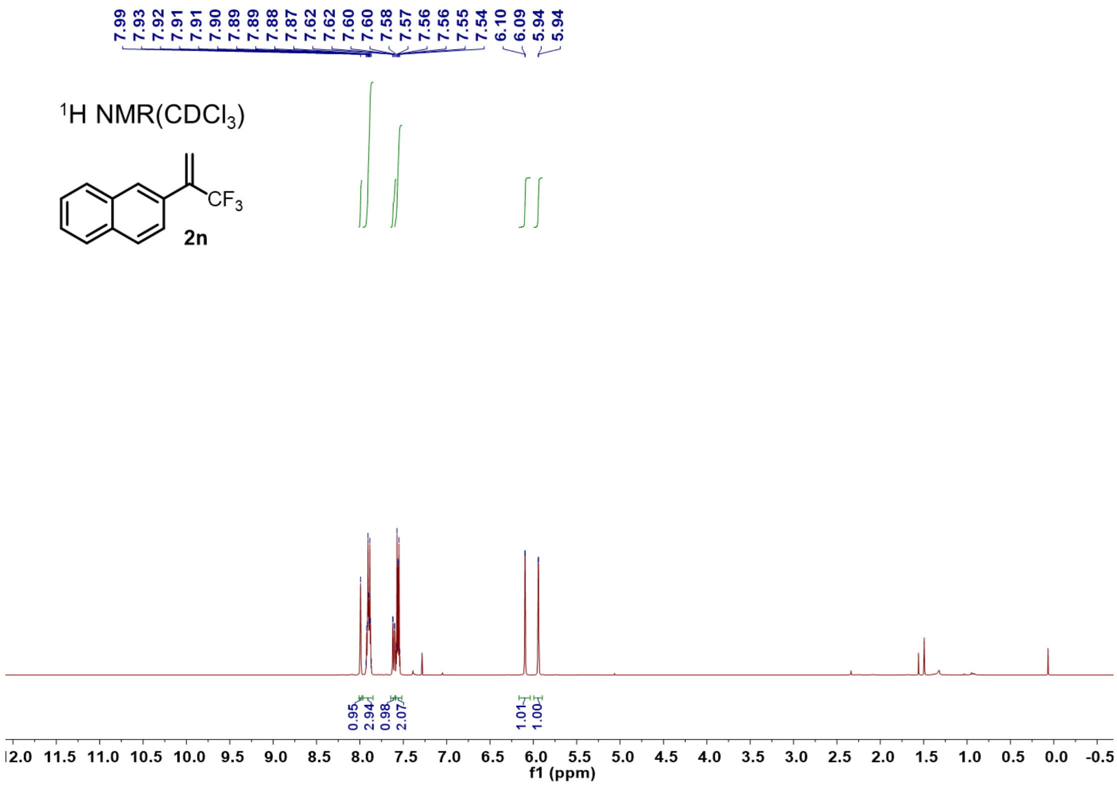

Supplementary Figure 42. ^{1}H NMR Spectrum of **2n**.

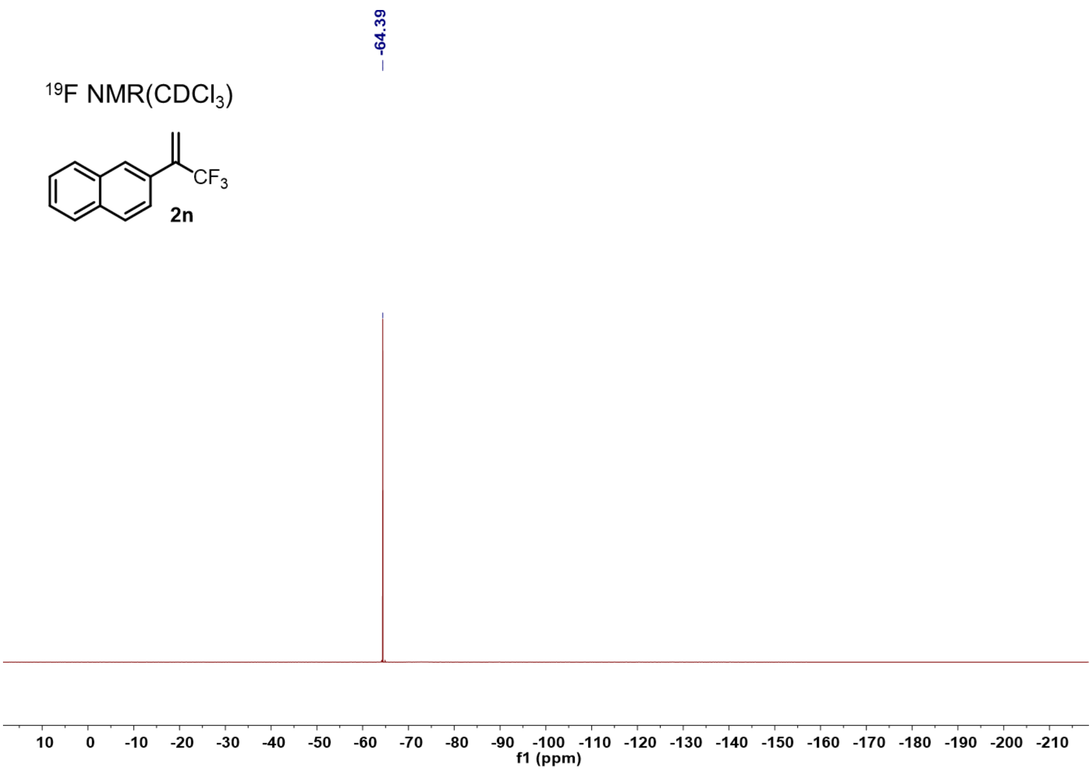

Supplementary Figure 43. ^{19}F NMR Spectrum of **2n**.

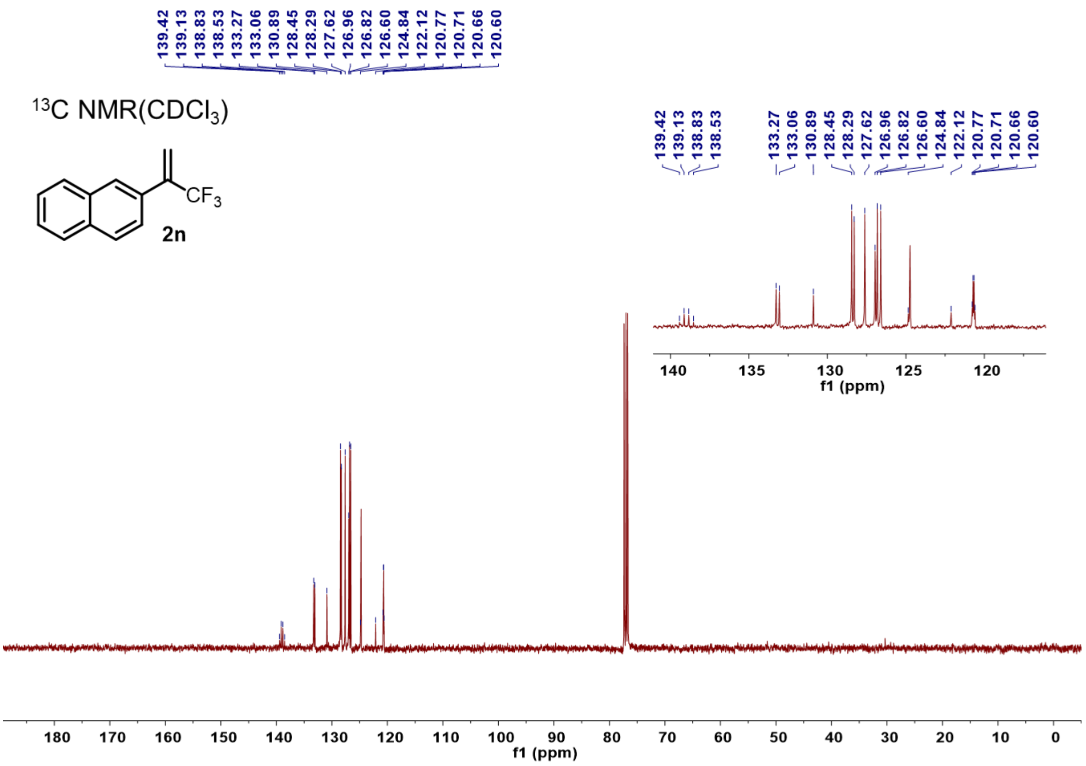

Supplementary Figure 44. ^{13}C NMR Spectrum of **2n**.

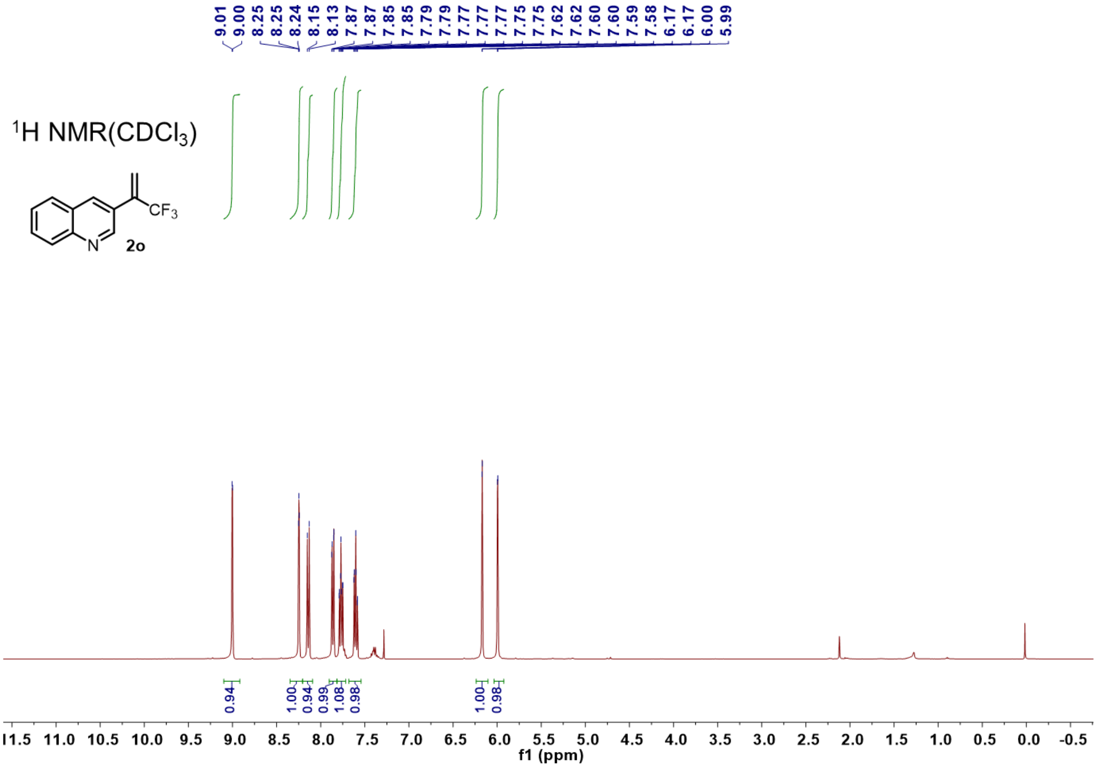

Supplementary Figure 45. ^{1}H NMR Spectrum of **2o**.

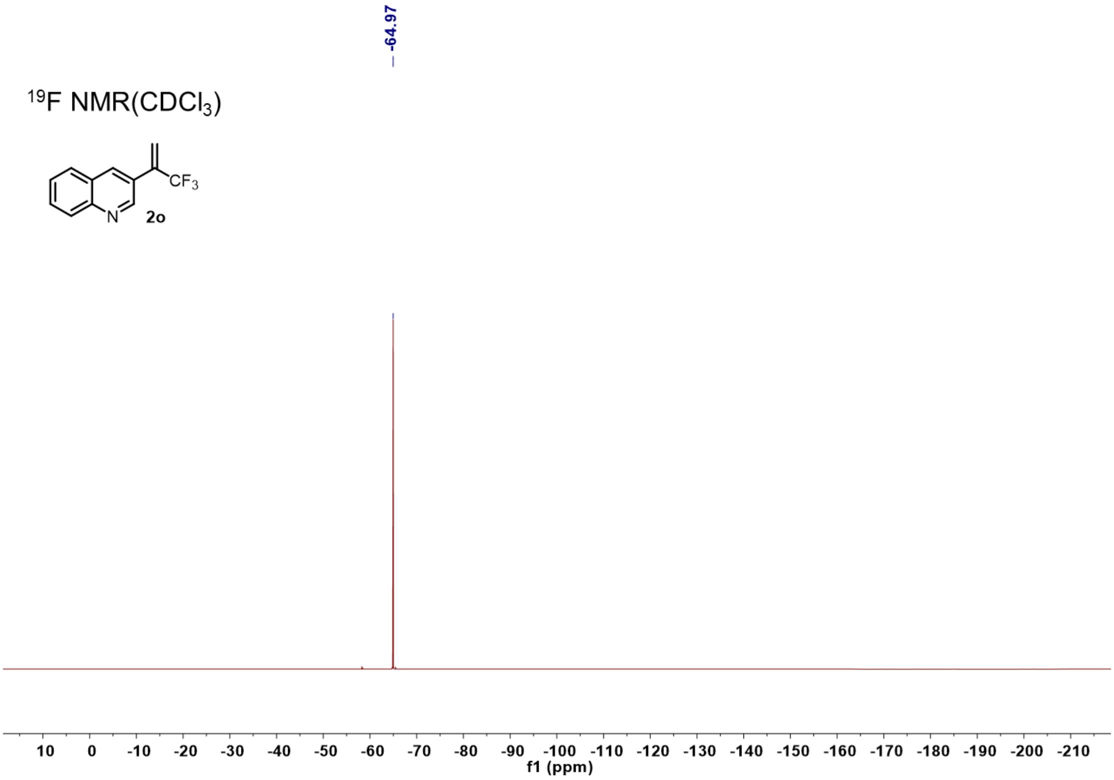

Supplementary Figure 46. ^{19}F NMR Spectrum of **2o**.

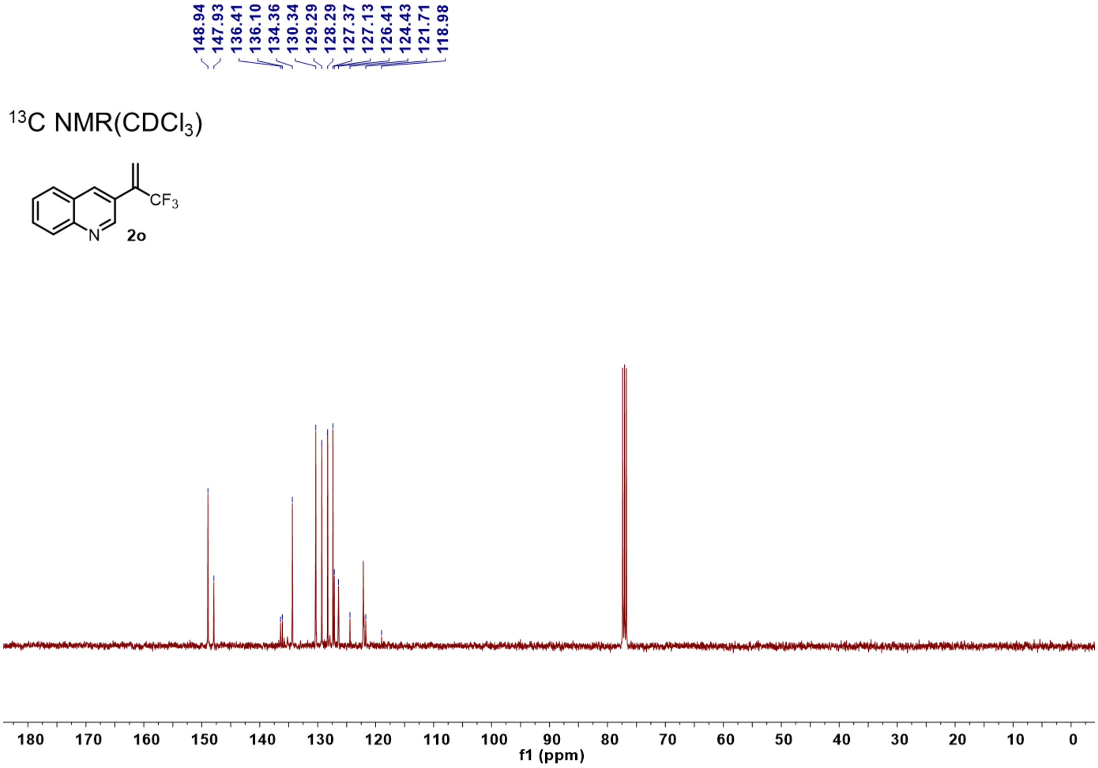

Supplementary Figure 47. ^{13}C NMR Spectrum of **2o**.

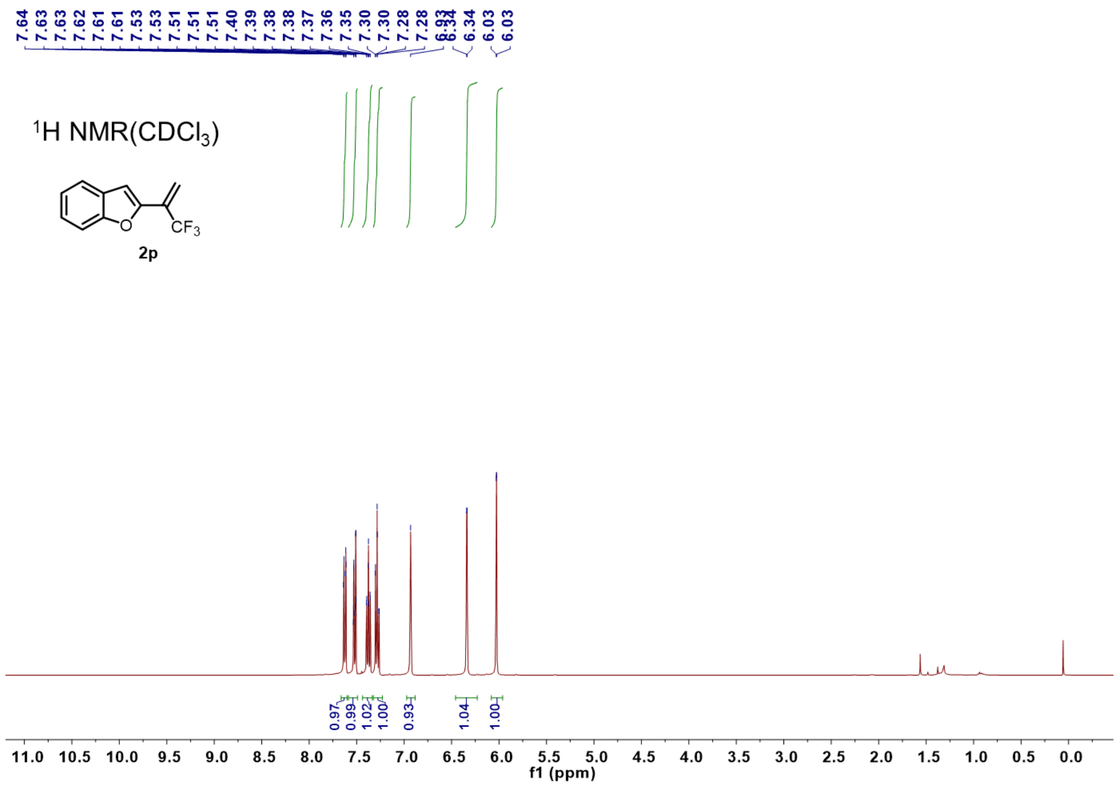

Supplementary Figure 48. ^{1}H NMR Spectrum of **2p**.

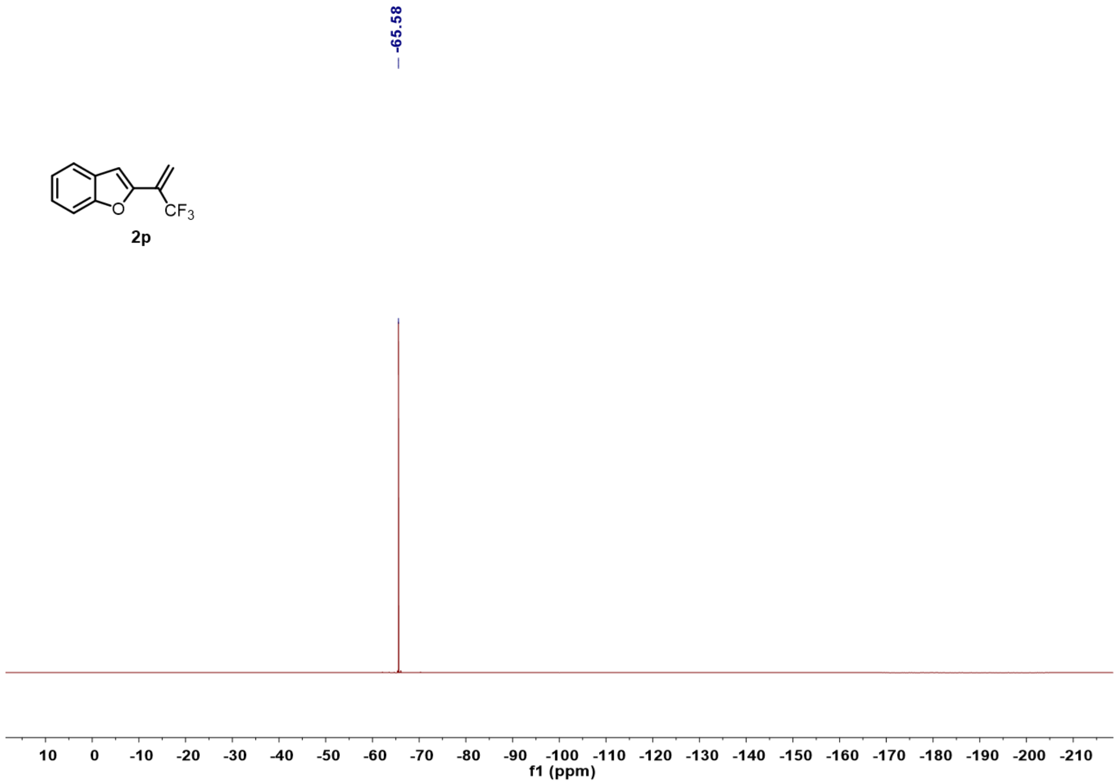

Supplementary Figure 49. ^{19}F NMR Spectrum of **2p**.

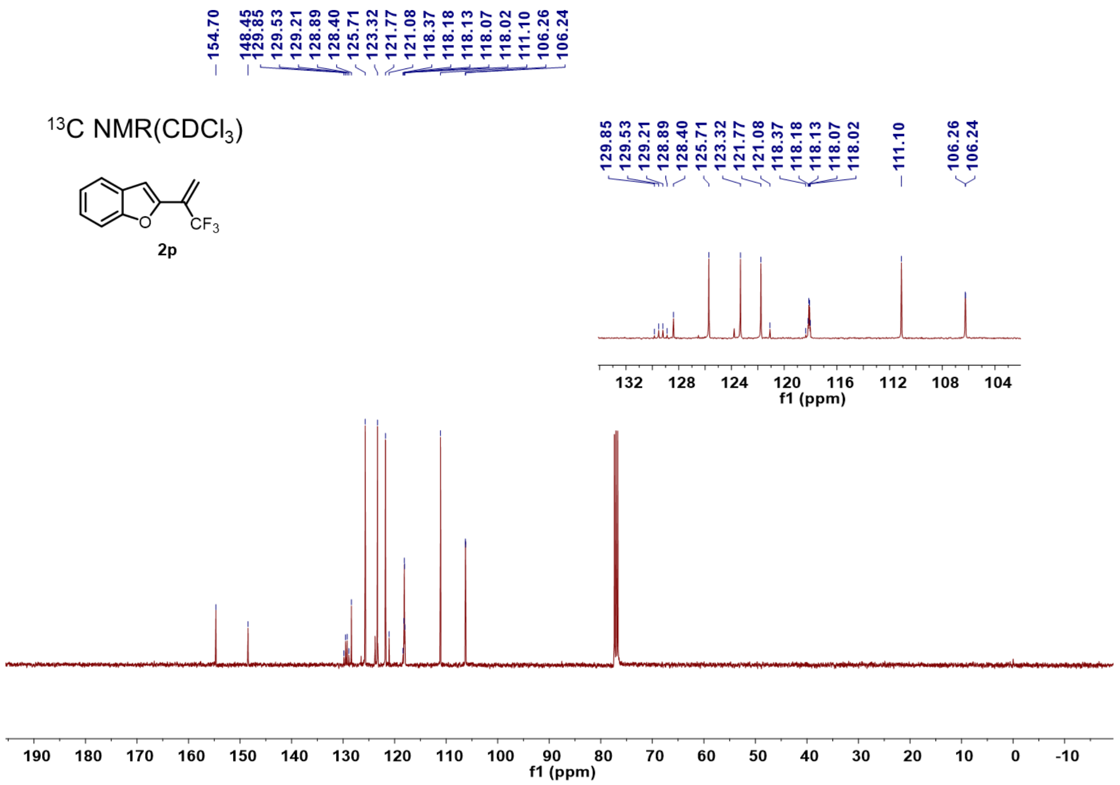

Supplementary Figure 50. ^{13}C NMR Spectrum of **2p**.

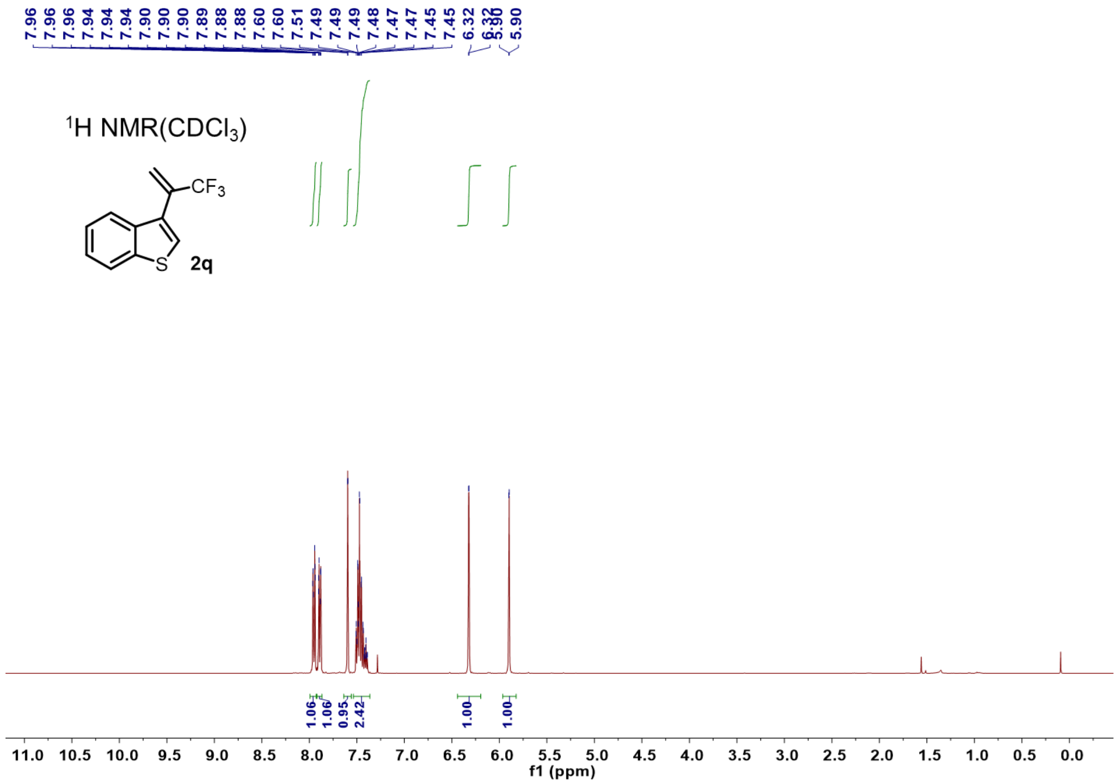

Supplementary Figure 51. ^{1}H NMR Spectrum of **2q**.

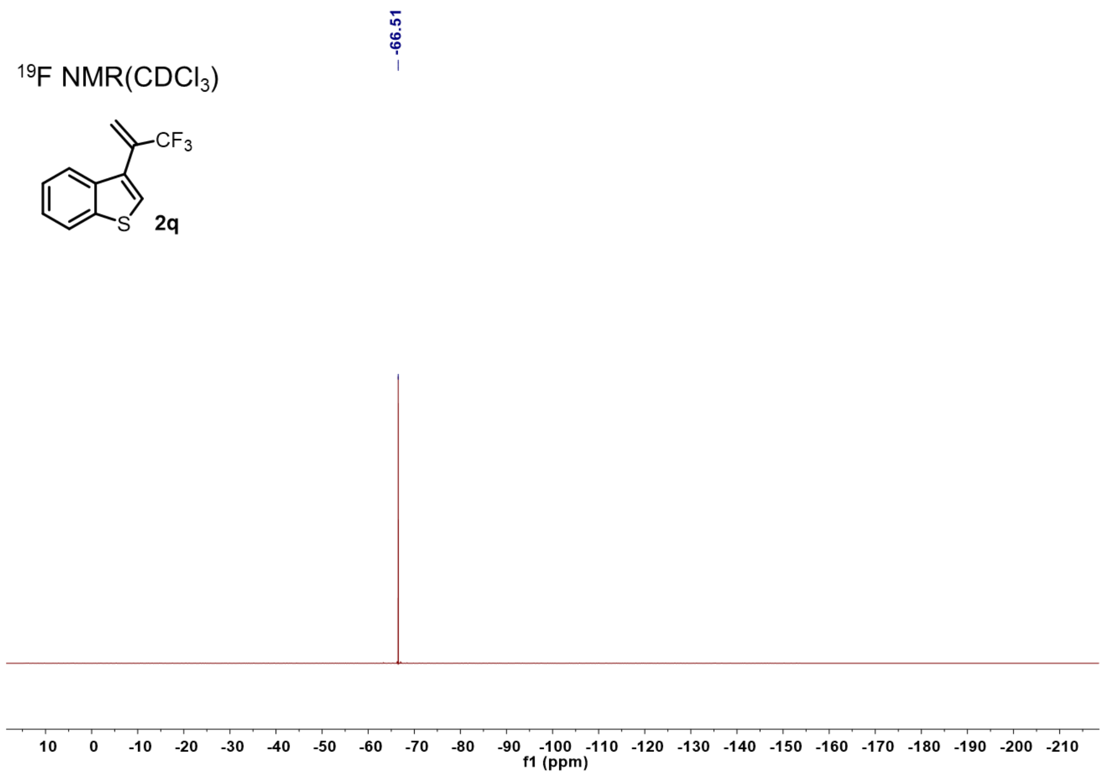

Supplementary Figure 52. ^{19}F NMR Spectrum of **2q**.

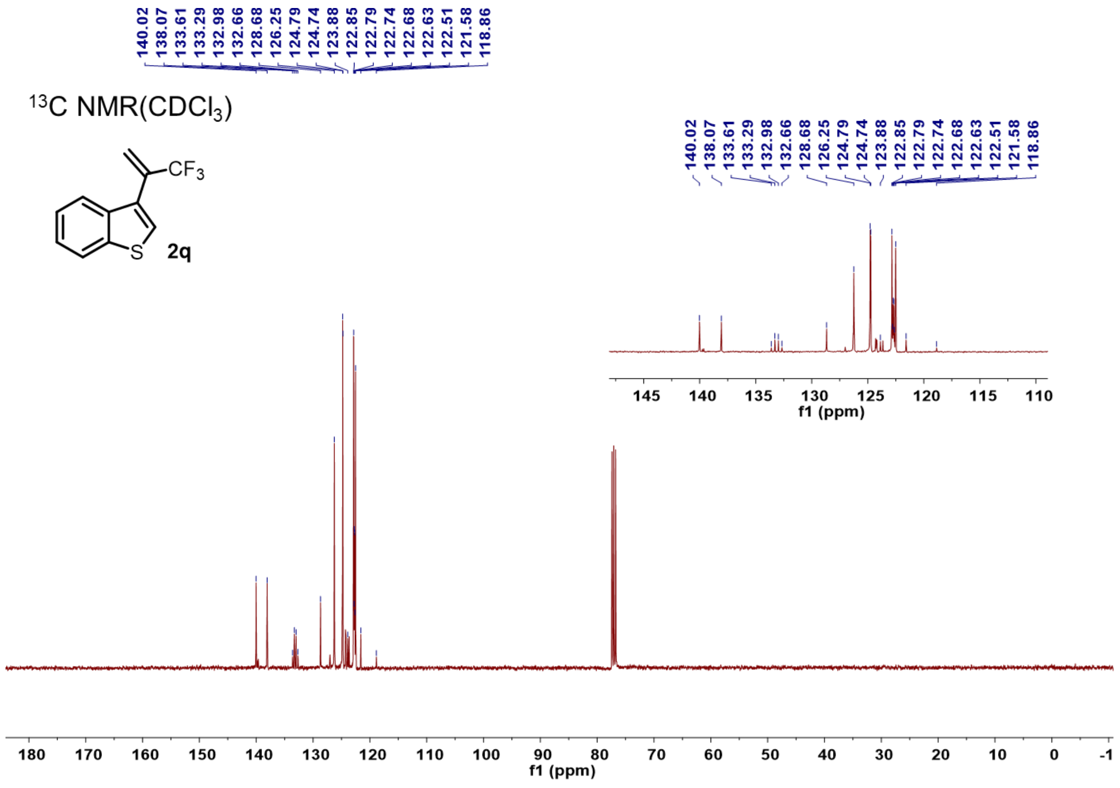

Supplementary Figure 53. ^{13}C NMR Spectrum of **2q**.

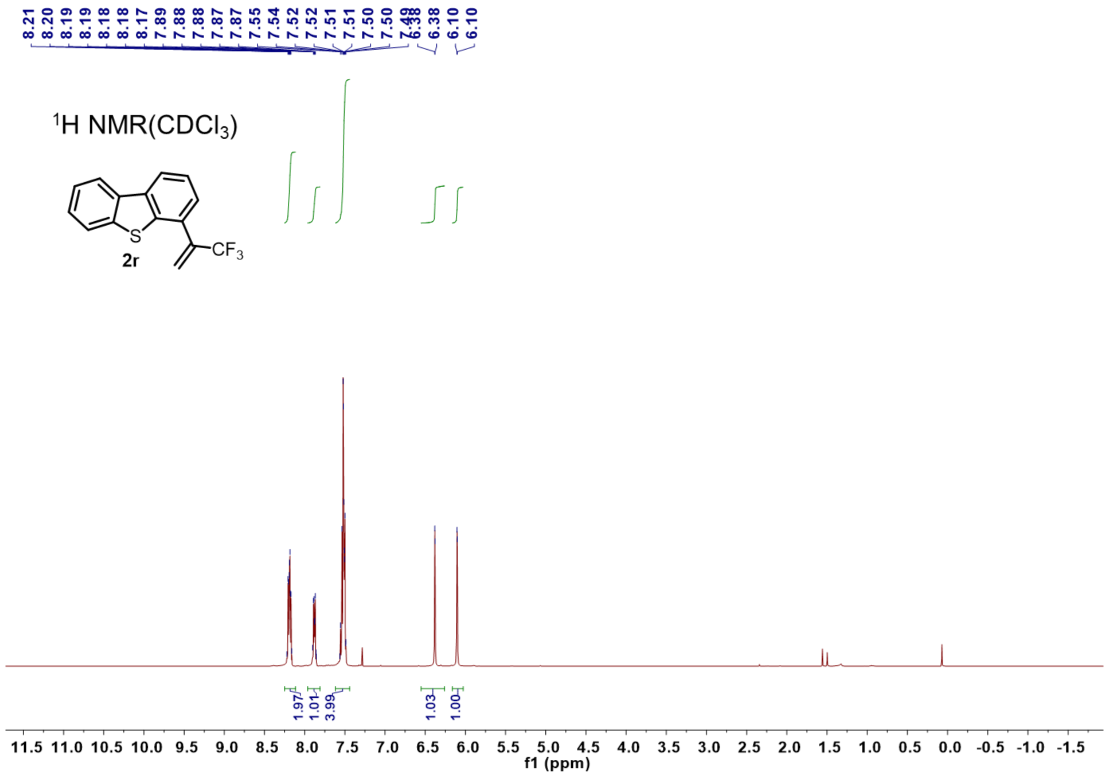

Supplementary Figure 54. ^{1}H NMR Spectrum of **2r**.

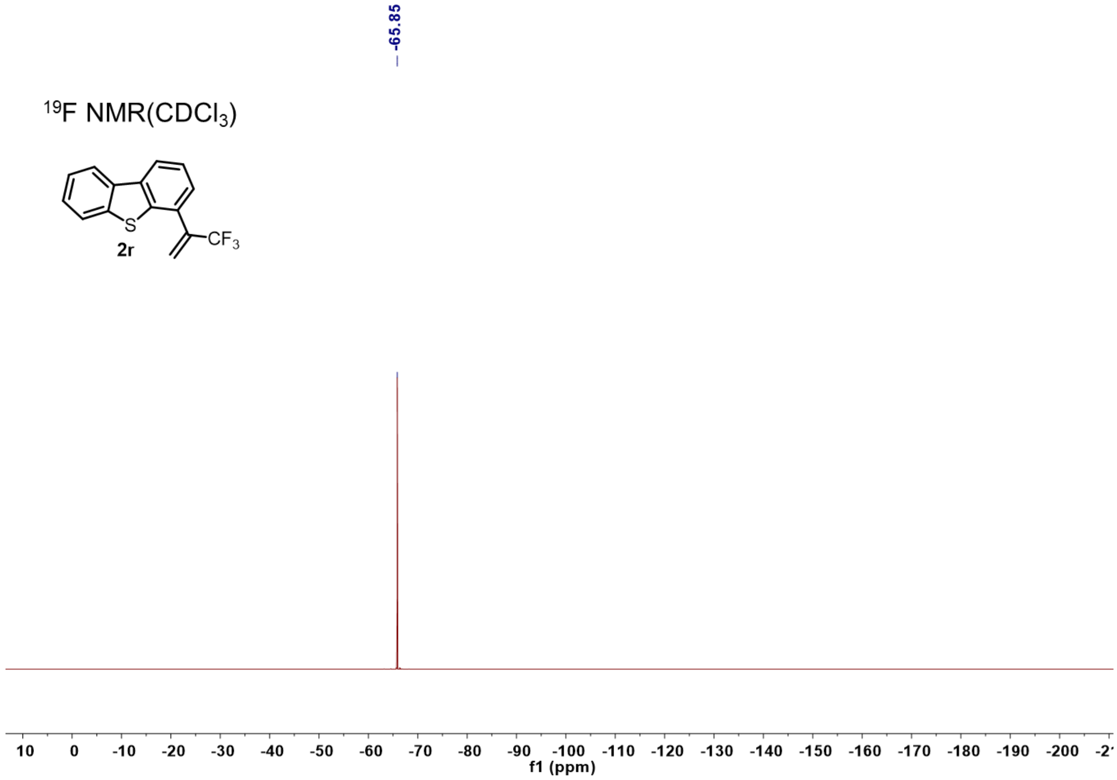

Supplementary Figure 55. ^{19}F NMR Spectrum of **2r**.

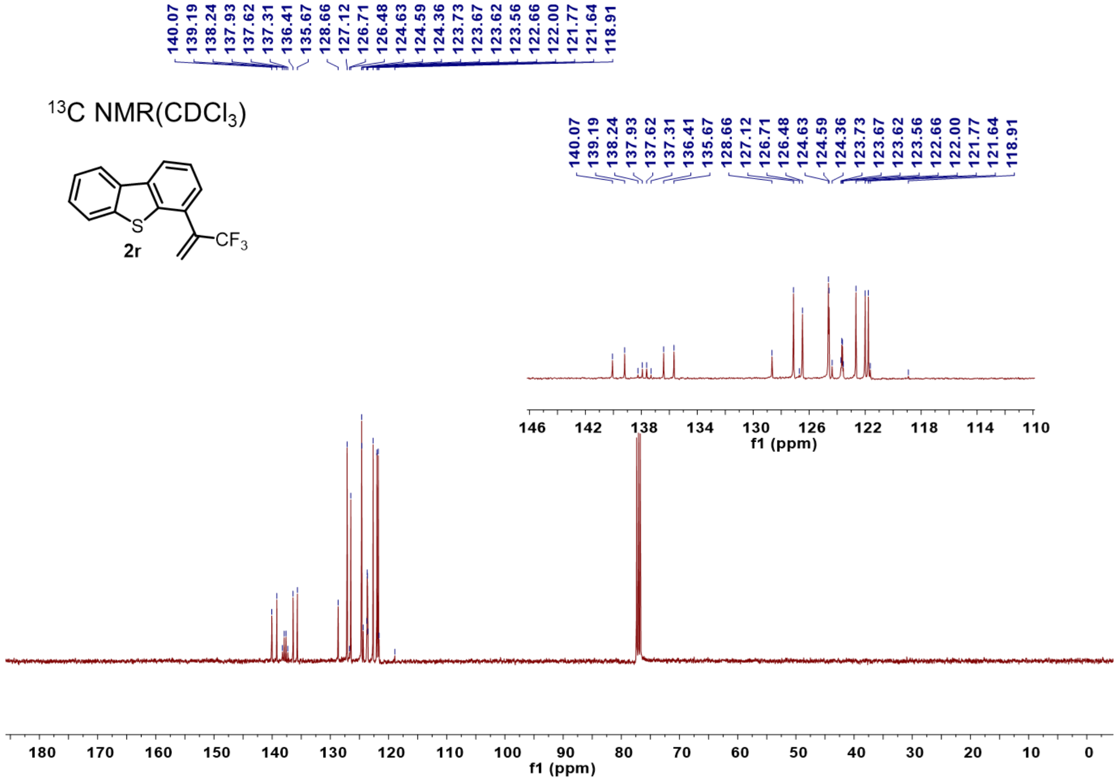

Supplementary Figure 56. ^{13}C NMR Spectrum of **2r**.

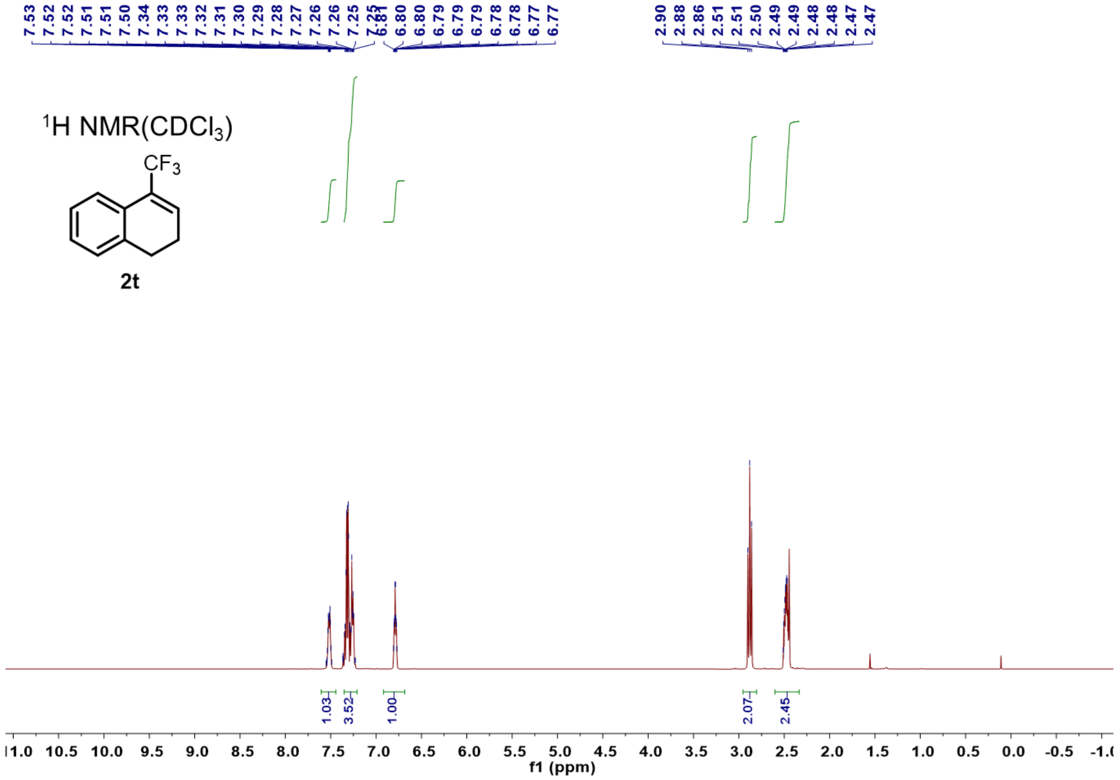

Supplementary Figure 57. ^{1}H NMR Spectrum of **2t**.

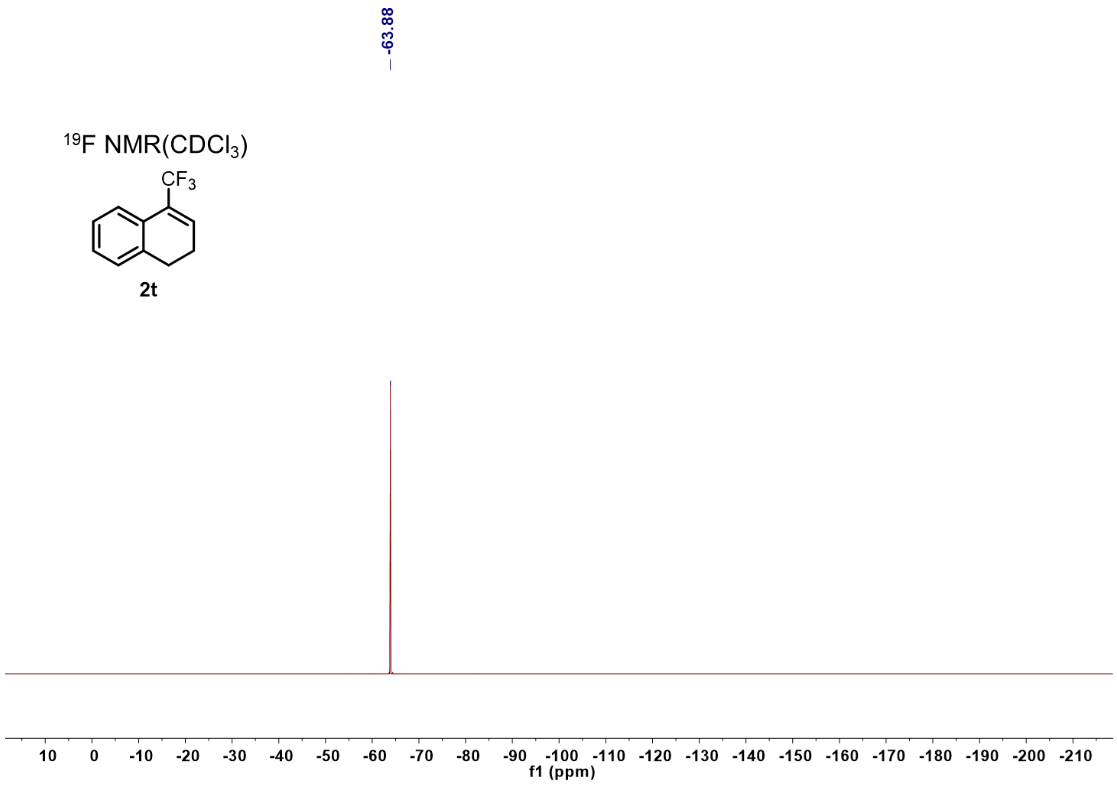

Supplementary Figure 58. ^{19}F NMR Spectrum of **2t**.

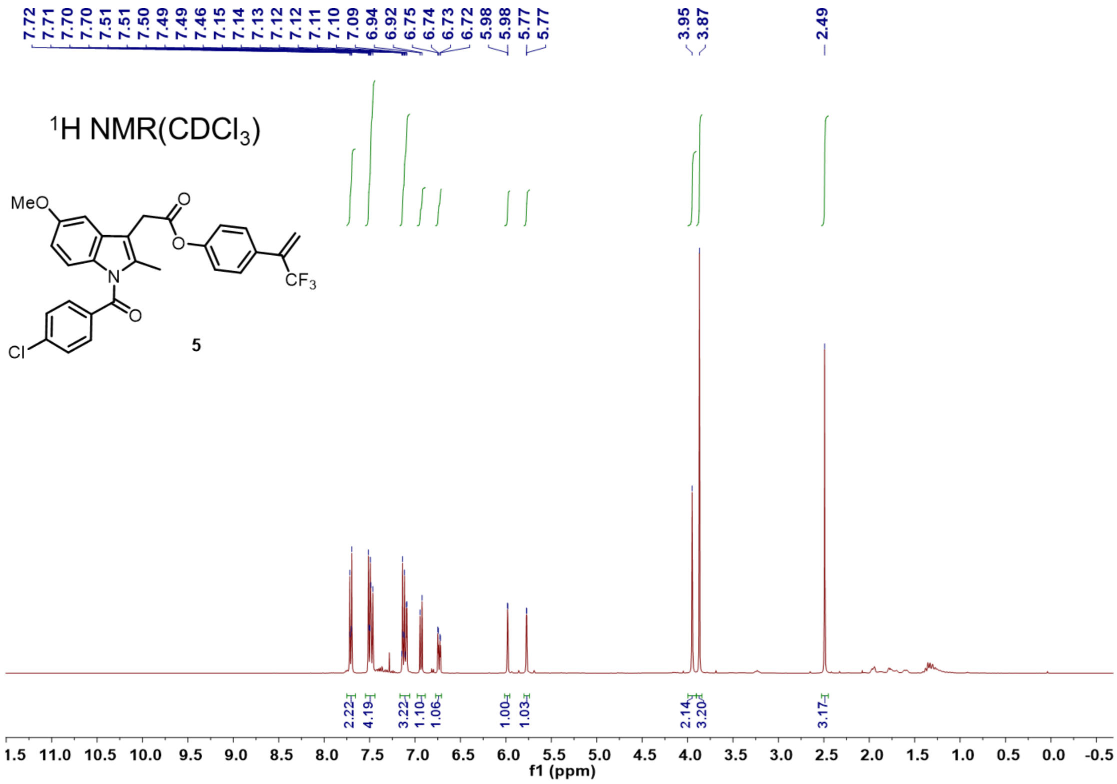

Supplementary Figure 59. ^{1}H NMR Spectrum of **5**.

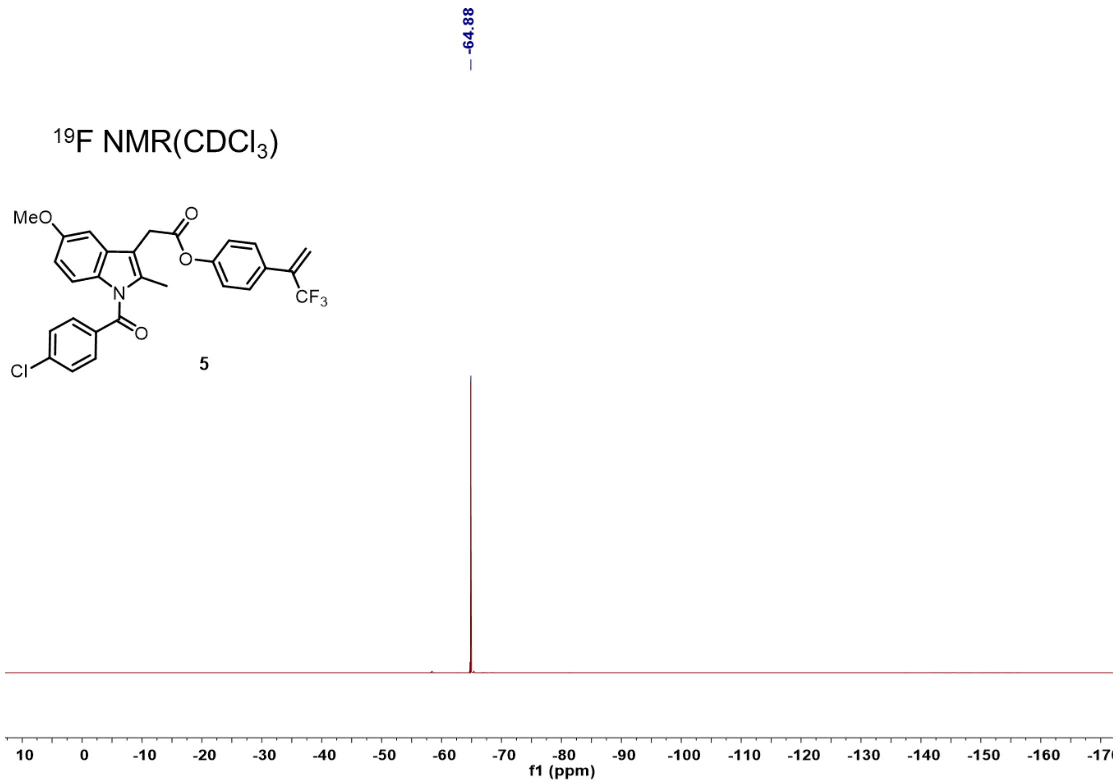

Supplementary Figure 60. ^{19}F NMR Spectrum of **5**.

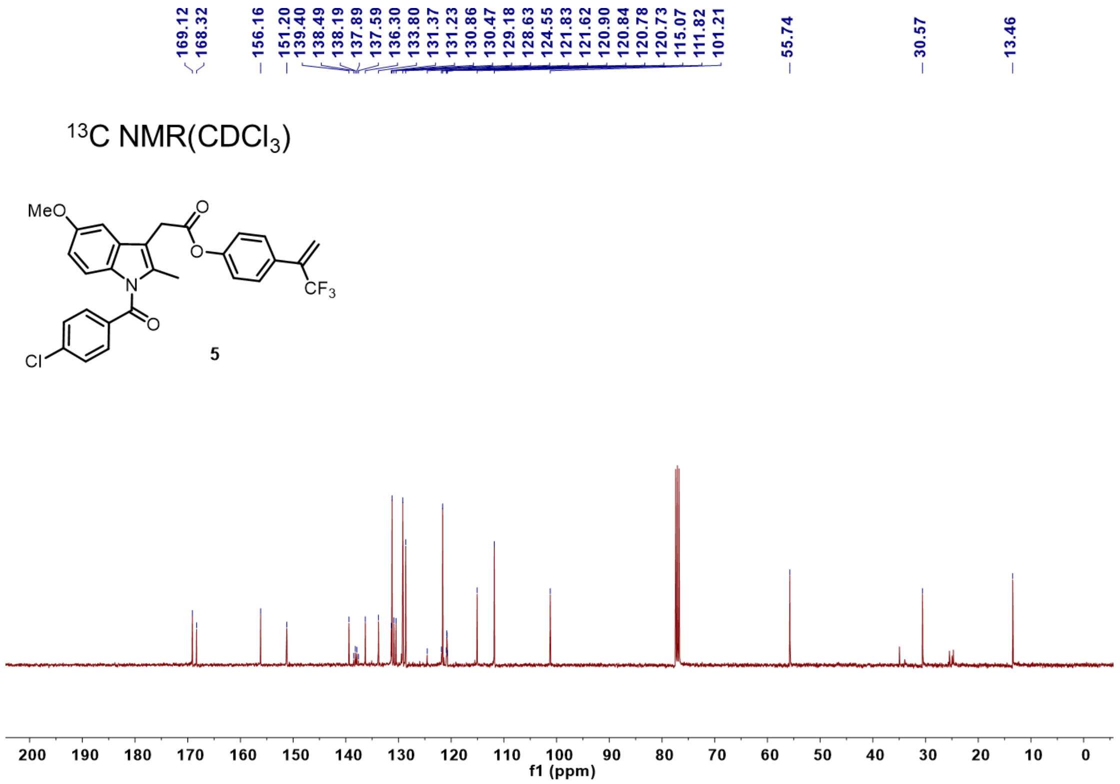

Supplementary Figure 61. ^{13}C NMR Spectrum of **5**.

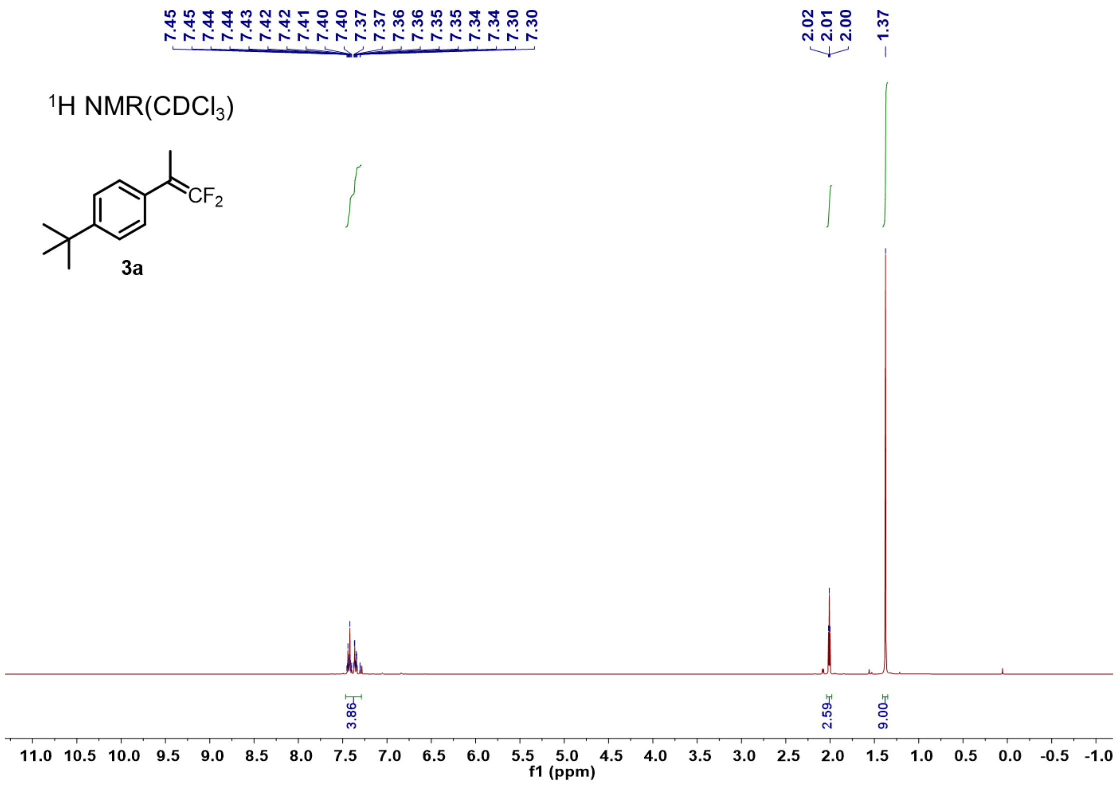

Supplementary Figure 62. ^{1}H NMR Spectrum of **3a**.

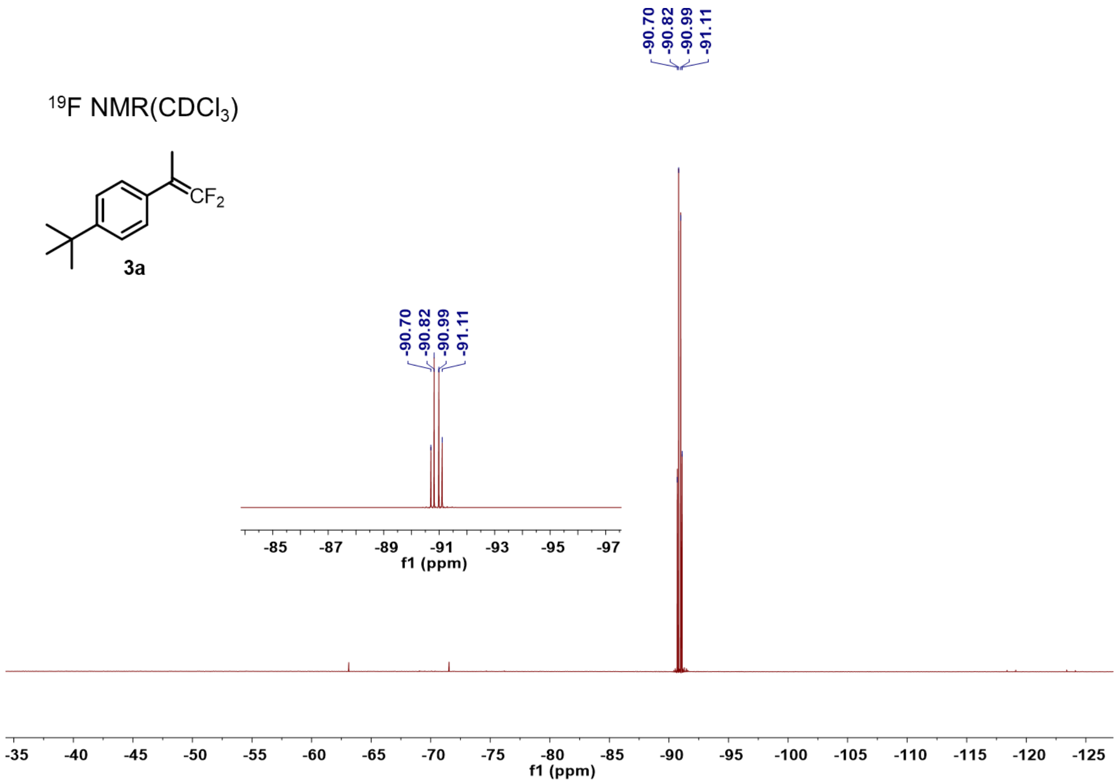

Supplementary Figure 63. ^{19}F NMR Spectrum of **3a**.

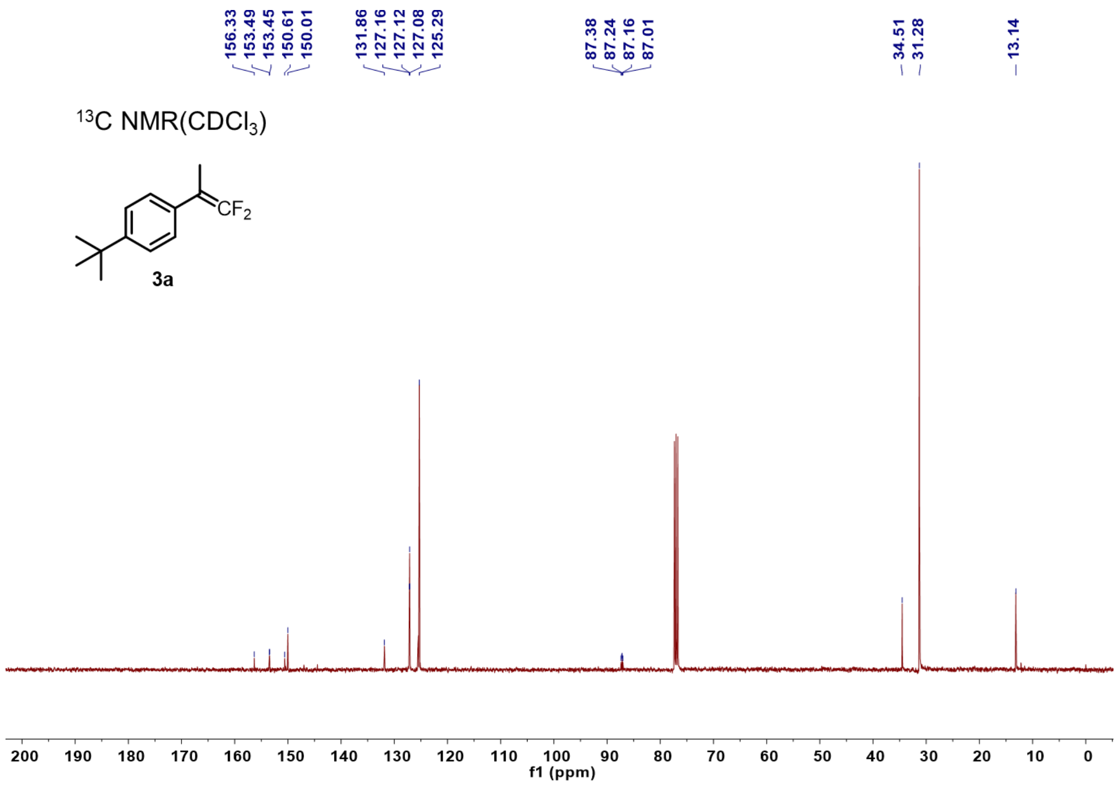

Supplementary Figure 64. ^{13}C NMR Spectrum of **3a**.

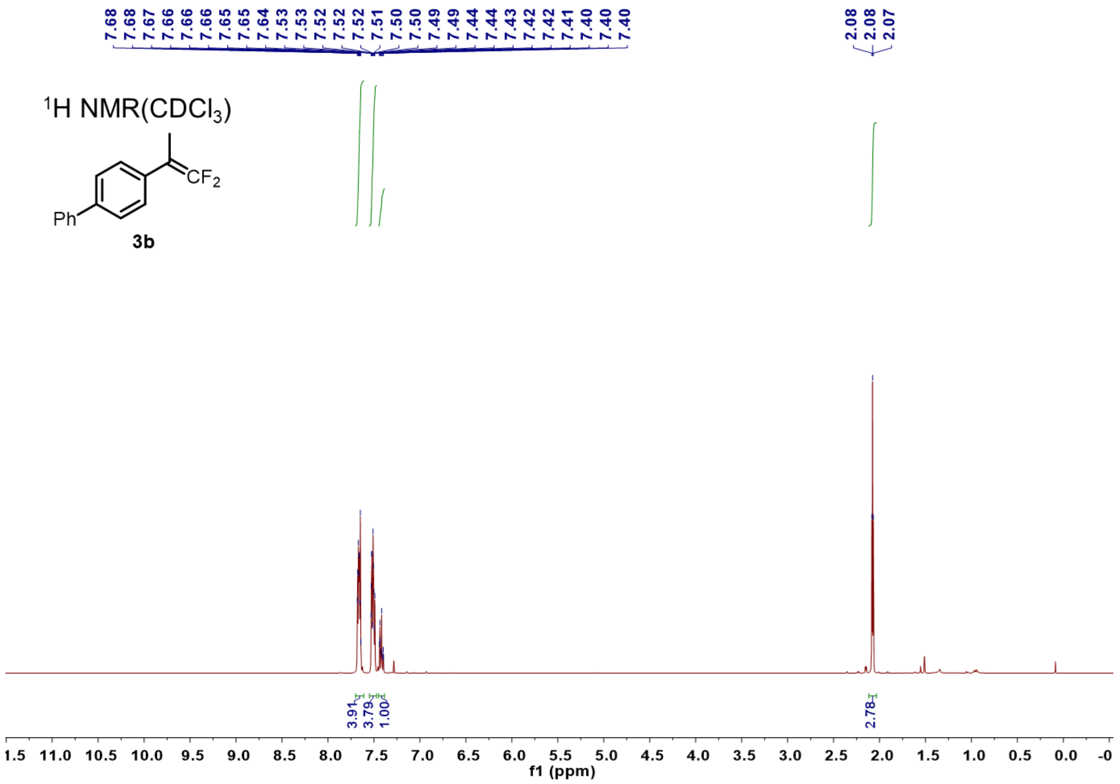

Supplementary Figure 65. ^{1}H NMR Spectrum of **3b**.

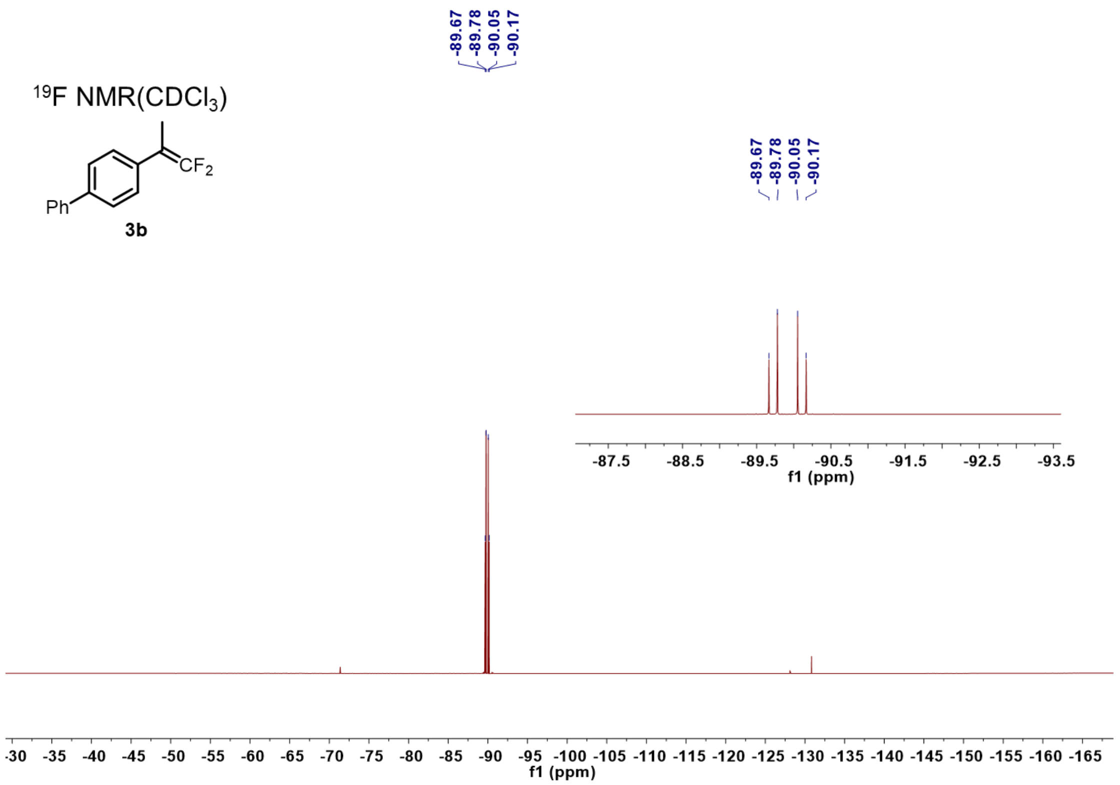

Supplementary Figure 66. ^{19}F NMR Spectrum of **3b**.

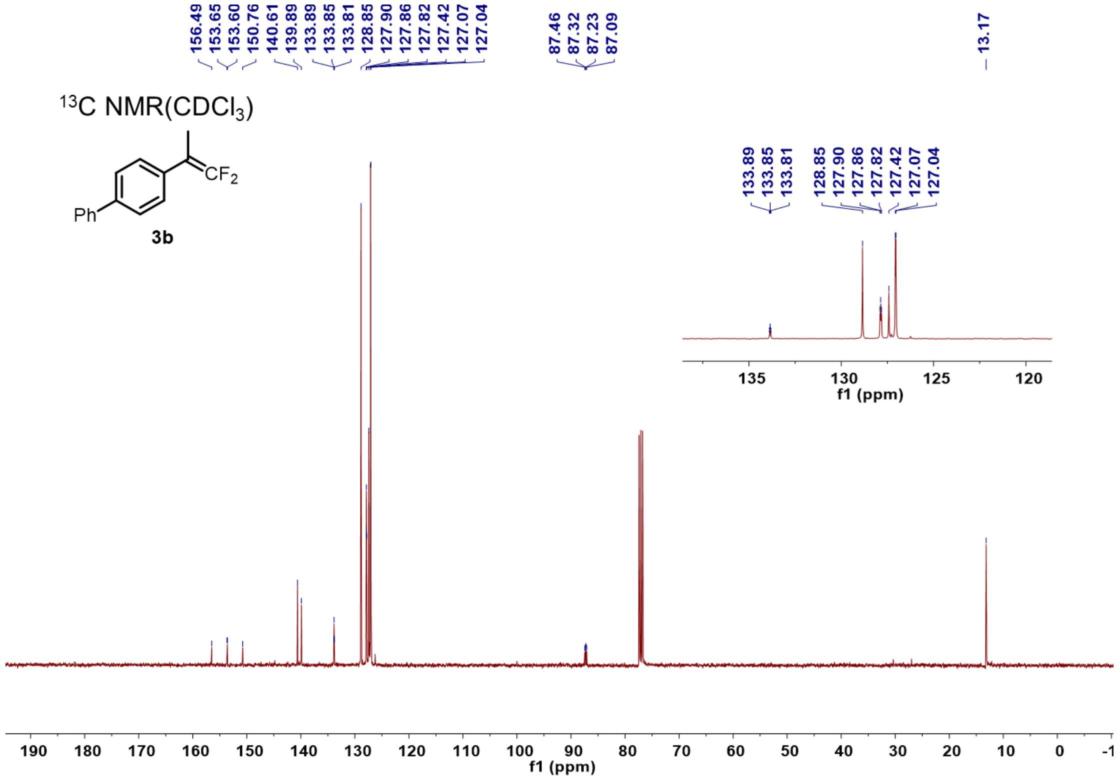

Supplementary Figure 67. ^{13}C NMR Spectrum of **3b**.

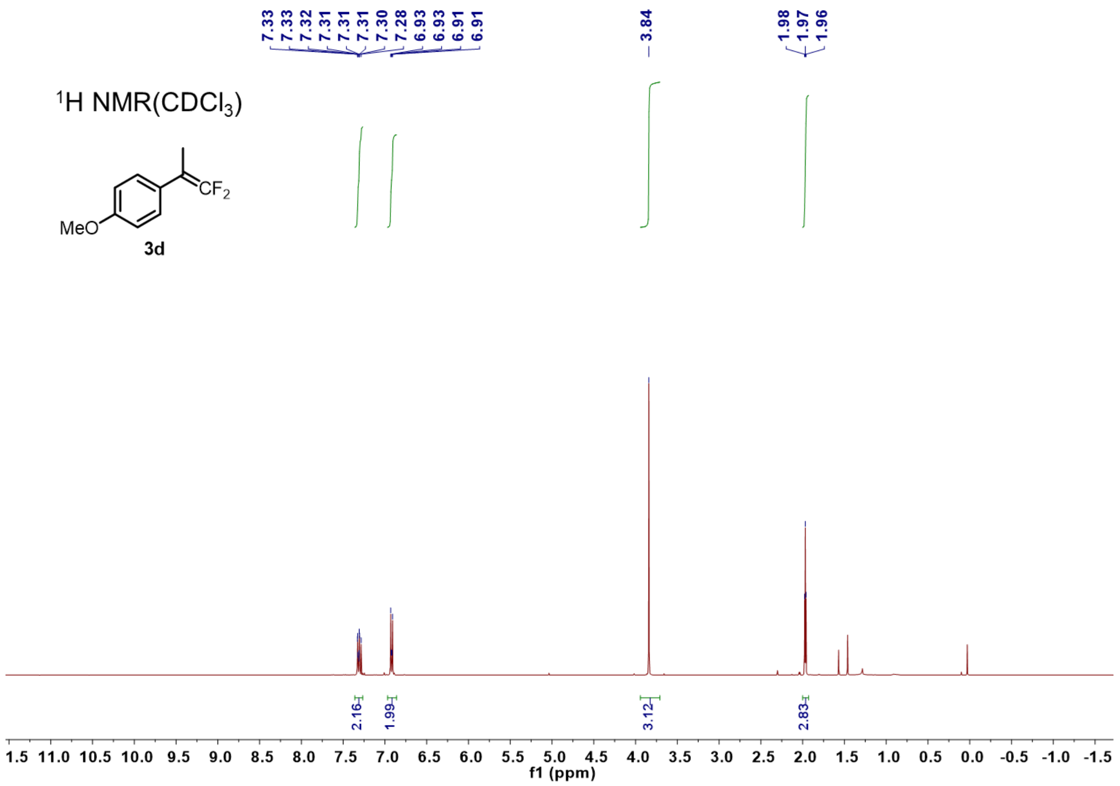

Supplementary Figure 68. ^{1}H NMR Spectrum of **3d**.

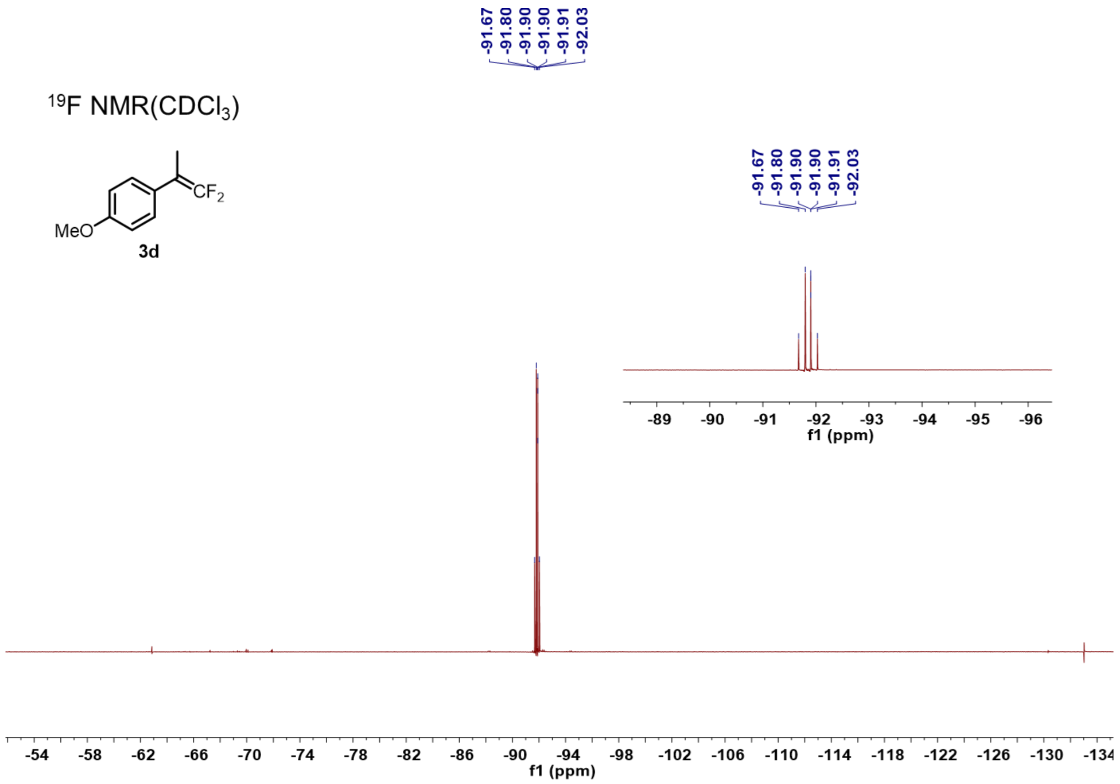

Supplementary Figure 69. ^{19}F NMR Spectrum of **3d**.

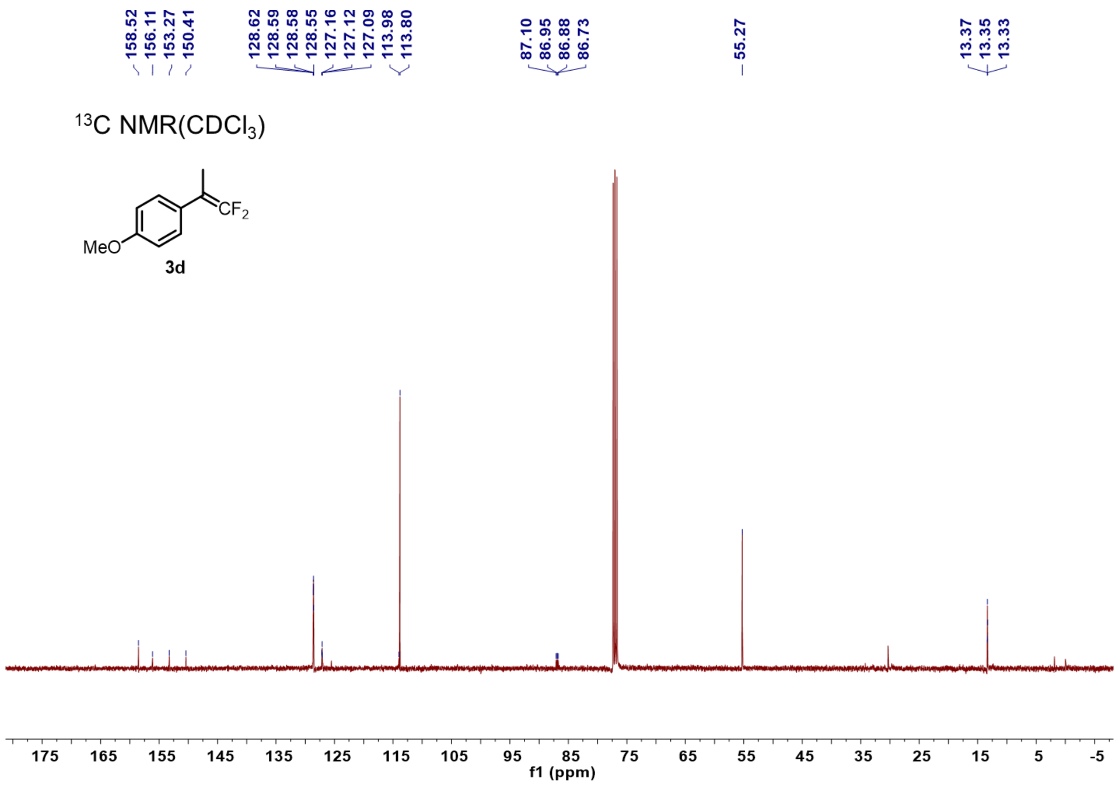

Supplementary Figure 70. ^{13}C NMR Spectrum of **3d**.

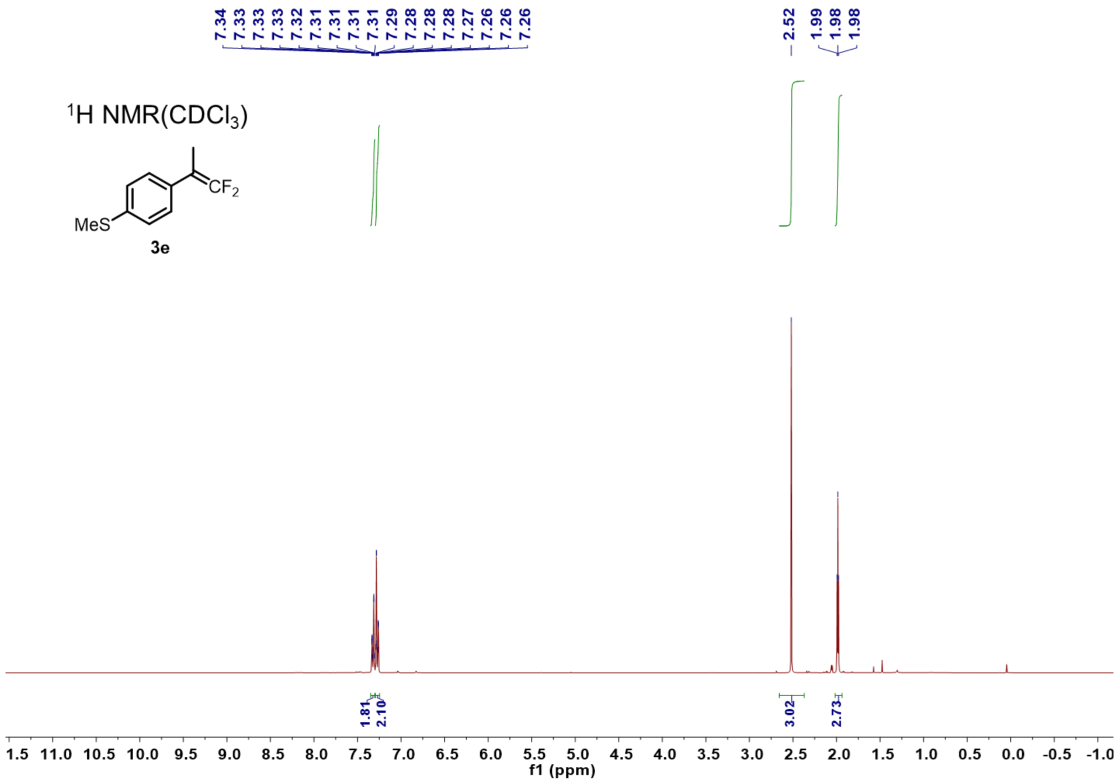

Supplementary Figure 71. ^{1}H NMR Spectrum of **3e**.

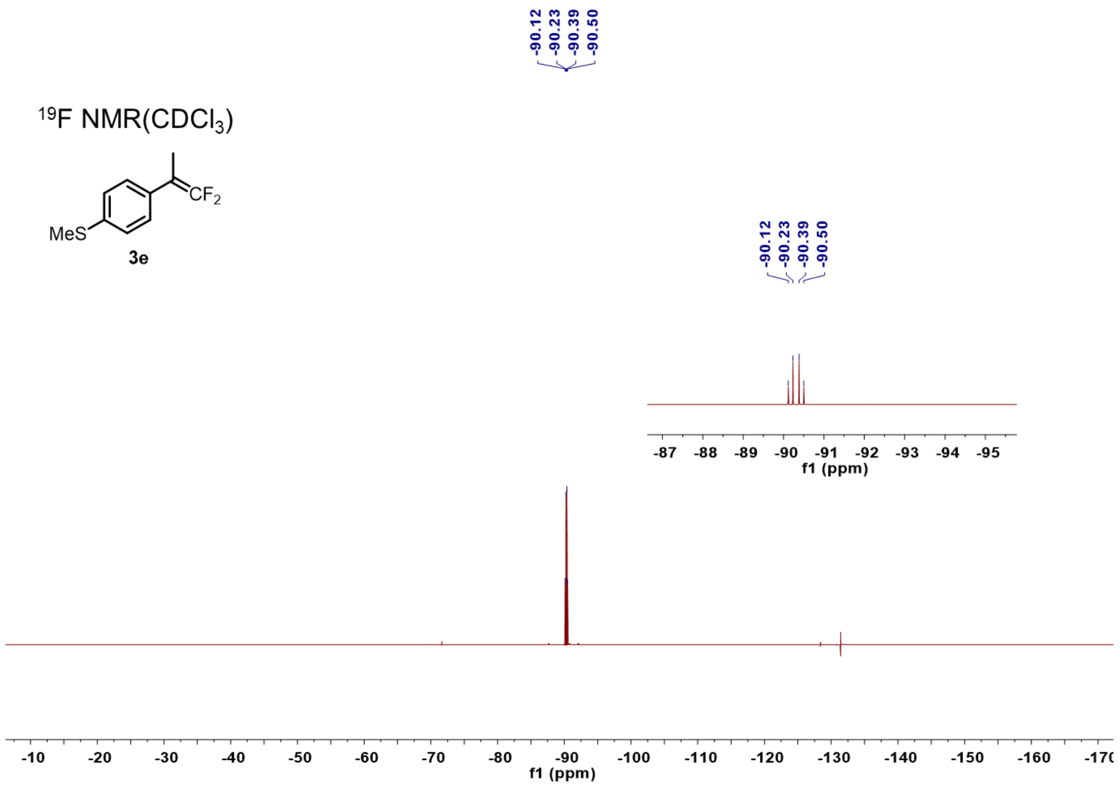

Supplementary Figure 72. ^{19}F NMR Spectrum of **3e**.

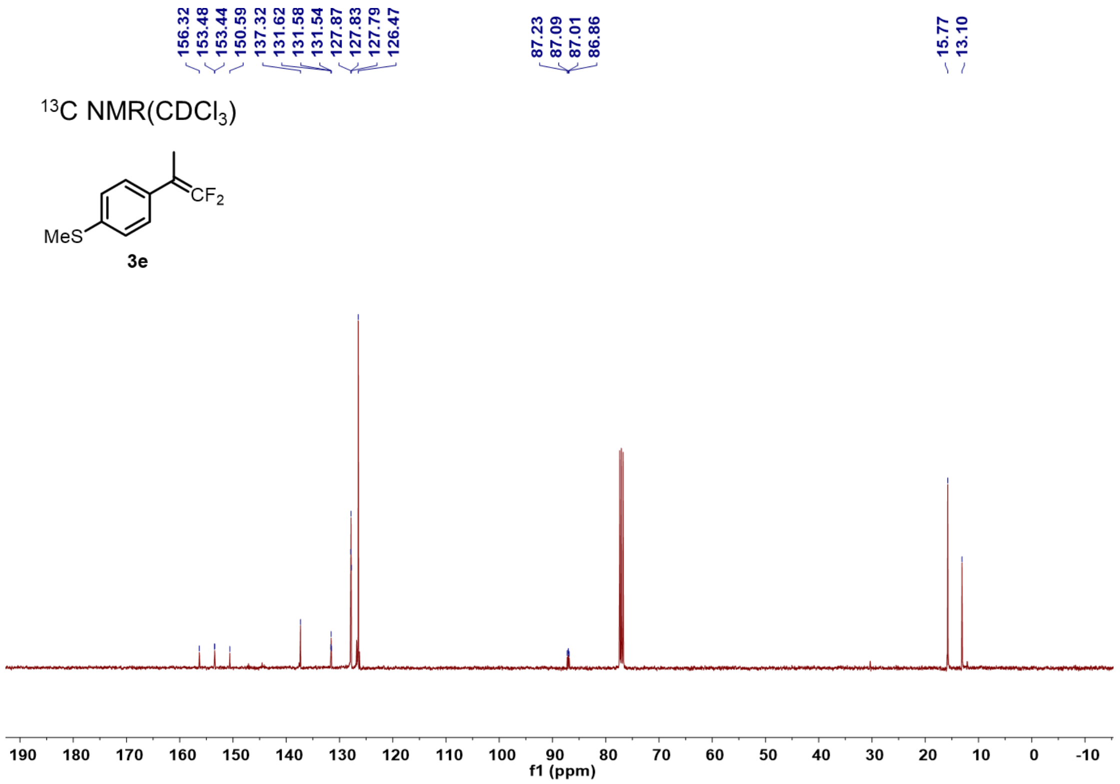

Supplementary Figure 73. ^{13}C NMR Spectrum of **3e**.

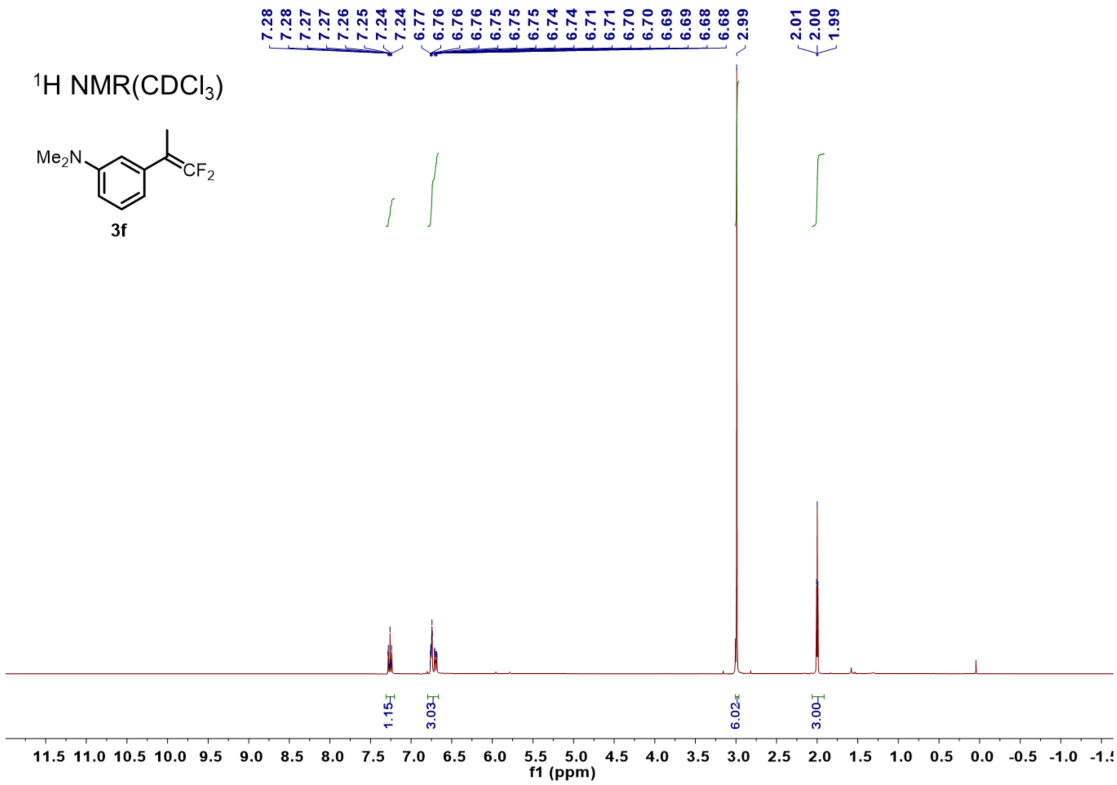

Supplementary Figure 74. ^{1}H NMR Spectrum of **3f**.

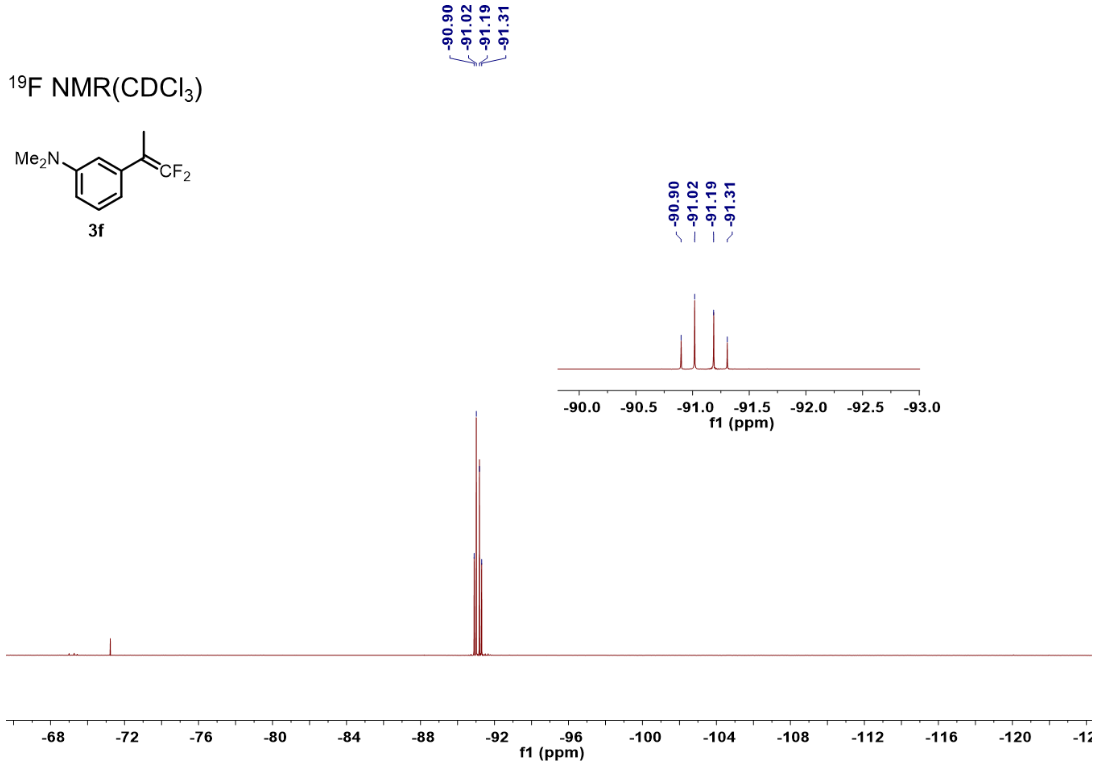

Supplementary Figure 75. ^{19}F NMR Spectrum of **3f**.

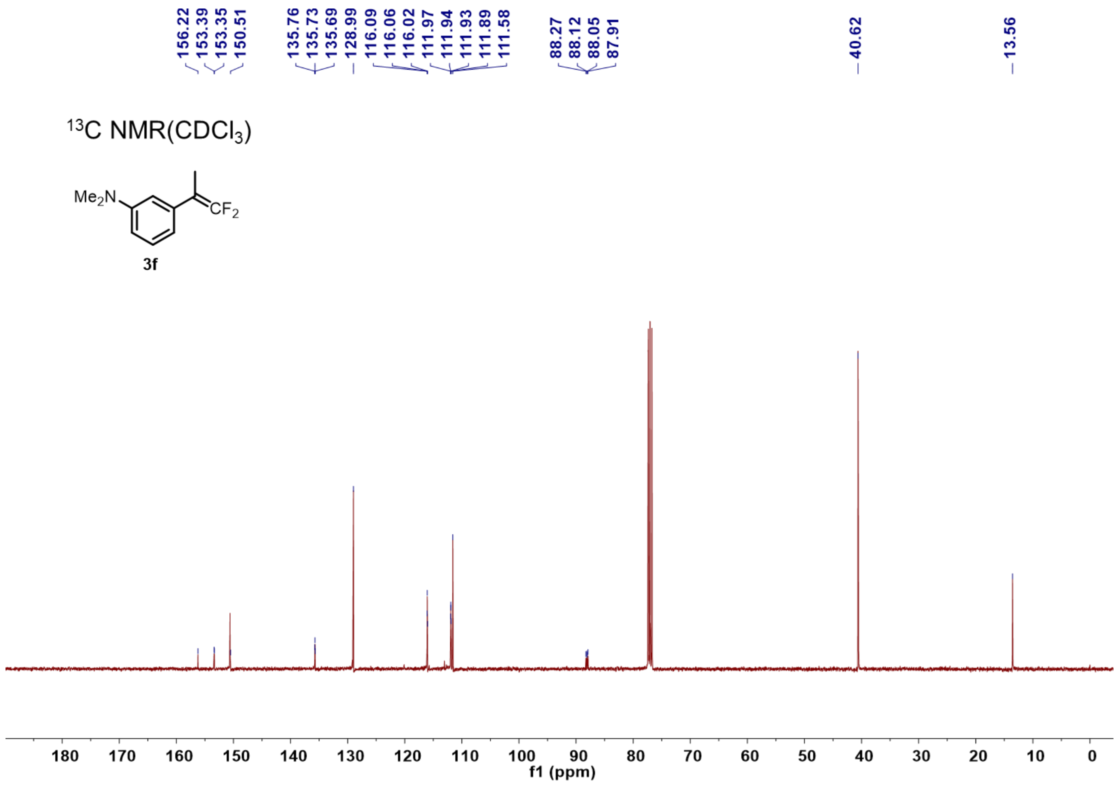

Supplementary Figure 76. ^{13}C NMR Spectrum of **3f**.

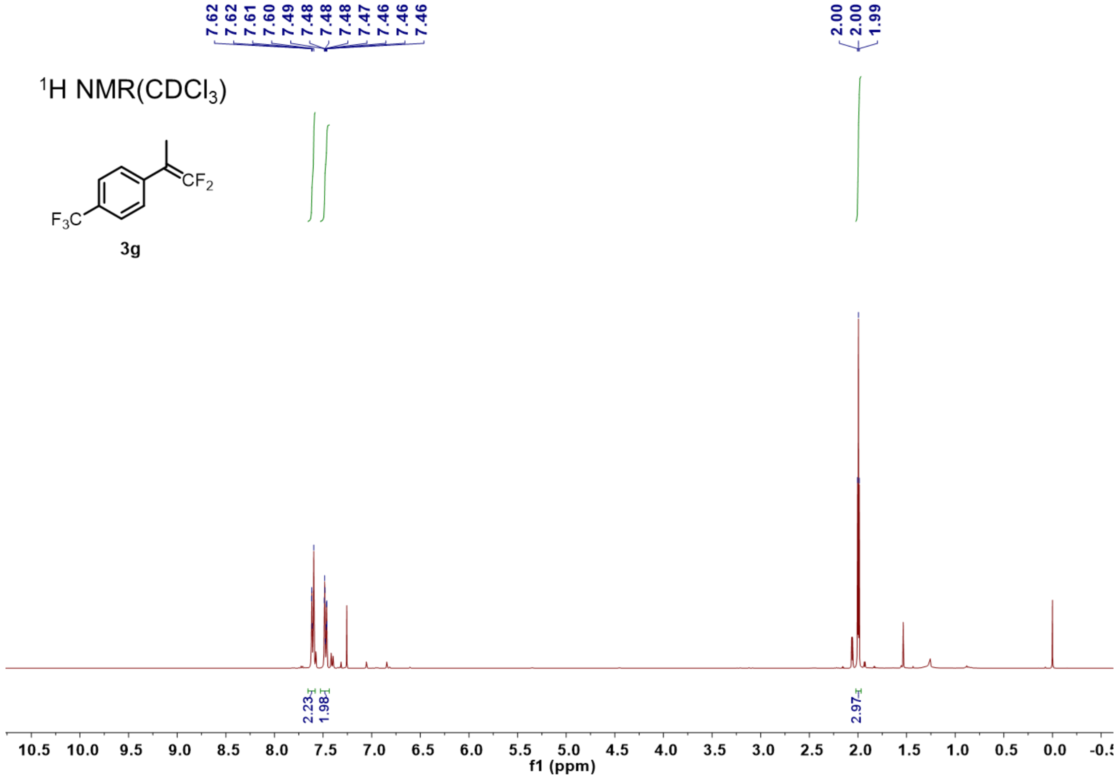

Supplementary Figure 77. ^{1}H NMR Spectrum of **3g**.

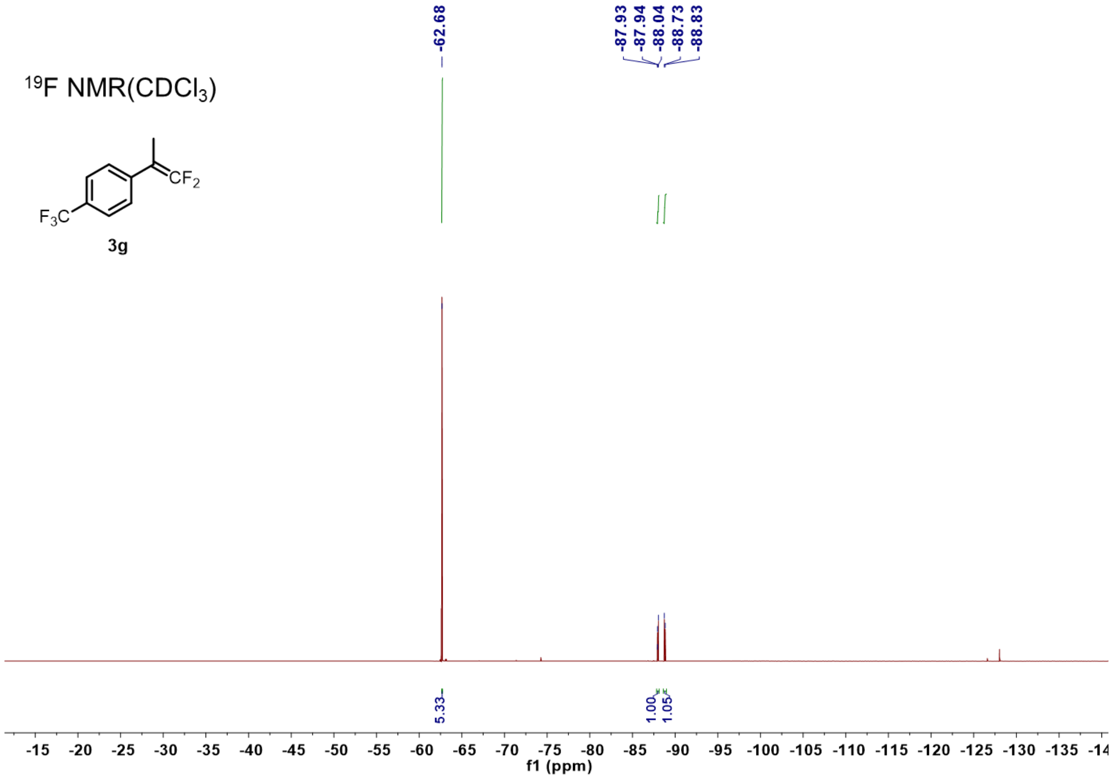

Supplementary Figure 78. ^{19}F NMR Spectrum of **3g**.

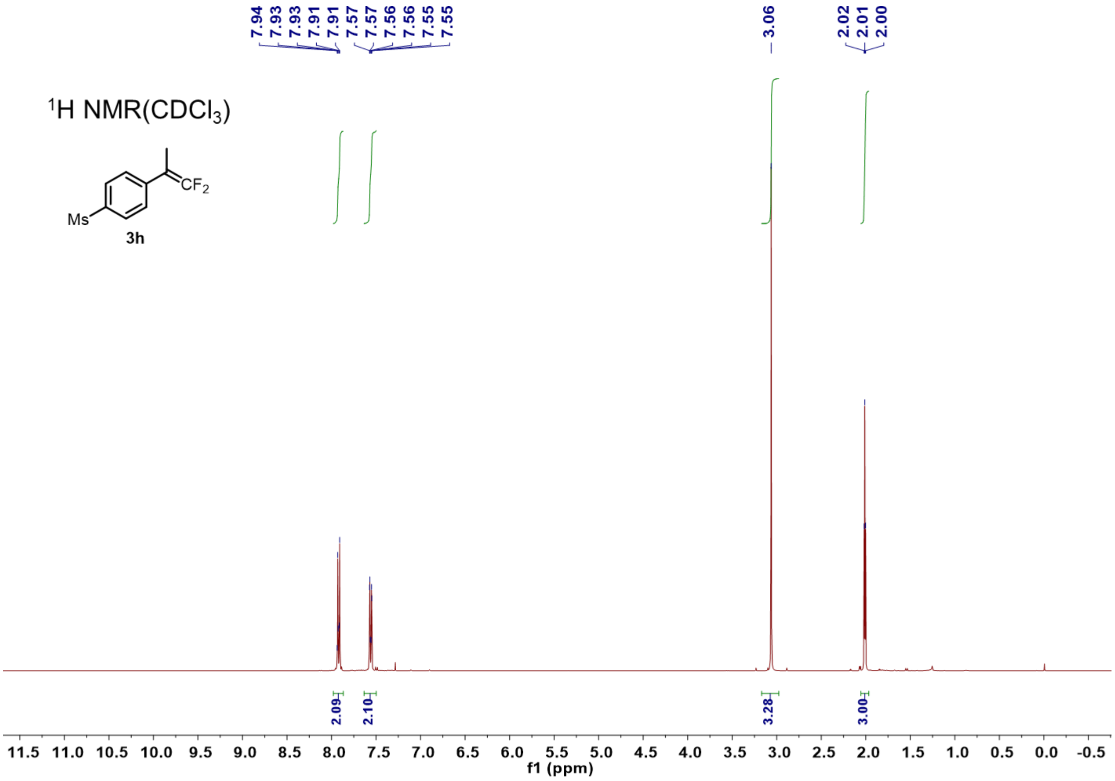

Supplementary Figure 79. ^{1}H NMR Spectrum of **3h**.

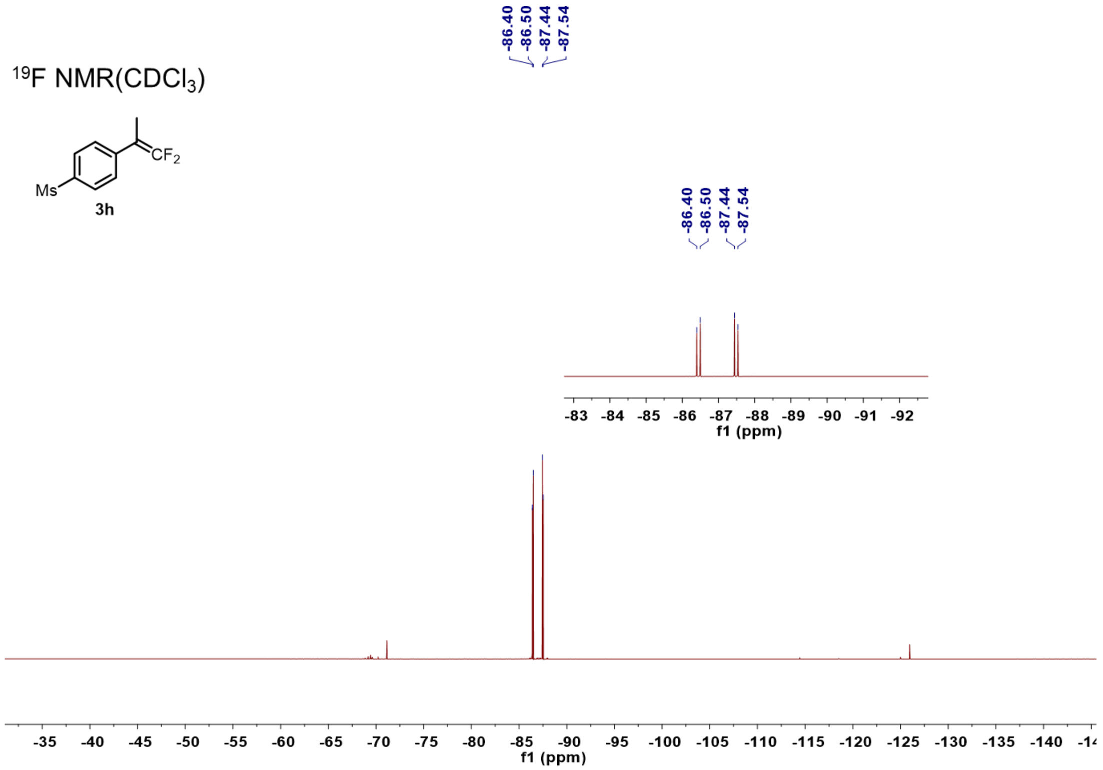

Supplementary Figure 80. ^{19}F NMR Spectrum of **3h**.

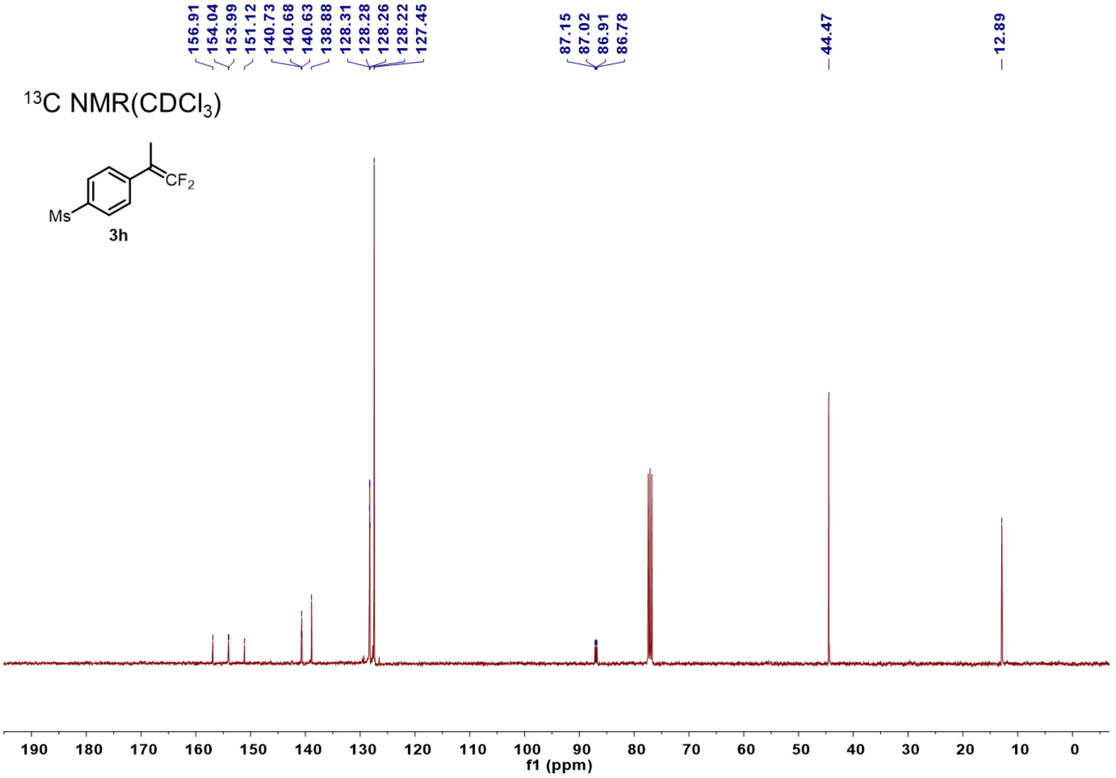

Supplementary Figure 81. ^{13}C NMR Spectrum of **3h**.

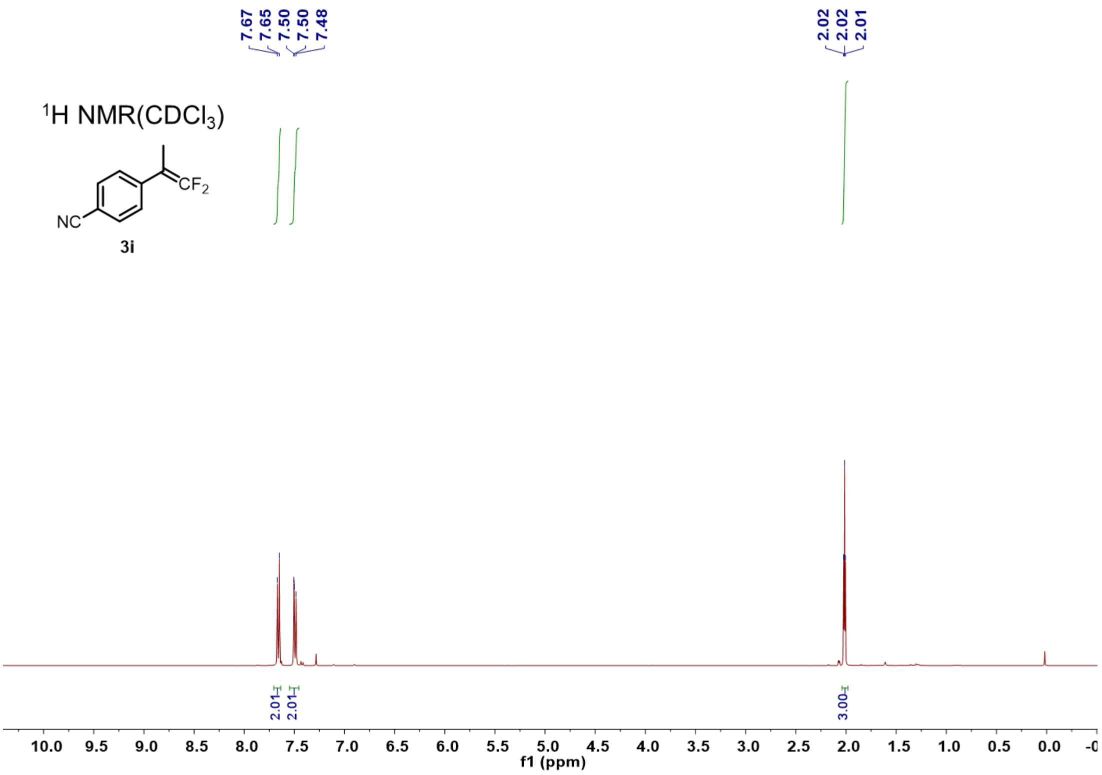

Supplementary Figure 82. ^{1}H NMR Spectrum of **3i**.

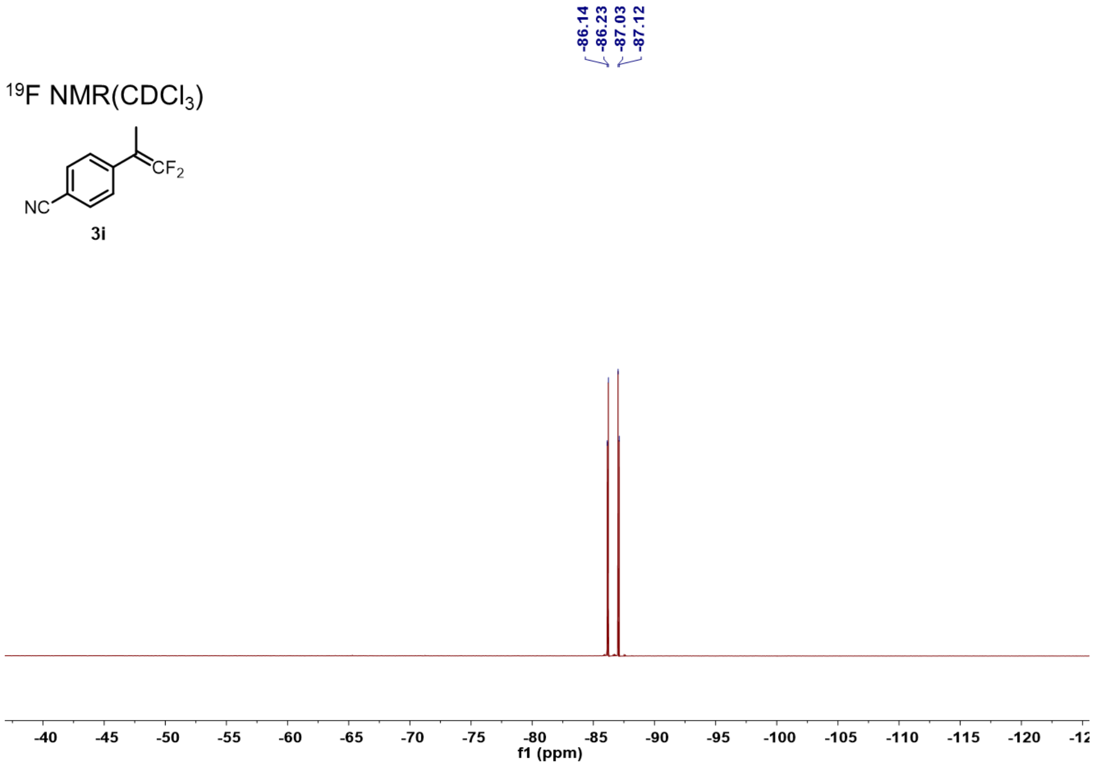

Supplementary Figure 83. ^{19}F NMR Spectrum of **3i**.

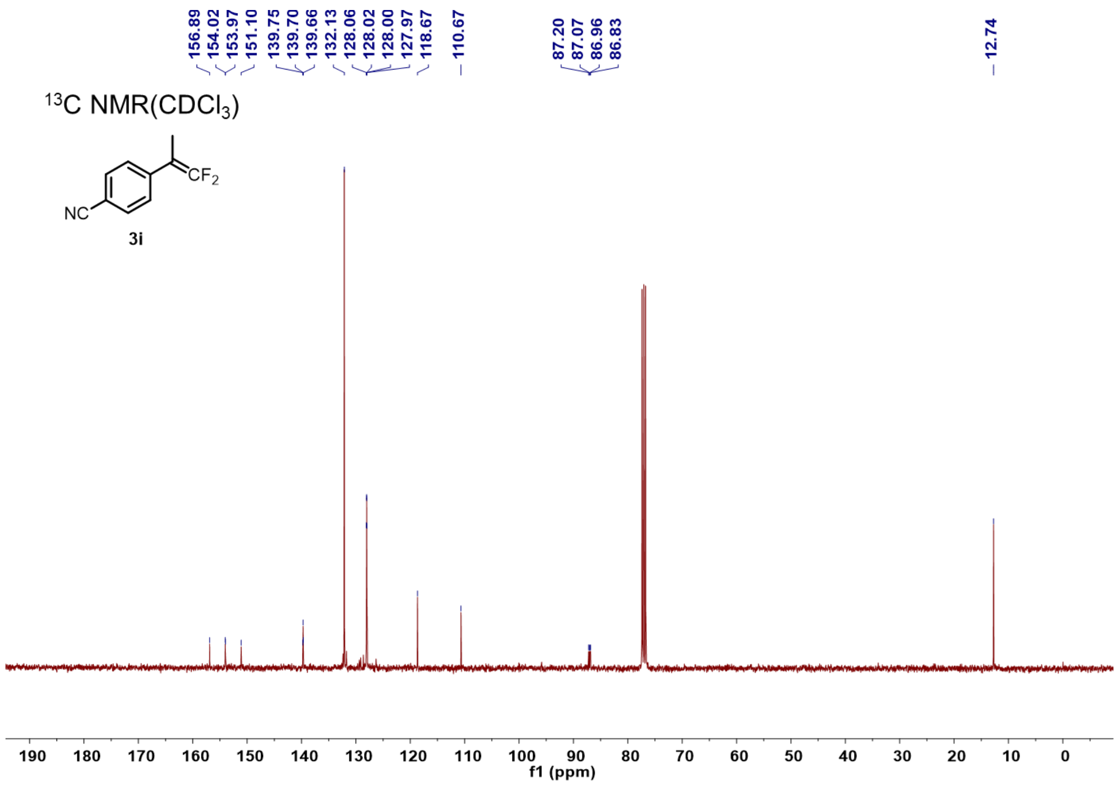

Supplementary Figure 84. ^{13}C NMR Spectrum of **3i**.

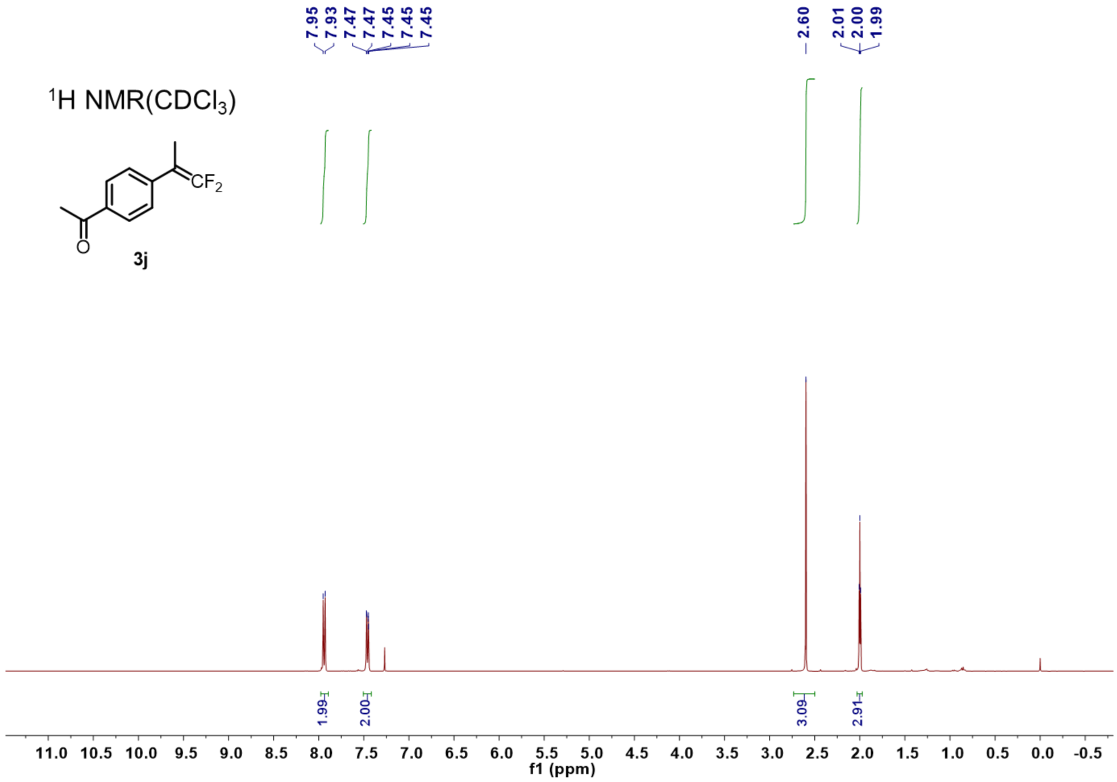

Supplementary Figure 85. ^{1}H NMR Spectrum of **3j**.

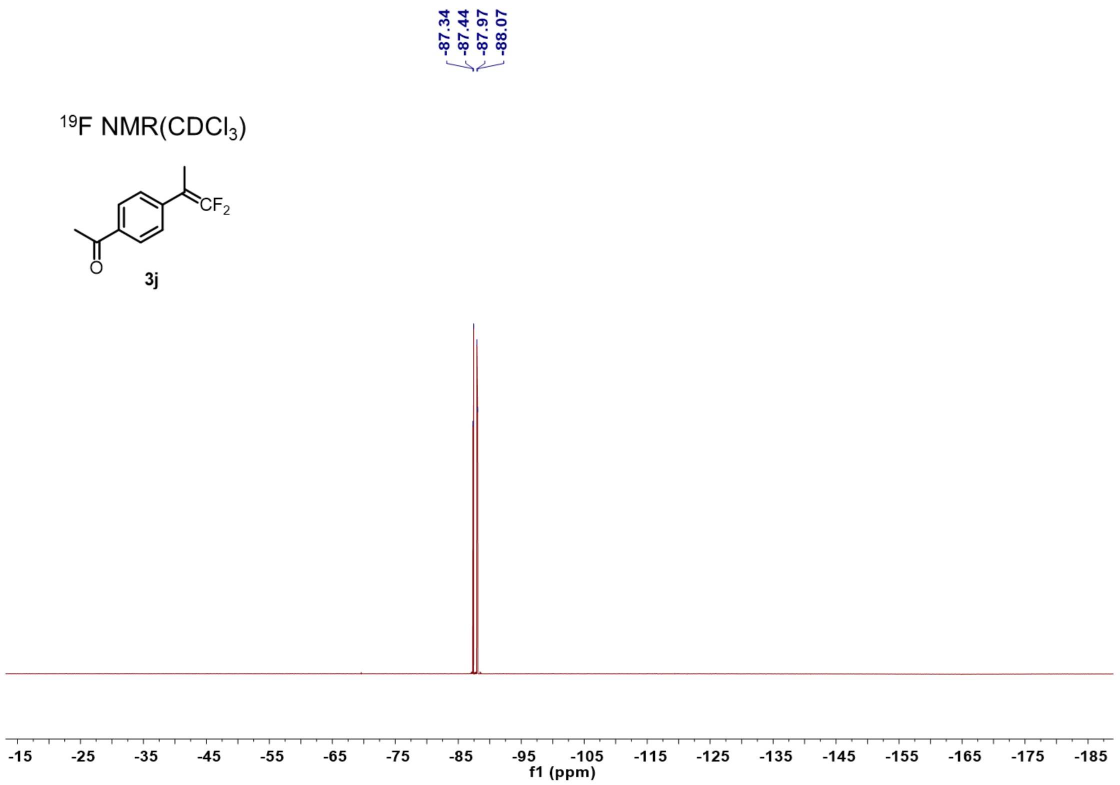

Supplementary Figure 86. ^{19}F NMR Spectrum of **3j**.

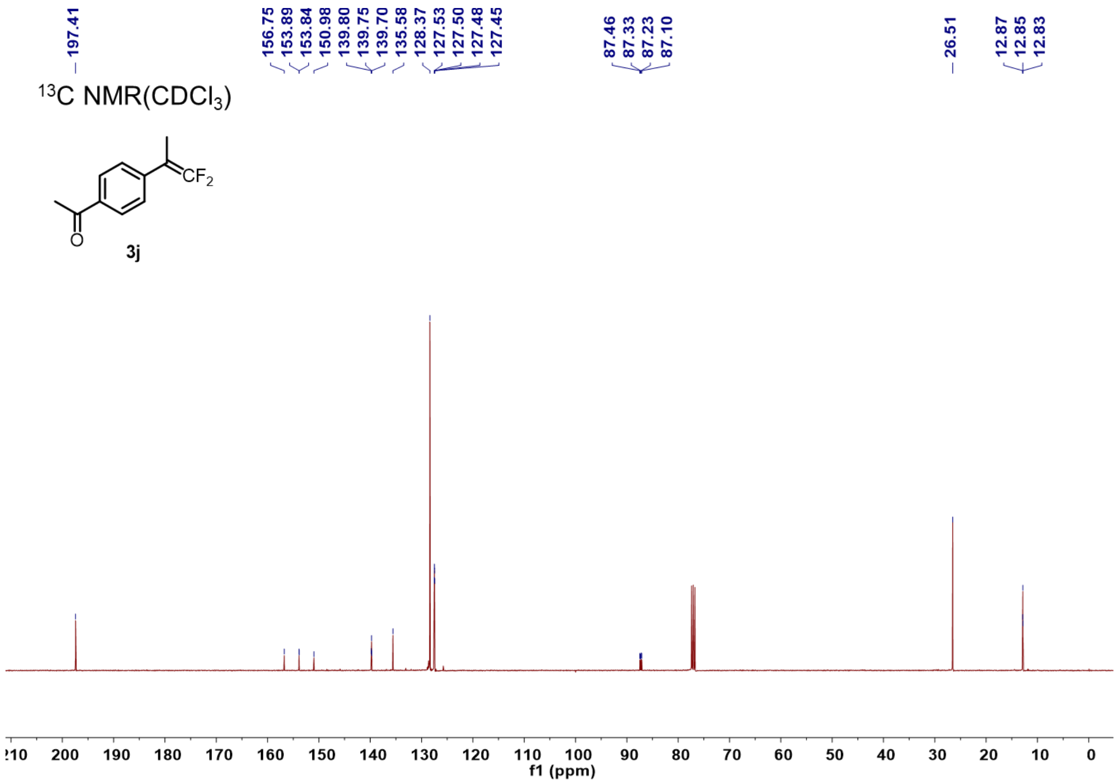

Supplementary Figure 87. ^{13}C NMR Spectrum of **3j**.

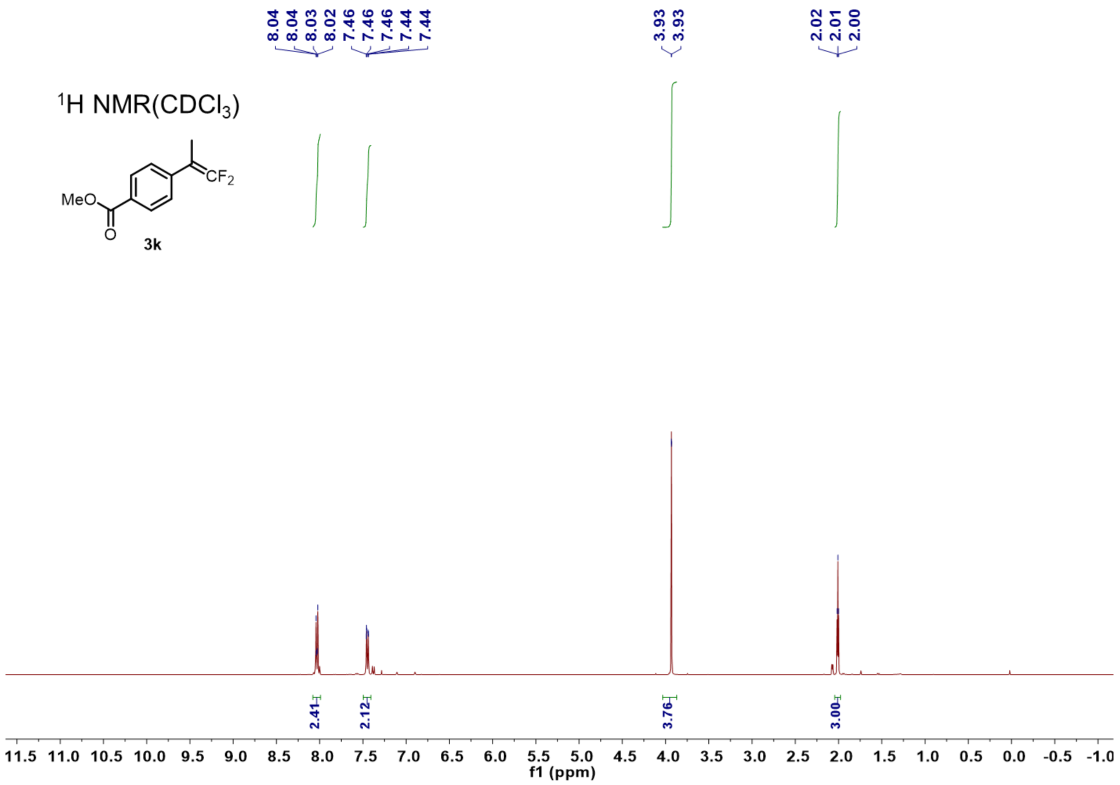

Supplementary Figure 88. ^{1}H NMR Spectrum of **3k**.

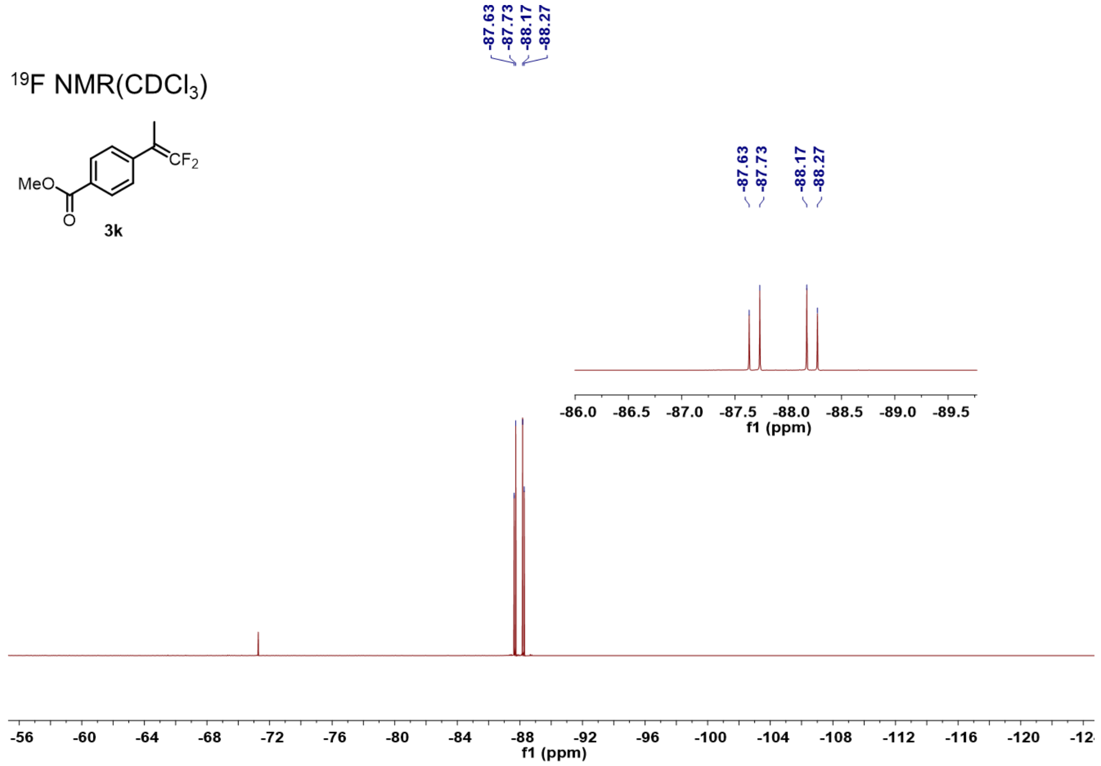

Supplementary Figure 89. ^{19}F NMR Spectrum of **3k**.

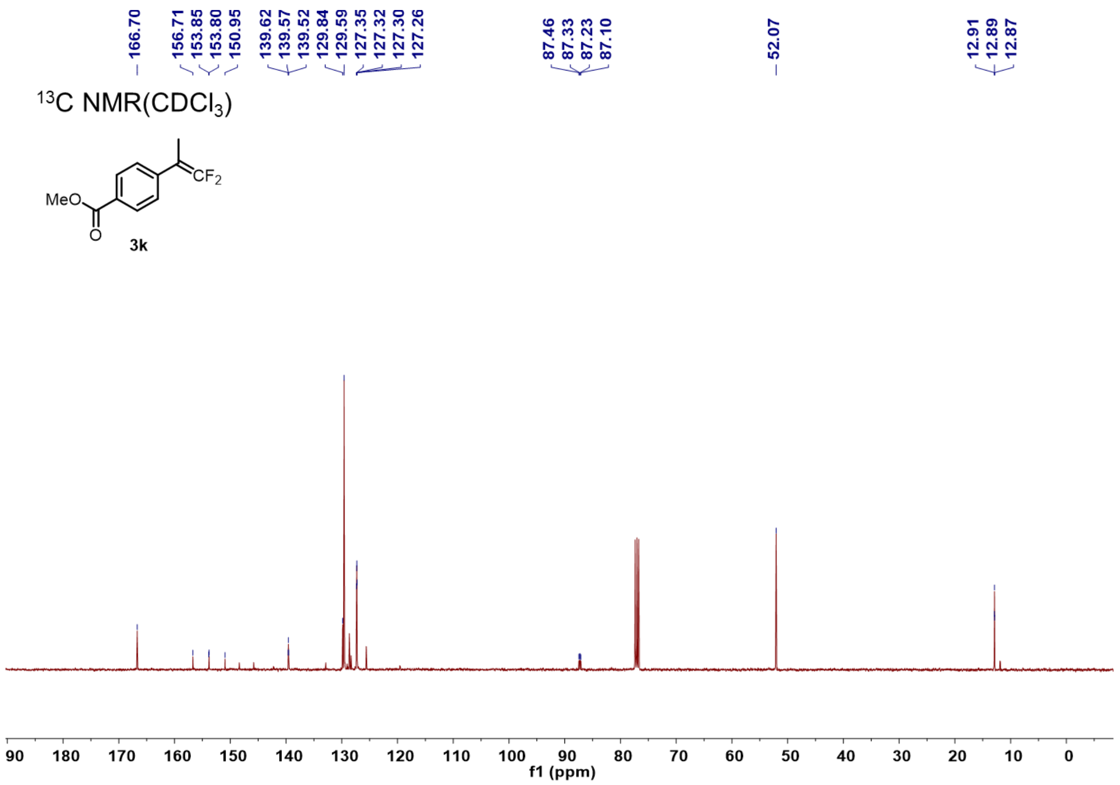

Supplementary Figure 90. ^{13}C NMR Spectrum of **3k**.

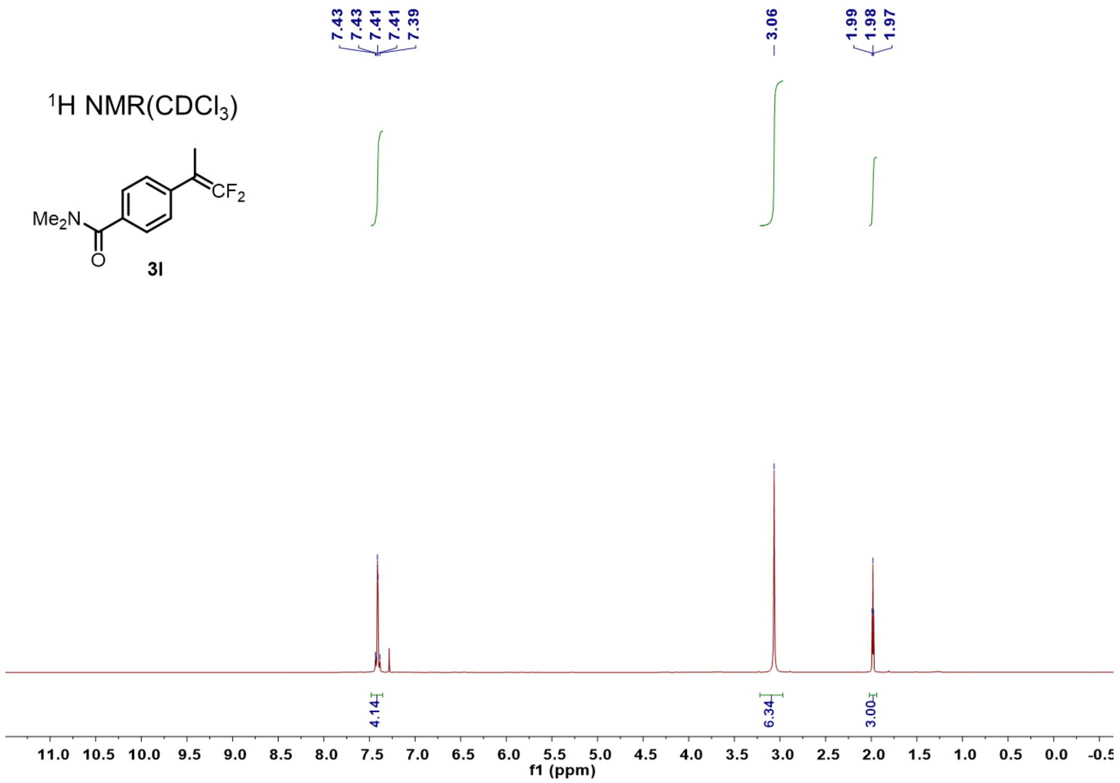

Supplementary Figure 91. ^{1}H NMR Spectrum of **3l**.

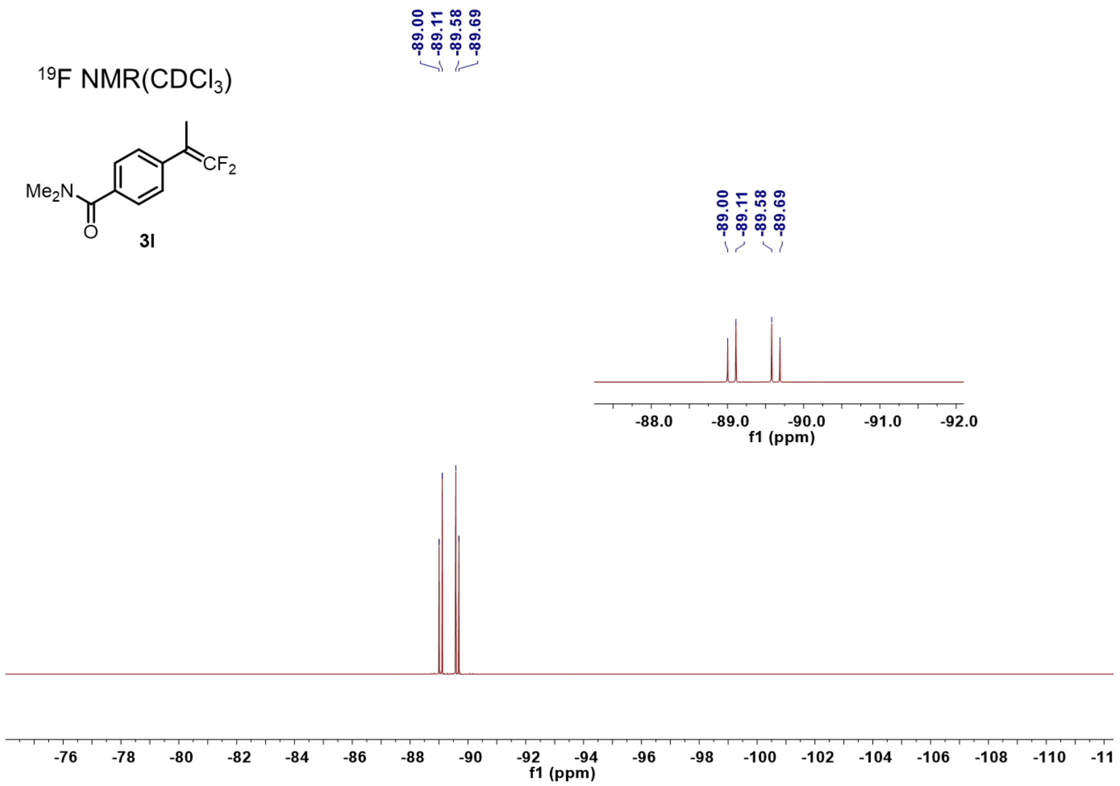

Supplementary Figure 92. ^{19}F NMR Spectrum of **3l**.

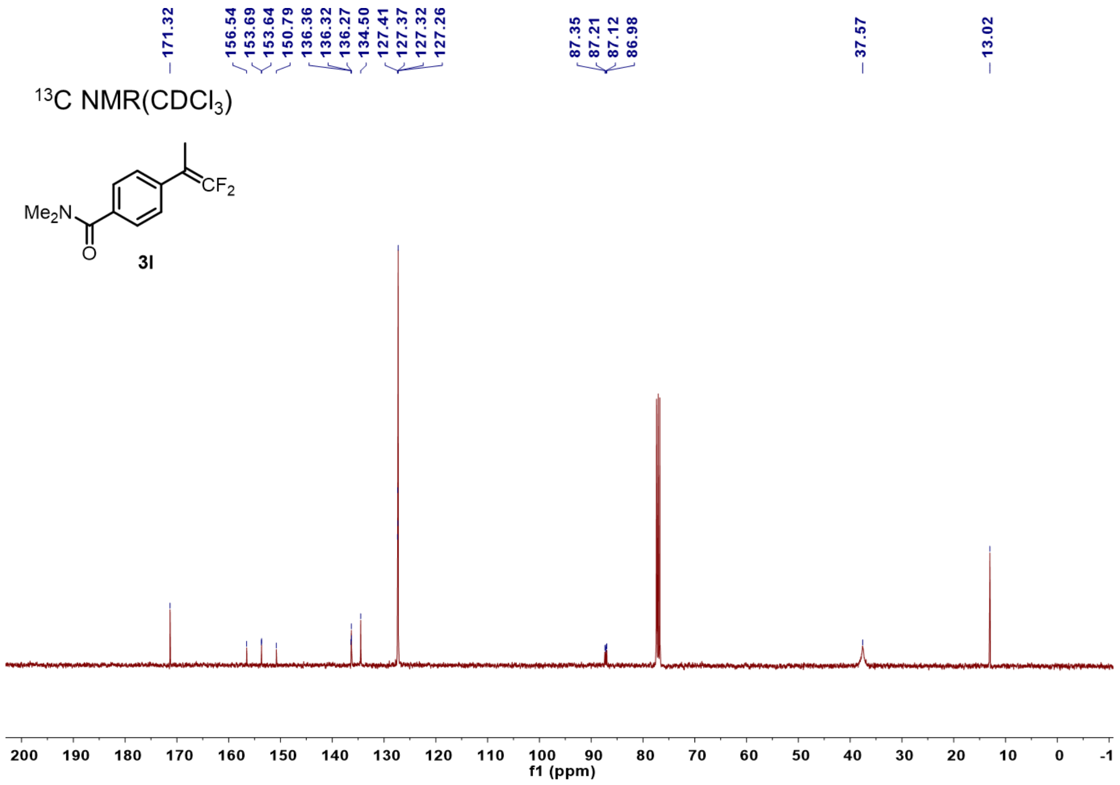

Supplementary Figure 93. ^{13}C NMR Spectrum of **3l**.

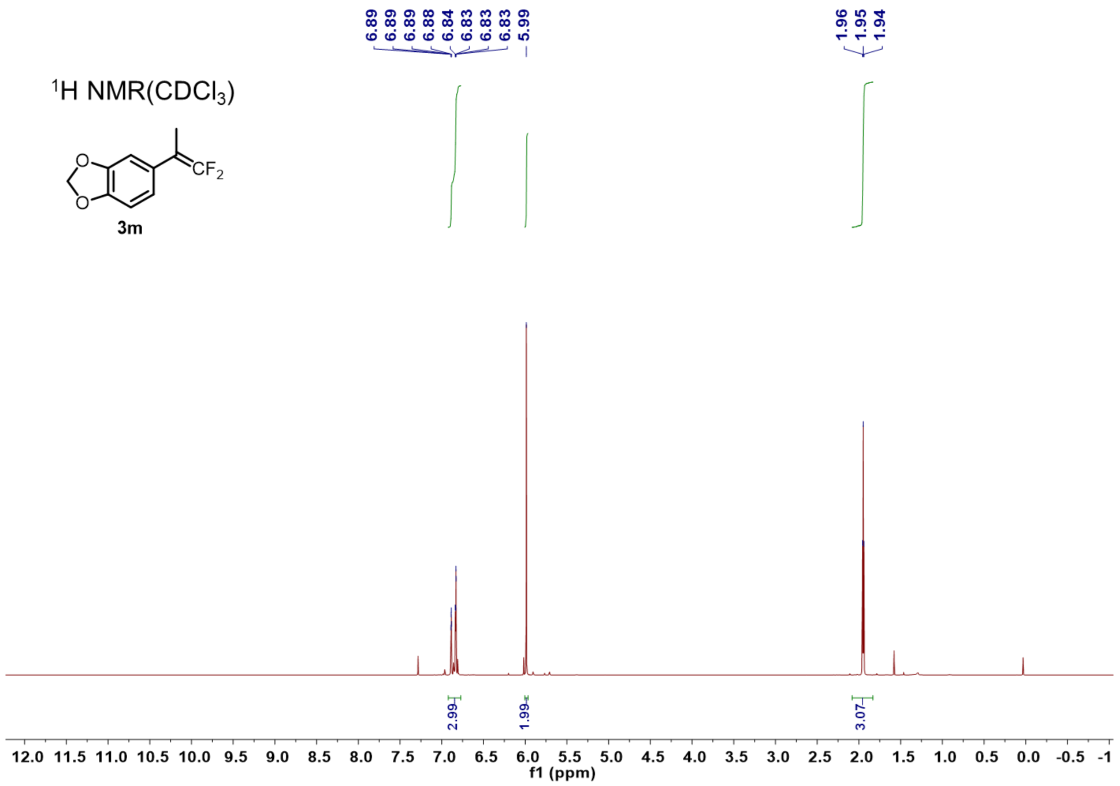

Supplementary Figure 94. ^{1}H NMR Spectrum of **3m**.

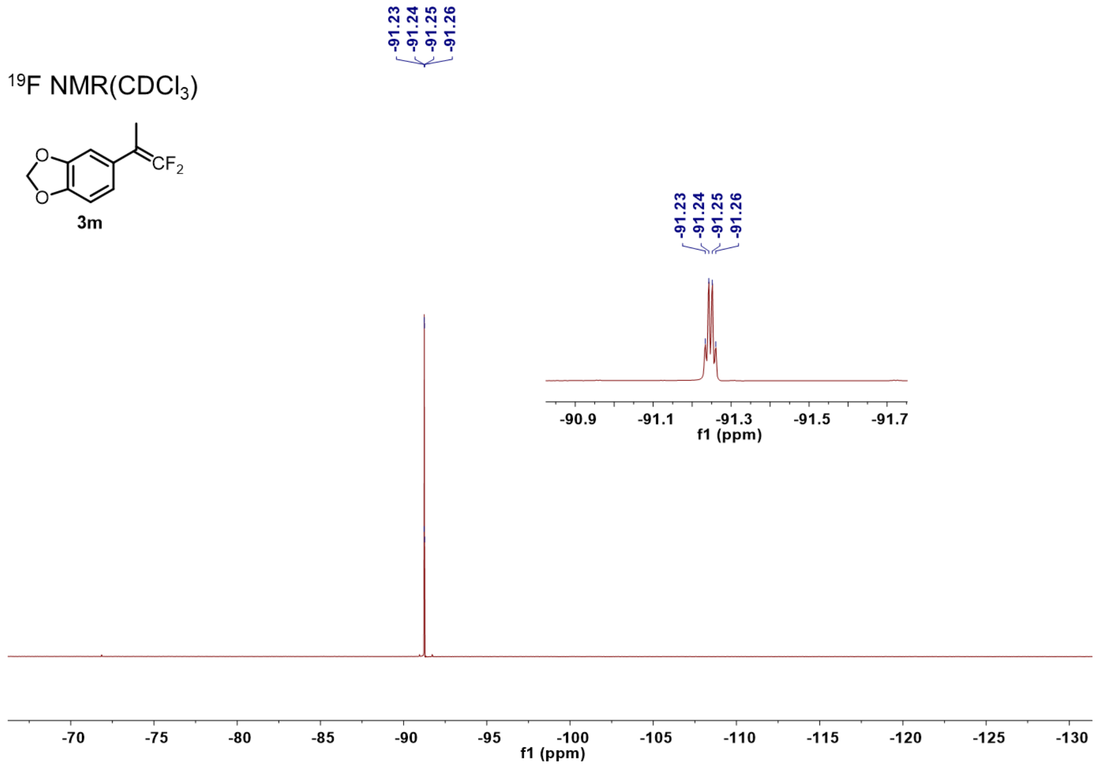

Supplementary Figure 95. ^{19}F NMR Spectrum of **3m**.

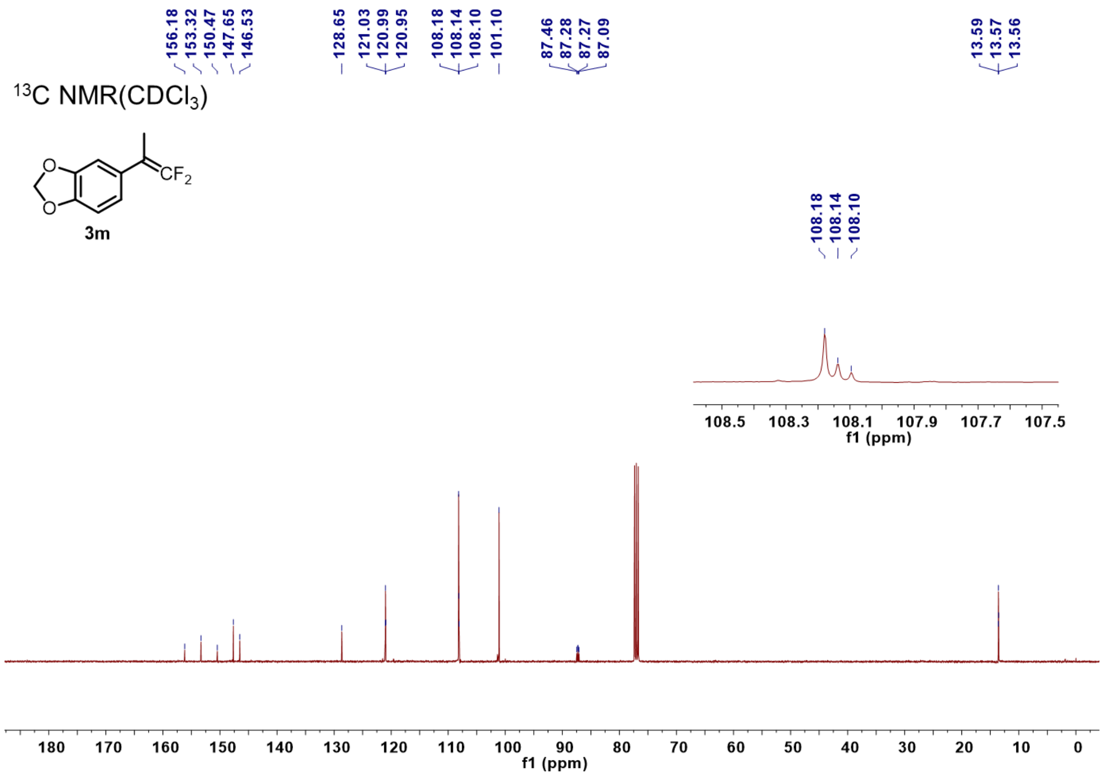

Supplementary Figure 96. ^{13}C NMR Spectrum of **3m**.

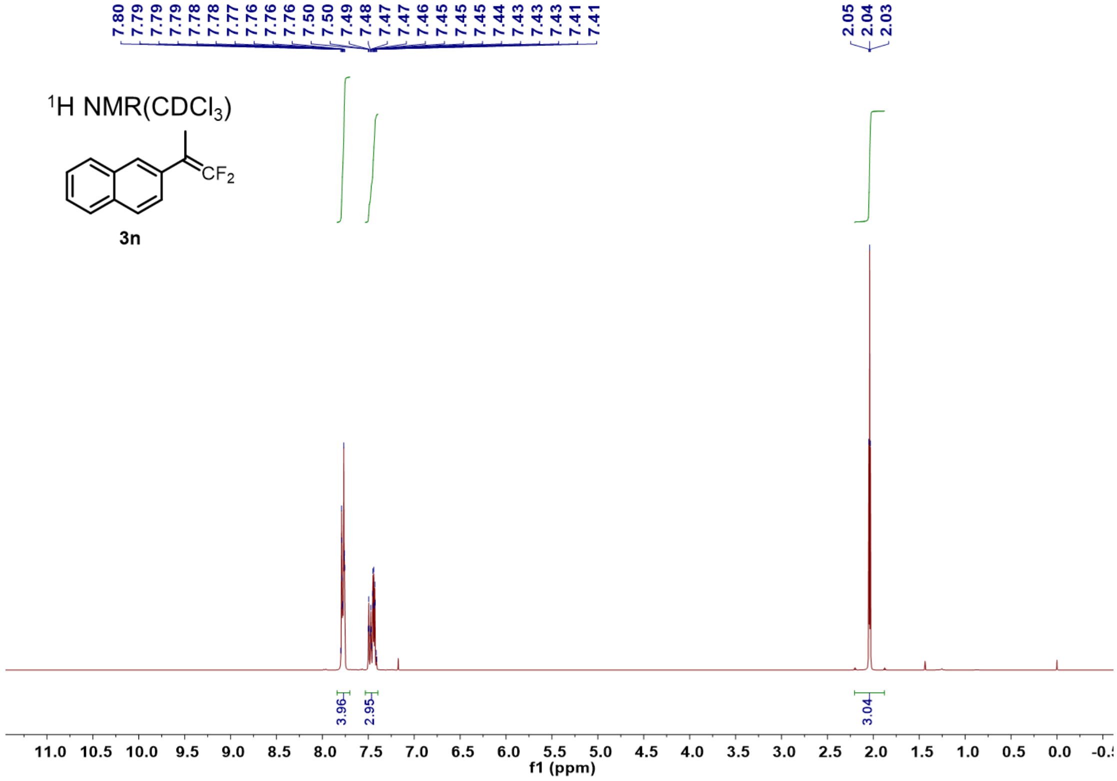

Supplementary Figure 97. ^{1}H NMR Spectrum of **3n**.

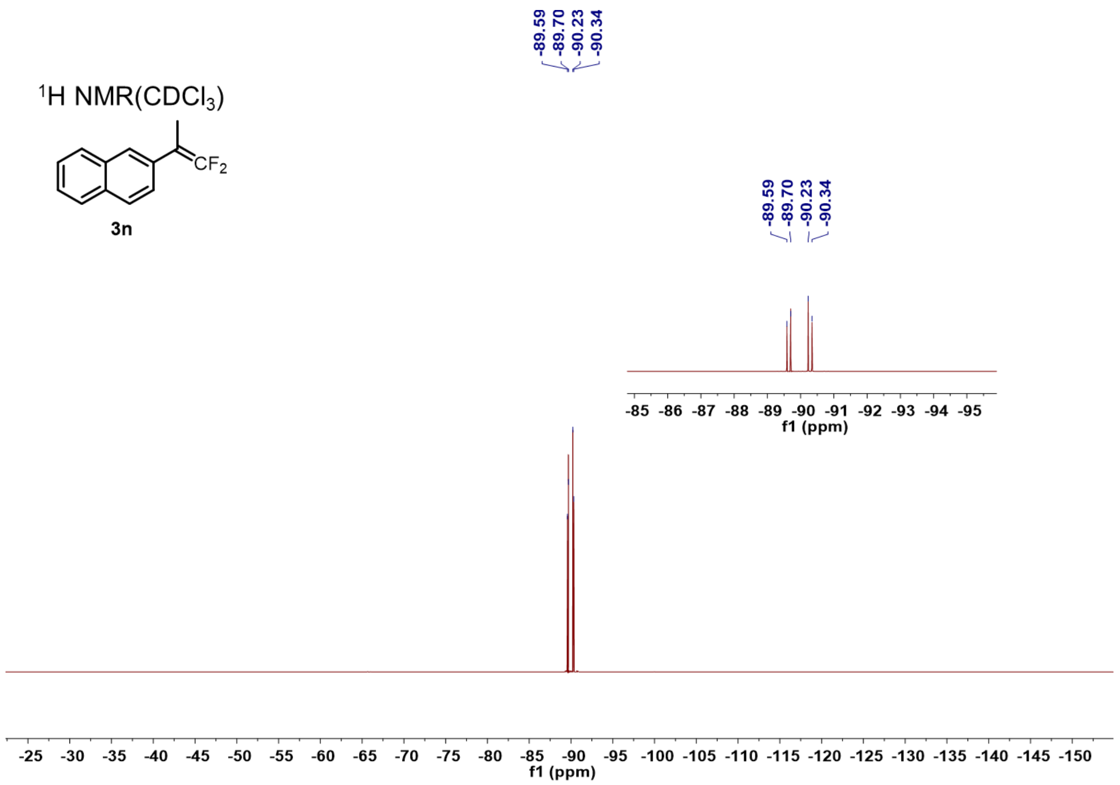

Supplementary Figure 98. ^{19}F NMR Spectrum of **3n**.

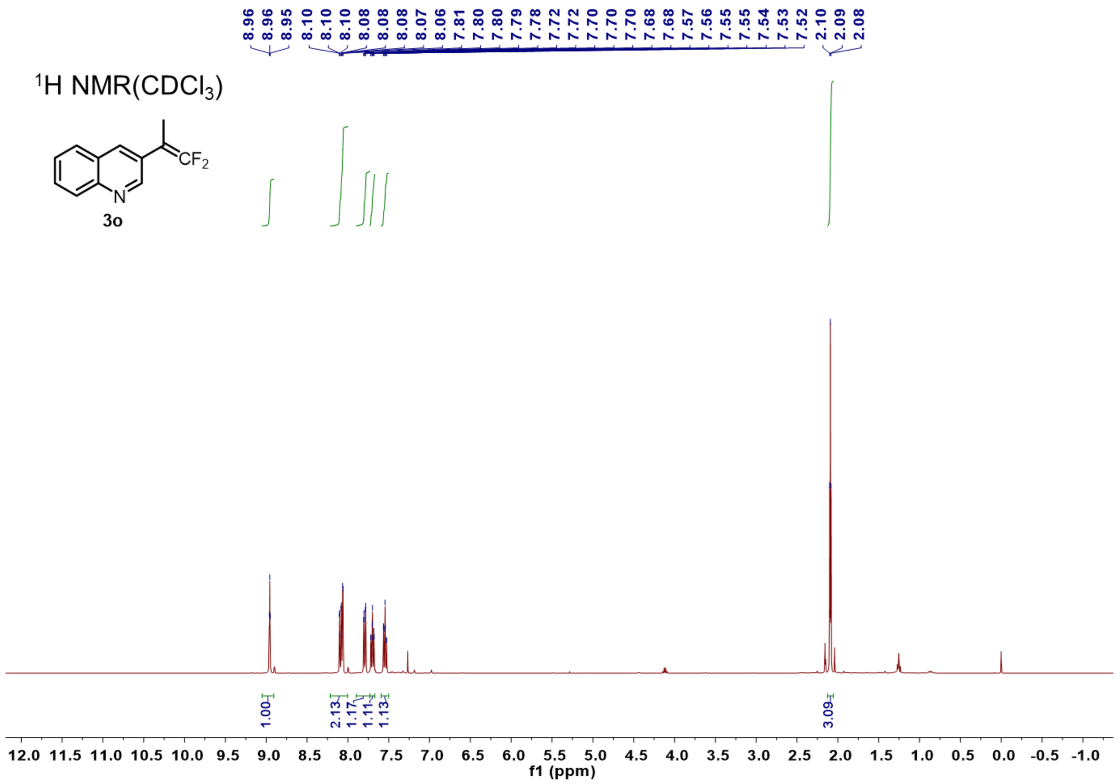

Supplementary Figure 99. ^{1}H NMR Spectrum of **3o**.

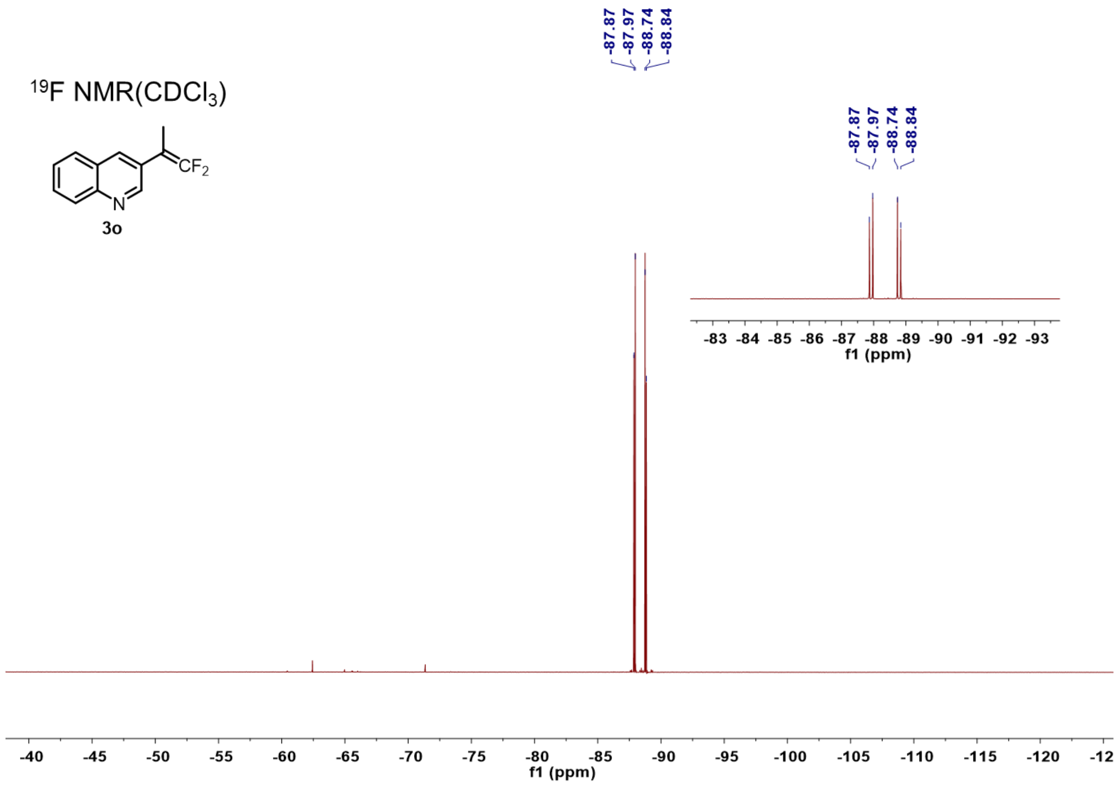

Supplementary Figure 100. ^{19}F NMR Spectrum of **3o**.

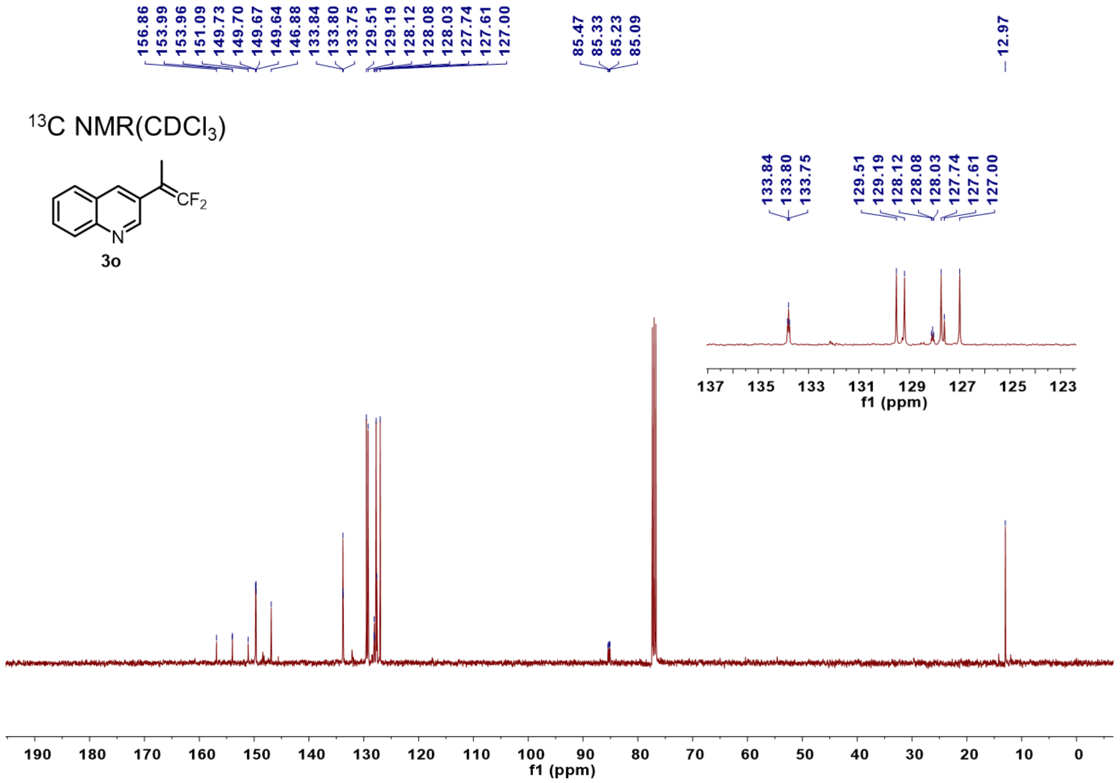

Supplementary Figure 101. ^{13}C NMR Spectrum of **3o**.

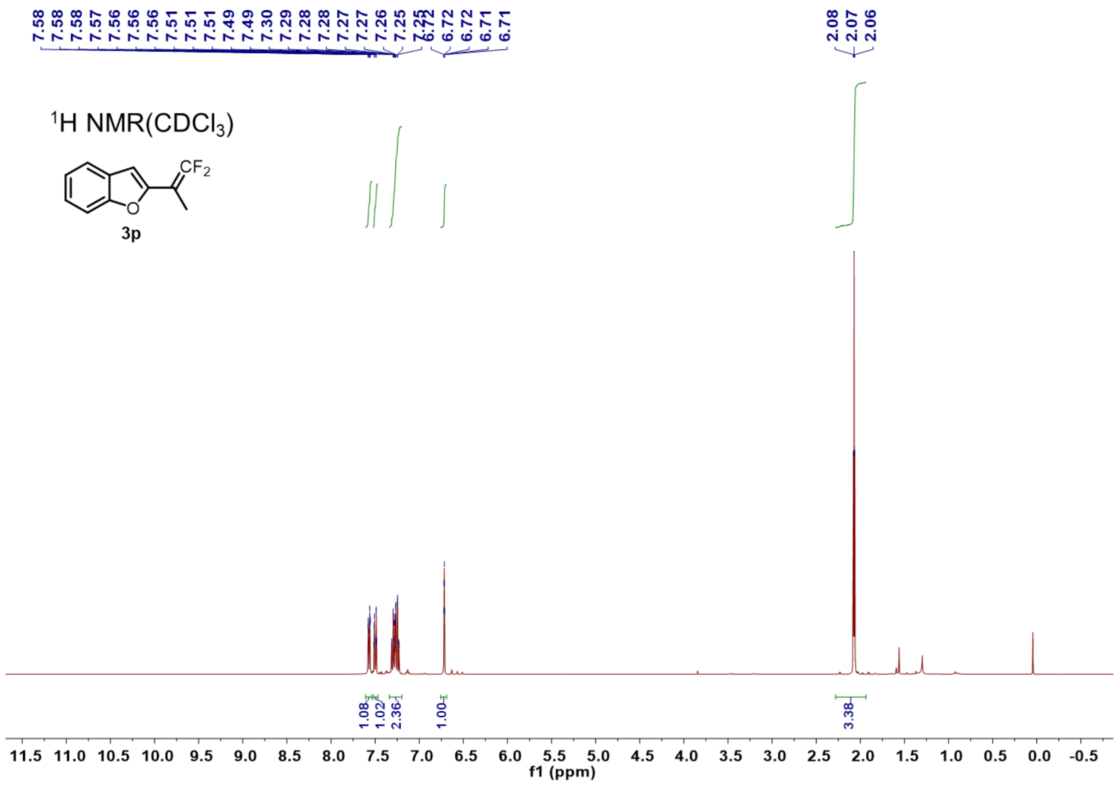

Supplementary Figure 102. ^{1}H NMR Spectrum of **3p**.

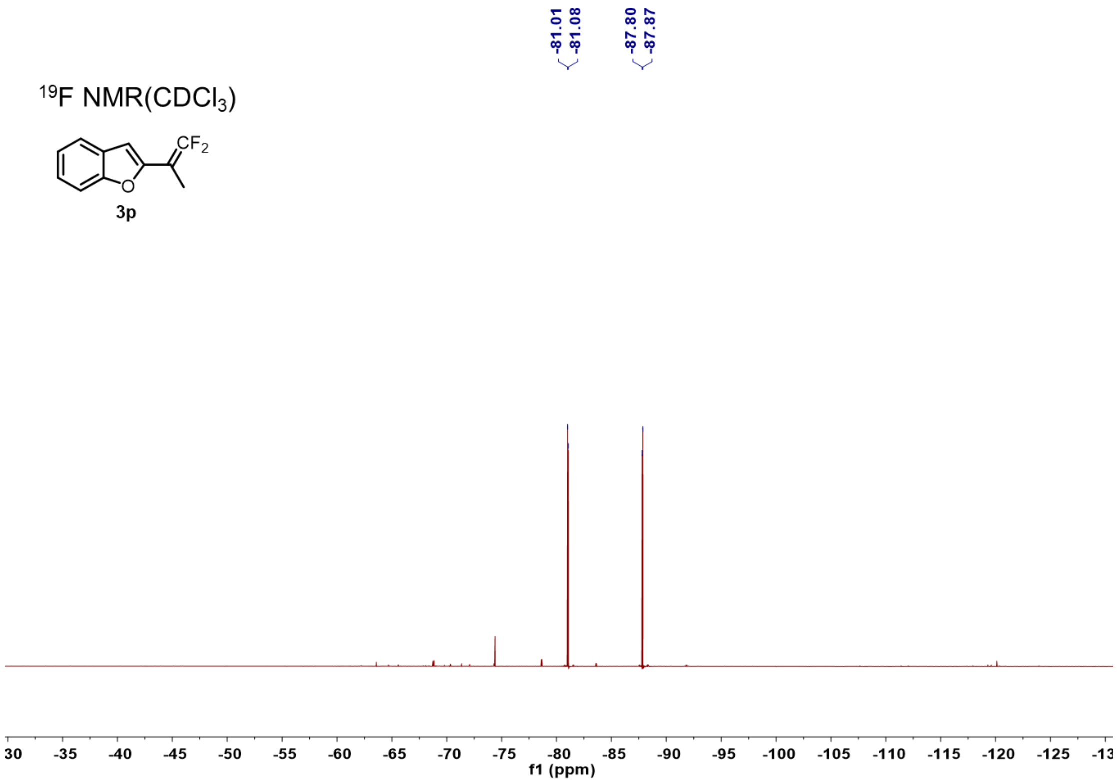

Supplementary Figure 103. ^{19}F NMR Spectrum of **3p**.

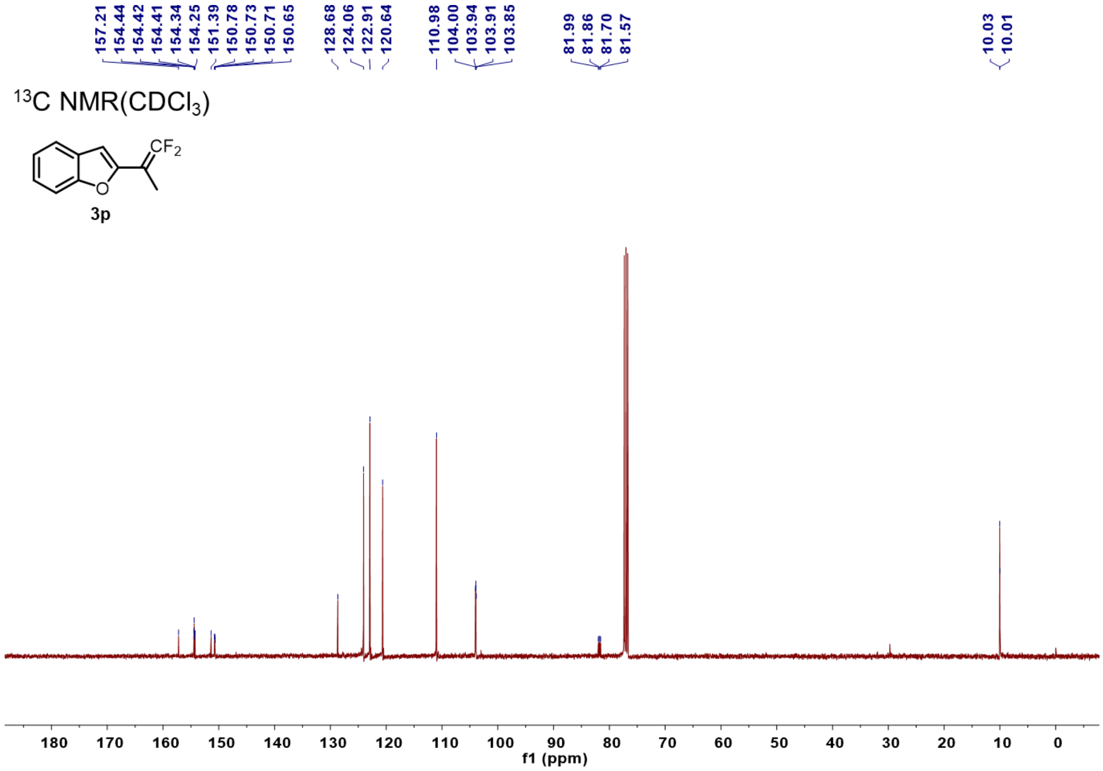

Supplementary Figure 104. ^{13}C NMR Spectrum of **3p**.

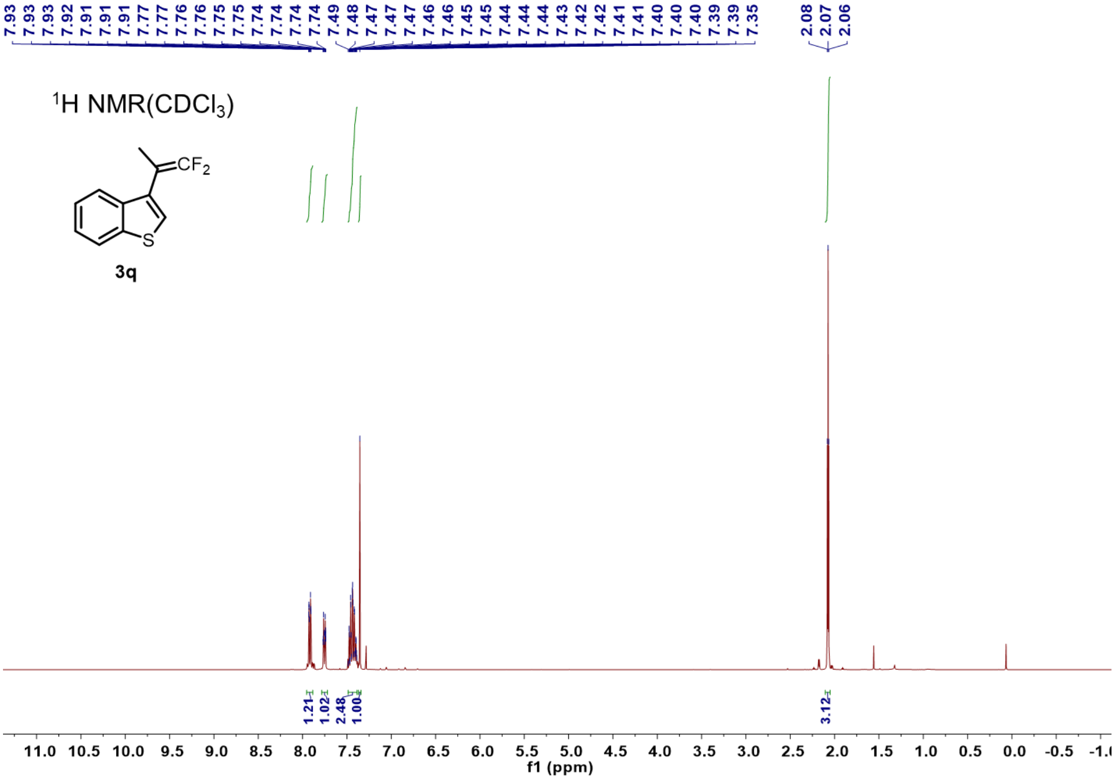

Supplementary Figure 105. ^{1}H NMR Spectrum of **3q**.

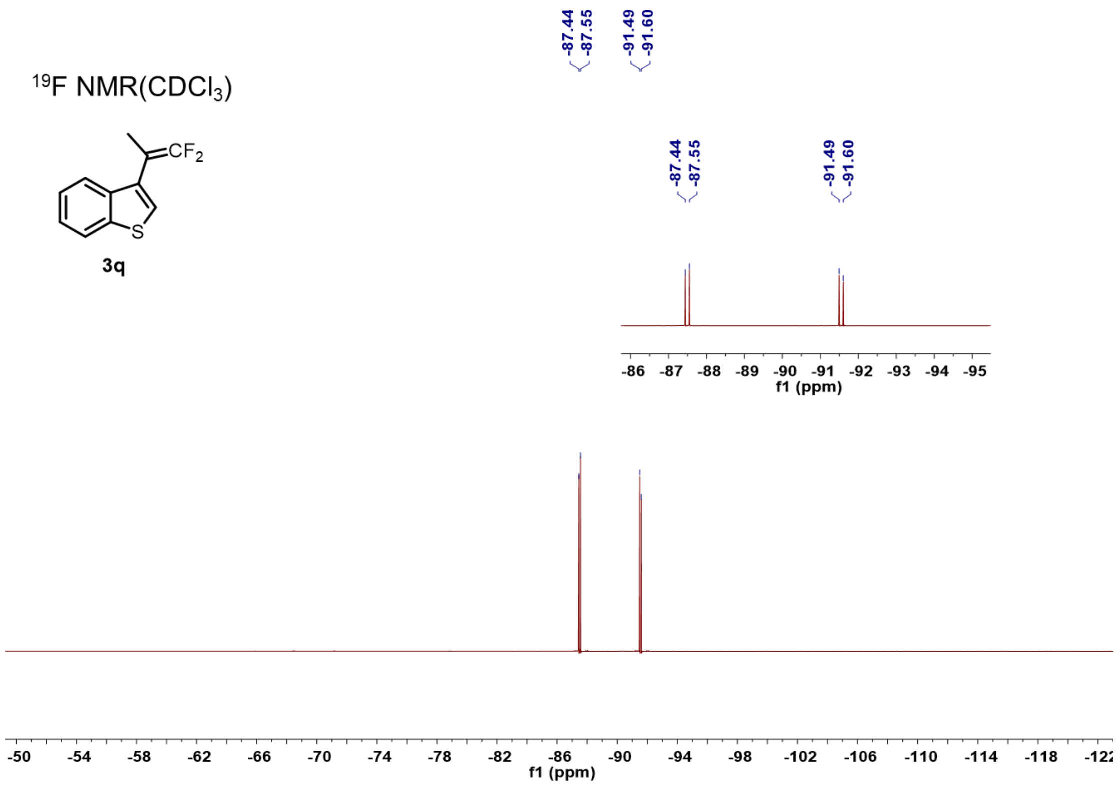

Supplementary Figure 106. ^{19}F NMR Spectrum of **3q**.

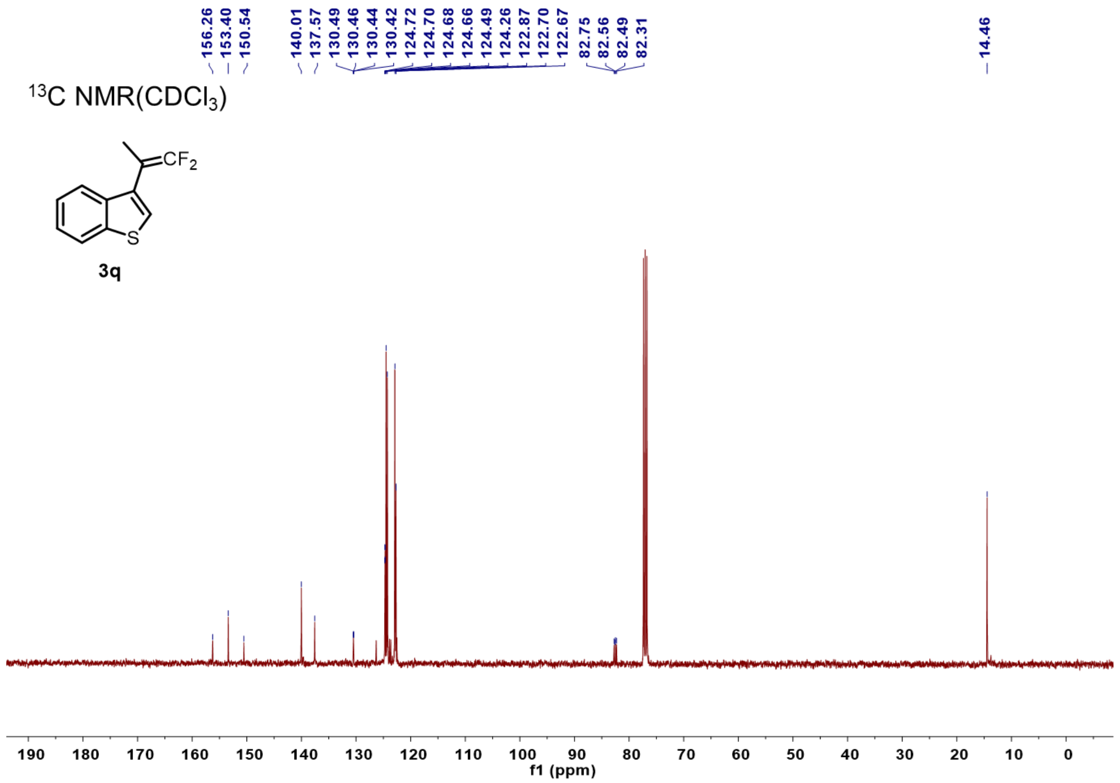

Supplementary Figure 107. ^{13}C NMR Spectrum of **3q**.

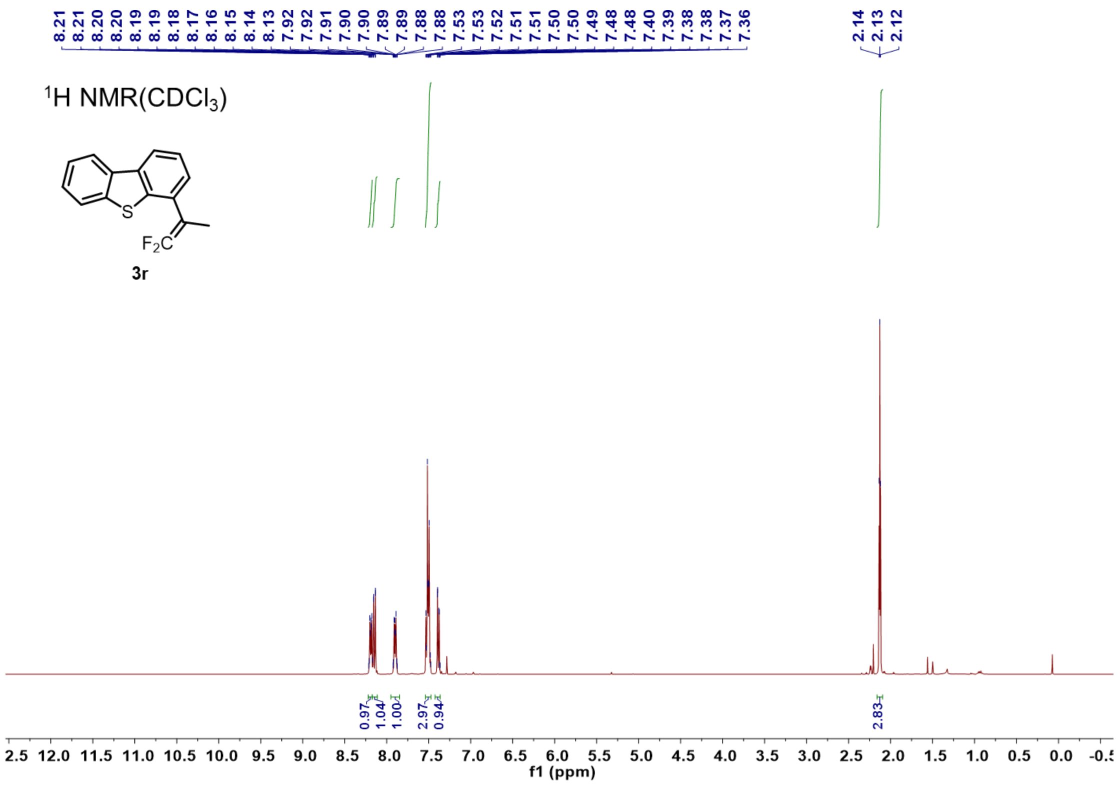

Supplementary Figure 108. ^{1}H NMR Spectrum of **3r**.

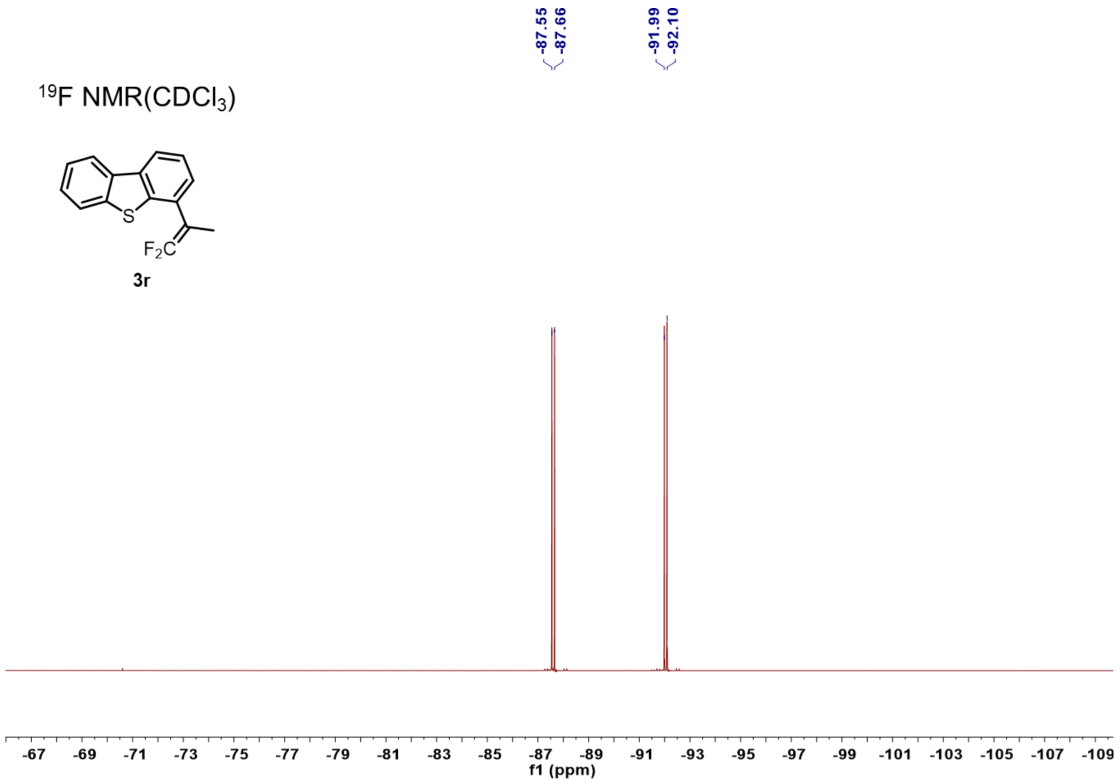

Supplementary Figure 109. ^{19}F NMR Spectrum of **3r**.

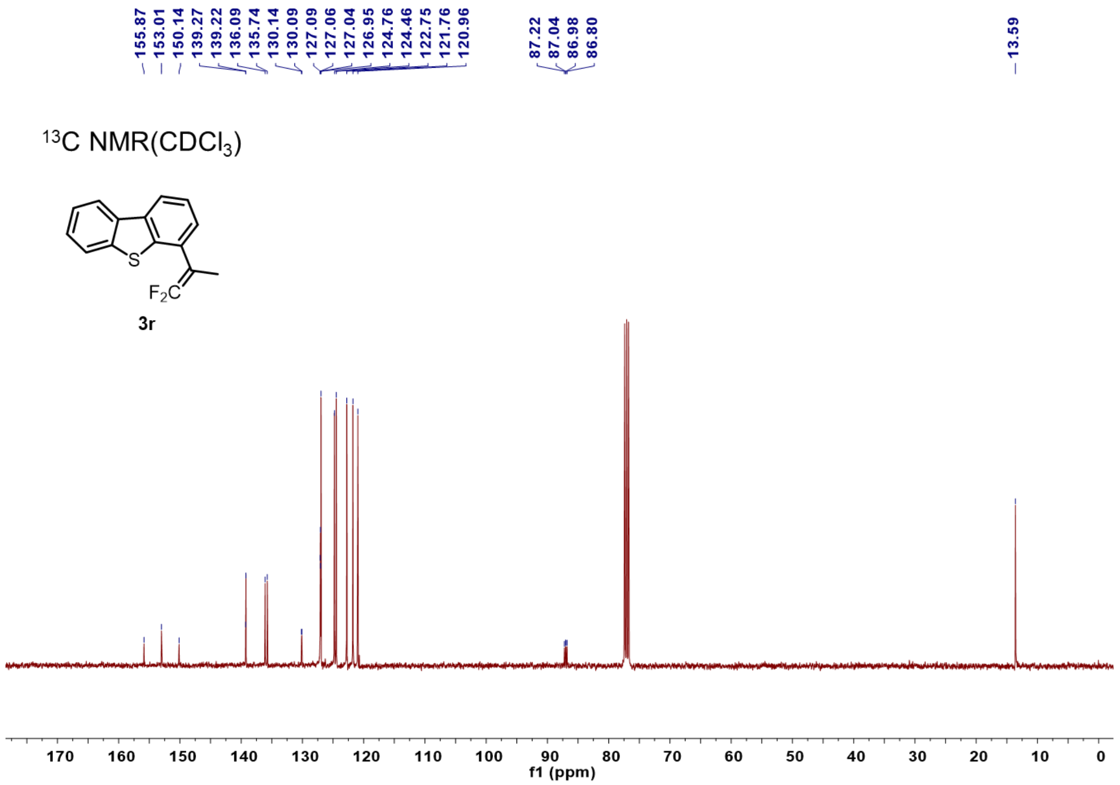

Supplementary Figure 110. ^{13}C NMR Spectrum of **3r**.

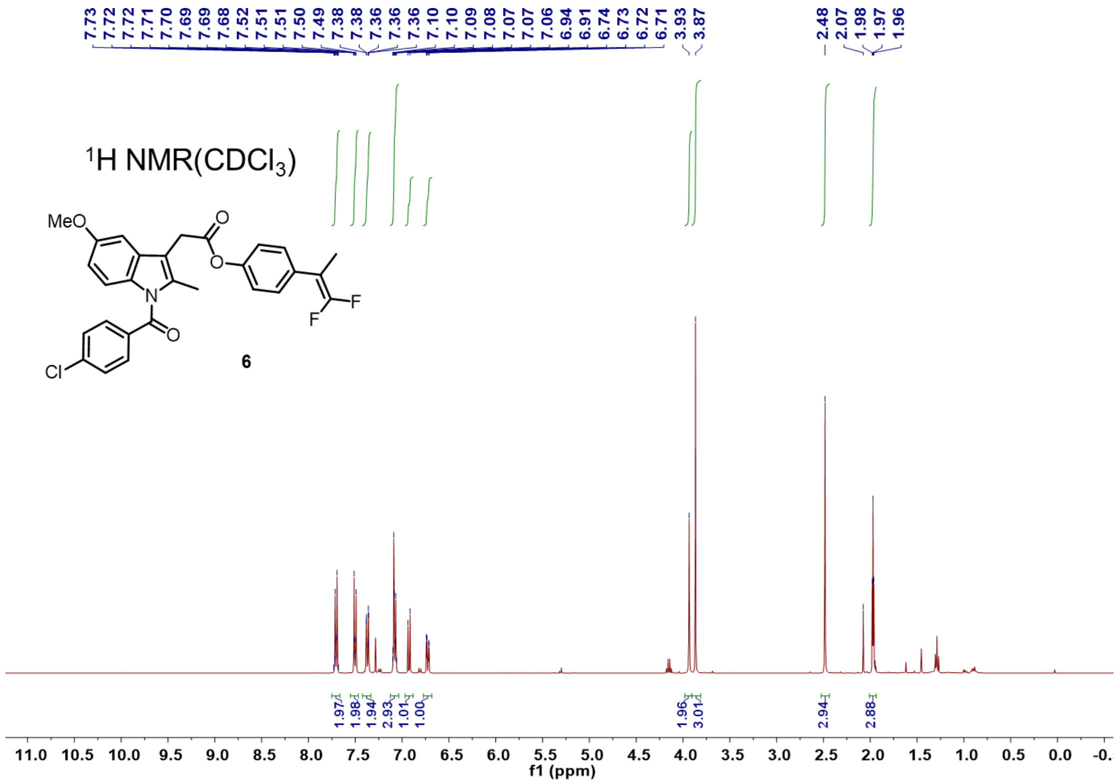

Supplementary Figure 111. ^{1}H NMR Spectrum of **6**.

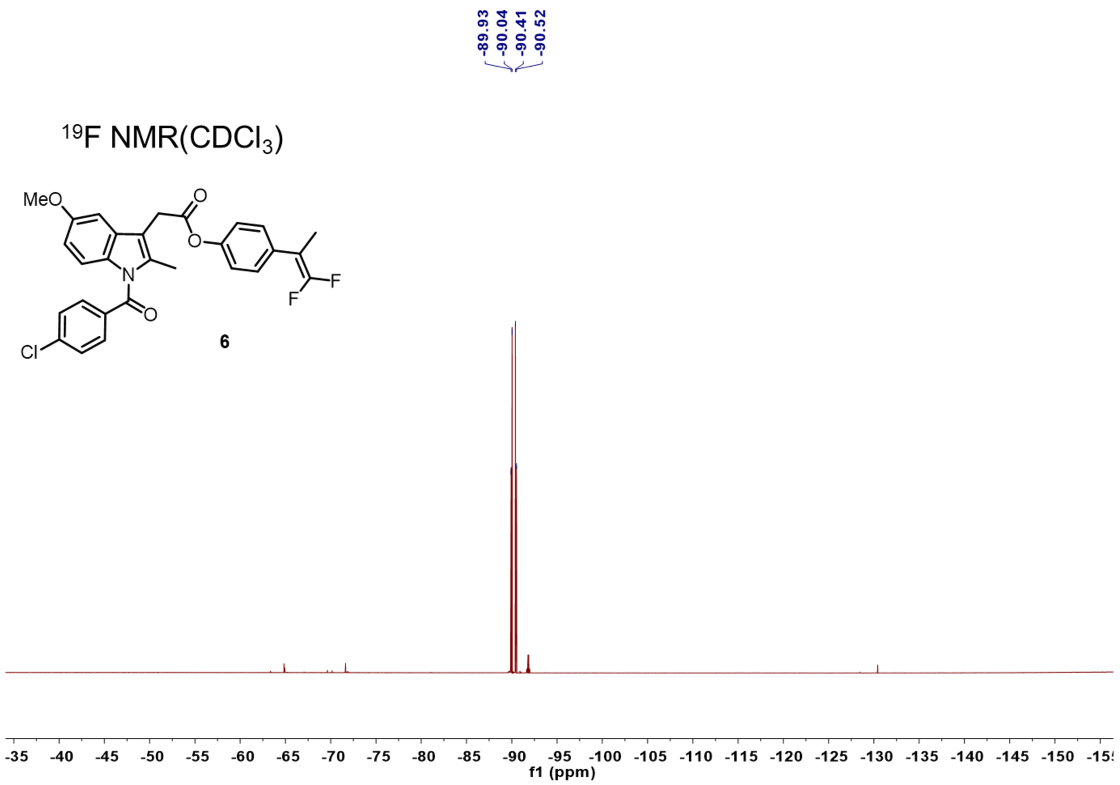

Supplementary Figure 112. ^{19}F NMR Spectrum of **6**.

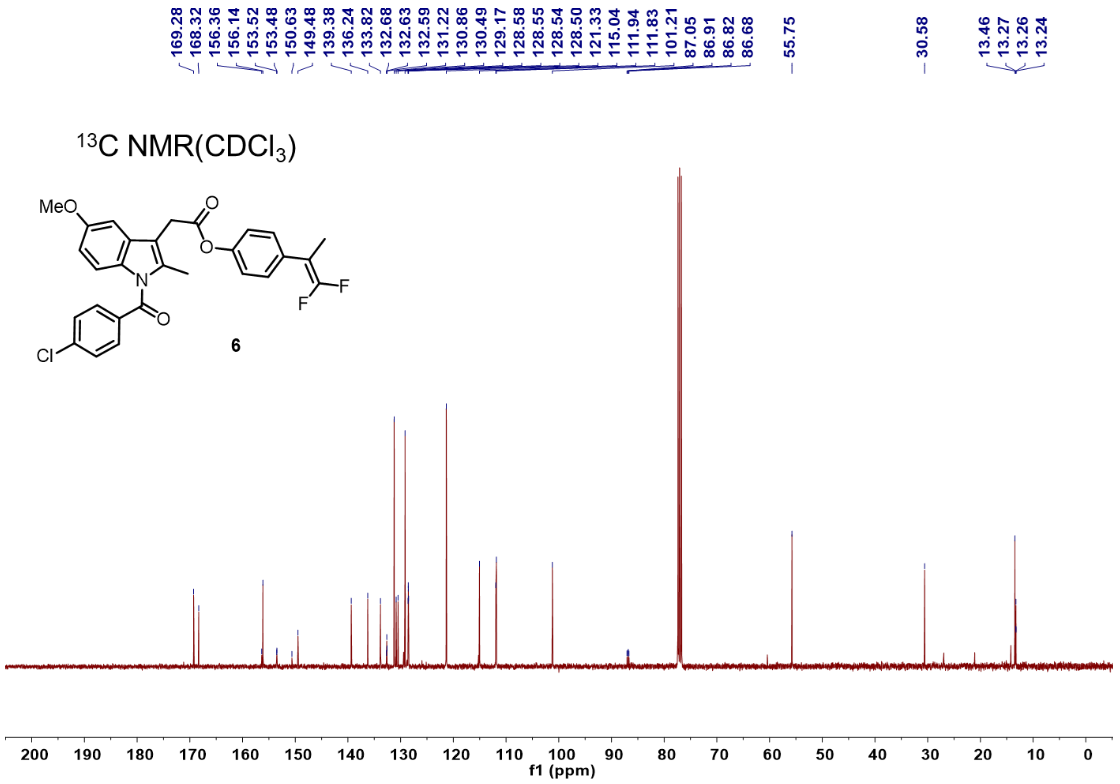

Supplementary Figure 113. ^{13}C NMR Spectrum of **6**.

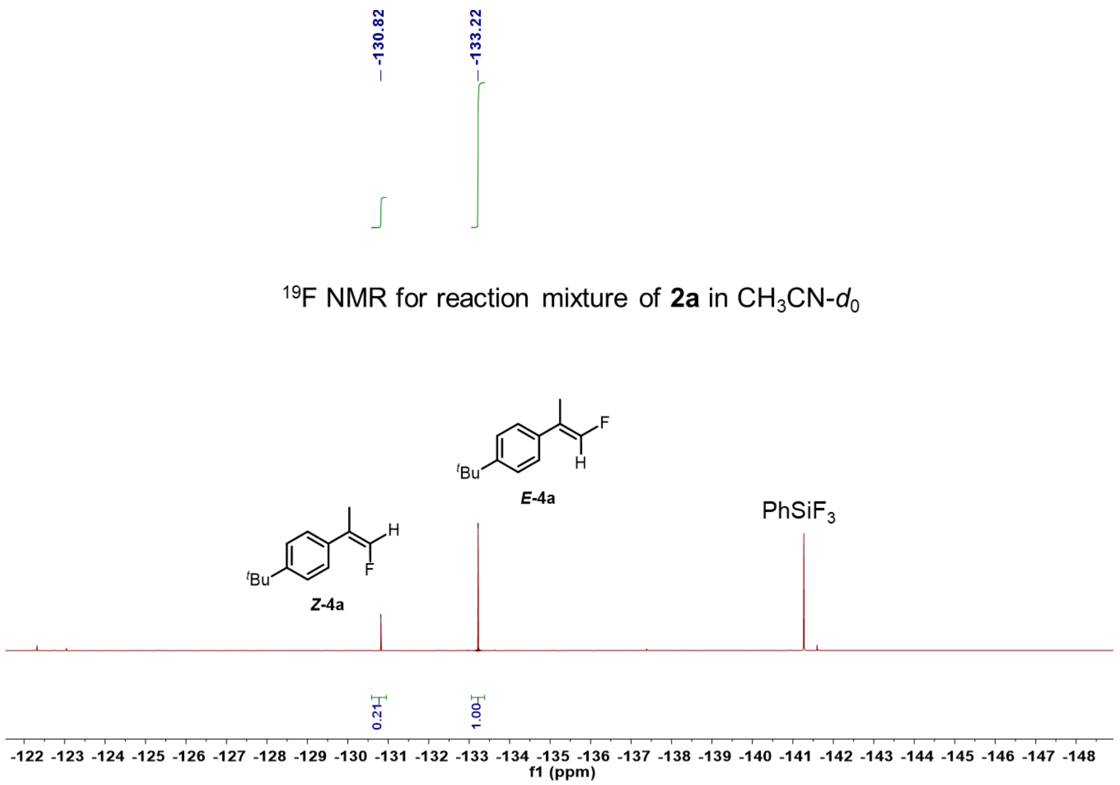

Supplementary Figure 114. ^{19}F NMR Spectrum for Reaction Mixture of **2a**.

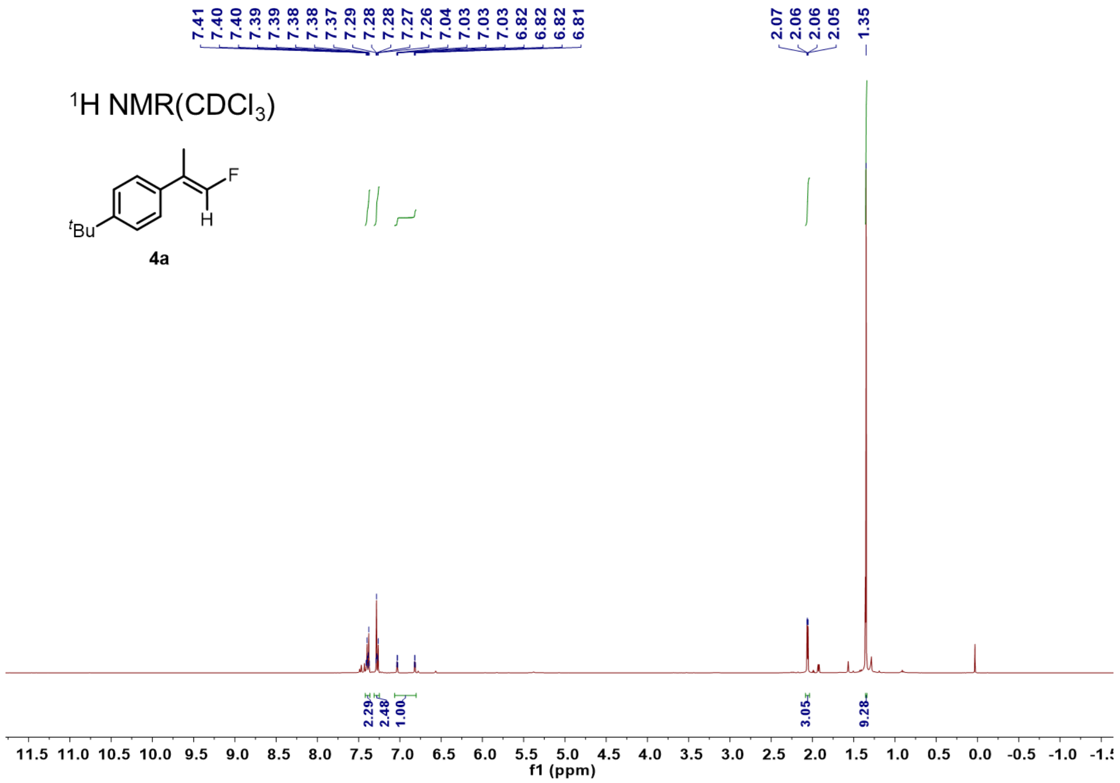

Supplementary Figure 115. ^{1}H NMR Spectrum of **4a**.

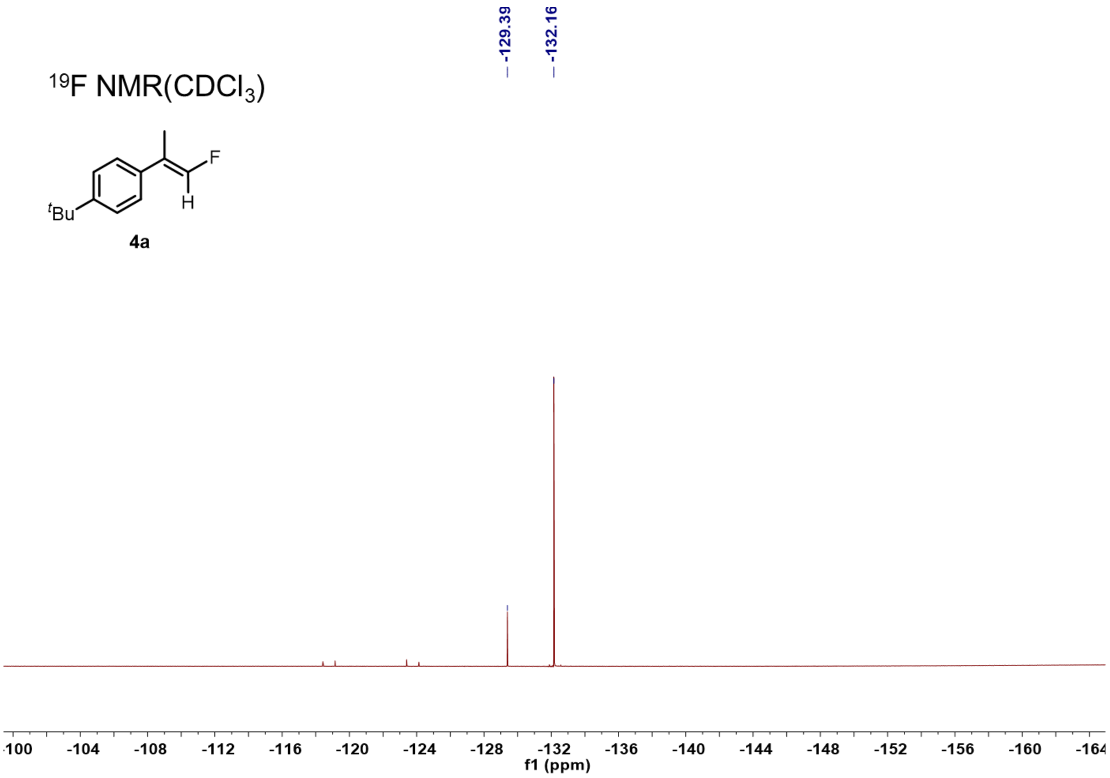

Supplementary Figure 116. ^{19}F NMR Spectrum of **4a**.

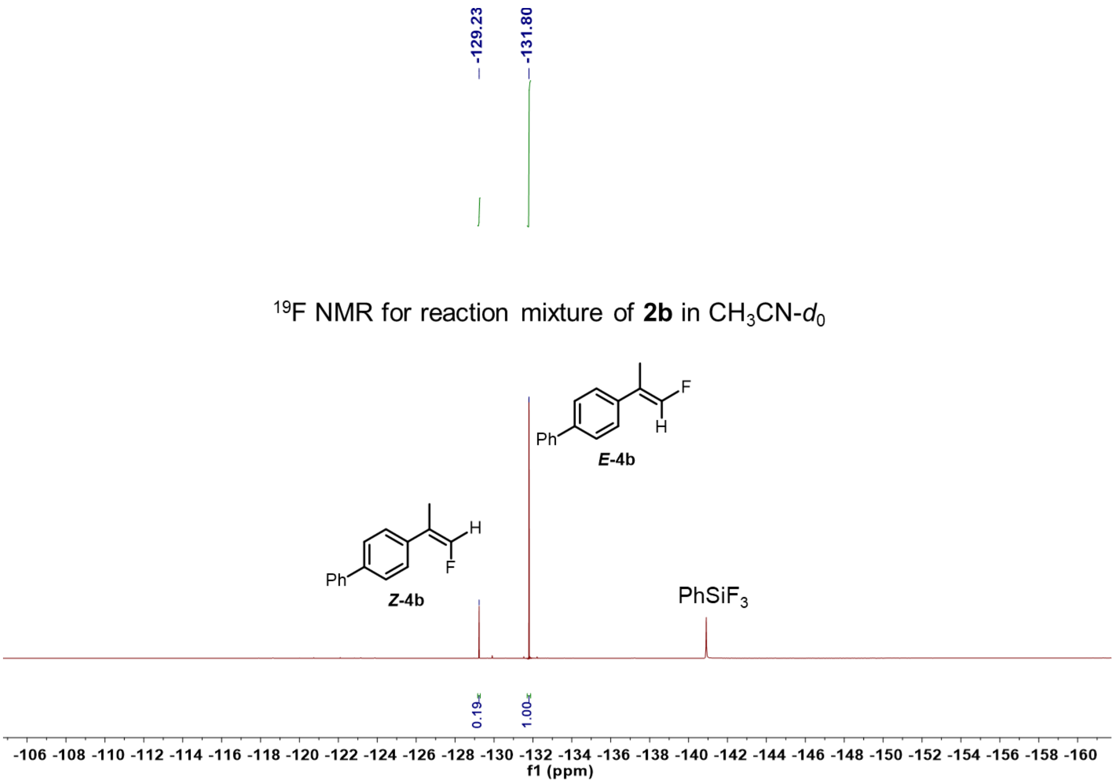

Supplementary Figure 117. ^{19}F NMR Spectrum for Reaction Mixture of **2b**.

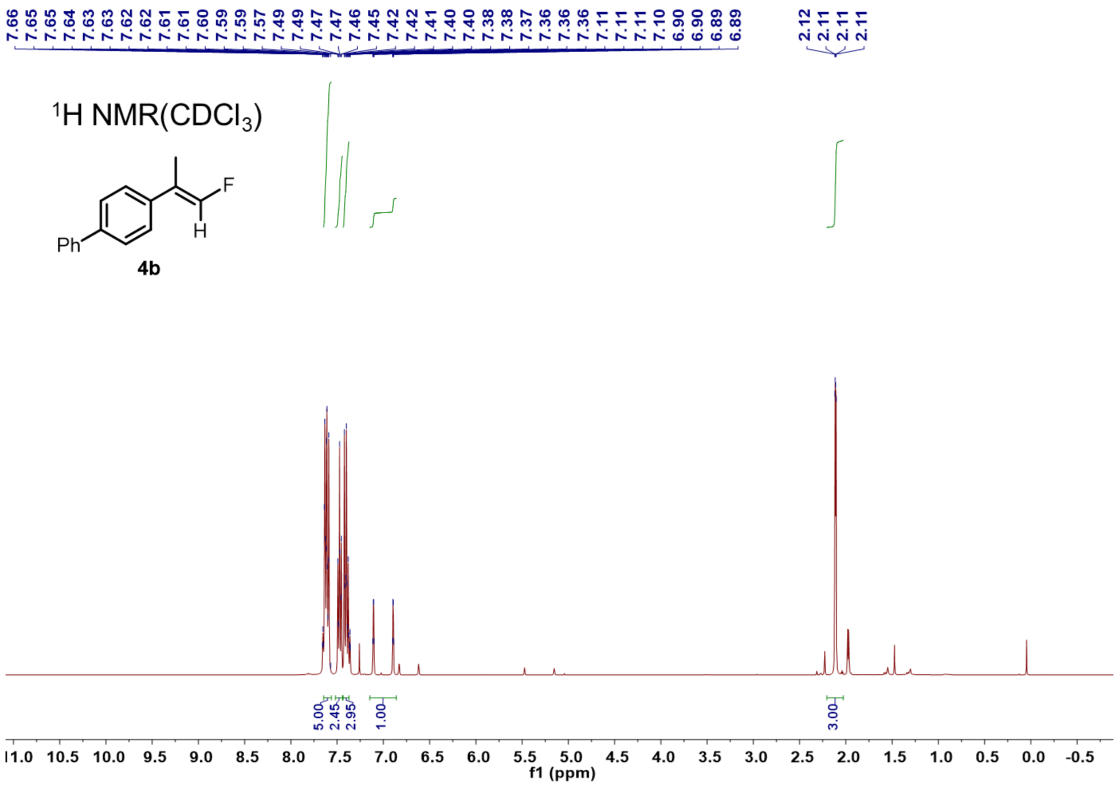

Supplementary Figure 118. ^{1}H NMR Spectrum of **4b**.

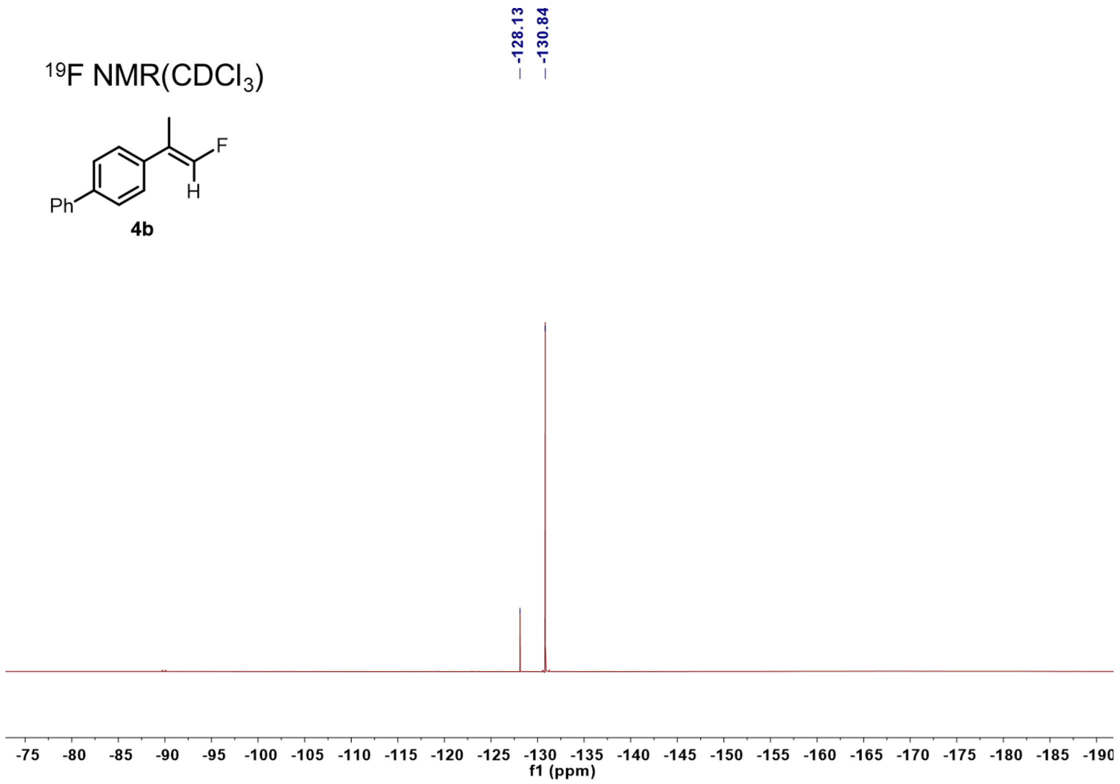

Supplementary Figure 119. ^{19}F NMR Spectrum of **4b**.

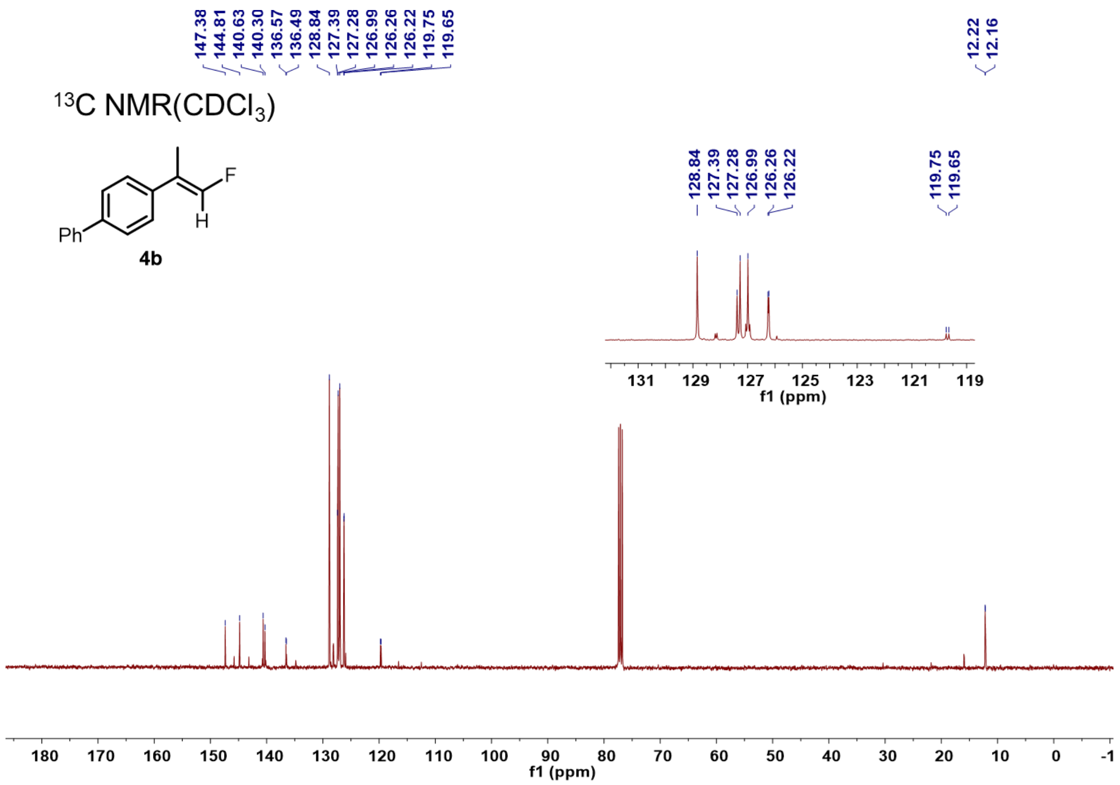

Supplementary Figure 120. ^{13}C NMR Spectrum of **4b**.

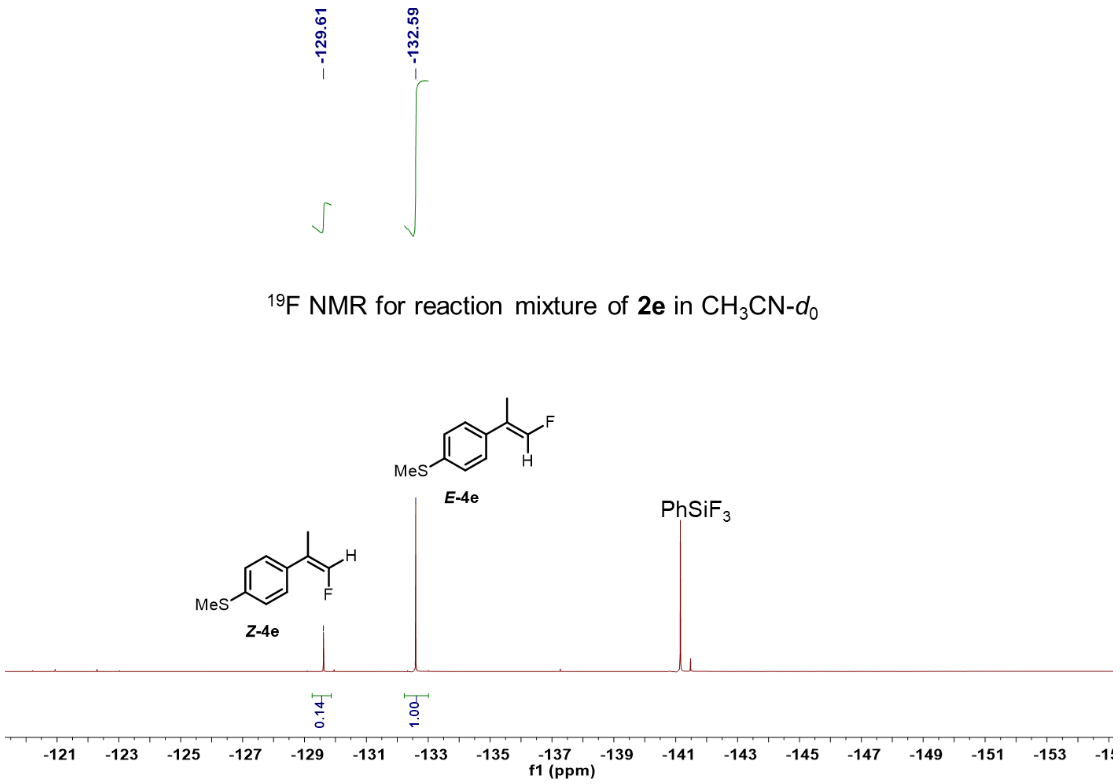

Supplementary Figure 121. ^{19}F NMR Spectrum for Reaction Mixture of **2e**.

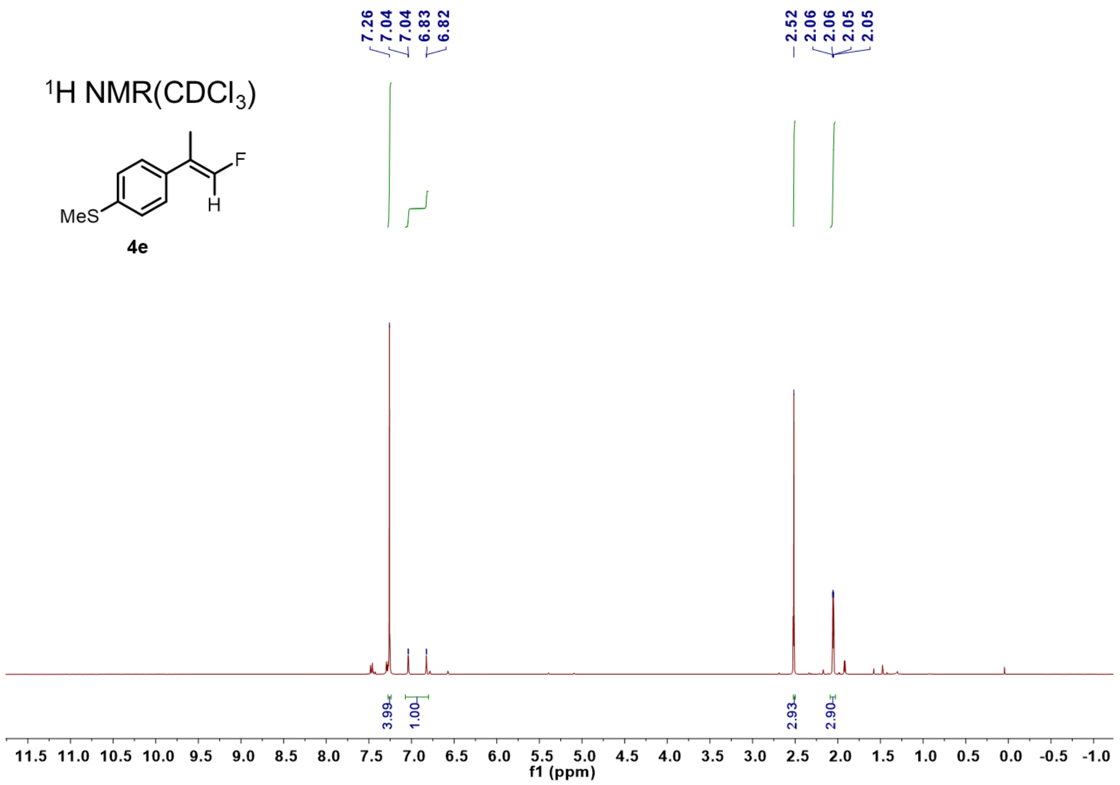

Supplementary Figure 122. ^{1}H NMR Spectrum of **4e**.

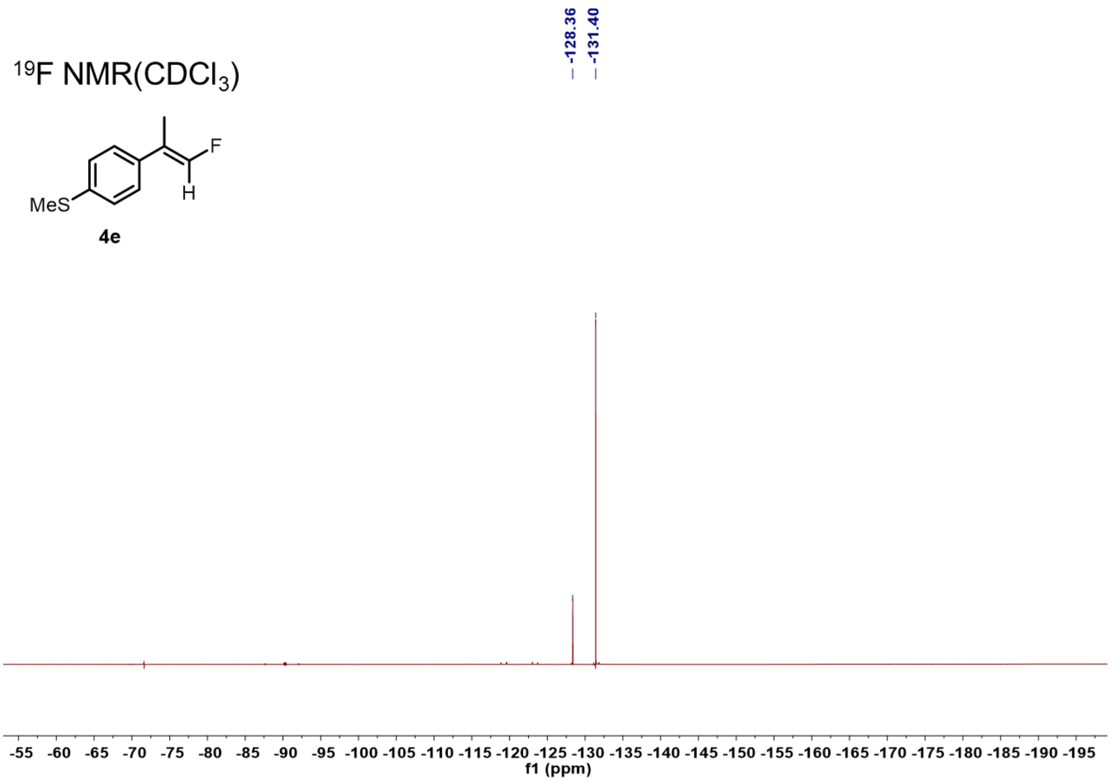

Supplementary Figure 123. ^{19}F NMR Spectrum of **4e**.

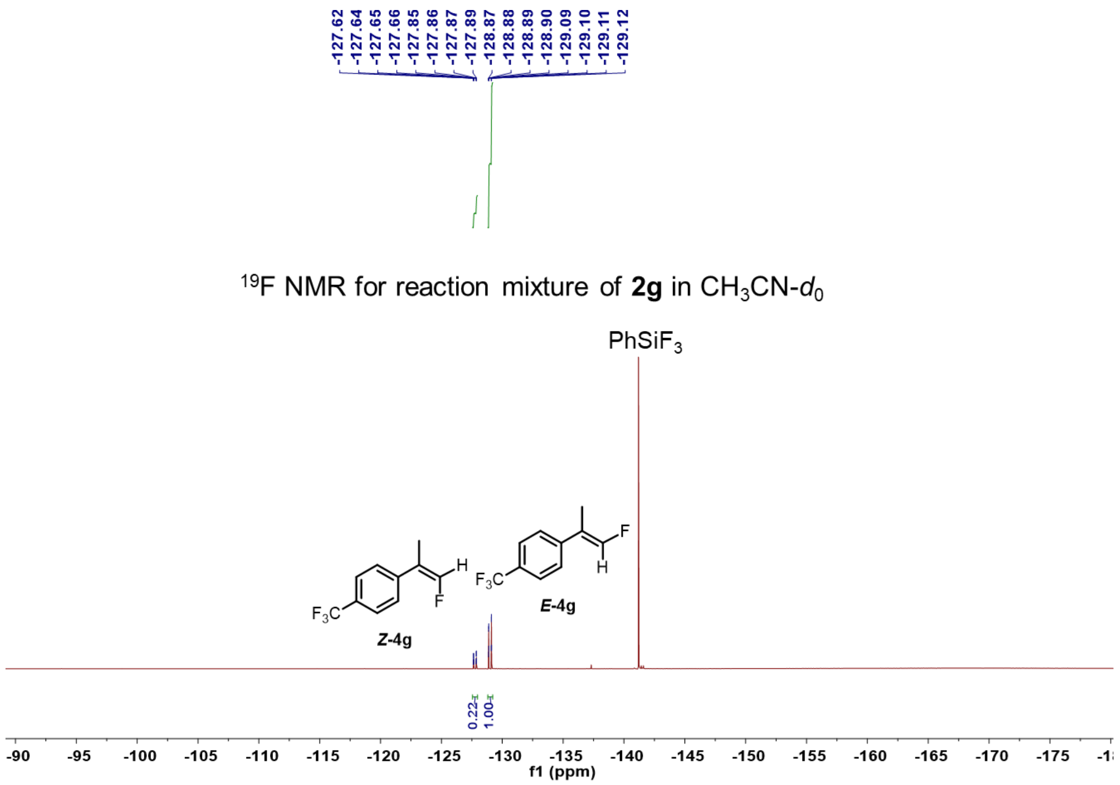

Supplementary Figure 124. ^{19}F NMR Spectrum for Reaction Mixture of **2g**.

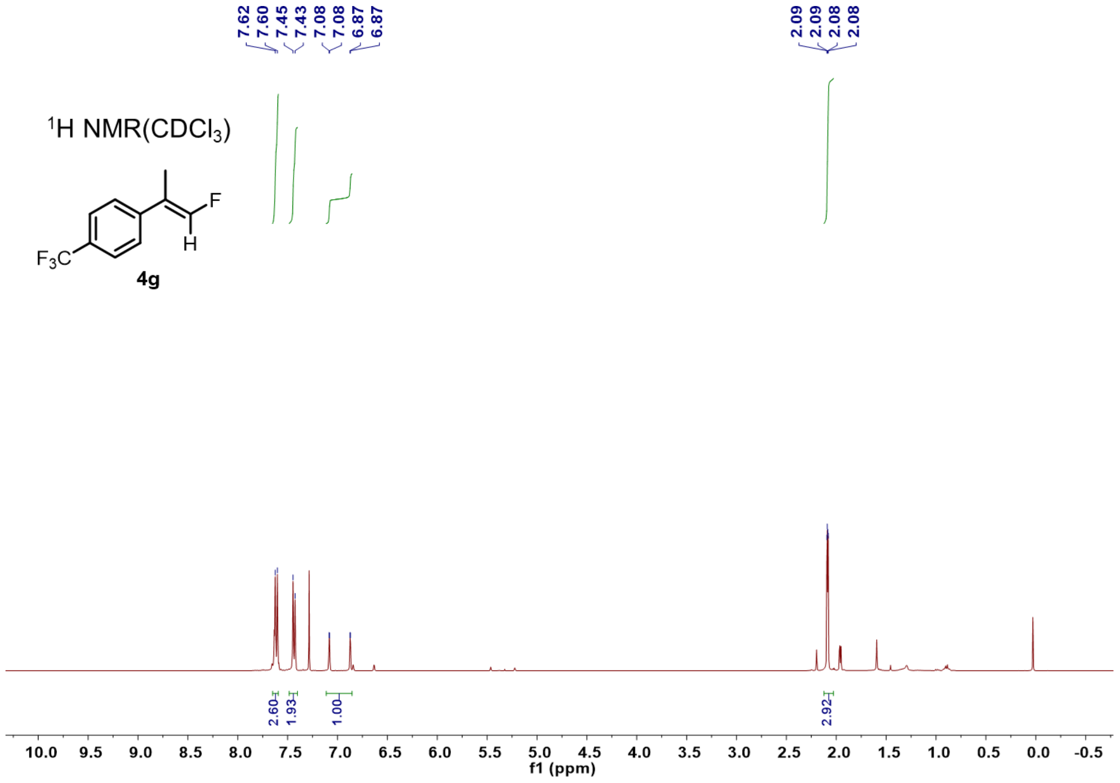

Supplementary Figure 125. ^{1}H NMR Spectrum of **4g**.

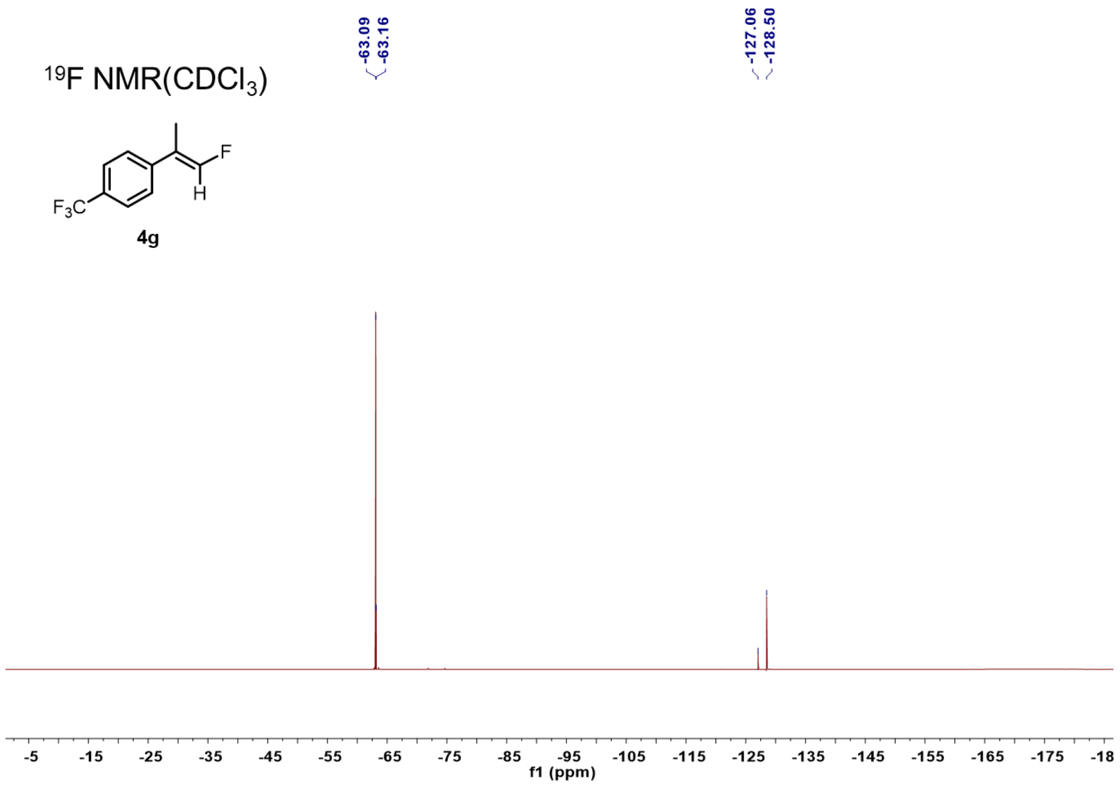

Supplementary Figure 126. ^{19}F NMR Spectrum of **4g**.

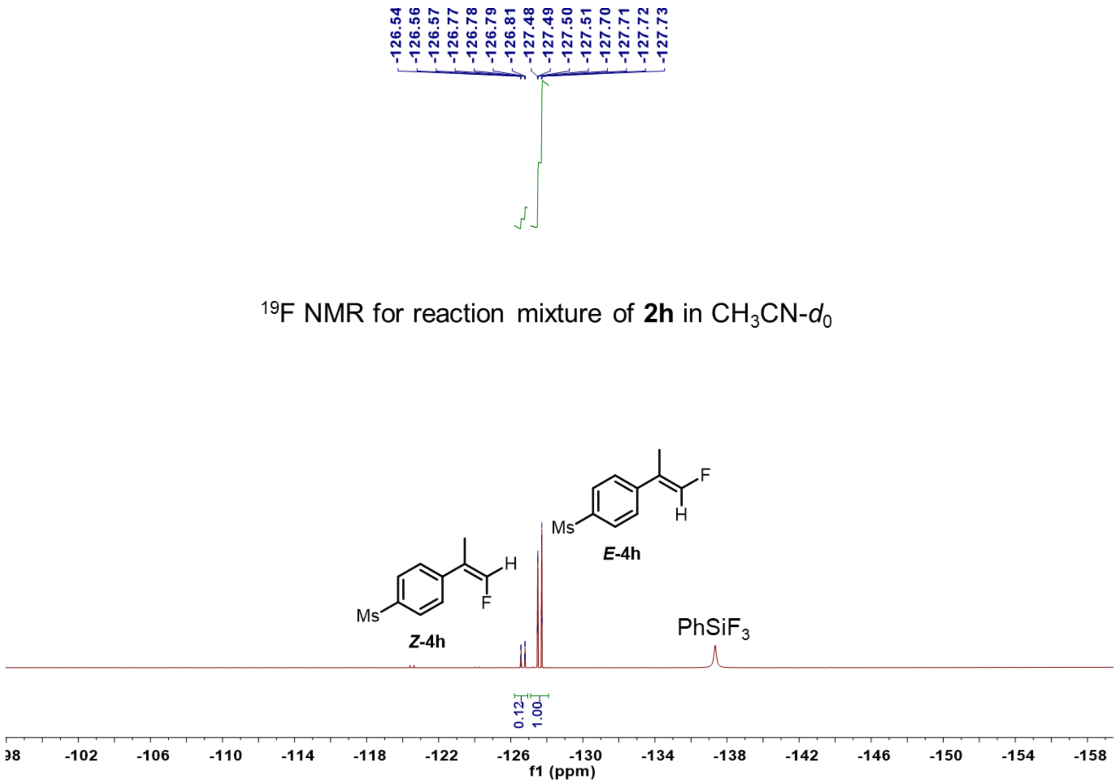

Supplementary Figure 127. ^{19}F NMR Spectrum for Reaction Mixture of **2h**.

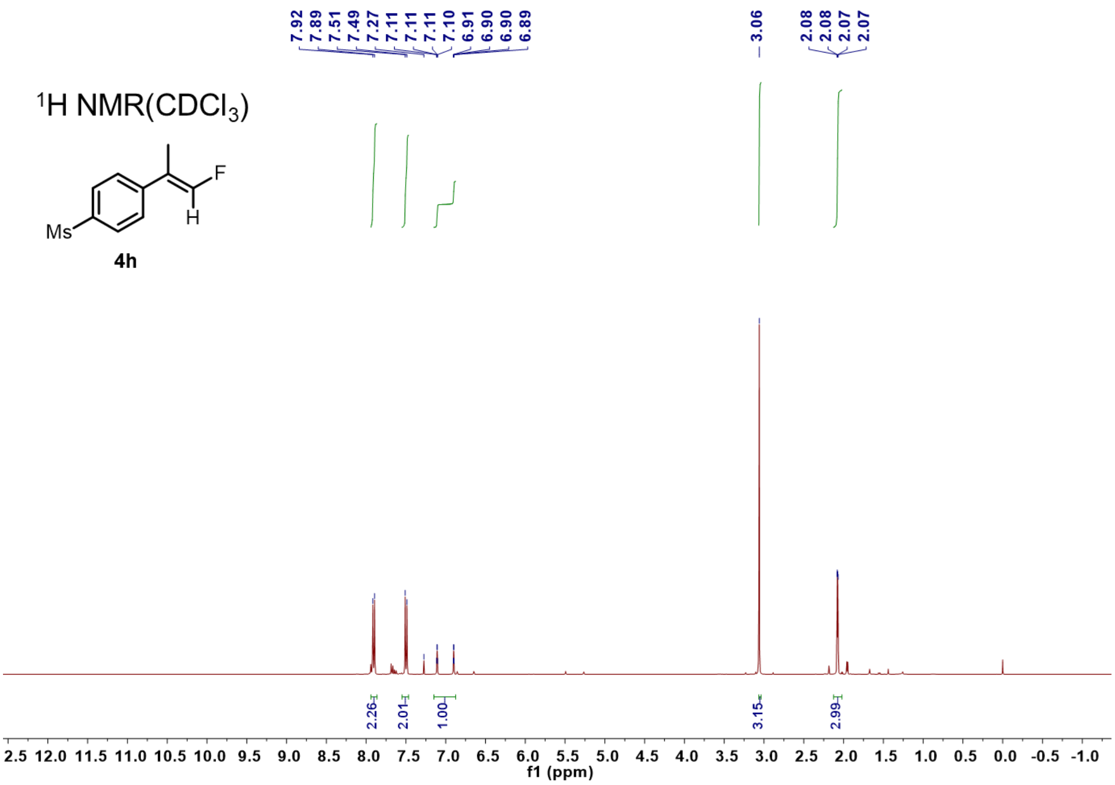

Supplementary Figure 128. ^{1}H NMR Spectrum of **4h**.

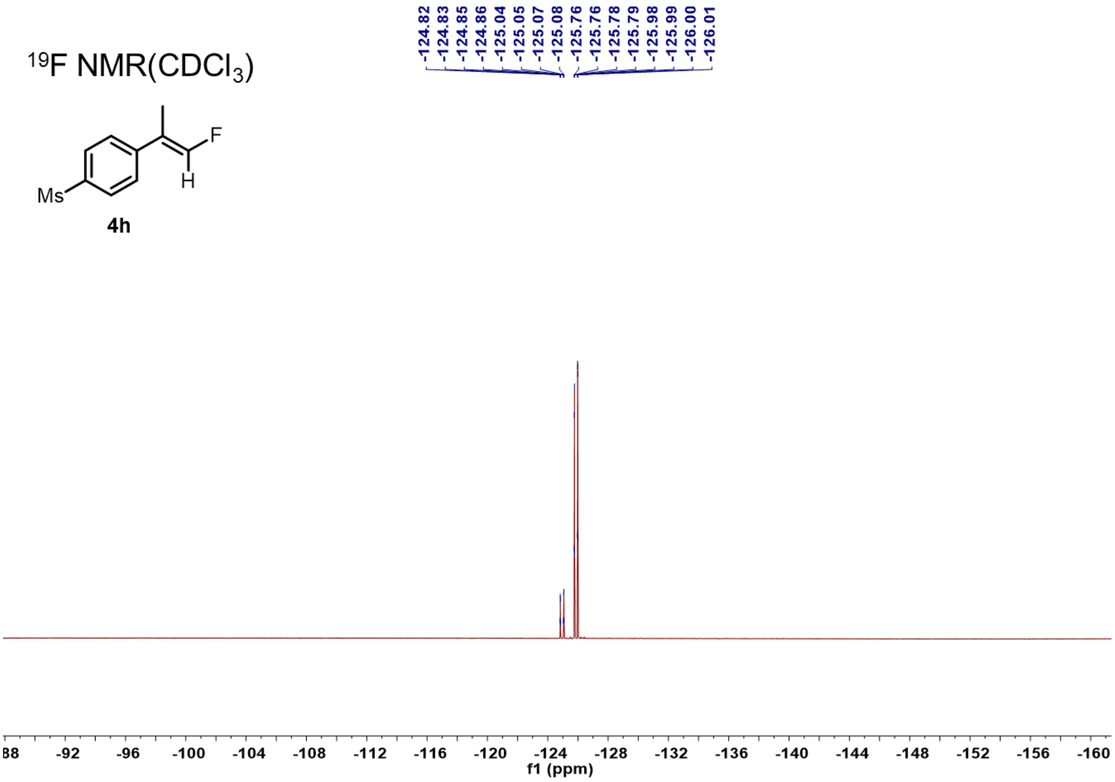

Supplementary Figure 129. ^{19}F NMR Spectrum of **4h**.

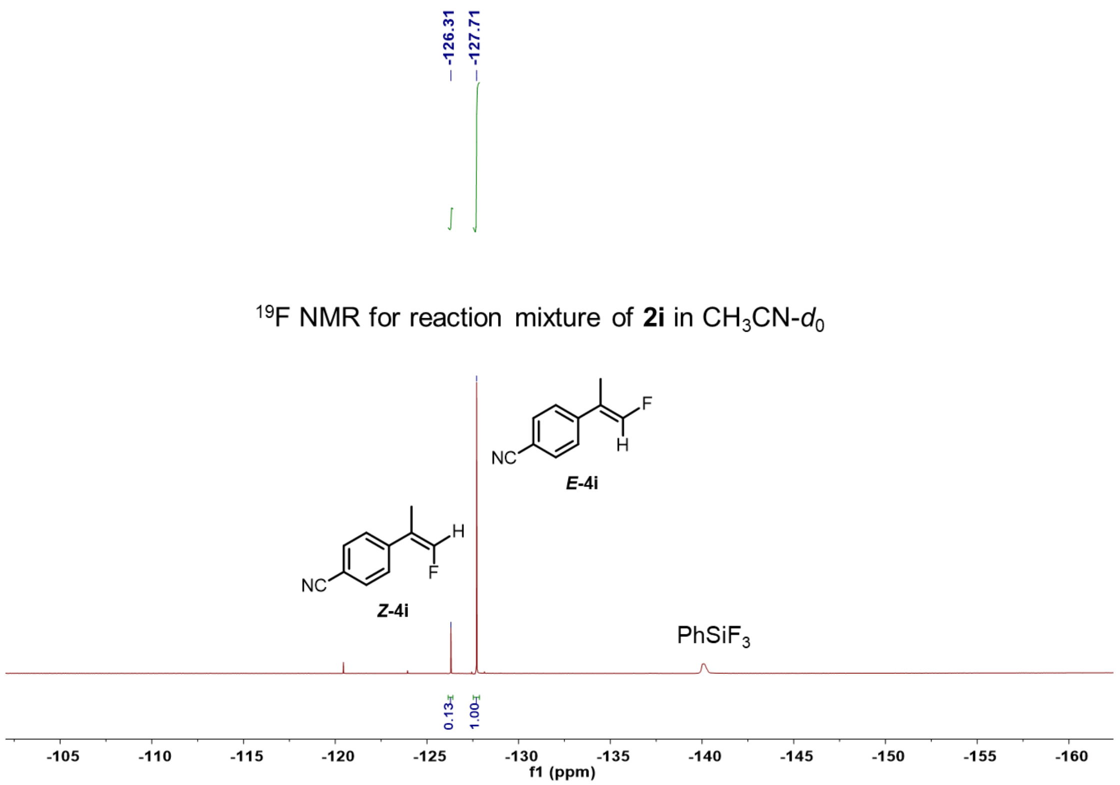

Supplementary Figure 130. ^{19}F NMR Spectrum for Reaction Mixture of **2i**.

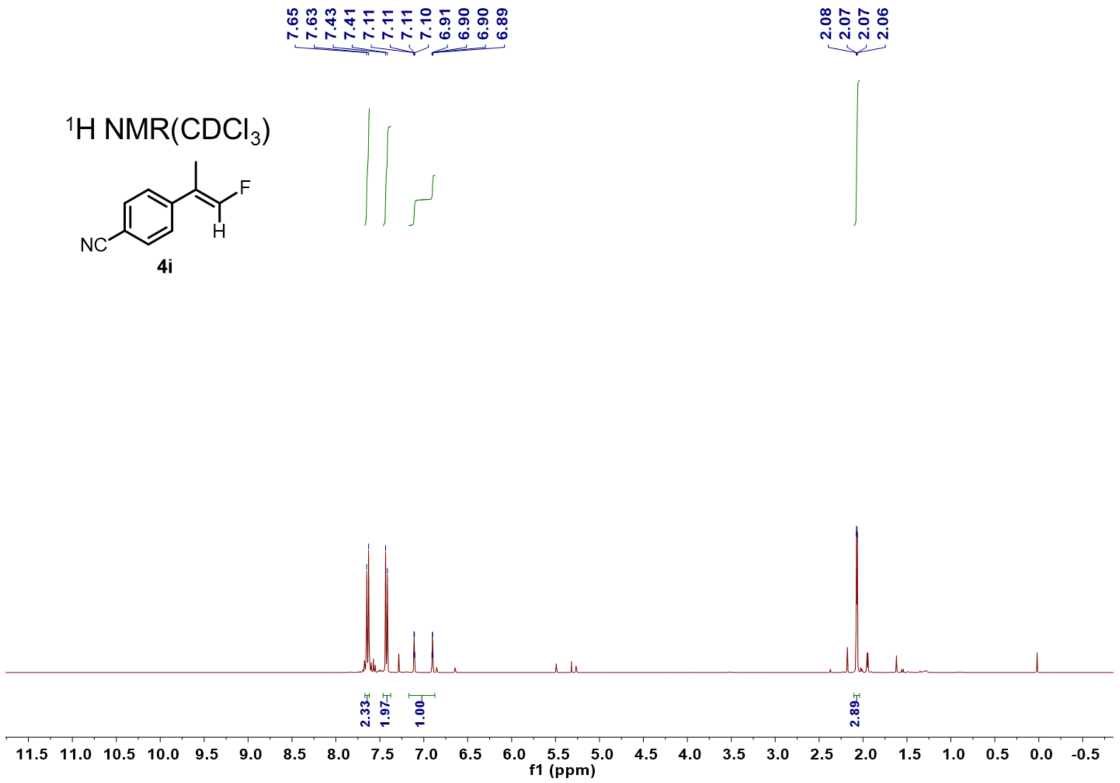

Supplementary Figure 131. ^{1}H NMR Spectrum of **4i**.

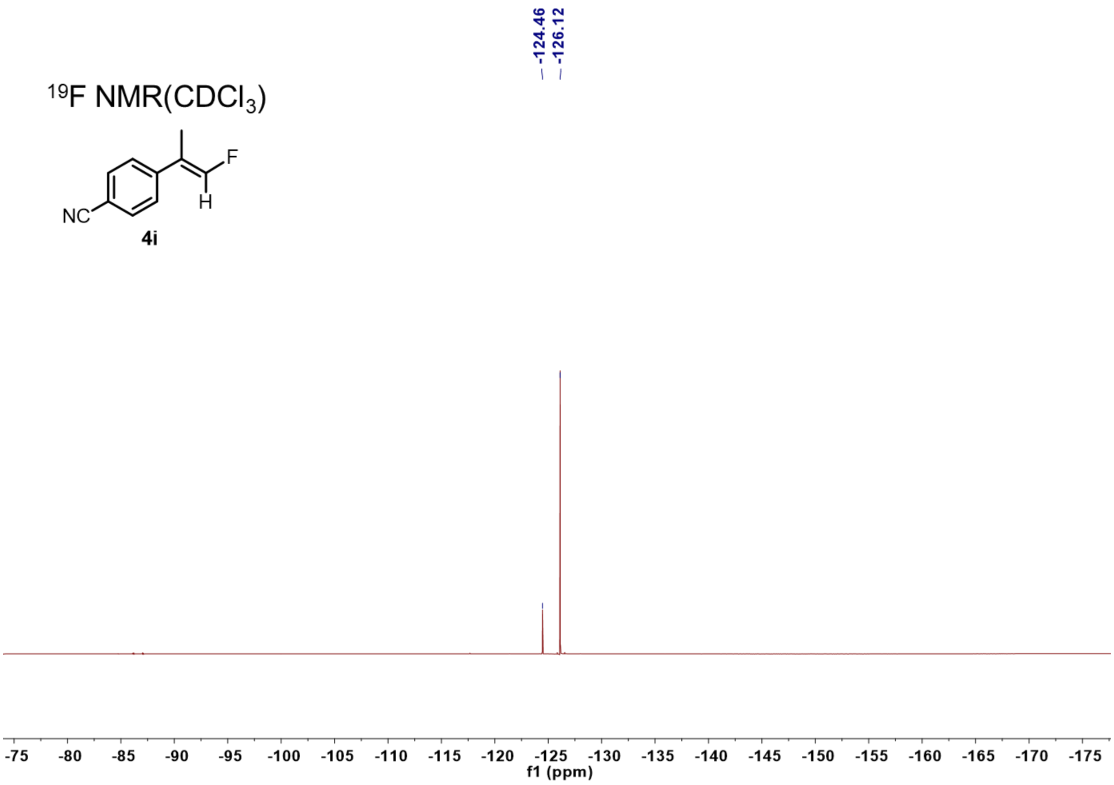

Supplementary Figure 132. ^{19}F NMR Spectrum of **4i**.

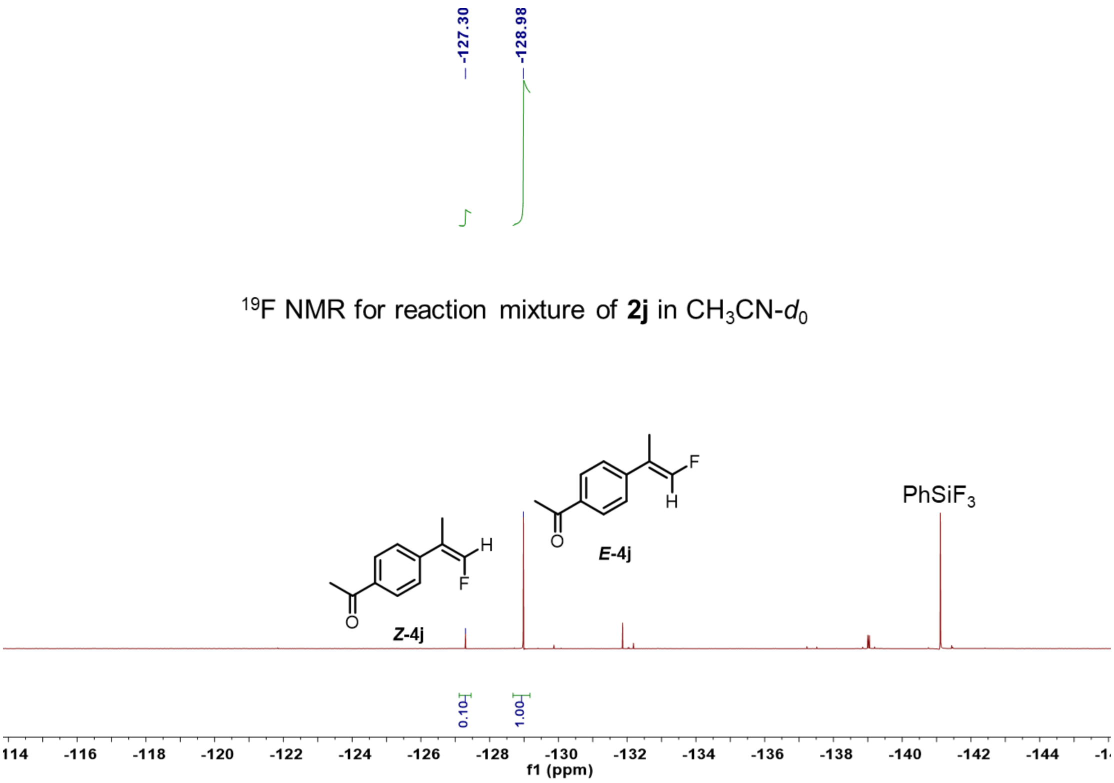

Supplementary Figure 133. ^{19}F NMR Spectrum for Reaction Mixture of **2j**.

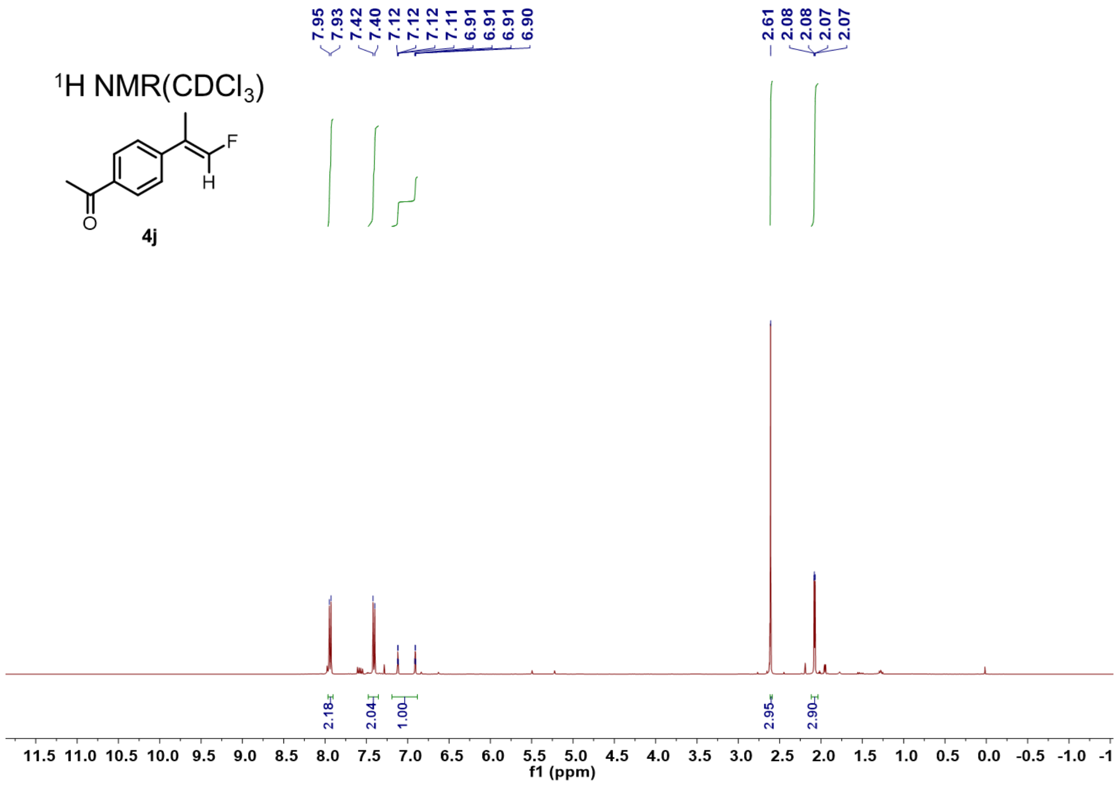

Supplementary Figure 134. ^{1}H NMR Spectrum of **4j**.

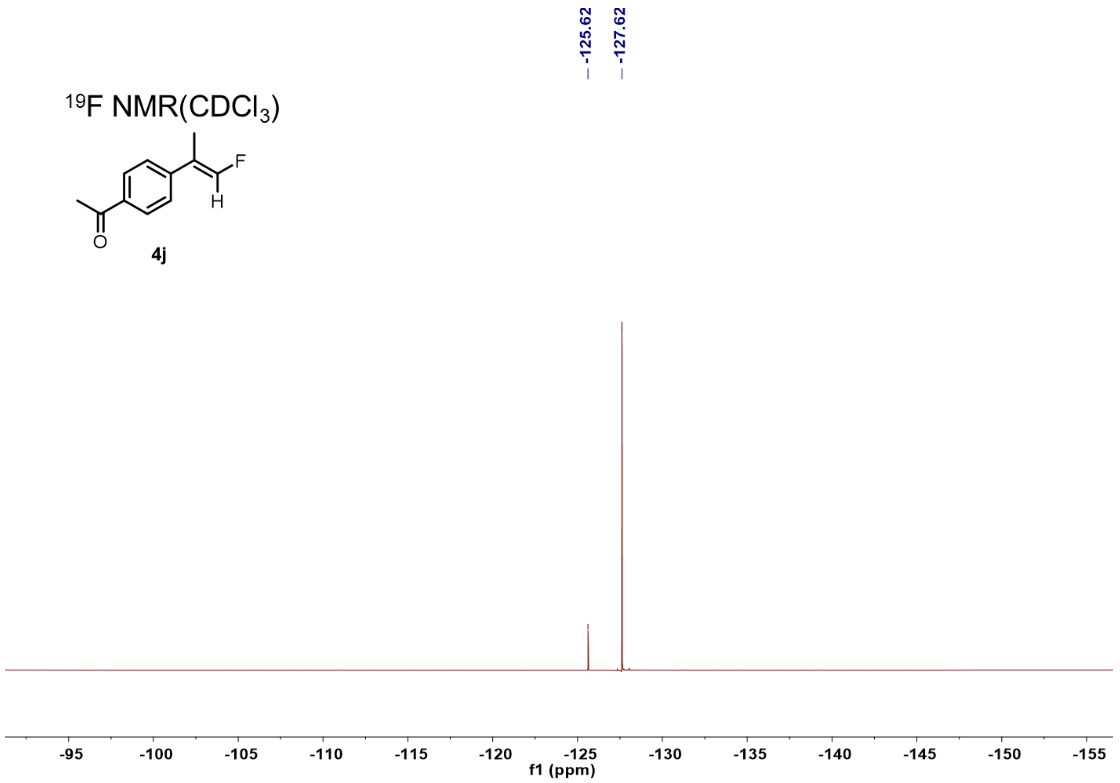

Supplementary Figure 135. ^{19}F NMR Spectrum of **4j**.

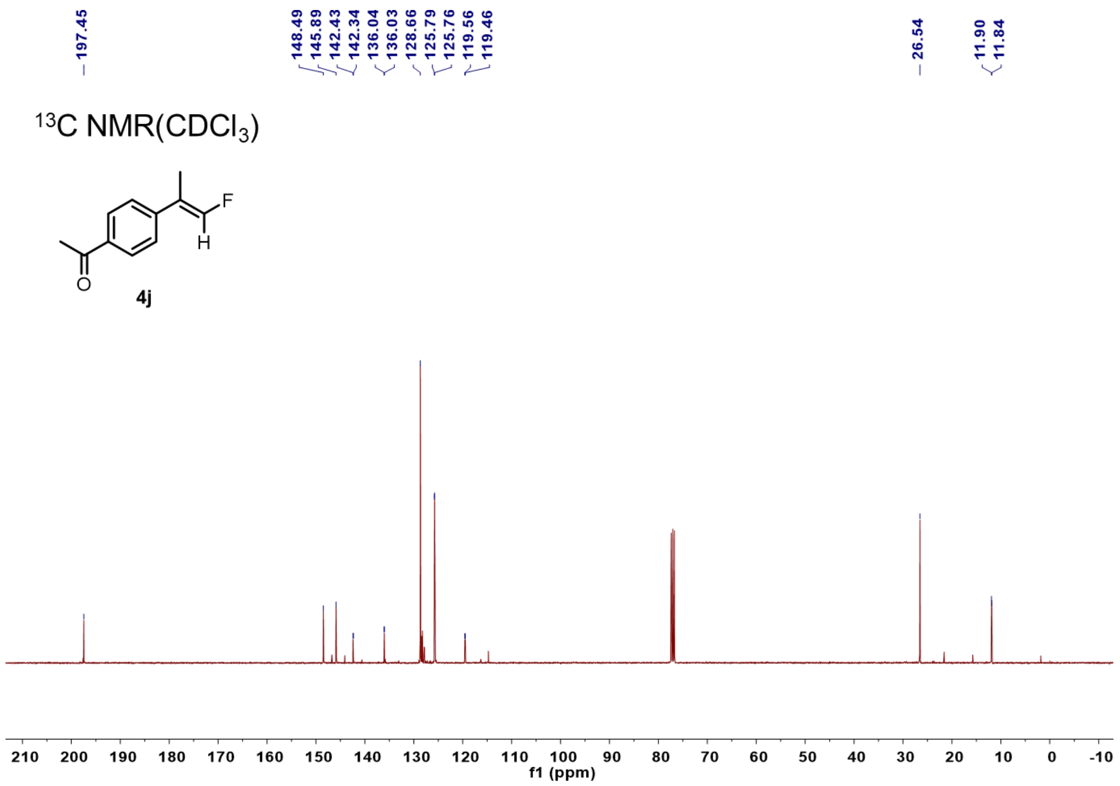

Supplementary Figure 136. ^{13}C NMR Spectrum of **4j**.

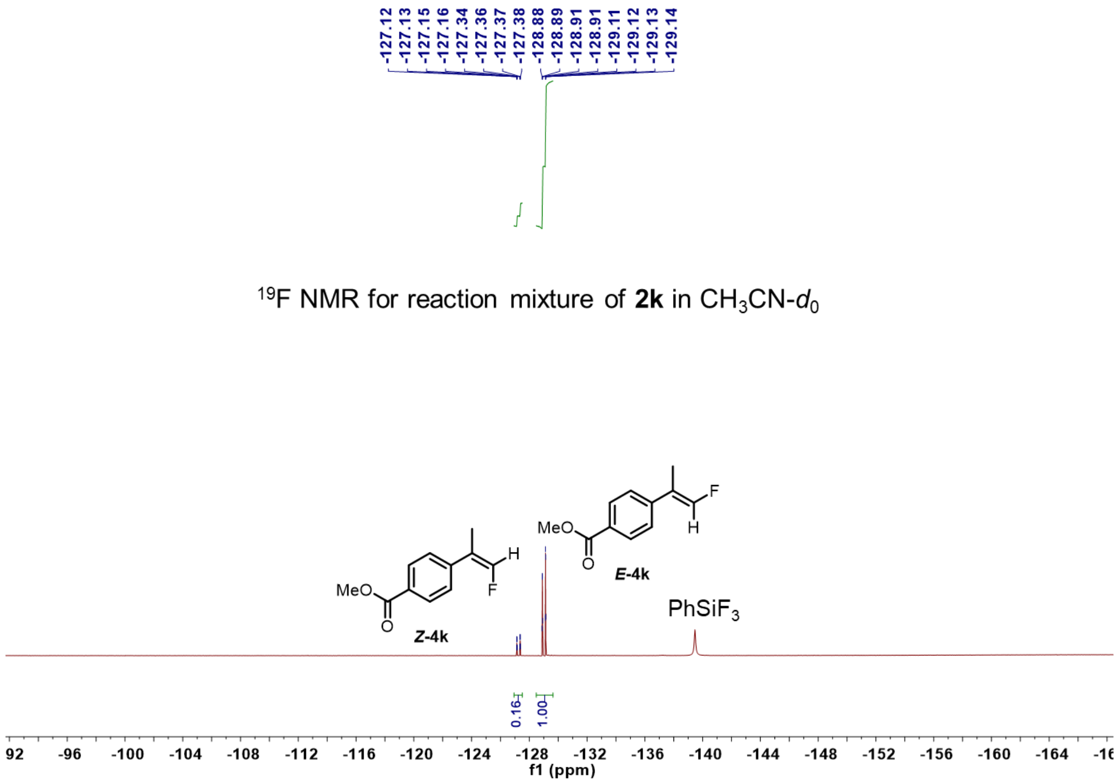

Supplementary Figure 137. ^{19}F NMR Spectrum for Reaction Mixture of **2k**.

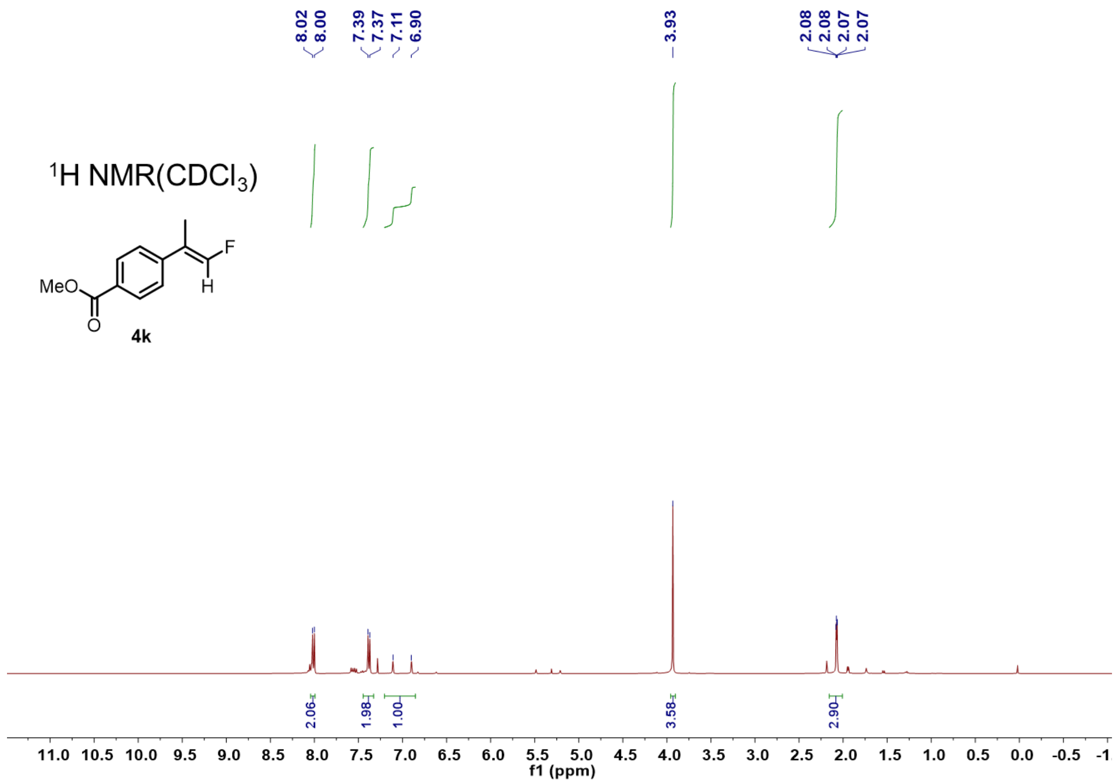

Supplementary Figure 138. ^{1}H NMR Spectrum of **4k**.

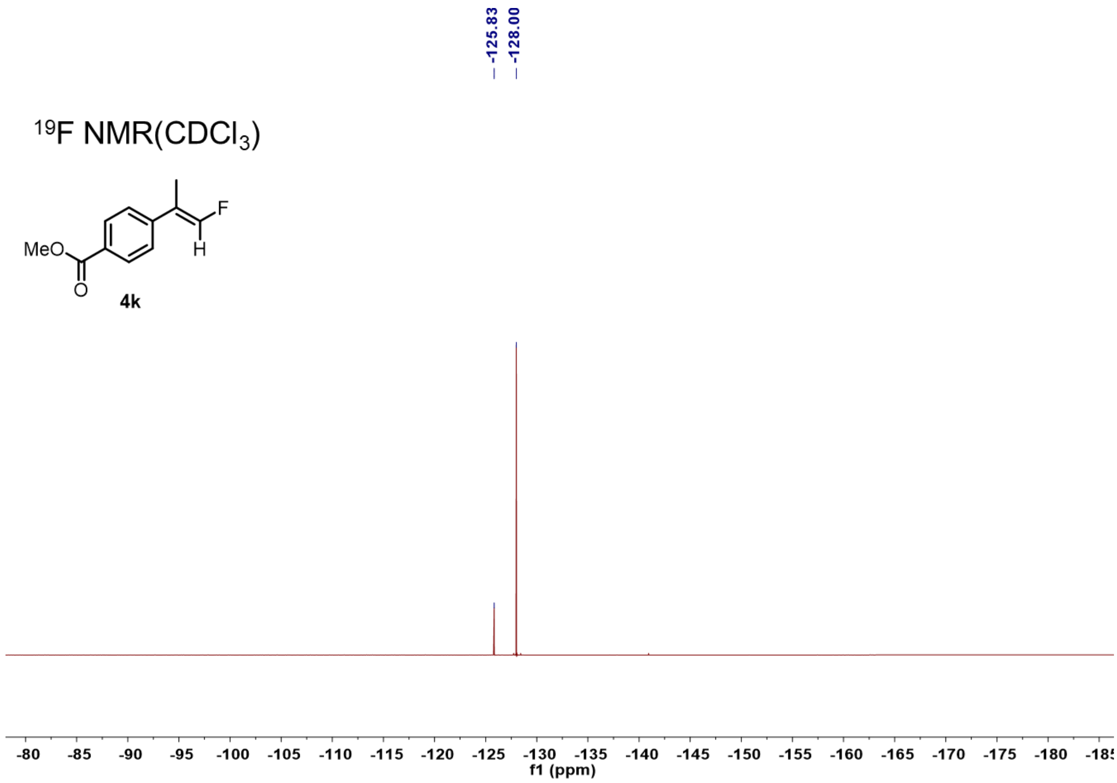

Supplementary Figure 139. ^{19}F NMR Spectrum of **4k**.

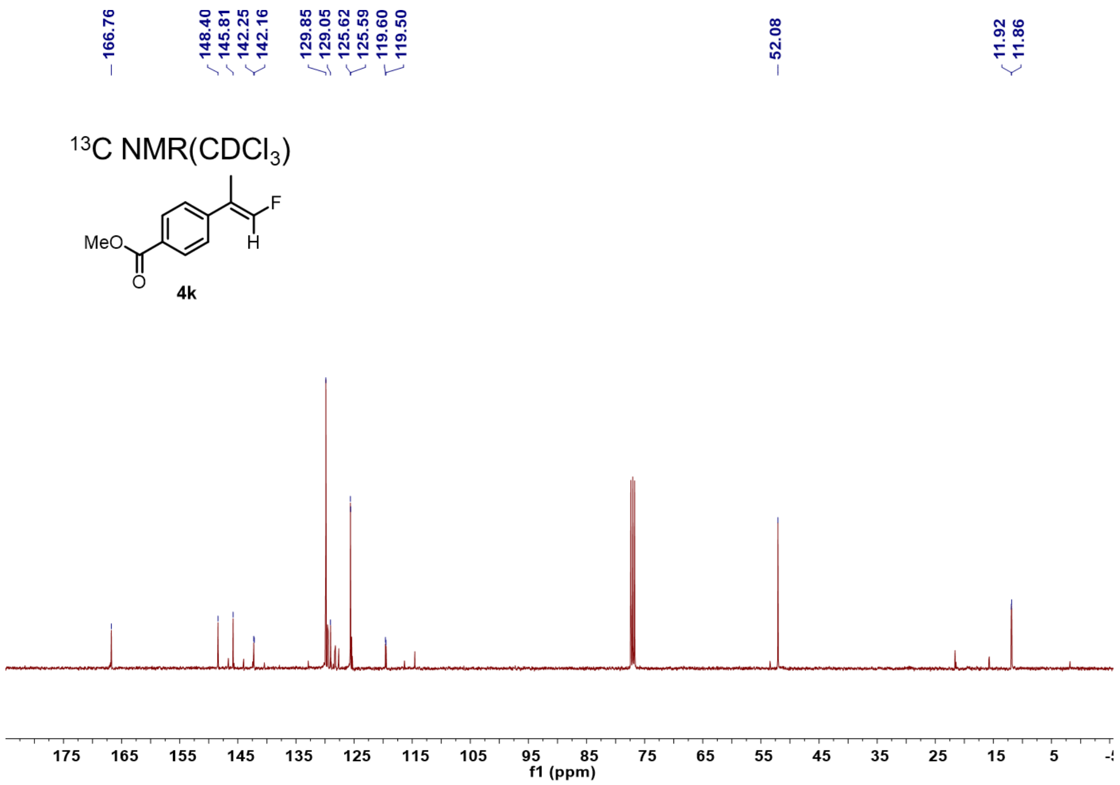

Supplementary Figure 140. ^{13}C NMR Spectrum of **4k**.

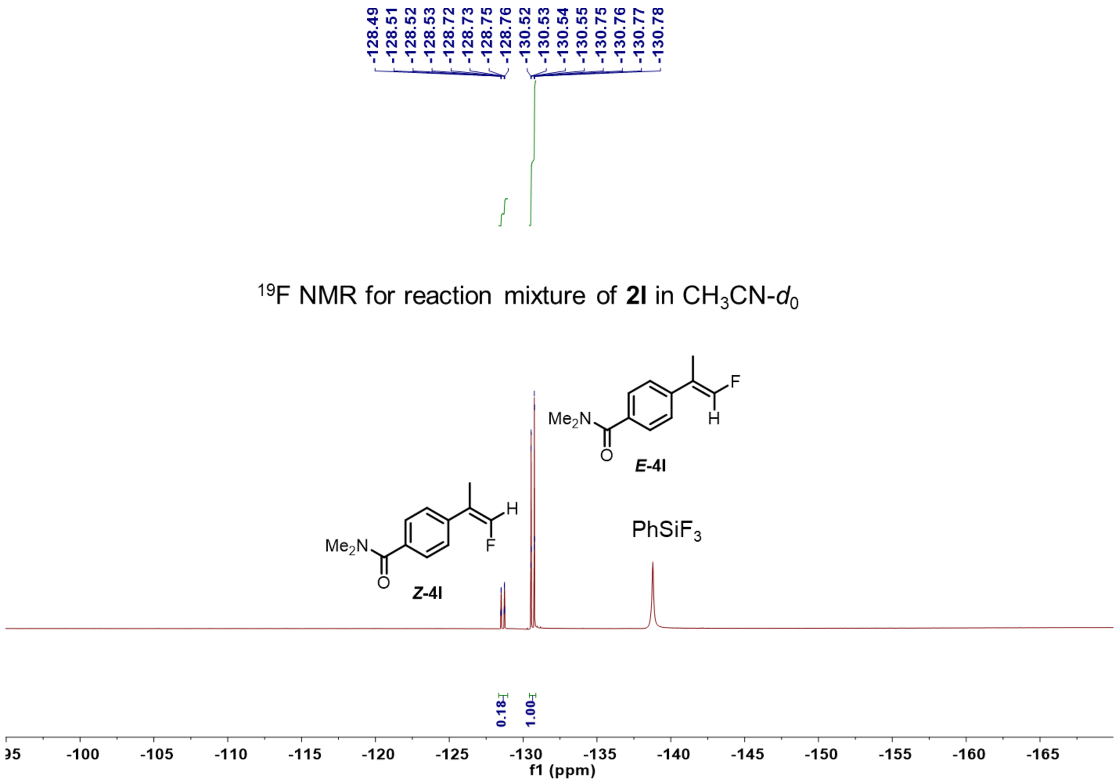

Supplementary Figure 141. ^{19}F NMR Spectrum for Reaction Mixture of **2l**.

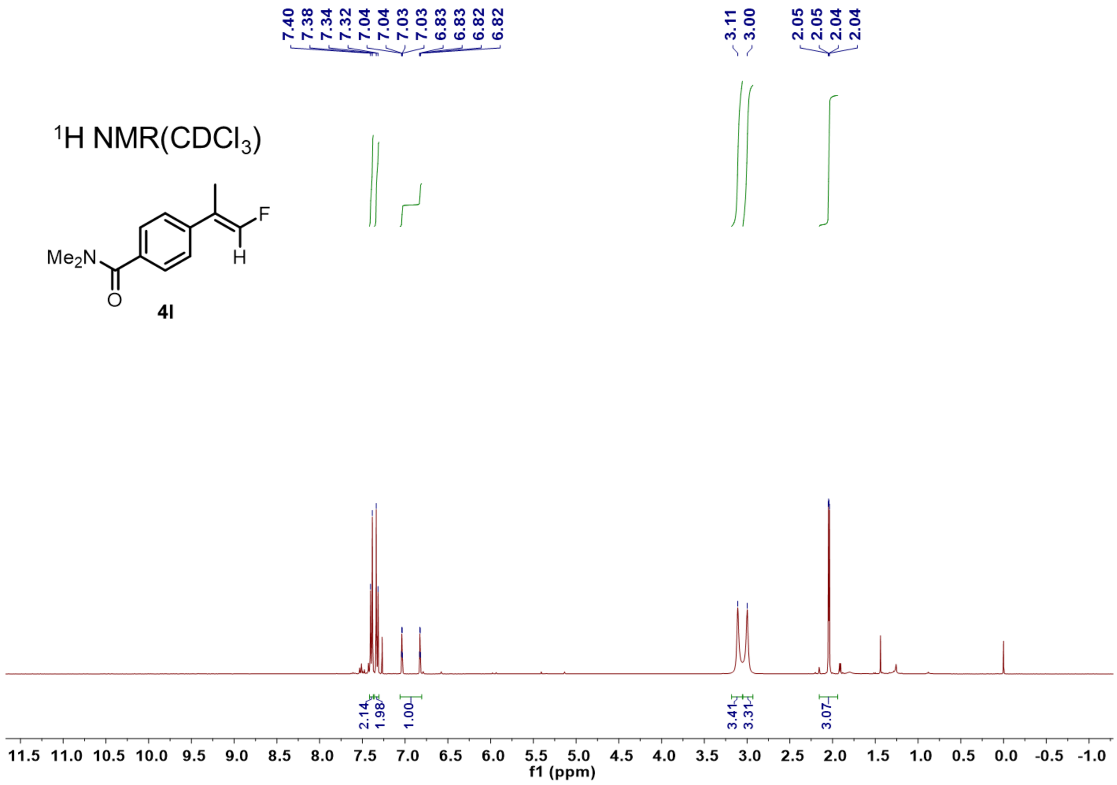

Supplementary Figure 142. ^{1}H NMR Spectrum of **4l**.

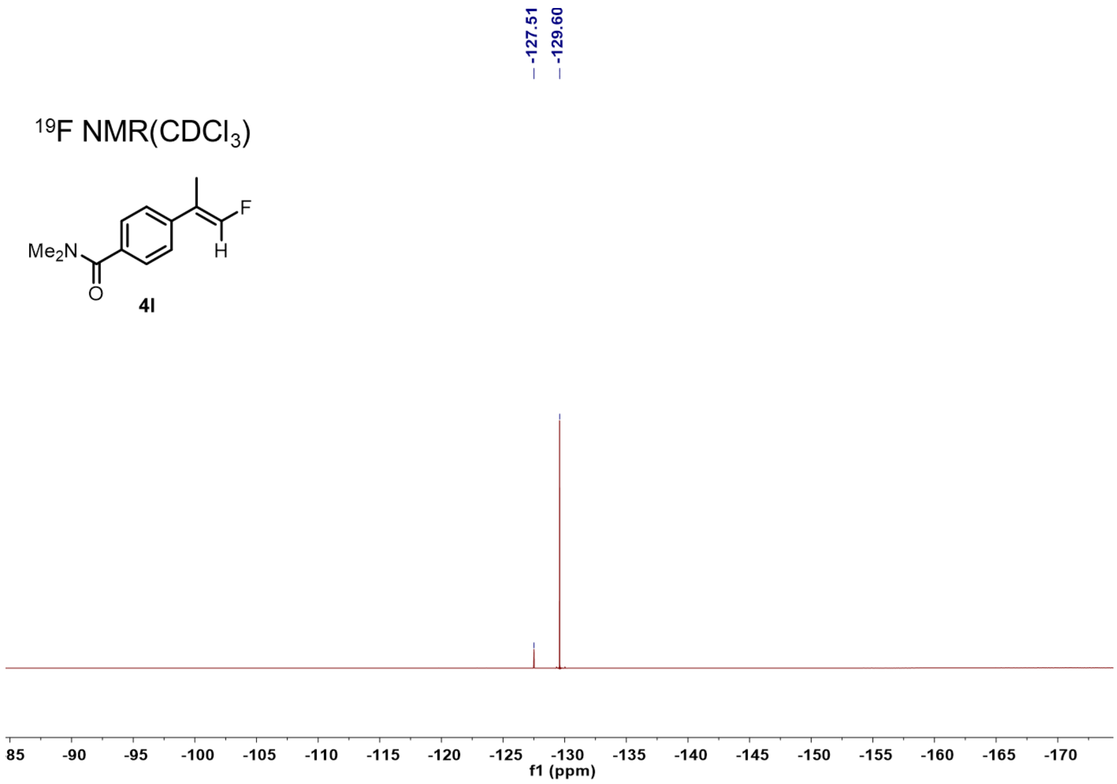

Supplementary Figure 143. ^{19}F NMR Spectrum of **4l**.

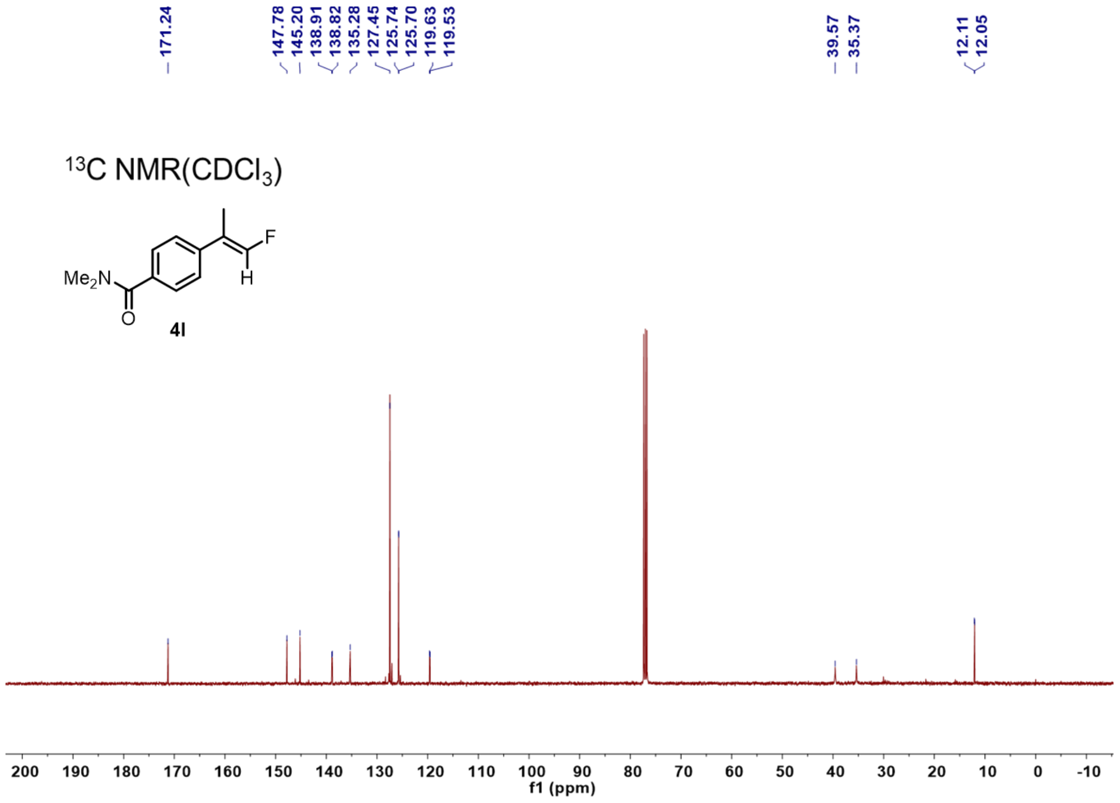

Supplementary Figure 144. ^{13}C NMR Spectrum of **4l**.

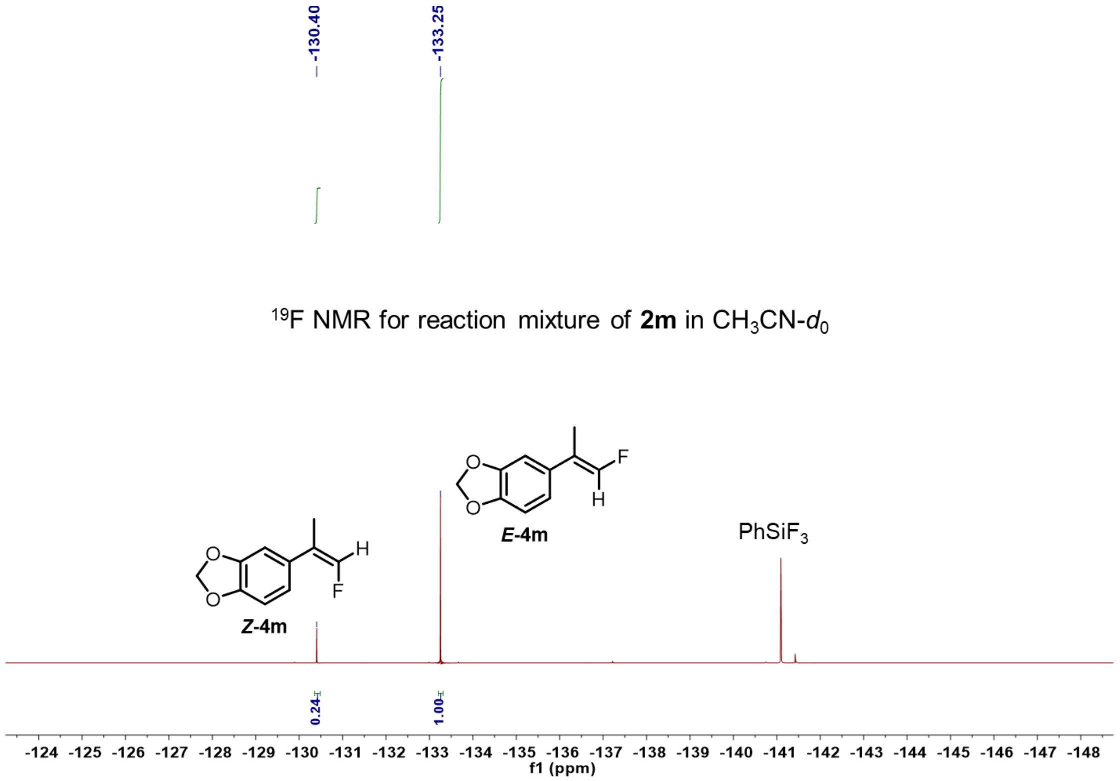

Supplementary Figure 145. ^{19}F NMR Spectrum for Reaction Mixture of **2m**.

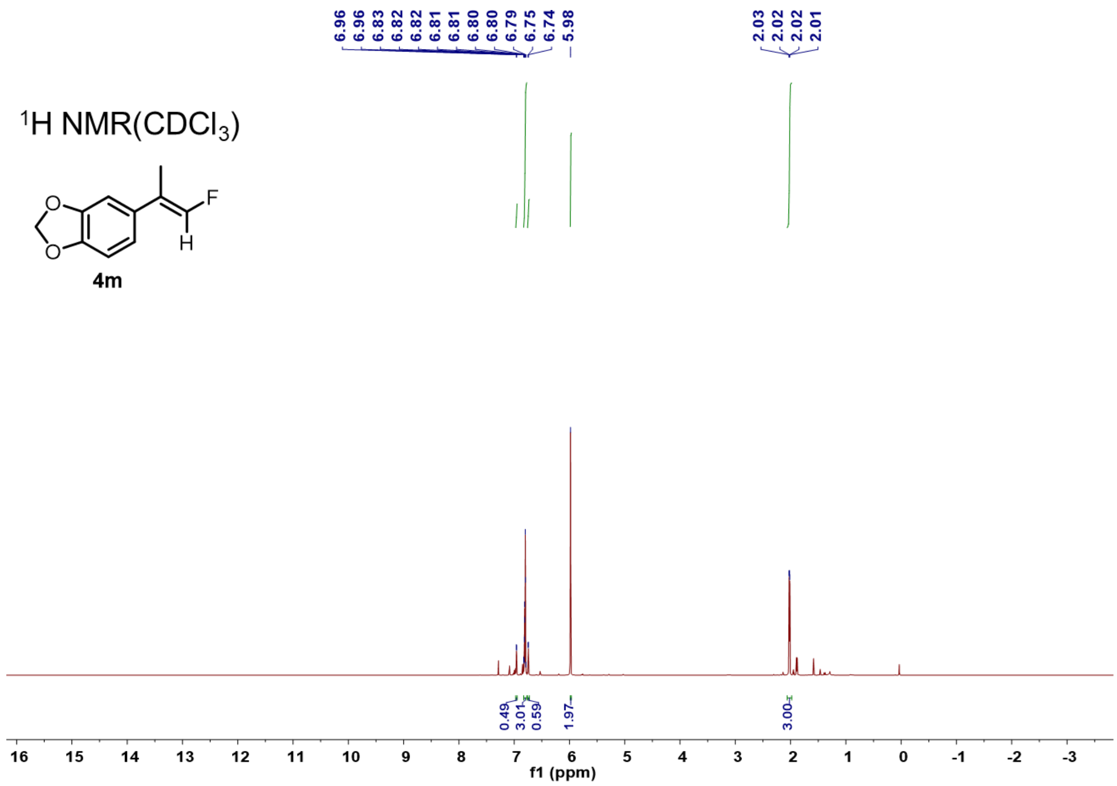

Supplementary Figure 146. ^{1}H NMR Spectrum of **4m**.

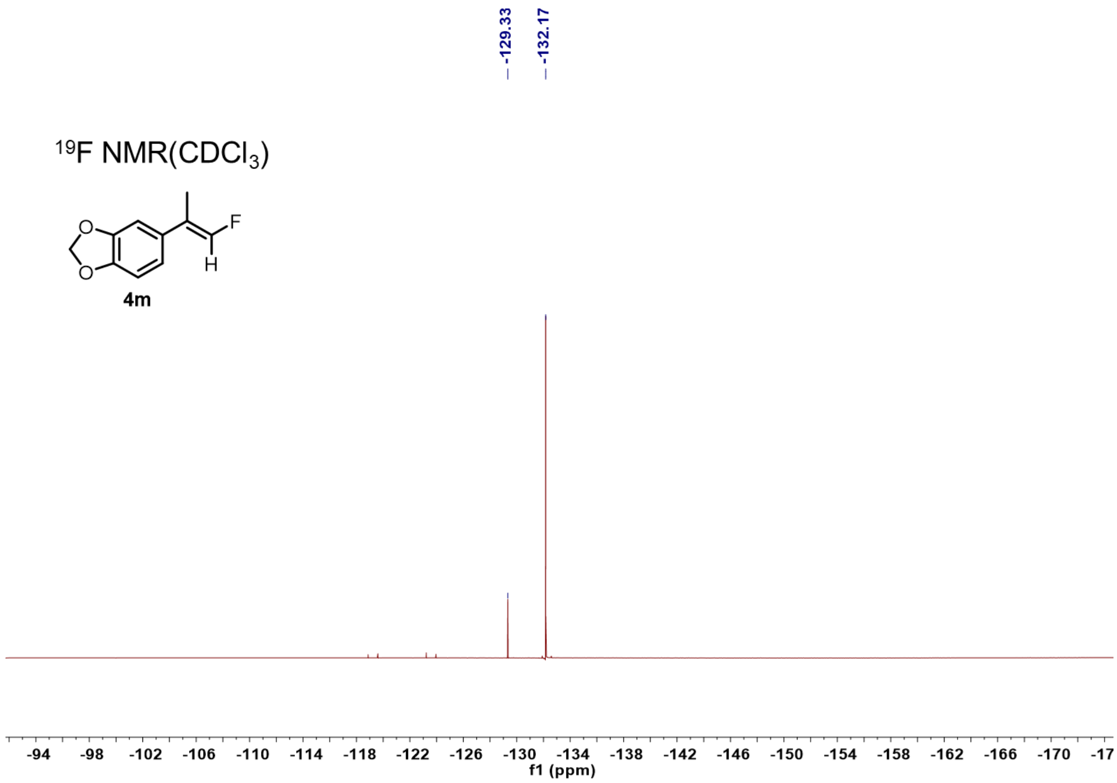

Supplementary Figure 147. ^{19}F NMR Spectrum of **4m**.

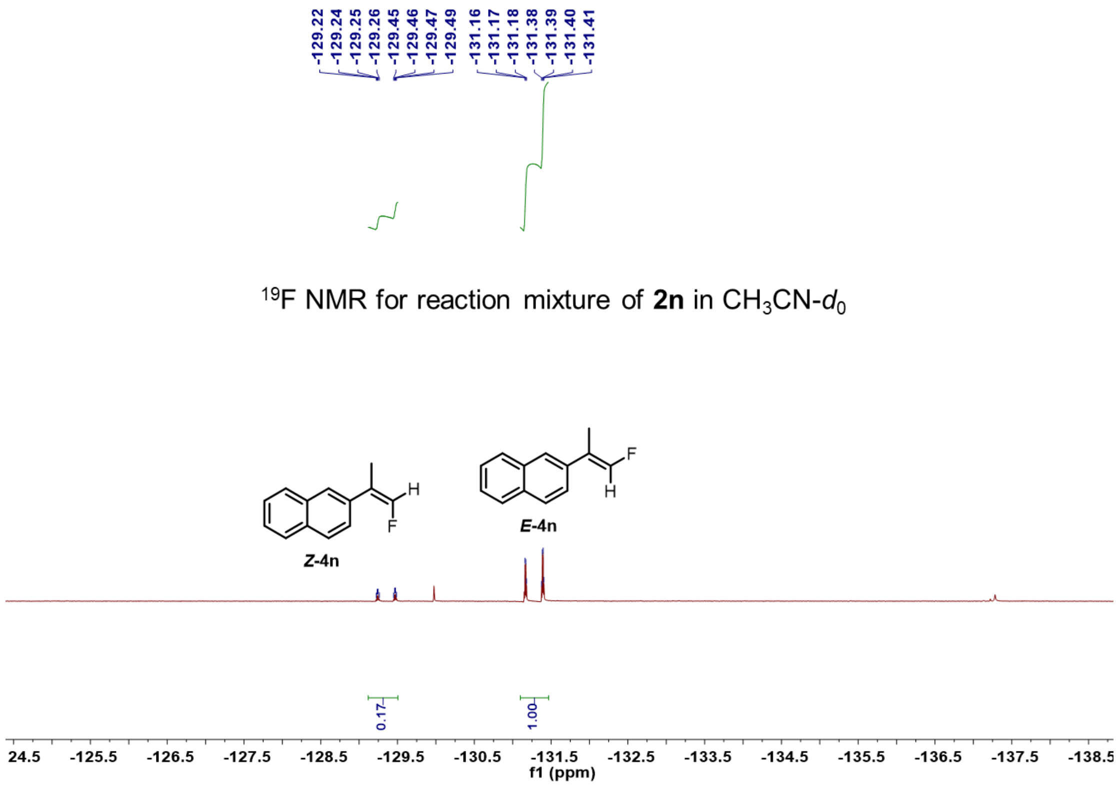

Supplementary Figure 148. ^{19}F NMR Spectrum for Reaction Mixture of **2n**.

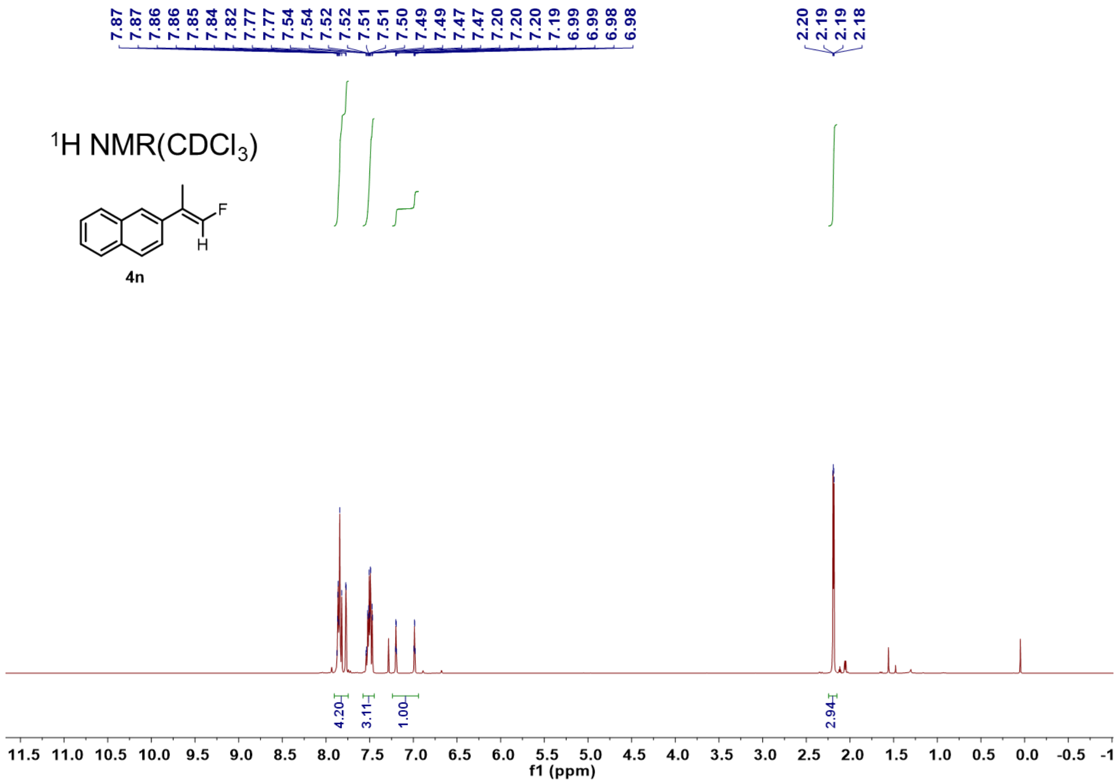

Supplementary Figure 149. ^{1}H NMR Spectrum of **4n**.

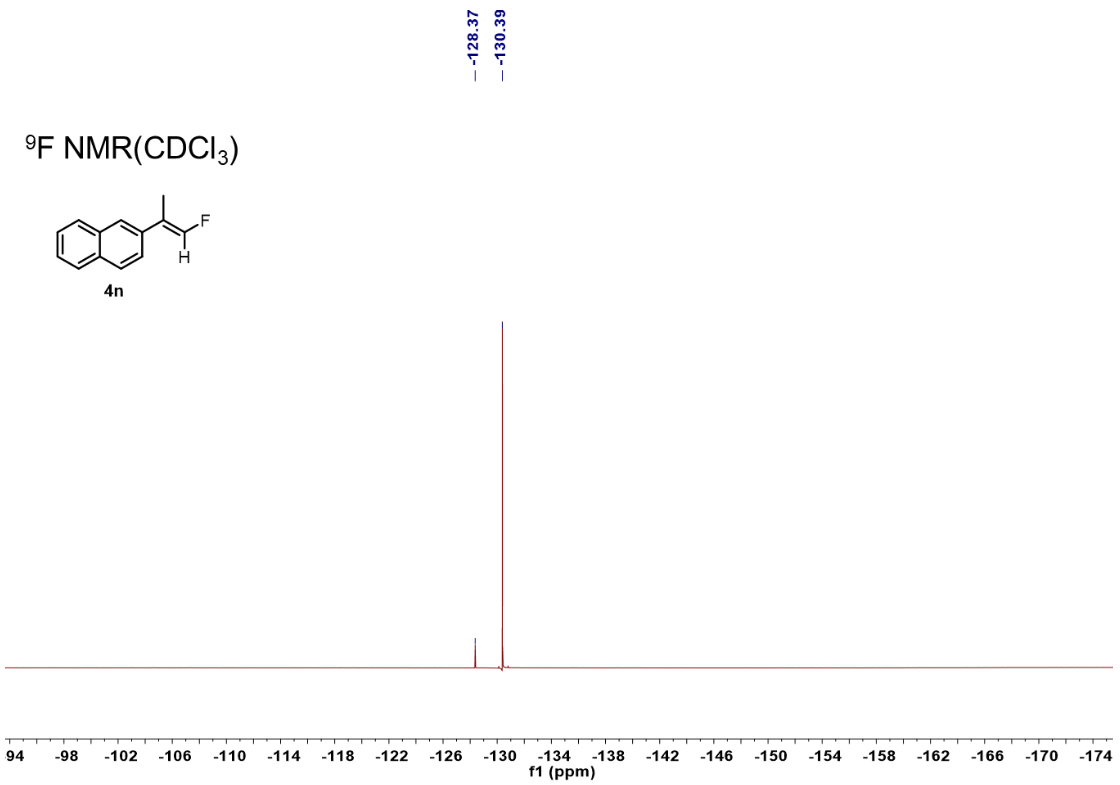

Supplementary Figure 150. ^{19}F NMR Spectrum of **4n**.

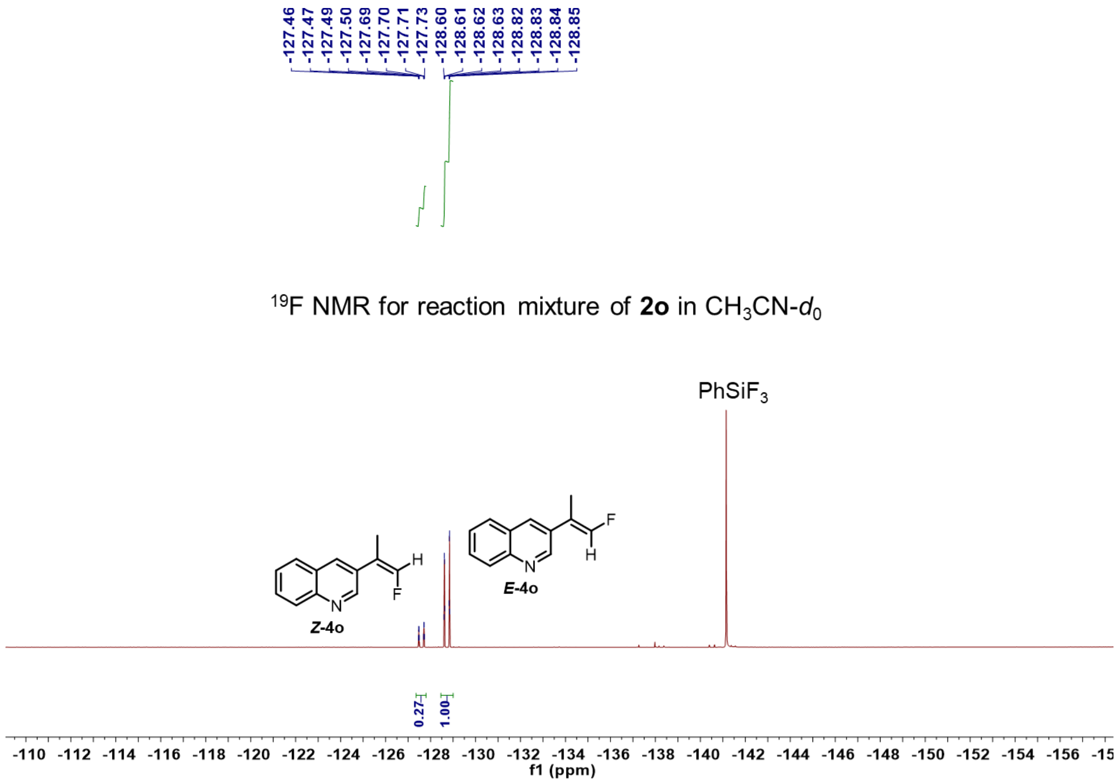

Supplementary Figure 151. ^{19}F NMR Spectrum for Reaction Mixture of **2o**.

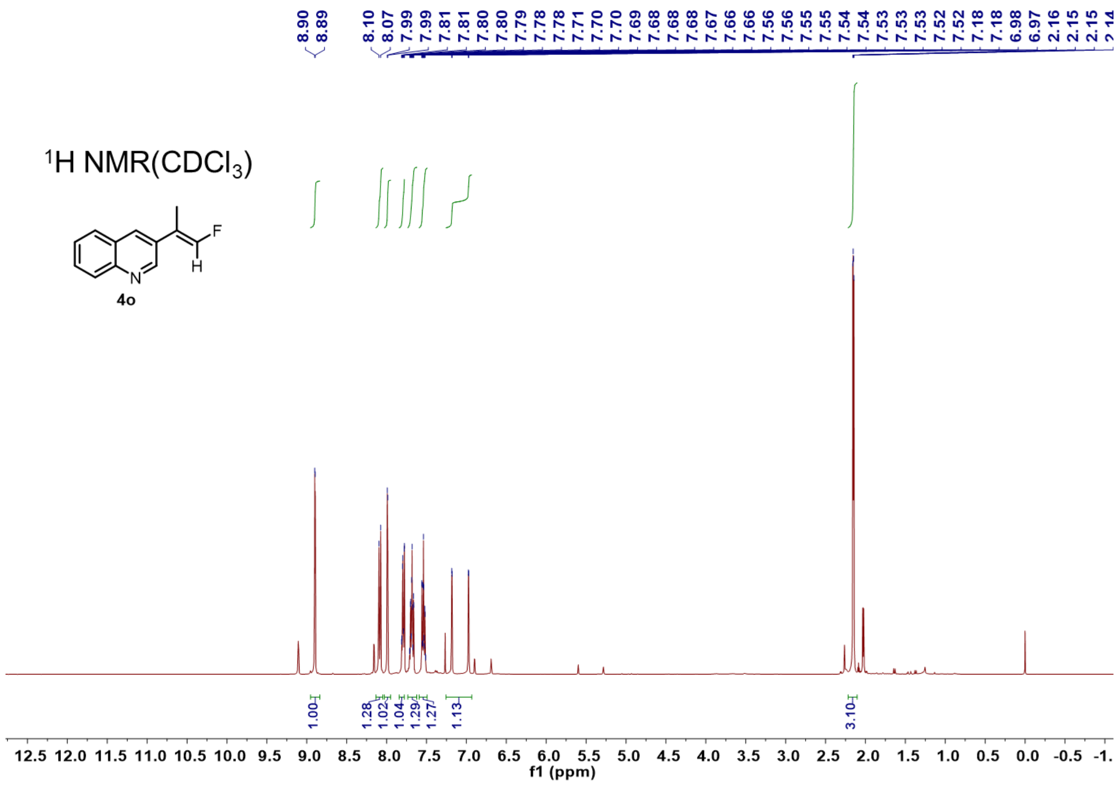

Supplementary Figure 152. ^{1}H NMR Spectrum of **4o**.

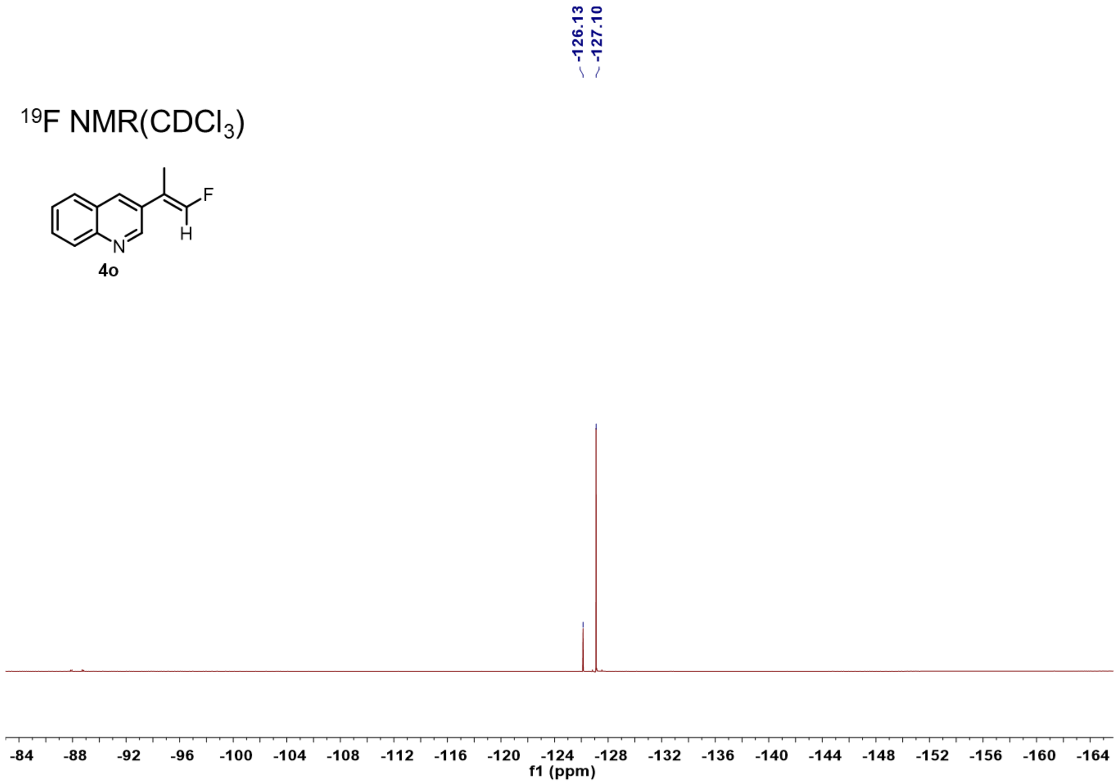

Supplementary Figure 153. ^{19}F NMR Spectrum of **4o**.

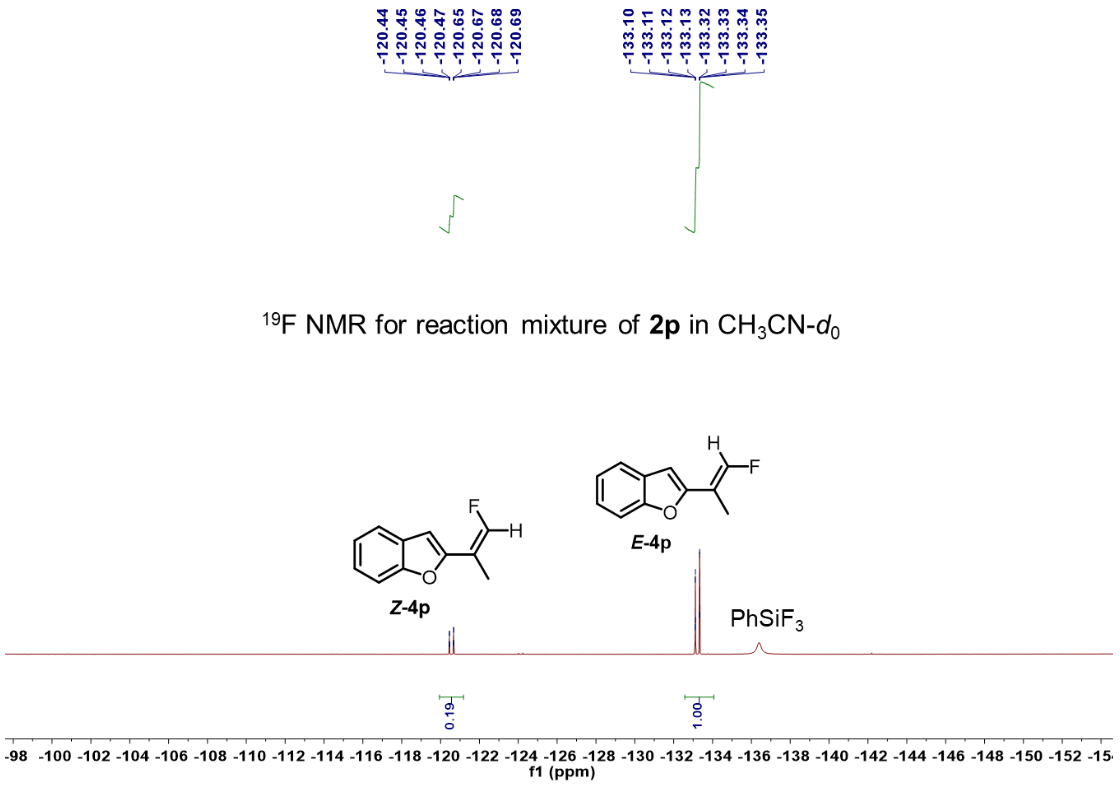

Supplementary Figure 154. ^{19}F NMR Spectrum for Reaction Mixture of **2p**.

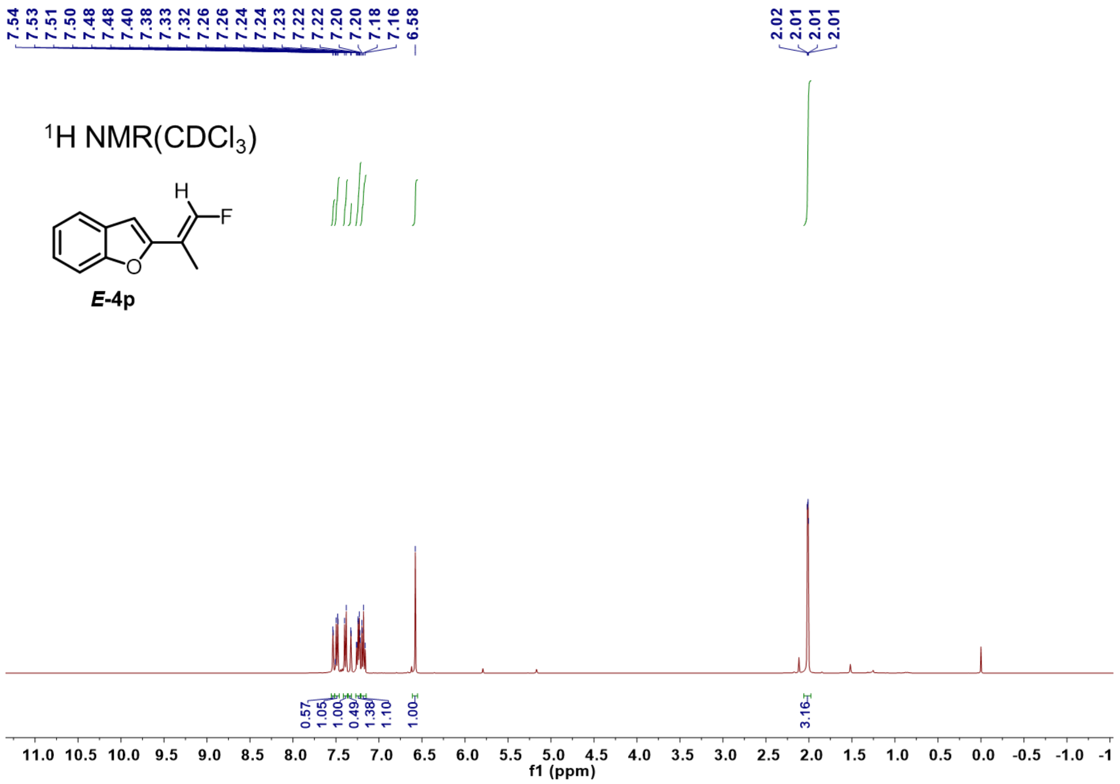

Supplementary Figure 155. ^{1}H NMR Spectrum of ***E*-4p**.

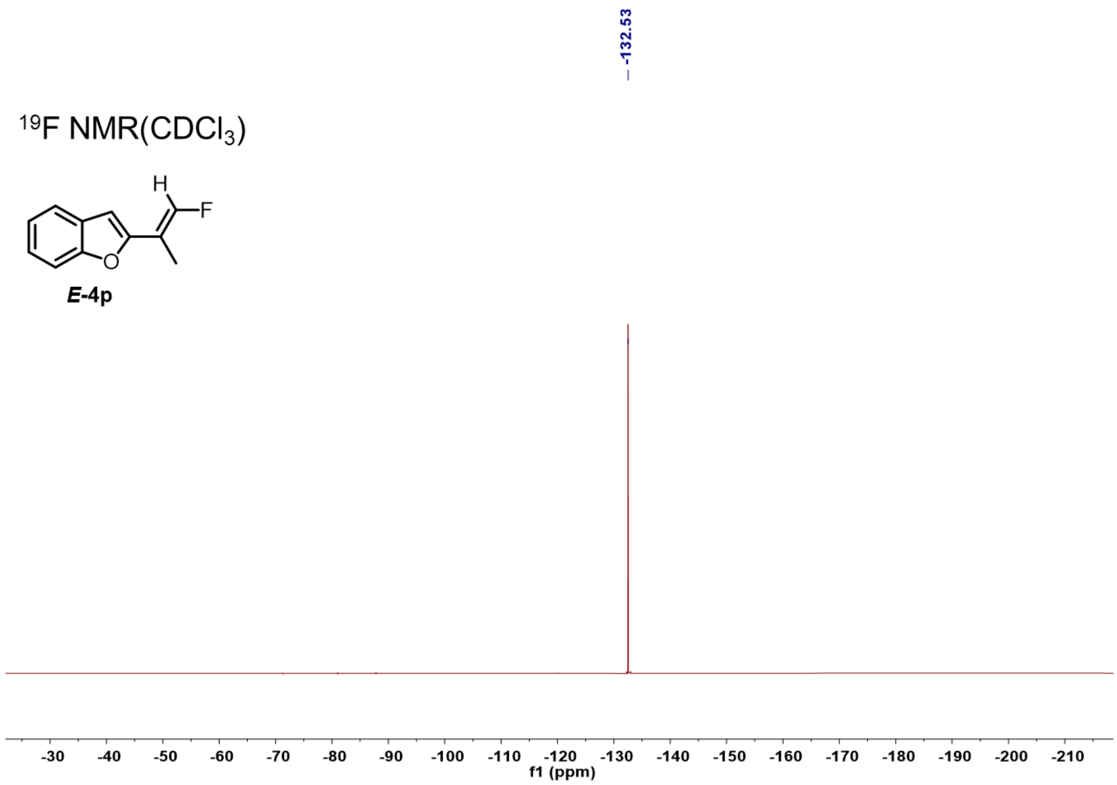

Supplementary Figure 156. ^{19}F NMR Spectrum of ***E*-4p**.

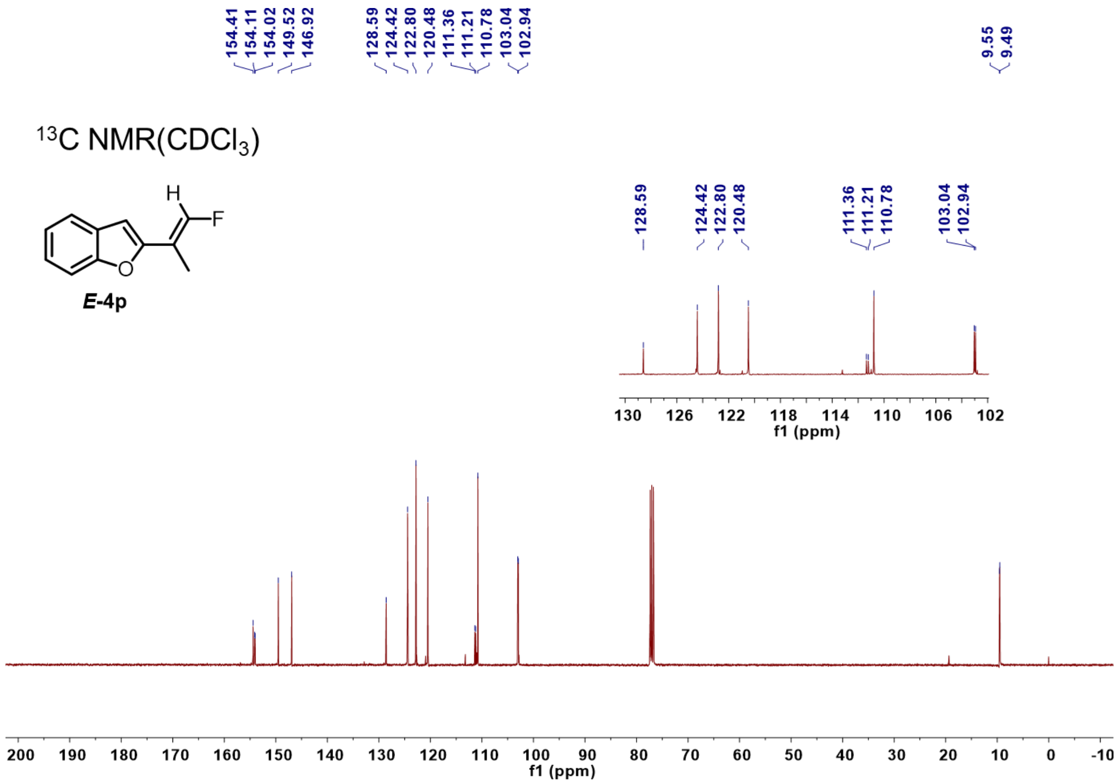

Supplementary Figure 157. ^{13}C NMR Spectrum of ***E*-4p**.

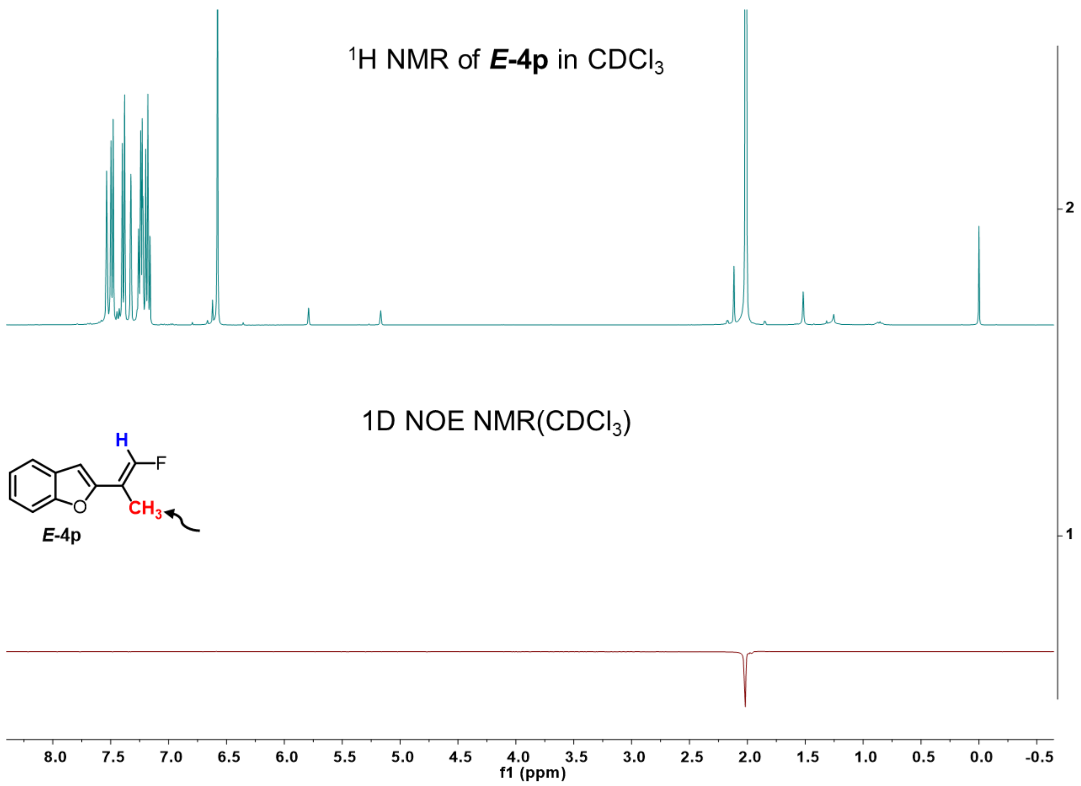

Supplementary Figure 158. 1D NOE Spectrum of ***E*-4p**.

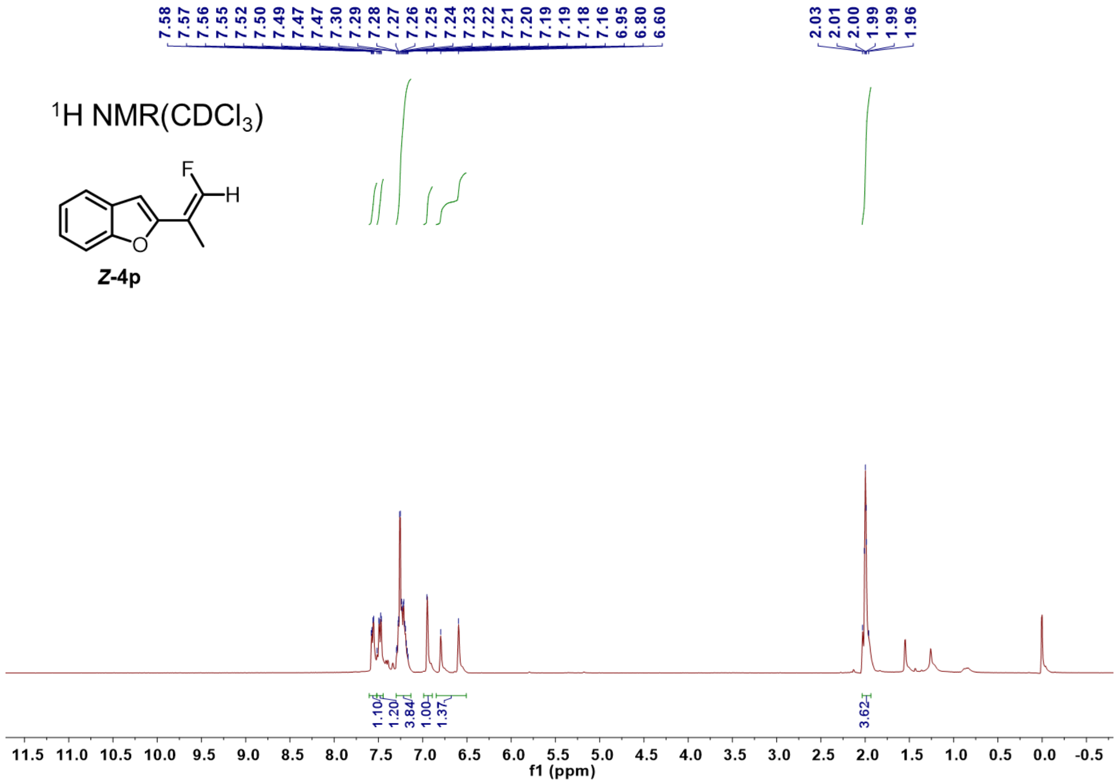

Supplementary Figure 159. ^{1}H NMR Spectrum of ***Z*-4p**.

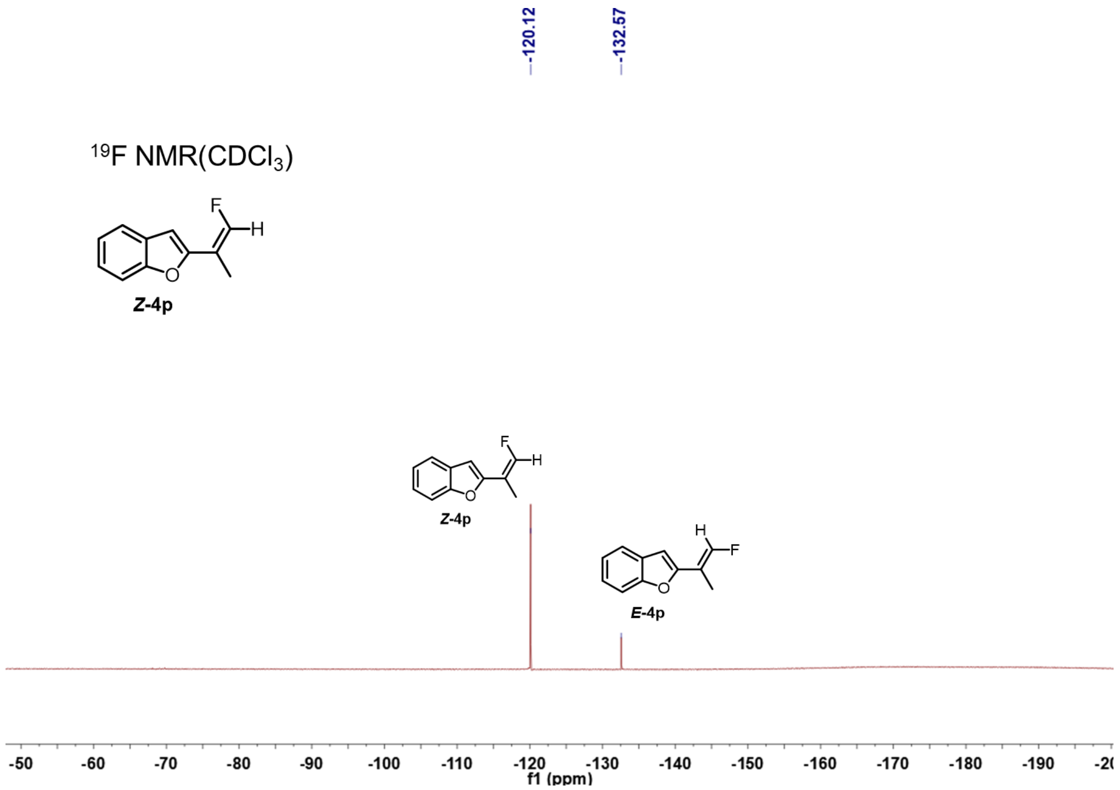

Supplementary Figure 160. ^{19}F NMR Spectrum of ***Z*-4p**.

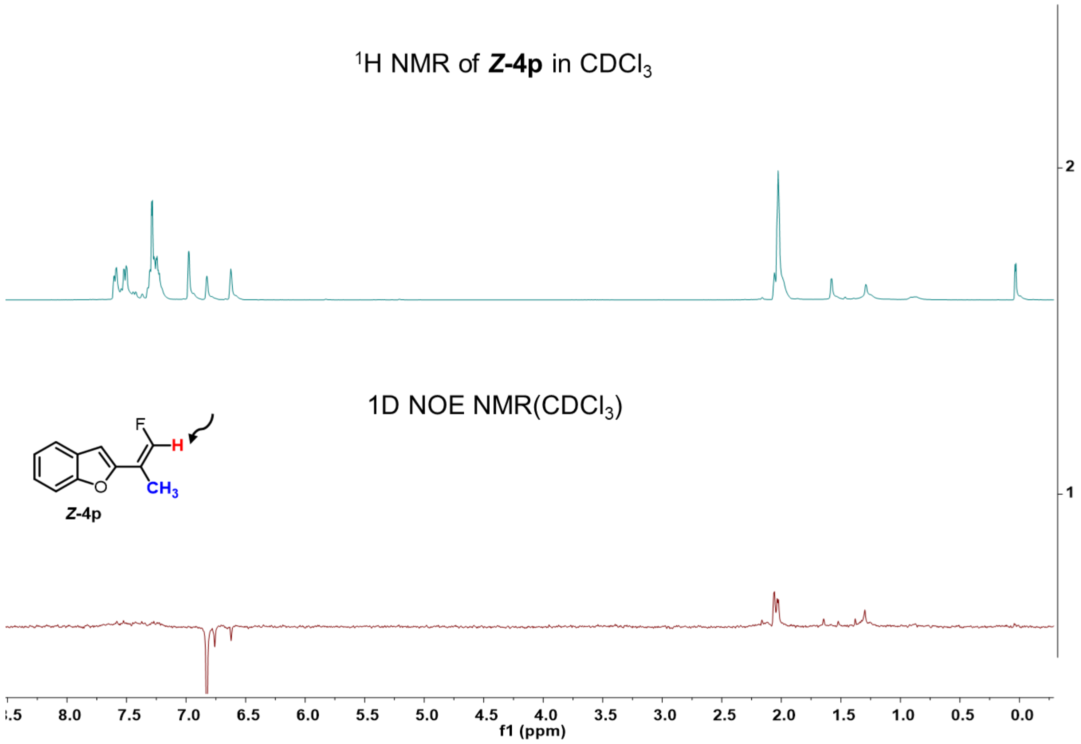

Supplementary Figure 161. 1D NOE Spectrum of ***Z*-4p**.

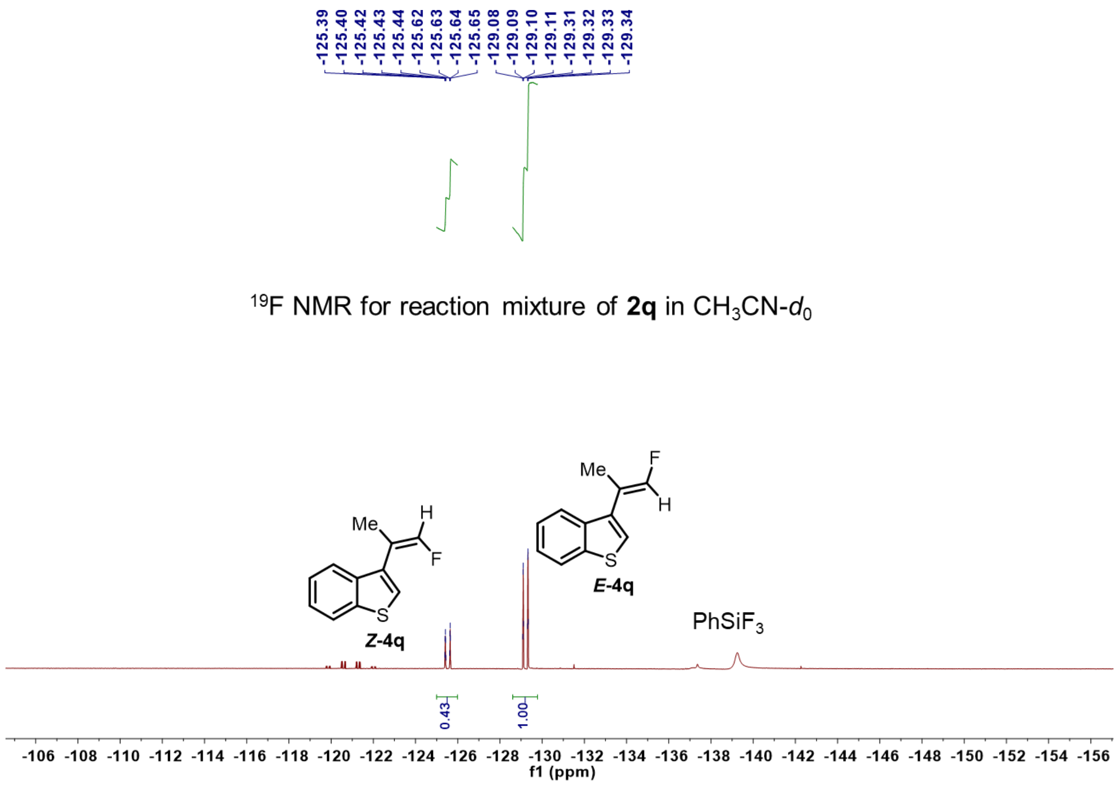

Supplementary Figure 162. ^{19}F NMR Spectrum for Reaction Mixture of **2q**.

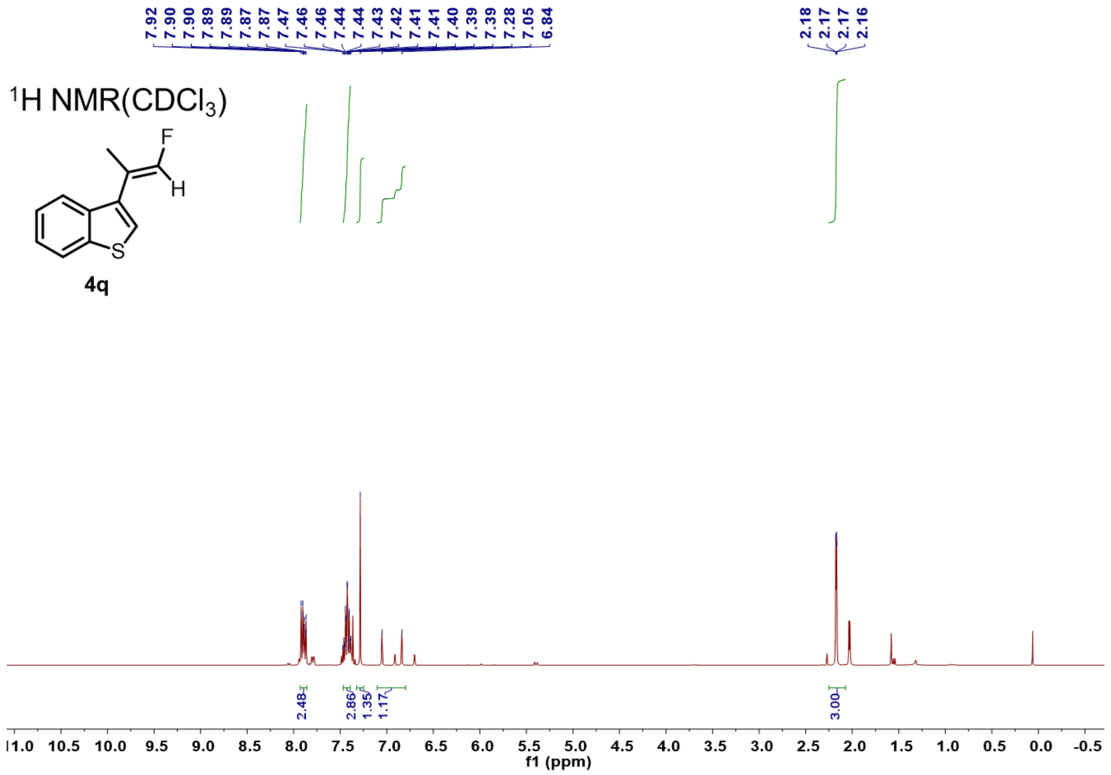

Supplementary Figure 163. ^{1}H NMR Spectrum for **4q**.

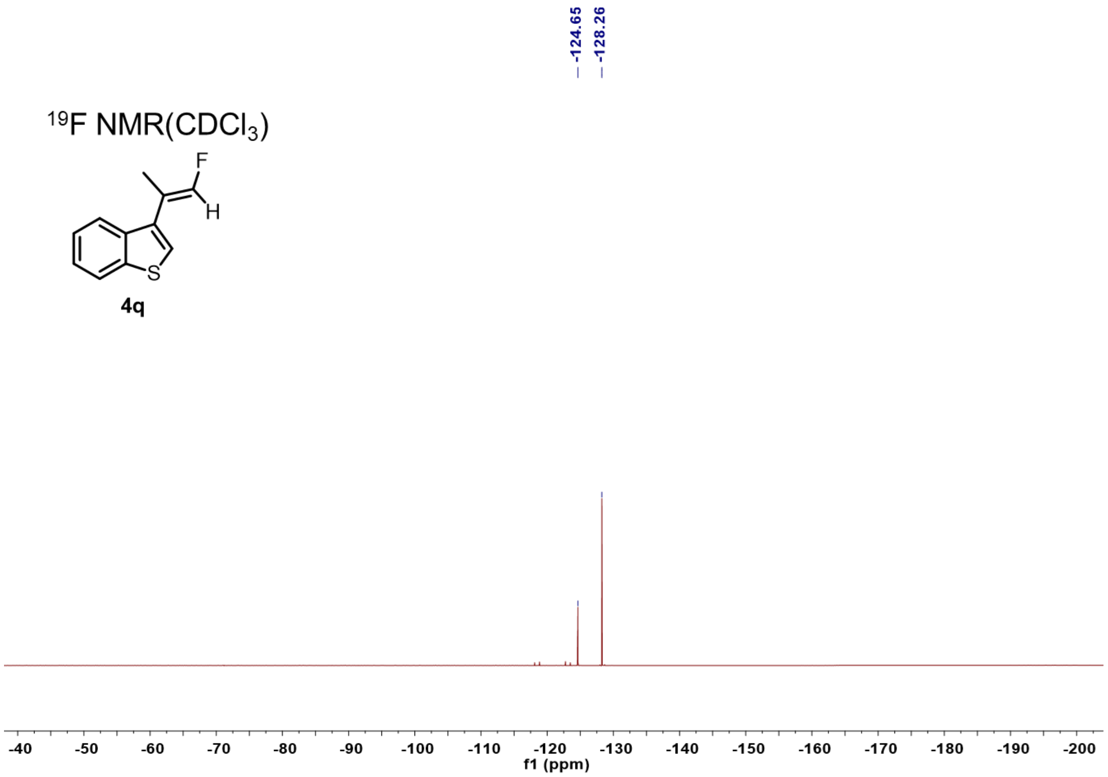

Supplementary Figure 164. ^{19}F NMR Spectrum for **4q**.

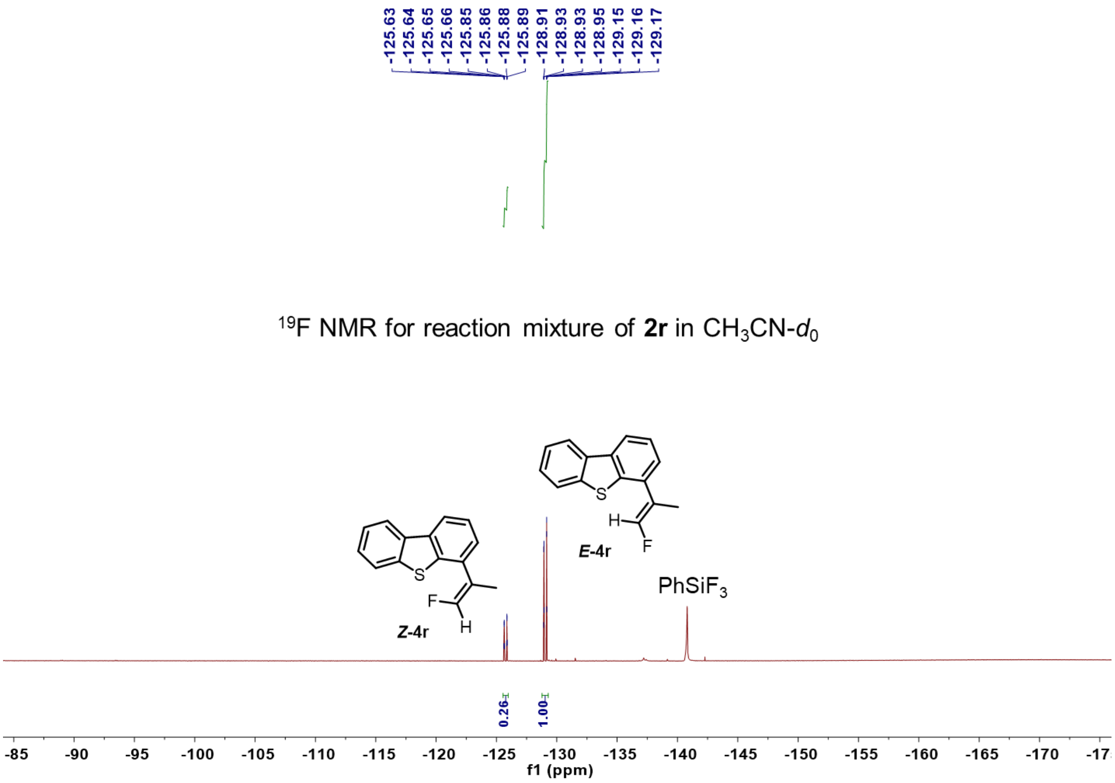

Supplementary Figure 165. ^{19}F NMR Spectrum for Reaction Mixture of **2r**.

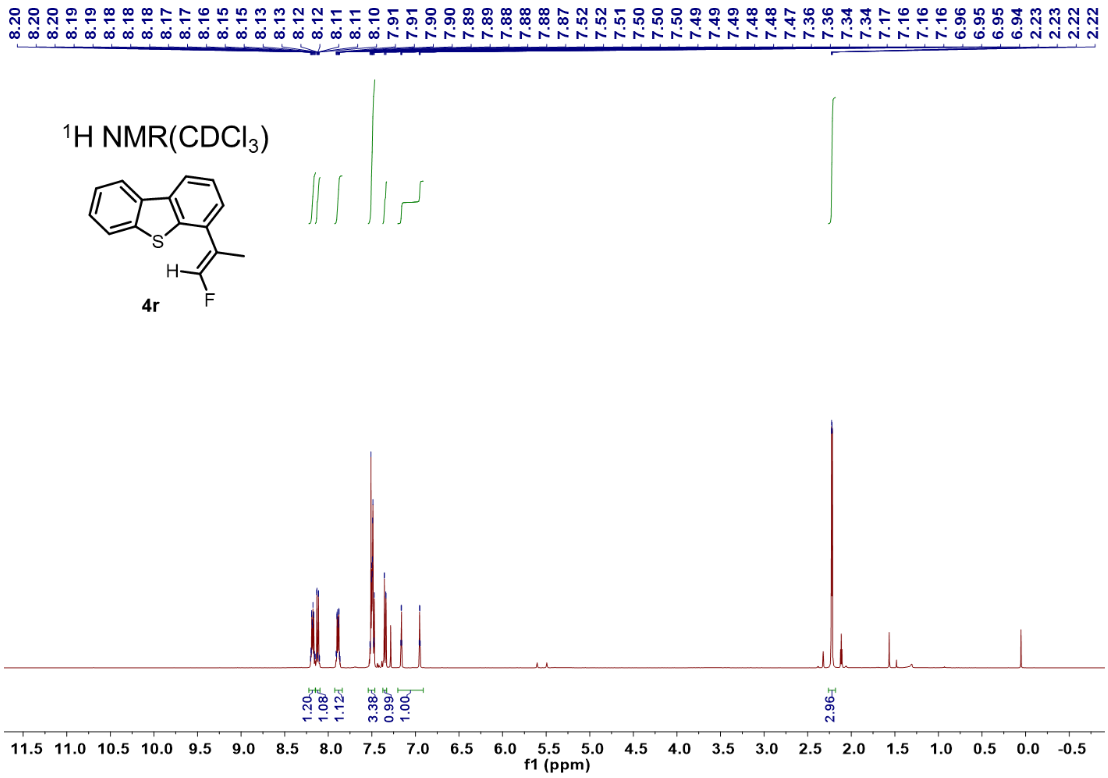

Supplementary Figure 166. ^{1}H NMR Spectrum for **4r**.

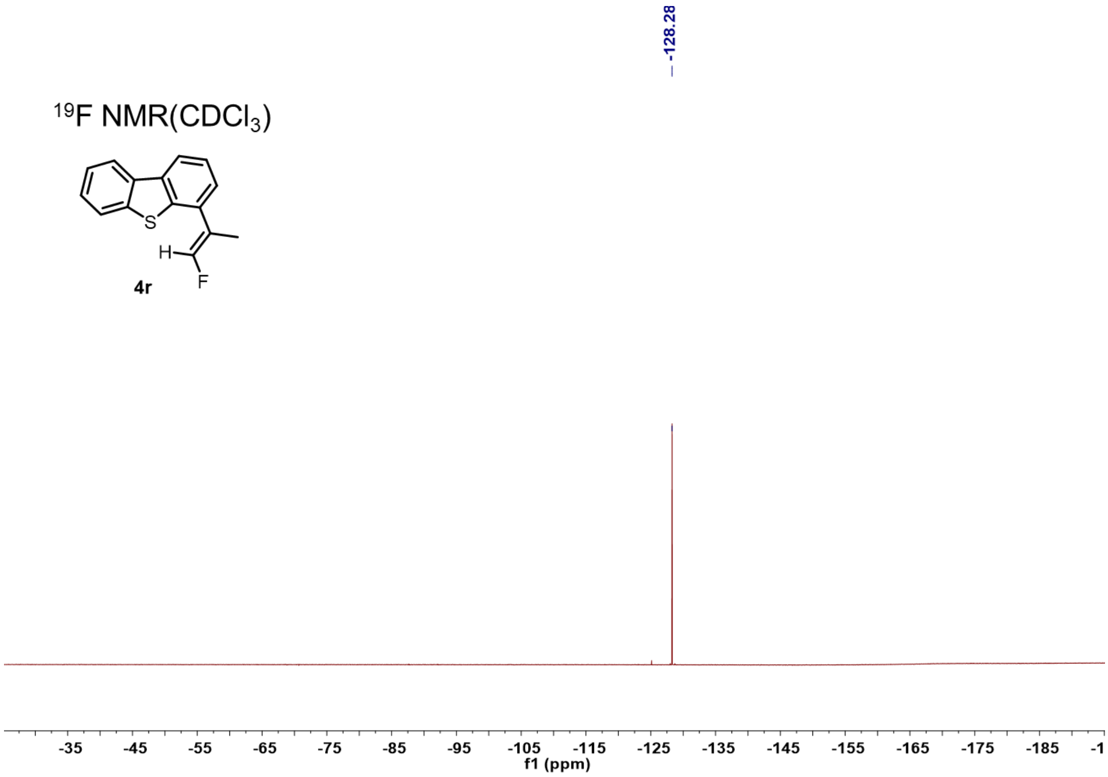

Supplementary Figure 167. ^{19}F NMR Spectrum for **4r**.

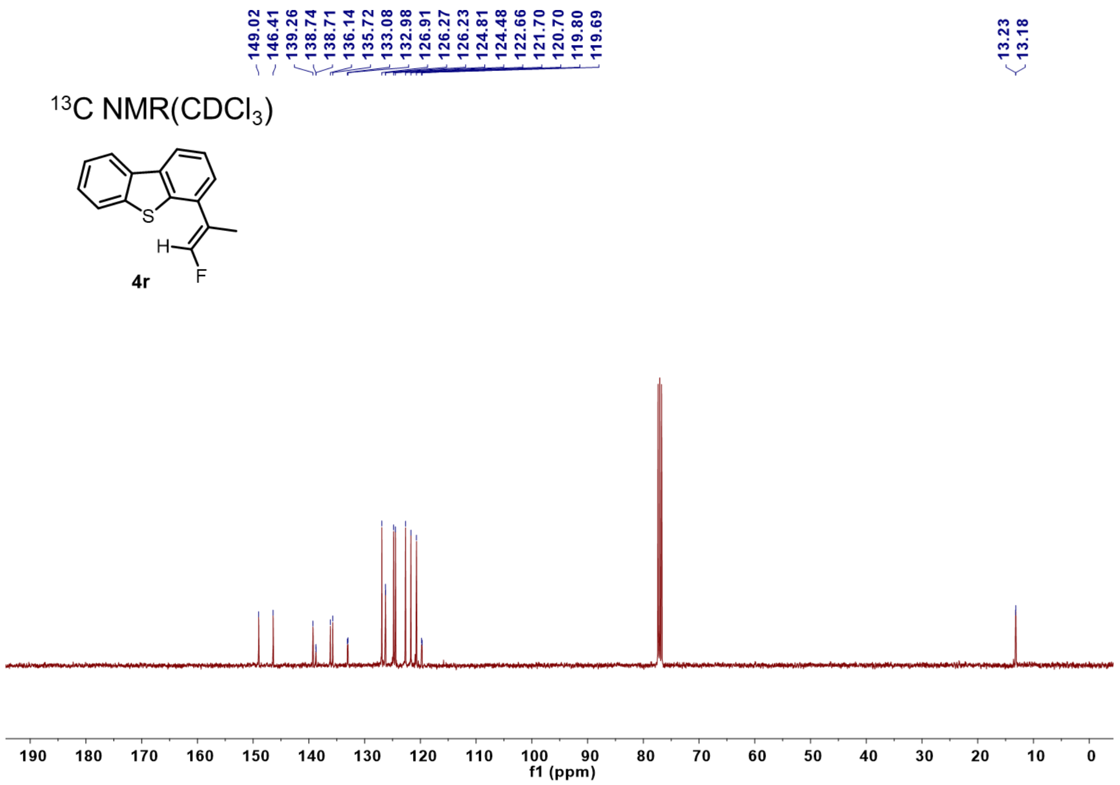

Supplementary Figure 168. ^{13}C NMR Spectrum for **4r**.

SMD-M06-2X/6-31+G(d) calculated cartesian coordinates and energies

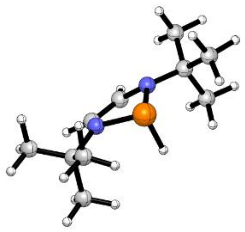

1

C	0.67199900	-1.47102800	-0.00052700
C	-0.67199100	-1.47103400	-0.00052900
H	1.30943400	-2.34009900	0.10508000
H	-1.30941700	-2.34011200	0.10507200
N	-1.22405600	-0.20749000	-0.31245700
N	1.22405500	-0.20748100	-0.31244300
C	-2.65439200	0.05669300	-0.02542700
C	-2.97999900	1.49828400	-0.41849200
C	-3.50562200	-0.89136400	-0.87487700
C	-2.95874100	-0.15121400	1.46344500
H	-2.47183900	2.22348900	0.22907800
H	-2.69463500	1.69650300	-1.45779900
H	-4.05713800	1.66650300	-0.31752900
H	-3.30982600	-1.94151500	-0.63538000
H	-4.56793600	-0.69989600	-0.68873300
H	-3.30376600	-0.73464600	-1.94032500

H	-4.00929500	0.08082200	1.67046500
H	-2.77419100	-1.18921300	1.76244000
H	-2.33513800	0.50065200	2.08589300
C	2.65439200	0.05669900	-0.02542800
C	3.50559400	-0.89142900	-0.87482400
C	2.98003800	1.49826100	-0.41856800
C	2.95873300	-0.15114300	1.46345500
H	3.30973700	-1.94156000	-0.63528900
H	3.30376600	-0.73474200	-1.94028200
H	4.56791400	-0.70000600	-0.68866500
H	2.47190000	2.22351500	0.22896500
H	4.05718100	1.66645700	-0.31761500
H	2.69467900	1.69643500	-1.45788500
H	4.00929400	0.08087400	1.67046600
H	2.33514700	0.50077400	2.08586700
H	2.77415300	-1.18912000	1.76250300
P	-0.00000500	0.99545600	-0.02788700
H	-0.00001500	0.95148400	1.43690700

Zero-point correction= 0.301634 (Hartree/Particle)

Thermal correction to Energy= 0.316973

Thermal correction to Enthalpy= 0.317917

Thermal correction to Gibbs Free Energy= 0.260728

Sum of electronic and zero-point Energies= -843.995832

Sum of electronic and thermal Energies= -843.980493

Sum of electronic and thermal Enthalpies= -843.979549

Sum of electronic and thermal Free Energies= -844.036738

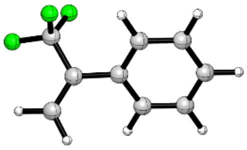

2c

C	2.96590700	0.88362600	0.32995800
C	1.62607200	1.25423800	0.25483600
C	0.64414300	0.32051000	-0.10692900
C	1.03466300	-1.00134400	-0.36610700
C	2.37666500	-1.37043300	-0.28712700
C	3.34729500	-0.43089800	0.05714100
H	3.71144600	1.62113500	0.61446800
H	1.33643900	2.27333400	0.49666500
H	0.30013800	-1.75098400	-0.64500400
H	2.66046200	-2.39753700	-0.49898200
H	4.39233700	-0.72139900	0.11874400
C	-0.77382600	0.75651100	-0.20702200
C	-1.16121100	1.98265500	-0.56744100

H	-0.43170200	2.73621400	-0.84914700
H	-2.20807800	2.26615900	-0.60062000
C	-1.83292700	-0.26564300	0.11585900
F	-1.97051100	-1.18857300	-0.86445900
F	-1.54895000	-0.95341900	1.24136400
F	-3.04961900	0.27507500	0.29252500

Zero-point correction= 0.140004 (Hartree/Particle)

Thermal correction to Energy= 0.149875

Thermal correction to Enthalpy= 0.150819

Thermal correction to Gibbs Free Energy= 0.103647

Sum of electronic and zero-point Energies= -646.335958

Sum of electronic and thermal Energies= -646.326087

Sum of electronic and thermal Enthalpies= -646.325142

Sum of electronic and thermal Free Energies= -646.372315

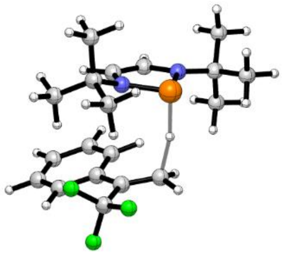

TS1

C	2.14510200	0.63940900	1.25611000
C	1.11425700	-0.08355900	1.73564000
H	2.56564200	1.53237600	1.70044200
H	0.57284500	0.10326200	2.65320700
N	0.77881400	-1.16228800	0.90954900
C	0.01955400	-2.33313300	1.41755600
C	-0.40610100	-3.19259600	0.22647200
C	0.91291300	-3.14484100	2.36047900
C	-1.22609800	-1.83943700	2.15814600
H	-0.94552600	-2.59236800	-0.51548600
H	0.45451000	-3.66383900	-0.26126800
H	-1.07226800	-3.99030400	0.57031900
H	1.22014800	-2.53779900	3.21970100
H	0.37558500	-4.02371100	2.73459300
H	1.81239600	-3.48471200	1.83403500
H	-1.85076500	-2.69811000	2.42618800
H	-0.97306300	-1.31553300	3.08542600
H	-1.81261600	-1.16692400	1.52355400
C	3.70135300	0.83664200	-0.70577900
C	4.91052900	1.12010500	0.18940300
C	4.13989300	-0.03528300	-1.88191100
C	3.10251700	2.14789600	-1.22917100
H	4.65218400	1.77171900	1.03035900

H	5.32049500	0.18476400	0.58567200
H	5.68897500	1.62230100	-0.39464500
H	3.31187100	-0.22972900	-2.57546100
H	4.92265000	0.48584500	-2.44177400
H	4.54444500	-0.99316300	-1.53663500
H	3.84669200	2.69258700	-1.82083400
H	2.23448700	1.94549900	-1.86707100
H	2.78648800	2.79330500	-0.40151900
C	-2.73771200	-0.57716600	-1.54391600
C	-1.66157400	0.40562000	-1.24595400
C	-1.86518500	1.35692900	-0.15080700
C	-3.09243900	1.48666600	0.53836000
C	-0.80814800	2.19818800	0.25439800
C	-3.25142800	2.42084700	1.55683500
H	-3.94100700	0.86322600	0.27473800
C	-0.97554800	3.13704400	1.27068400
H	0.16313200	2.11602600	-0.22445100
C	-2.19712500	3.25917500	1.93077200
H	-4.21216900	2.49788200	2.05969500
H	-0.13563200	3.76822300	1.55177200
H	-2.32736700	3.99053500	2.72322400
F	-3.17214300	-1.29176000	-0.47156100
F	-2.38129200	-1.49276300	-2.46870800
F	-3.86823000	0.01201600	-2.03520200
C	-0.54292500	0.41181600	-2.05763200
H	0.05928800	1.31571200	-2.11962500
H	-0.52678400	-0.20375900	-2.95294300
P	1.70064000	-1.16080900	-0.53692000
N	2.67793000	0.10888600	0.07782600
H	0.66253600	-0.33074100	-1.41791300

Zero-point correction= 0.440653 (Hartree/Particle)

Thermal correction to Energy= 0.466991

Thermal correction to Enthalpy= 0.467935

Thermal correction to Gibbs Free Energy= 0.384772

Sum of electronic and zero-point Energies= -1490.324394

Sum of electronic and thermal Energies= -1490.298056

Sum of electronic and thermal Enthalpies= -1490.297112

Sum of electronic and thermal Free Energies= -1490.380275

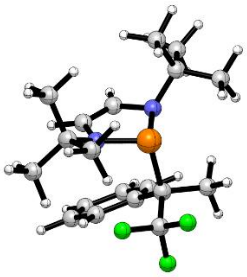

A

C	-2.63071500	-2.07951400	-2.63153700
C	-2.80307600	-0.74197200	-2.27534100
C	-2.28879000	-0.25595100	-1.07515700
C	-1.58995700	-1.09635500	-0.19095500
C	-1.44883900	-2.44481800	-0.55184500
C	-1.95532300	-2.92932200	-1.75780900
H	-3.02479000	-2.45496600	-3.57192900
H	-3.33385700	-0.06398100	-2.93906200
H	-2.41638900	0.79571600	-0.84987100
H	-0.92708800	-3.13709800	0.09950600
H	-1.81859100	-3.97774100	-2.00989500
C	-0.94159200	-0.57570600	1.07939700
C	-1.85794300	0.39184700	1.81290000
C	-0.58609600	-1.68826500	2.07789900
H	-0.14339300	-1.27461000	2.98858600
H	-1.47438900	-2.26667600	2.35539400
H	0.14384000	-2.36973900	1.63927400
F	-2.09093200	1.54901300	1.15536100
F	-3.07427600	-0.14270200	2.06775100
F	-1.35723900	0.76158100	3.01262400
C	0.71107600	0.45339400	-1.78237000
C	1.31934900	-0.70398200	-1.48876500
N	1.55720000	-0.91739300	-0.09912100
N	0.42172500	1.26122800	-0.66071200
P	0.74918200	0.36064900	0.76823800
C	0.51338800	2.74251300	-0.79835700
C	0.56119700	3.40058500	0.58041700
C	1.78473300	3.12009600	-1.56751200
C	-0.72595400	3.23260700	-1.55287000
H	-0.28902600	3.10453900	1.20346500
H	1.48715700	3.15590300	1.11222800
H	0.52254000	4.48738100	0.45203100
H	1.76116500	2.74335800	-2.59565600
H	1.88566700	4.21022400	-1.61002600
H	2.67044500	2.70914100	-1.06815800
H	-0.65421900	4.31072300	-1.73657000
H	-0.82477000	2.73130200	-2.52246500

H	-1.63256900	3.03977000	-0.96919700
C	2.95399400	-1.30119700	0.28923600
C	3.37969700	-2.50353200	-0.55807600
C	3.91995800	-0.13366600	0.05717600
C	2.97392200	-1.71270000	1.76091300
H	2.62468700	-3.29706900	-0.50939700
H	3.54132500	-2.23510800	-1.60673500
H	4.32355800	-2.90092200	-0.17040800
H	3.68455200	0.70837800	0.72054600
H	4.95245100	-0.44311500	0.25675700
H	3.86234100	0.21327100	-0.98137100
H	4.00124800	-1.95341400	2.05478200
H	2.61998100	-0.90781800	2.41502400
H	2.35273900	-2.59862700	1.92926700
H	0.40286000	0.77360300	-2.77218500
H	1.58016300	-1.47441700	-2.20435500

Zero-point correction= 0.447470 (Hartree/Particle)

Thermal correction to Energy= 0.473285

Thermal correction to Enthalpy= 0.474229

Thermal correction to Gibbs Free Energy= 0.393899

Sum of electronic and zero-point Energies= -1490.379673

Sum of electronic and thermal Energies= -1490.353858

Sum of electronic and thermal Enthalpies= -1490.352913

Sum of electronic and thermal Free Energies= -1490.433244

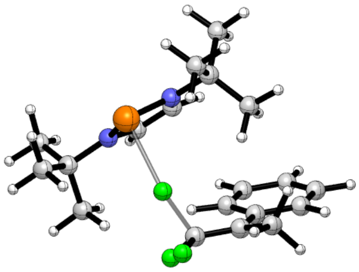

TS2

C	-2.86829500	0.64104700	3.04985000
C	-1.70135100	-0.05084300	2.71067600
C	-1.58438000	-0.70716600	1.48983200
C	-2.64107800	-0.71254800	0.54618900
C	-3.80750700	0.00227800	0.90658500
C	-3.91793600	0.66044800	2.13074000
H	-2.95296100	1.15548500	4.00302500
H	-0.85927300	-0.06487600	3.40002400
H	-0.64326500	-1.19425800	1.25031300
H	-4.64455400	0.04348900	0.21502200
H	-4.83549300	1.19602800	2.36425600
C	-2.54910300	-1.33177300	-0.76920800
C	-1.47485900	-2.12452000	-1.09706900

C	-3.48155200	-0.87773400	-1.86794800
H	-3.16700400	-1.26352700	-2.84120800
H	-4.51545100	-1.21407200	-1.70865300
H	-3.51557000	0.21952300	-1.94537500
F	-0.90421300	-2.93006000	-0.17569000
F	-1.49112200	-2.79235300	-2.26441000
F	-0.00273600	-1.26731800	-1.42025600
C	1.89711400	0.70887300	1.49229000
C	1.02976800	1.66818300	1.07073000
N	0.98267800	1.73982000	-0.29873300
N	2.49296000	0.06806400	0.43421600
P	1.97462100	0.62420700	-1.05784800
C	3.46141800	-1.05935700	0.63692800
C	3.94982000	-1.54636700	-0.72482800
C	4.63901300	-0.53447800	1.45849400
C	2.72769300	-2.18616600	1.36510700
H	3.12537800	-1.93035500	-1.33729200
H	4.47246100	-0.75435100	-1.27361200
H	4.65652400	-2.36620000	-0.56712100
H	4.32454000	-0.20507600	2.45372800
H	5.37233000	-1.33722400	1.58446500
H	5.12324700	0.30267500	0.94468200
H	3.41664700	-3.02422900	1.51093600
H	2.37049200	-1.86449000	2.34871400
H	1.87390900	-2.53333900	0.77401500
C	0.06444800	2.69118600	-1.00875800
C	-1.37068100	2.30127600	-0.65324700
C	0.39802500	4.11050600	-0.55016400
C	0.27979800	2.56399000	-2.51438900
H	-1.57172400	1.27333400	-0.97335700
H	-1.55274700	2.37676600	0.42513200
H	-2.06514200	2.97467500	-1.16668600
H	1.44575500	4.34702800	-0.76434400
H	-0.23750800	4.81746500	-1.09270000
H	0.21556900	4.24558800	0.52032600
H	-0.39120300	3.26552600	-3.01847700
H	1.30724700	2.81626200	-2.80105800
H	0.03792400	1.55716800	-2.87503600
H	2.12321800	0.43655900	2.51434300
H	0.41837100	2.31268200	1.68761400

Zero-point correction= 0.443837 (Hartree/Particle)

Thermal correction to Energy= 0.471168

Thermal correction to Enthalpy= 0.472112

Thermal correction to Gibbs Free Energy= 0.386131

Sum of electronic and zero-point Energies= -1490.342784

Sum of electronic and thermal Energies= -1490.315453

Sum of electronic and thermal Enthalpies= -1490.314509

Sum of electronic and thermal Free Energies= -1490.400490

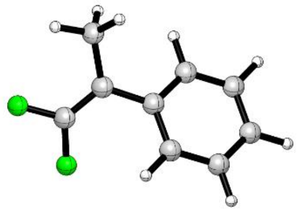

3c

C	2.67882600	0.92277300	-0.45918800
C	1.31061200	1.18321900	-0.40151300
C	0.41523300	0.20933800	0.06433100
C	0.92882700	-1.02331700	0.49454100
C	2.29686200	-1.28162100	0.43689000
C	3.17768500	-0.31163600	-0.04329000
H	3.35566500	1.68782500	-0.82972000
H	0.93705000	2.14920100	-0.73002000
H	0.25934500	-1.77835800	0.89606900
H	2.67528500	-2.24060800	0.78015600
H	4.24444700	-0.51346600	-0.08433100
C	-1.03455200	0.52560600	0.13566700
C	-1.94990800	-0.39738400	-0.13051800
C	-1.46789400	1.92521900	0.49316500
H	-1.25663100	2.62054200	-0.32642500
H	-2.53754800	1.97336900	0.70705100
H	-0.92085300	2.27202800	1.37564000
F	-1.72221600	-1.64960800	-0.51546400
F	-3.26566200	-0.20413800	-0.07663800

Zero-point correction= 0.147866 (Hartree/Particle)

Thermal correction to Energy= 0.157499

Thermal correction to Enthalpy= 0.158443

Thermal correction to Gibbs Free Energy= 0.112310

Sum of electronic and zero-point Energies= -547.095010

Sum of electronic and thermal Energies= -547.085377

Sum of electronic and thermal Enthalpies= -547.084433

Sum of electronic and thermal Free Energies= -547.130566

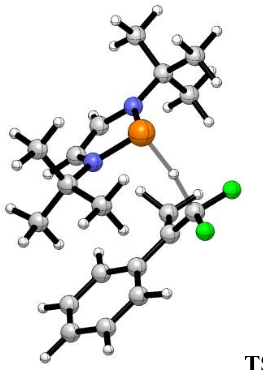

TS3

C	-3.80102300	-0.61682400	-1.91047300
C	-2.57260800	-1.18274700	-1.57104600
C	-2.19611200	-1.38614900	-0.22377500
C	-3.13207600	-0.99673200	0.76403600
C	-4.35272300	-0.42707200	0.41922300
C	-4.70155700	-0.22865200	-0.92024200
H	-4.04868100	-0.47521300	-2.95976900
H	-1.89587400	-1.46693600	-2.37097300
H	-2.91279000	-1.15501600	1.81341600
H	-5.04466600	-0.14277800	1.20847800
H	-5.65885600	0.21296000	-1.18300400
C	-0.90413600	-2.01326300	0.06847600
C	-0.19809500	-1.77506400	1.22585400
C	-0.12707400	-2.62810100	-1.06286800
H	0.27818400	-1.85705600	-1.74252200
H	0.71463800	-3.22061700	-0.69788100
H	-0.76527300	-3.28485700	-1.66435900
F	-0.81219900	-1.40414100	2.37687800
F	0.82489900	-2.60724300	1.56992600
C	1.60967600	0.55805300	-1.63022900
C	0.42585000	1.17821000	-1.43886300
H	2.00843300	0.20106700	-2.57071100
H	-0.29952200	1.43114700	-2.20045200
N	0.20039200	1.49480600	-0.09714800
N	2.34797400	0.43969000	-0.44823400
C	-0.80136400	2.49558300	0.35739500
C	-1.21208900	2.15227300	1.79076000
C	-0.17708300	3.89246800	0.29724900
C	-2.03736100	2.42928900	-0.54000600
H	-1.63095300	1.14088900	1.83983900
H	-0.36758800	2.21931100	2.48692600
H	-1.97513000	2.85932300	2.13196500
H	0.14559200	4.12368000	-0.72440100
H	-0.90614500	4.64900800	0.60940400

H	0.69355700	3.95502700	0.96039600
H	-2.81823600	3.06754500	-0.11361100
H	-1.83189500	2.79031700	-1.55297000
H	-2.41954200	1.40552100	-0.59739600
C	3.66057300	-0.24814000	-0.41251200
C	4.60172800	0.42724700	-1.41322200
C	4.24956700	-0.13129100	0.99298800
C	3.46601500	-1.72547900	-0.76470300
H	4.23305000	0.33766600	-2.44016600
H	4.71673100	1.49013500	-1.17417600
H	5.58692400	-0.04886200	-1.36772200
H	3.61976600	-0.62967400	1.74089400
H	5.22784900	-0.62164000	1.00891600
H	4.38689000	0.91611000	1.28413600
H	4.42827200	-2.24802300	-0.72872300
H	2.78585100	-2.19901700	-0.04856000
H	3.05023700	-1.84220300	-1.77187600
P	1.41872400	0.85898700	0.92074000
H	0.66480000	-0.57329600	1.14198200

Zero-point correction= 0.447019 (Hartree/Particle)

Thermal correction to Energy= 0.473187

Thermal correction to Enthalpy= 0.474131

Thermal correction to Gibbs Free Energy= 0.392486

Sum of electronic and zero-point Energies= -1391.076730

Sum of electronic and thermal Energies= -1391.050563

Sum of electronic and thermal Enthalpies= -1391.049619

Sum of electronic and thermal Free Energies= -1391.131263

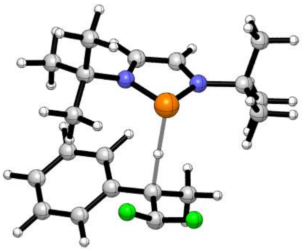

TS3′

C	-2.87581800	0.58196400	-2.03329800
C	-1.80541500	-0.16684900	-1.54889300
C	-2.00357400	-1.17654300	-0.59681600
C	-3.30951000	-1.42410100	-0.15111600
C	-4.38009300	-0.66868000	-0.62802000
C	-4.16903000	0.33946000	-1.56927200
H	-2.69639600	1.36194000	-2.76893400
H	-0.79892700	0.05508200	-1.89700400
H	-3.49143900	-2.21937100	0.56561600
H	-5.38417800	-0.87654000	-0.26767400

H	-5.00473600	0.92610400	-1.94119900
C	-0.82277600	-1.94981600	-0.08540100
C	-1.01230000	-2.73520800	1.06228700
C	0.09935600	-2.53481200	-1.14196000
H	0.43817400	-1.75296200	-1.82721400
H	0.97845900	-2.99544700	-0.68355000
H	-0.42912100	-3.29427700	-1.72928700
F	-1.60292800	-2.10172000	2.13602700
F	0.12426400	-3.32209700	1.57506700
C	1.88933200	1.27016400	-1.41543900
C	0.83608200	2.04313200	-1.08066100
H	2.46708900	1.32251500	-2.32862100
H	0.39650100	2.82605700	-1.68293200
N	0.30529400	1.71438100	0.16681500
N	2.21057400	0.34058900	-0.41978400
C	-0.62787800	2.57730300	0.94118900
C	-1.60324400	1.67797500	1.70470200
C	0.18632300	3.43416100	1.91690100
C	-1.41547800	3.48075300	-0.00606300
H	-2.21904000	1.09287900	1.01208500
H	-1.07742000	0.98729800	2.37653200
H	-2.26265800	2.29601600	2.32283400
H	0.90831100	4.05002500	1.36864100
H	-0.47498500	4.09655700	2.48661900
H	0.73398000	2.80259400	2.62592300
H	-2.17571100	4.01421100	0.57334900
H	-0.77606100	4.22999100	-0.48411200
H	-1.92291700	2.89533300	-0.78036100
C	3.50260100	-0.39335400	-0.42372200
C	4.64291300	0.62019500	-0.28653500
C	3.53122400	-1.36543700	0.75535700
C	3.64208600	-1.17962100	-1.73015800
H	4.65144900	1.32136800	-1.12829600
H	4.53309300	1.19075600	0.64263400
H	5.60659900	0.09960300	-0.26536300
H	2.68763300	-2.06603000	0.71789300
H	4.45517000	-1.95046500	0.71212500
H	3.51379500	-0.83810400	1.71541200
H	4.59686700	-1.71611200	-1.72987400
H	2.83446500	-1.91161400	-1.83085900
H	3.62993800	-0.52194700	-2.60519100
P	1.07510900	0.35069100	0.85065200
H	0.02352000	-0.84038000	0.34347900

Zero-point correction= 0.446764 (Hartree/Particle)

Thermal correction to Energy= 0.473259

Thermal correction to Enthalpy= 0.474203

Thermal correction to Gibbs Free Energy= 0.390169

Sum of electronic and zero-point Energies= -1391.052992

Sum of electronic and thermal Energies= -1391.026497

Sum of electronic and thermal Enthalpies= -1391.025553

Sum of electronic and thermal Free Energies= -1391.109588

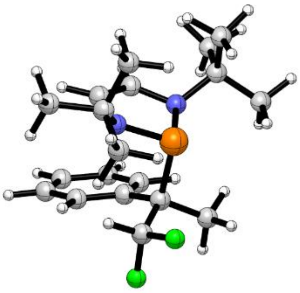

B

C	-0.92497400	0.97009900	-1.44984700
C	-0.65400800	-0.30034100	-1.77837000
H	-0.93605700	1.80104600	-2.14560300
H	-0.41722200	-0.64553800	-2.77835300
N	-0.59738000	-1.20627900	-0.68729700
N	-1.11586900	1.20970700	-0.06208700
C	-1.30560000	-2.51569600	-0.83310300
C	-0.92686500	-3.41765100	0.34140000
C	-2.82390500	-2.31332000	-0.86966100
C	-0.83911500	-3.18270600	-2.13032300
H	0.14954700	-3.62273000	0.34471300
H	-1.20352000	-2.97087900	1.30320100
H	-1.45616100	-4.37247700	0.25434500
H	-3.10045500	-1.61556000	-1.66890700
H	-3.33401000	-3.26588800	-1.05416800
H	-3.18764000	-1.91275100	0.08413400
H	-1.22258700	-4.20785100	-2.16634000
H	-1.20823200	-2.66278000	-3.01999700
H	0.25561700	-3.22210500	-2.17419000
C	-2.30553500	2.02691500	0.33772800
C	-3.60428800	1.24210900	0.12322200
C	-2.17017600	2.42261100	1.80762600
C	-2.32720700	3.30446700	-0.50677100
H	-3.66449300	0.86941100	-0.90632000
H	-3.66337100	0.38682800	0.80741800
H	-4.47391800	1.88394500	0.30636000
H	-1.31373500	3.08957600	1.95481600
H	-3.07454600	2.95172600	2.12725100
H	-2.04840600	1.54765600	2.45662200

H	-3.07122900	3.99430700	-0.09495500
H	-1.34903500	3.79938500	-0.48451600
H	-2.59974400	3.10803800	-1.54846800
P	-0.74019200	-0.26912000	0.77673400
C	2.63318700	2.43189900	-1.42424300
C	1.99966800	1.94427600	-0.28216300
C	1.89827100	0.56627300	-0.03799400
C	2.49247500	-0.30549200	-0.96603100
C	3.13274700	0.18042100	-2.10466200
C	3.19793800	1.55240300	-2.34757800
H	2.68435000	3.50490600	-1.59047600
H	1.56540800	2.65372100	0.41442500
H	2.45158300	-1.38113500	-0.82453500
H	3.57680100	-0.52064300	-2.80658000
H	3.69149800	1.93122900	-3.23852100
C	1.12911500	0.05844100	1.16480900
C	1.70063400	-1.26983500	1.63479000
C	1.13522300	1.04108100	2.34385000
H	0.65308500	0.59968100	3.22133300
H	2.16025600	1.31982700	2.61147700
H	0.59142400	1.95500500	2.09667900
F	3.00785400	-1.11492400	2.03908000
F	1.02953600	-1.72077000	2.74690700
H	1.67838700	-2.07762900	0.90387100

Zero-point correction= 0.456254 (Hartree/Particle)

Thermal correction to Energy= 0.481395

Thermal correction to Enthalpy= 0.482339

Thermal correction to Gibbs Free Energy= 0.403657

Sum of electronic and zero-point Energies= -1391.146580

Sum of electronic and thermal Energies= -1391.121440

Sum of electronic and thermal Enthalpies= -1391.120496

Sum of electronic and thermal Free Energies= -1391.199177

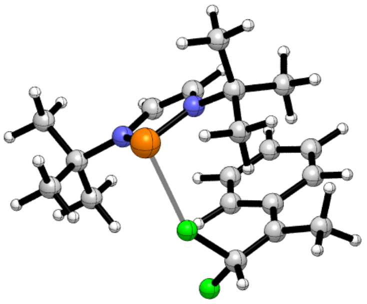

TS4

C	-1.73932000	1.89417300	-0.77074000
C	-2.50084400	0.79974500	-1.06283300
H	-1.65840300	2.80601000	-1.34728700
H	-3.14824300	0.66688900	-1.91979700

N	-2.35376500	-0.17992900	-0.12156100
N	-1.03863900	1.72260800	0.38965200
C	-3.08252700	-1.49009900	-0.21578900
C	-2.69709100	-2.36165200	0.97625200
C	-4.58240500	-1.19718300	-0.19202100
C	-2.66582400	-2.17617800	-1.51644700
H	-1.62314100	-2.58398300	0.98705100
H	-2.98311200	-1.90009900	1.92860400
H	-3.23173800	-3.31235400	0.89311900
H	-4.88733500	-0.59100100	-1.05066800
H	-5.12961200	-2.14412700	-0.23237100
H	-4.85717300	-0.67227100	0.72896100
H	-3.18785600	-3.13524100	-1.59229400
H	-2.92904100	-1.57673500	-2.39360800
H	-1.58720200	-2.36570500	-1.52386500
C	-0.10410200	2.77301700	0.91596300
C	-0.91308200	4.04504700	1.16737100
C	0.52146100	2.27871000	2.21705800
C	0.98627500	2.99133900	-0.13195500
H	-1.34387100	4.43772400	0.24111400
H	-1.72116200	3.85345000	1.88135500
H	-0.25089100	4.80968800	1.58533200
H	1.11565500	1.36984700	2.06163800
H	1.19503800	3.05520200	2.59093100
H	-0.23355900	2.09200100	2.98966400
H	1.70551100	3.72358600	0.24923800
H	1.51334900	2.05136800	-0.32873200
H	0.57468300	3.37445400	-1.07125900
P	-1.28897600	0.22869200	1.11216900
C	2.66214200	0.85192300	-2.62236400
C	3.20111600	0.23206100	-1.49586100
C	2.45189800	-0.67755500	-0.70542300
C	1.11142300	-0.90289500	-1.12626100
C	0.58008900	-0.27765600	-2.24927500
C	1.34588600	0.60920900	-3.01584000
H	3.28275100	1.53661600	-3.19707800
H	4.22943600	0.45510200	-1.22479600
H	0.48402600	-1.58641200	-0.56238600
H	-0.44871200	-0.48976800	-2.53337600
H	0.92458300	1.09367100	-3.89241600
C	3.03941000	-1.29712700	0.46535800
C	2.37781300	-2.24098700	1.23843200
C	4.34617900	-0.76208600	1.00597500
H	4.62259400	-1.28364700	1.92931500

H	5.18390900	-0.89225200	0.30505500
H	4.30447700	0.31362800	1.24194900
F	1.12487600	-1.63156600	2.21127100
F	1.56554200	-3.16419000	0.60684500
H	2.92176100	-2.72381200	2.04480500

Zero-point correction= 0.453035 (Hartree/Particle)

Thermal correction to Energy= 0.479213

Thermal correction to Enthalpy= 0.480158

Thermal correction to Gibbs Free Energy= 0.397824

Sum of electronic and zero-point Energies= -1391.107678

Sum of electronic and thermal Energies= -1391.081499

Sum of electronic and thermal Enthalpies= -1391.080555

Sum of electronic and thermal Free Energies= -1391.162889

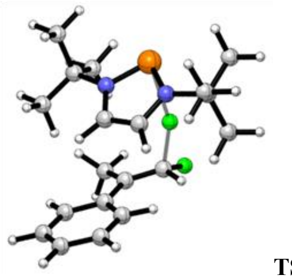

TS5

C	-0.49954100	1.18635400	-1.14029300
C	-1.46334100	0.29031000	-1.49817400
H	0.35630000	1.49342600	-1.72636000
H	-1.53126800	-0.25789700	-2.42866400
N	-2.40602600	0.14470000	-0.51662300
N	-0.72854600	1.70350600	0.10573300
C	-3.55451100	-0.81023800	-0.65583000
C	-2.98187200	-2.21256400	-0.86645200
C	-4.38989300	-0.77666200	0.62109500
C	-4.39980100	-0.36503500	-1.84884500
H	-2.40033300	-2.27638000	-1.79182700
H	-2.34337900	-2.49677100	-0.02393400
H	-3.80897000	-2.92641600	-0.93547100
H	-4.81074700	0.21843800	0.80631700
H	-5.22325600	-1.47531500	0.50392100
H	-3.80850800	-1.09467500	1.49448600
H	-5.25454400	-1.04098000	-1.95152700
H	-4.77437200	0.65255800	-1.69591600
H	-3.82852800	-0.39566200	-2.78203100
C	0.19919600	2.70236600	0.73241600
C	0.21628600	3.94995500	-0.15086600
C	-0.31327400	3.05195700	2.12648700
C	1.58737800	2.06762900	0.82784600
H	0.60609700	3.72995000	-1.14972000

H	-0.79158200	4.36721700	-0.24761400
H	0.86491100	4.70282600	0.30786300
H	-0.33580700	2.17348600	2.78212600
H	0.36885400	3.78205100	2.57148100
H	-1.31124500	3.50431600	2.09100000
H	2.27210900	2.77986900	1.29975700
H	1.55358100	1.15696500	1.43574600
H	1.98587700	1.81693500	-0.16161500
P	-2.08765900	1.06328700	0.84542100
C	4.72859800	0.14386200	-0.60733800
C	3.95269700	-0.67416700	0.21521200
C	2.69492200	-1.18559100	-0.19743400
C	2.29604700	-0.83446000	-1.51736700
C	3.06944500	-0.01570100	-2.32884400
C	4.29809700	0.49443000	-1.88606400
H	5.68170100	0.51396400	-0.23463900
H	4.32509800	-0.90979300	1.20787400
H	1.35836700	-1.21244900	-1.91760900
H	2.71196100	0.22575900	-3.32824400
H	4.90080700	1.13314900	-2.52540400
C	1.91354500	-2.02119500	0.69410900
C	0.54837400	-2.18513000	0.48555100
C	2.47870200	-2.39347200	2.04437800
H	1.79208800	-3.05234900	2.58508400
H	3.43919900	-2.92166600	1.96480100
H	2.65895700	-1.51726400	2.68932400
F	-0.02968100	-3.32078000	1.02609400
F	-0.39078100	-1.13044700	1.36728200
H	0.10137900	-1.97489100	-0.48219500

Zero-point correction= 0.453000 (Hartree/Particle)

Thermal correction to Energy= 0.479298

Thermal correction to Enthalpy= 0.480243

Thermal correction to Gibbs Free Energy= 0.396948

Sum of electronic and zero-point Energies= -1391.107631

Sum of electronic and thermal Energies= -1391.081333

Sum of electronic and thermal Enthalpies= -1391.080389

Sum of electronic and thermal Free Energies= -1391.163683

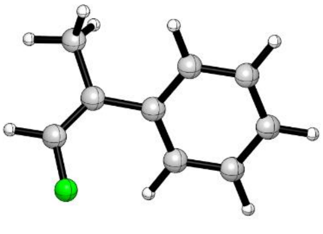

***Z*-4c**

C	-2.28322600	0.98916400	-0.44932600

C	-0.90550900	1.19828400	-0.40372700
C	-0.04099100	0.19289700	0.05468100
C	-0.59840000	-1.01945500	0.49110500
C	-1.97600800	-1.22495000	0.45141000
C	-2.82438600	-0.22390300	-0.02373800
H	-2.93443800	1.77796300	-0.81628400
H	-0.50173400	2.14980500	-0.73872400
H	0.04812800	-1.79938800	0.88196800
H	-2.38704900	-2.16818900	0.80111400
H	-3.89834200	-0.38571900	-0.05442000
C	1.42359800	0.43947200	0.11104000
C	2.33244600	-0.49745300	-0.15697300
C	1.91235100	1.81848200	0.47762900
H	3.00001800	1.83000400	0.59249700
H	1.64940400	2.55033300	-0.29387400
H	1.45911000	2.15198800	1.41774500
F	1.99074900	-1.75242500	-0.55393100
H	3.40891700	-0.37020600	-0.11724700

Zero-point correction= 0.155447 (Hartree/Particle)

Thermal correction to Energy= 0.164346

Thermal correction to Enthalpy= 0.165290

Thermal correction to Gibbs Free Energy= 0.120929

Sum of electronic and zero-point Energies= -447.873984

Sum of electronic and thermal Energies= -447.865084

Sum of electronic and thermal Enthalpies= -447.864140

Sum of electronic and thermal Free Energies= -447.908502

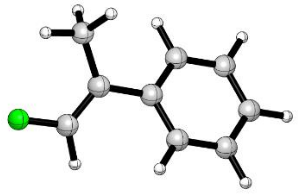

***E*-4c**

C	2.41880800	1.03329600	-0.30623100
C	1.03134400	1.16409300	-0.25821700
C	0.21538800	0.06021200	0.03093800
C	0.83159500	-1.17290300	0.29690800
C	2.21752800	-1.30388000	0.24422200
C	3.01834400	-0.20148000	-0.05902000
H	3.03113600	1.90051400	-0.53845400
H	0.58121100	2.13306700	-0.45643900
H	0.22419100	-2.03226100	0.56860000
H	2.67389000	-2.26736800	0.45537000
H	4.09958600	-0.30281900	-0.09198600
C	-1.26274600	0.21687700	0.06867800

C	-2.02994700	-0.81057000	-0.29225100
C	-1.84788600	1.54031800	0.48487800
H	-2.92000500	1.45734500	0.67533400
H	-1.35782000	1.90647700	1.39323300
H	-1.70275200	2.29626400	-0.29544300
F	-3.38638400	-0.71833400	-0.25791000
H	-1.70655600	-1.78200500	-0.64845700

Zero-point correction= 0.155672 (Hartree/Particle)

Thermal correction to Energy= 0.164539

Thermal correction to Enthalpy= 0.165484

Thermal correction to Gibbs Free Energy= 0.121488

Sum of electronic and zero-point Energies= -447.874892

Sum of electronic and thermal Energies= -447.866024

Sum of electronic and thermal Enthalpies= -447.865080

Sum of electronic and thermal Free Energies= -447.909075

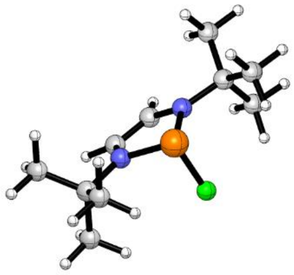

1-F

C	-0.67205600	1.44233400	0.59624700
C	0.67206700	1.44233100	0.59624500
H	-1.32636800	2.20252100	1.00080600
H	1.32639000	2.20251800	1.00079100
N	1.19583500	0.30604400	-0.04340600
N	-1.19583300	0.30605400	-0.04341100
C	2.66019100	0.09610400	-0.16245600
C	2.92982700	-1.15541700	-0.99721500
C	3.27932200	1.30839800	-0.86436400
C	3.26273400	-0.07683800	1.23572000
H	2.53734000	-2.05959000	-0.51715200
H	2.49316500	-1.06848500	-1.99847700
H	4.01169600	-1.28171800	-1.10574200
H	3.15173200	2.22510700	-0.27957700
H	4.35366000	1.14562200	-1.00081200
H	2.82196700	1.45642600	-1.84881000
H	4.34525400	-0.22982800	1.16343900
H	3.08465700	0.81035200	1.85315200
H	2.82150400	-0.94453200	1.73842600
C	-2.66019000	0.09610900	-0.16245400
C	-3.27931900	1.30829100	-0.86456200
C	-2.92980900	-1.15553700	-0.99702900

C	-3.26275500	-0.07662200	1.23574000
H	-3.15178300	2.22509100	-0.27990800
H	-2.82192700	1.45618500	-1.84901200
H	-4.35364600	1.14546600	-1.00103300
H	-2.53736900	-2.05964300	-0.51680300
H	-4.01167600	-1.28183300	-1.10559300
H	-2.49308800	-1.06877600	-1.99828000
H	-4.34527800	-0.22959300	1.16347000
H	-2.82155300	-0.94425500	1.73857500
H	-3.08465600	0.81065300	1.85304400
P	-0.00000300	-0.85374000	-0.35925900
F	-0.00000700	-1.70724400	1.14343100

Zero-point correction= 0.295975 (Hartree/Particle)
Thermal correction to Energy= 0.312508
Thermal correction to Enthalpy= 0.313453
Thermal correction to Gibbs Free Energy= 0.253493
Sum of electronic and zero-point Energies= -943.285001
Sum of electronic and thermal Energies= -943.268467
Sum of electronic and thermal Enthalpies= -943.267523
Sum of electronic and thermal Free Energies= -943.327482

Supplementary References

1. Zhang, J., Yang, J.-D. & Cheng, J.-P. A Nucleophilicity Scale for the Reactivity of Diazaphospholenium Hydrides: Structural Insights and Synthetic Applications. *Angew. Chem. Int. Ed.* **58**, 5983-5987 (2019).
2. Frisch, M. J. T., G. W.; Schlegel, H. B.; Scuseria, G. E.;Robb, M. A.; Cheeseman, J. R.; Scalmani, G.; Barone, V.; Mennucci,B.; Petersson, G. A.; Nakatsuji, H.; Caricato, M.; Li, X.; Hratchian, H.P.; Izmaylov, A. F.; Bloino, J.; Zheng, G.; Sonnenberg, J. L.; Hada, M.;Ehara, M.; Toyota, K.; Fukuda, R.; Hasegawa, J.; Ishida, M.; Nakajima,T.; Honda, Y.; Kitao, O.; Nakai, H.; Vreven, T.; Montgomery, J. A., Jr.;J. E, P.; Ogliaro, F.; Bearpark, M.; Heyd, J. J.; Brothers, E.; Kudin, K.N.; Staroverov, V. N.; Keith, T.; Kobayashi, R.; Normand, J.;Raghavachari, K.; Rendell, A.; Burant, J. C.; Iyengar, S. S.; Tomasi,J.; Cossi, M.; Rega, N.; Millam, J. M.; Klene, M.; Knox, J. E.; Cross, J.B.; Bakken, V.; Adamo, C.; Jaramillo, J.; Gomperts, R.; Stratmann, R.E.; Yazyev, O.; Austin, A. J.; Cammi, R.; Pomelli, C.; Ochterski, J. W.;Martin, R. L.; Morokuma, K.; Zakrzewski, V. G.; Voth, G. A.; Salvador,P.; Dannenberg, J. J.; Dapprich, S.; Daniels, A. D.; Farkas, O.;Foresman, J. B.; Ortiz, J. V.; Cioslowski, J.; Fox, D. J.Gaussian 09,Revision D.01, Gaussian, Inc., Wallingford, CT, 2013.
3. Zhao, Y. & Truhlar, D. G. Density Functionals with Broad Applicability in Chemistry. *Acc. Chem. Res.* **41**, 157-167 (2008).
4. Zhao, Y. & Truhlar, D. G. Applications and Validations of the Minnesota Density Functionals. *Chem. Phys. Lett.* **502**, 1-13 (2011).
5. Marenich, A. V., Cramer, C. J. & Truhlar, D. G. Universal Solvation Model Based on Solute Electron Density and on a Continuum Model of the Solvent Defined by the Bulk Dielectric Constant and Atomic Surface Tensions. *J. Phys. Chem. B* **113**, 6378-6396 (2009).
6. Yao, C., Wang, S., Norton, J. & Hammond, M. Catalyzing the Hydrodefluorination of CF_3-Substituted

Alkenes by $PhSiH_3$. H• Transfer from a Nickel Hydride. *J. Am. Chem. Soc.* **142**, 4793-4799 (2020).

7. Engman, M., Cheruku, P., Tolstoy, P., Bergquist, J., Völker, S. F. & Andersson, P. G. Highly Selective Iridium-Catalyzed Asymmetric Hydrogenation of Trifluoromethyl Olefins: A New Route to Trifluoromethyl- Bearing Stereocenters. *Adv. Synth. Catal.* **351**, 375-378 (2009).
8. Guo, Y.-Q., Wang, R., Song, H., Liu, Y. & Wang, Q. Visible-Light-Induced Deoxygenation/Defluorination Protocol for Synthesis of γ,γ-Difluoroallylic Ketones. *Org. Lett.* **22**, 709-713 (2020).
9. Hamlin, T. A., Kelly, C. B., Cywar, R. M. & Leadbeater, N. E. Methylenation of Perfluoroalkyl Ketones using a Peterson Olefination Approach. *J. Org. Chem.* **79**, 1145-1155 (2014).
10. Phelan, J. P.*, et al.* Redox-Neutral Photocatalytic Cyclopropanation via Radical/Polar Crossover. *J. Am. Chem. Soc.* **140**, 8037-8047 (2018).
11. Chen, F., Xu, X., He, Y., Huang, G. & Zhu, S. NiH-Catalyzed Migratory Defluorinative Olefin Cross-Coupling: Trifluoromethyl-Substituted Alkenes as Acceptor Olefins to Form gem-Difluoroalkenes. *Angew. Chem. Int. Ed.* **59**, 5398-5402 (2020).
12. Li, Y.*, et al.* Palladium-catalyzed Suzuki–Miyaura Reaction of Fluorinated Vinyl Chloride: A New Approach for Synthesis α and α,β-Trifluoromethylstyrenes. *Tetrahedron* **72**, 5684-5690 (2016).
13. Fujita, T.*, et al.* Flash Generation and Borylation of 1-(Trifluoromethyl)vinyllithium toward Synthesis of α-(Trifluoromethyl)styrenes. *J. Fluorine Chem.* **207**, 72-76 (2018).
14. Liu, Y., Zhou, Y., Zhao, Y. & Qu, J. Synthesis of gem-Difluoroallylboronates via $FeCl_2$-Catalyzed Boration/β-Fluorine Elimination of Trifluoromethyl Alkenes. *Org. Lett.* **19**, 946-949 (2017).
15. Feng, S., Tang, Y., Yang, C., Shen, C. & Dong, K. Synthesis of Enantioenriched α,α-Difluoro-β-arylbutanoic Esters by Pd-Catalyzed Asymmetric Hydrogenation. *Org. Lett.* **22**, 7508-7512 (2020).
16. Lang, S. B., Wiles, R. J., Kelly, C. B. & Molander, G. A. Photoredox Generation of Carbon-Centered Radicals Enables the Construction of 1,1-Difluoroalkene Carbonyl Mimics. *Angew. Chem. Int. Ed.* **56**, 15073-15077 (2017).
17. Trost, B. M. & Debien, L. Palladium-Catalyzed Trimethylenemethane Cycloaddition of Olefins Activated by the σ-Electron-Withdrawing Trifluoromethyl Group. *J. Am. Chem. Soc.* **137**, 11606-11609 (2015).
18. Wang, X.*, et al.* Pd–Carbene Migratory Insertion: Application to the Synthesis of Trifluoromethylated Alkenes and Dienes. *Chem. Eur. J.* **20**, 961-965 (2014).
19. Liu, Z., Tu, X.-S., Guo, L.-T. & Wang, X.-C. Aluminum-Catalyzed Tunable Halodefluorination of Trifluoromethyl- and Difluoroalkyl-Substituted Olefins. *Chem. Sci.* **11**, 11548-11553 (2020).
20. Lu, X., Wang, X.-X., Gong, T.-J., Pi, J.-J., He, S.-J. & Fu, Y. Nickel-Catalyzed Allylic Defluorinative Alkylation of Trifluoromethyl Alkenes with Reductive Decarboxylation of Redox-Active Esters. *Chem. Sci.* **10**, 809-814 (2019).
21. Krishnamoorthy, S., Kothandaraman, J., Saldana, J. & Prakash, G. K. S. Direct Difluoromethylenation of Carbonyl Compounds by Using $TMSCF_3$: The Right Conditions. *Eur. J. Org. Chem.* **2016**, 4965-4969 (2016).
22. Zhang, Z., Yu, W., Wu, C., Wang, C., Zhang, Y. & Wang, J. Reaction of Diazo Compounds with Difluorocarbene: An Efficient Approach towards 1,1-Difluoroolefins. *Angew. Chem. Int. Ed.* **55**, 273-277 (2016).
23. Hu, M., Ni, C., Li, L., Han, Y. & Hu, J. gem-Difluoroolefination of Diazo Compounds with $TMSCF_3$ or $TMSCF_2Br$: Transition-Metal-Free Cross-Coupling of Two Carbene Precursors. *J. Am. Chem. Soc.* **137**, 14496-14501 (2015).
24. Tian, H., Shimakoshi, H., Imamura, K., Shiota, Y., Yoshizawa, K. & Hisaeda, Y. Photocatalytic Alkene Reduction by A B_{12}–TiO_2 Hybrid Catalyst Coupled with C–F Bond Cleavage for gem-Difluoroolefin

Synthesis. *Chem. Commun.* **53**, 9478-9481 (2017).

25. Loska, R. & Bukowska, P. A Three-Component Synthesis of Aryl(heteroaryl)acylamides. *Org. Biomol. Chem.* **13**, 9872-9882 (2015).

26. Qiao, Y., Si, T., Yang, M.-H. & Altman, R. A. Metal-Free Trifluoromethylation of Aromatic and Heteroaromatic Aldehydes and Ketones. *J. Org. Chem.* **79**, 7122-7131 (2014).

27. Hu, M., He, Z., Gao, B., Li, L., Ni, C. & Hu, J. Copper-Catalyzed gem-Difluoroolefination of Diazo Compounds with $TMSCF_3$ via C–F Bond Cleavage. *J. Am. Chem. Soc.* **135**, 17302-17305 (2013).

28. Greedy, B. & Gouverneur, V. Fluorodesilylation of Alkenyltrimethylsilanes: A New Route to Fluoroalkenes and Difluoromethyl-Substituted Amides, Alcohols or Ethers. *Chem. Commun.*, 233-234 (2001).

29. Poutrel, P., Pannecoucke, X., Jubault, P. & Poisson, T. Stereoselective Synthesis of Terminal Monofluoroalkenes from Trifluoromethylated Alkenes. *Org. Lett.* **22**, 4858-4863 (2020).

30. Liu, Q., Shen, X., Ni, C. & Hu, J. Stereoselective Carbonyl Olefination with Fluorosulfoximines: Facile Access to Z or E Terminal Monofluoroalkenes. *Angew. Chem. Int. Ed.* **56**, 619-623 (2017).
